# Integrating computed tomography and biopsy images to predict chemotherapy response in gastric cancer

**DOI:** 10.3389/fonc.2025.1666358

**Published:** 2025-10-21

**Authors:** Shenyan Zhang, Tao Luo, Kaikai Wei, Bochen Lai, Yuheng Luo, Yi Lin, Lei Lian, Yonghe Chen

**Affiliations:** ^1^ Department of Pathology, The Sixth Affiliated Hospital, Sun Yat-sen University, Guangzhou, China; ^2^ Department of Gastrointestinal Surgery, The First Affiliated Hospital, Sun Yat-sen University, Guangzhou, China; ^3^ Department of Radiology, The Sixth Affiliated Hospital, Sun Yat-sen University, Guangzhou, China; ^4^ Department of General Surgery, The Sixth Affiliated Hospital, Sun Yat-sen University, Guangzhou, China; ^5^ Guangdong Provincial Key Laboratory of Colorectal and Pelvic Floor Diseases, The Sixth Affiliated Hospital, Sun Yat-sen University, Guangzhou, China; ^6^ Biomedical Innovation Center, The Sixth Affiliated Hospital, Sun Yat-sen University, Guangzhou, China; ^7^ Bioinformatics Faculty, School of Life and Health Sciences, The Chinese University of Hong Kong, Shenzhen, China

**Keywords:** advanced gastric cancer, neoadjuvant chemotherapy, multimodal, pathological complete response, machine learning (ML)

## Abstract

**Aims:**

To predict pathological complete response to neoadjuvant chemotherapy in advanced gastric cancer by integrating multimodal radiomic and pathomic data.

**Methods:**

Eligible patients with advanced gastric cancer underwent neoadjuvant chemotherapy followed by radical gastrectomy. We collected pre-treatment venous-phase computed tomography (CT) scans and whole-slide H&E-stained gastroscopic biopsy sections for feature extraction. Three models were constructed: a unimodal radiomic model, a unimodal pathomic model, and a multimodal model combining both feature types. Model performance was evaluated using the area under the curve (AUC).

**Findings:**

Our study included 295 AGC patients who received NAC and radical surgery between February 2013 and September 2022 (236 in the training cohort, 59 in the validation cohort). A total of 42 patients (14.2%) achieved pCR. We extracted 615 radiomic and 548 pathomic features. The unimodal radiomic model (10 selected features) achieved an AUC of 0.672, while the pathomic model (13 selected features) achieved an AUC of 0.806. The multimodal model, constructed with 22 features (12 radiomic, 10 pathomic), achieved the highest AUC of 0.814. Decision curve analysis confirmed the multimodal model’s superior predictive efficacy compared to the unimodal models, highlighting the synergistic potential of combining radiomic and pathomic features.

**Conclusion:**

By integrating pathological images and CT features, we can maximize the utilization of pre-treatment information and enhance the accuracy of NAC prediction in AGC.

## Highlights

- Developed and validated a multimodal model to predict pathological complete response (pCR) to neoadjuvant chemotherapy (NAC) in advanced gastric cancer (AGC).
- Proved that integrating pathological image and CT features can maximize the utilization of pre-treatment information and enhance the accuracy of prediction.
- The model requires only easily obtainable data (biopsy pathology slides and pre-treatment CT scans), making it totally non-invasive and practical for clinical application.

## Introduction

Gastric cancer is the fifth most common malignancy in the world and the third leading cause of cancer-related death (1). The majority of patients are diagnosed at an advanced stage with a poor prognosis (2). Neoadjuvant chemotherapy (NAC) with subsequent radical gastrectomy is an important treatment modality for advanced gastric cancer (AGC) (3–6). NAC could potentially downstage tumors, eliminate micro-metastatic sites, thus improving curative resection rate and short-term treatment outcome (7). However, due to the great heterogeneity of gastric cancer, the response to NAC varies greatly (8, 9). Patients with tumors that were resistant to NAC were prone to the risk of tumor progression during chemotherapy, hindering their only chance for surgical curation (10). Therefore, treatment strategies shall be individualized based on the sensitivity to chemotherapy. Pathological complete response (pCR), namely no tumor cells remain in the primary site and dissected lymph nodes, is an important hallmark of chemo-sensitivity (11). Evidence showed that achieving pCR greatly benefit the patient by improving the survival significantly, making patients with the potential of achieving pCR the best subgroup for NAC intervention before surgery. However, pCR can only be confirmed after the resection surgery by thorough pathological examination. Thus, to differentiate patient with high sensitivity to NAC, a prediction model based on pre-intervention data is warranted.

Previous efforts have been made to explore biological markers based on genetic or serological information to predict pCR. However, these approaches have never been widely used in clinical practice due to the excessive cost and potential trauma for the patients. In 2021, we found that by analyzing the pre-intervention CT images of the tumor with radiomic method (high-throughput quantitative data from medical images), we can build a non-invasive, low-cost model to predict the response to NAC (12). More recently, pathomic analysis of pathological whole slide images has emerged as a powerful tool for characterizing tumor biology and predicting clinical outcomes (13–21). Thus, in this study, we intended to maximize the utility of pre-intervention information, by combining the high-quantity extracted from CT images and pathological slide images, we aimed to build a Radiomic-Pathomic multimodal model for predicting pCR in advanced gastric cancer patients.

## Methods

### Study population and data collection

This study was approved by the ethics committee of the Sixth Affiliated Hospital, Sun Yat-sen University and registered at https://www.clinicaltrials.gov/ (NCT06451393). We reviewed the gastric cancer database of our institution and included patients according to the following criteria:

**Inclusion criteria:**

➢ Histologically confirmed adenocarcinoma of the stomach or esophagogastric junction.
➢ Received NAC and radical gastrectomy.
➢ Received abdominal multidetector computed tomography (CT) inspection, gastroscope, and tumor tissue biopsy before any intervention started.

**Exclusion criteria:**

➢ CT unassessable by The Response Evaluation Criteria in Solid Tumors Version 1.1 (RECIST 1.1) (22).
➢ Insufficient data.

All available pre-intervention clinical information was retrieved from the database, including sex, age, tumor location, adenocarcinoma differentiation, Lauren type, and tumor staging information according to the staging system of the AJCC 8th edition (23).

### Extraction of radiomic and pathomic features, and determination of pathological response

**Figure 1:**
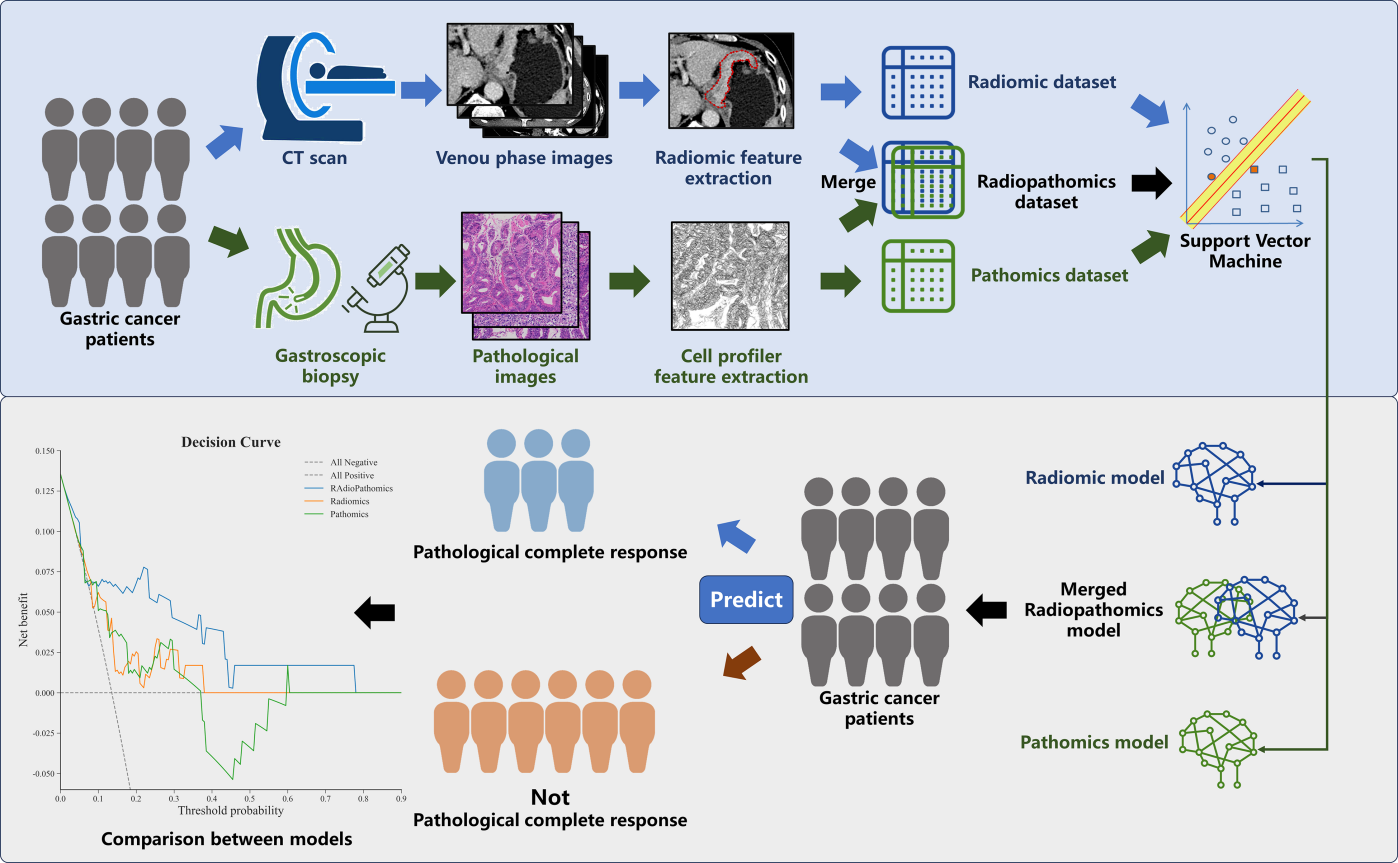
Workflow of the study. Patients underwent enhanced computed tomography (CT) scans and gastroscopy with primary lesion biopsies prior to intervention. Radiomics features were extracted from delineated tumor regions within venous phase CT images, while pathomics features were extracted from tumor cell regions on hematoxylin-eosin-stained pathological slides. Separate unimodal models were established using either radiomic or pathomic datasets to predict pathological complete response (pCR). Additionally, a multimodal model integrating both radiomic and pathomic features was constructed. Model prediction performance was evaluated and compared in a validation cohort.

The overall workflow of this study is illustrated in **Figure 1**. We retrieved venous-phase contrast-enhanced abdominal CT images from the picture archiving and communication system. The region of interest (ROI) was delineated at the largest cross section of the primary tumor lesions by a senior licensed radiologist. Delineations were strictly confined within the tumor border. Radiomic features of the ROI were extracted using the ‘pyradiomics’ package in the Python programming language ver. 3.7.0 (Python Software Foundation, Virginia, USA; www.python.org). The extracted features were listed in (**Supplementary Material 1**), marked as the Radiomic dataset.

The most typical tumor regions on the whole pathological slide images were sectioned into small patches with a size of approximately 512*512 pixels. These sections are then processed using CellProfiler (version 2.2.1, https://cellprofiler.org/) for nuclear feature extraction. Extracted features were listed in (**Supplementary Material 2**), marked as the Pathomic dataset.

For pathological response assessment, all resection specimens were examined by two senior pathologists. A pathological complete response (pCR) was defined as no viable cells remained in the primary tumor lesions and the dissected lymph nodes.

### Statistical analysis

All statistical analyses were performed by either Python programming language ver. 3.7.0 (Python Software Foundation, Virginia, USA; www.python.org), or R software version 3.6.1 (The R Foundation for Statistical Computing, Vienna, Austria; www.r-project.org). P-values<0.05 were identified as statistically significant.

### Development, validation, and comparison of models

The total cohort was randomly divided into a training cohort (n=236) and a validation cohort (n=59) according to a preset 4:1 ratio. The datasets of the training cohort was used to build the predictions models. Models were developed and validated in in 4 steps: Step 1, the correlations between the extracted features and pathological complete response were tested by univariate analysis, features with a P-value<0.05 were selected; Step 2, the machine learning algorithm of the least absolute shrinkage and selection operator (LASSO) method was used to reduce data dimensionalities, and features with a nonzero coefficient were further selected; Step 3, the selected features were combined by the Light Gradient Boosting Machine (LGBM) algorithm or the support vector machine (SVM) algorithm to build the prediction model. Step 4, the models were validated by the receiver operating characteristic (ROC) method to calculate the area under curve (AUC). The radiomic features were used to build the radiomic model, while the pathomic model was built by the pathomic dataset. The radio-pathomic multimodal model was built by the dataset merging the radiomic and pathomic features. Decision curve analysis was conducted to compare the predictive efficacy of the multimodal model and the singular model based on the radiomic or pathomic features alone. This work is reported in line with the STROCSS criteria (24).

## Results

### Patient characteristics

From February 2013 to September 2022, 295 patients who received NAC and radical tumor resection surgery were enrolled in the study. Patient characteristics in the training and validation cohorts are depicted in **Table 1**. The majority of patients were elderly males, with most of the lesions being poorly differentiated adenocarcinoma (67.8%, 200/295) in advanced clinical stages of T3-T4 and radiologically suspicious lymph node metastasis (97.3%, 287/295), cases were randomly assigned to a training cohort (n=236) for prediction model construction and a validation cohort (n=59) for model validation according to a preset 4:1 ratio. The demographic characteristics were similar in both cohorts, as shown in **Table 1**.

**Table 1:** Patient characteristics in the training and validation cohort.

Characteristic	All (n=295)	Validation cohort (n=59)	Training cohort (n=236)	p-value
Sex (%)				
*Male*	213 (72.2)	41 (69.5)	172 (72.9)	0.721
*Female*	82 (27.8)	18 (30.5)	64 (27.1)	
Age	64 [56, 70]	64 [57, 69]	64 [55, 70]	0.948
Location (%)				
*Upper*	133 (45.1)	27 (45.8)	106 (44.9)	0.948
*Lower*	110 (37.3)	21 (35.6)	89 (37.7)	
*Middle*	52 (17.6)	11 (18.6)	41 (17.4)	
Differentiation (%)				
*Poor*	200 (67.8)	39 (66.1)	161 (68.2)	0.419
*Moderate*	76 (25.8)	14 (23.7)	62 (26.3)	
*Well*	19 (6.4)	6 (10.2)	13 (5.5)	
Lauren type (%)				
*Diffuse*	145 (49.2)	29 (49.2)	116 (49.2)	0.196
*Mix*	67 (22.7)	9 (15.3)	58 (24.6)	
*Intestine*	83 (28.1)	21 (35.6)	62 (26.3)	
Clinical T stage (%)				
*T3*	206 (69.8)	41 (69.5)	165 (69.9)	0.667
*T4a*	71 (24.1)	13 (22.0)	58 (24.6)	
*T4b*	18 (6.1)	5 (8.5)	13 (5.5)	
Clinical N stage (%)				
*N0*	8 (2.7)	1 (1.7)	7 (3.0)	0.608
*N1*	94 (31.9)	19 (32.2)	75 (31.8)	
*N2*	138 (46.8)	31 (52.5)	107 (45.3)	
*N3*	55 (18.6)	8 (13.6)	47 (19.9)	
Regimen (%)				
*FLOT*	141(47.8)	23(39.0)	118(50.0)	0.182
*SOX*	108(36.6)	23(39.0)	85(36.0)	
*XELOX*	22(7.5)	6(10.2)	16(6.8)	
*FOLFOX*	15(5.1)	6(10.2)	9(3.8)	
*Others*	9(3.1)	1(1.7)	8(3.4)	
Cycles	4 [3, 4]	4 [3, 4.50]	4 [3, 4]	0.222
Resection extend (%)				
*Distal*	114 (38.6)	23 (39.0)	91 (38.6)	0.957
*Total*	169 (57.3)	34 (57.6)	135 (57.2)	
*Proximal*	12 (4.1)	2 (3.4)	10 (4.2)	
Laparoscopy surgery (%)				
*Yes*	269 (91.2)	53 (89.8)	216 (91.5)	0.878
*No*	26 (8.8)	6 (10.2)	20 (8.5)	
R0 resection (%)				
*R0*	273(92.5)	53(89.8)	220(93.2)	0.301
*R1*	19(6.4)	6(10.2)	13(5.5)	
*R2*	3(1.0)	0(0.0)	3(1.3)	
Pathological complete response (%)				
*Not pCR*	253(85.8)	51(86.4)	202(85.6)	1
*pCR*	42(14.2)	8(13.6)	34(14.4)	
Pathological T stage (%)				
*T0*	43(14.6)	8(13.6)	35(14.8)	0.69
*T1*	27(9.2)	4(6.8)	23(9.7)	
*T2*	34(11.5)	7(11.9)	27(11.4)	
*T3*	166(56.3)	32(54.2)	134(56.8)	
*T4a*	20(6.8)	6(10.2)	14(5.9)	
*T4b*	5(1.7)	2(3.4)	3(1.3)	
Pathological N stage (%)				
*N0*	144 (48.8)	26 (44.1)	118 (50.0)	0.83
*N1*	57 (19.3)	13 (22.0)	44 (18.6)	
*N2*	46 (15.6)	9 (15.3)	37 (15.7)	
*N3*	48 (16.3)	11 (18.6)	37 (15.7)	
Harvested Lymph Nodes	32 [23, 41]	31 [23.50, 41]	32 [23, 41]	0.896

### Neoadjuvant chemotherapy and pathological findings

Enrolled patients received a median of 4 cycles of NAC. Nearly half of the patients (47.8%, 141/295) received FLOT regimen with docetaxel, oxaliplatin and fluorouracil. Most lesions were resected through laparoscopy (91.2%, 269/295). In the final pathological analysis, a total of 42 patients (14.2%) achieved pathological complete responses.

### Radiomic unimodal model development and validation

**Figure 2:**
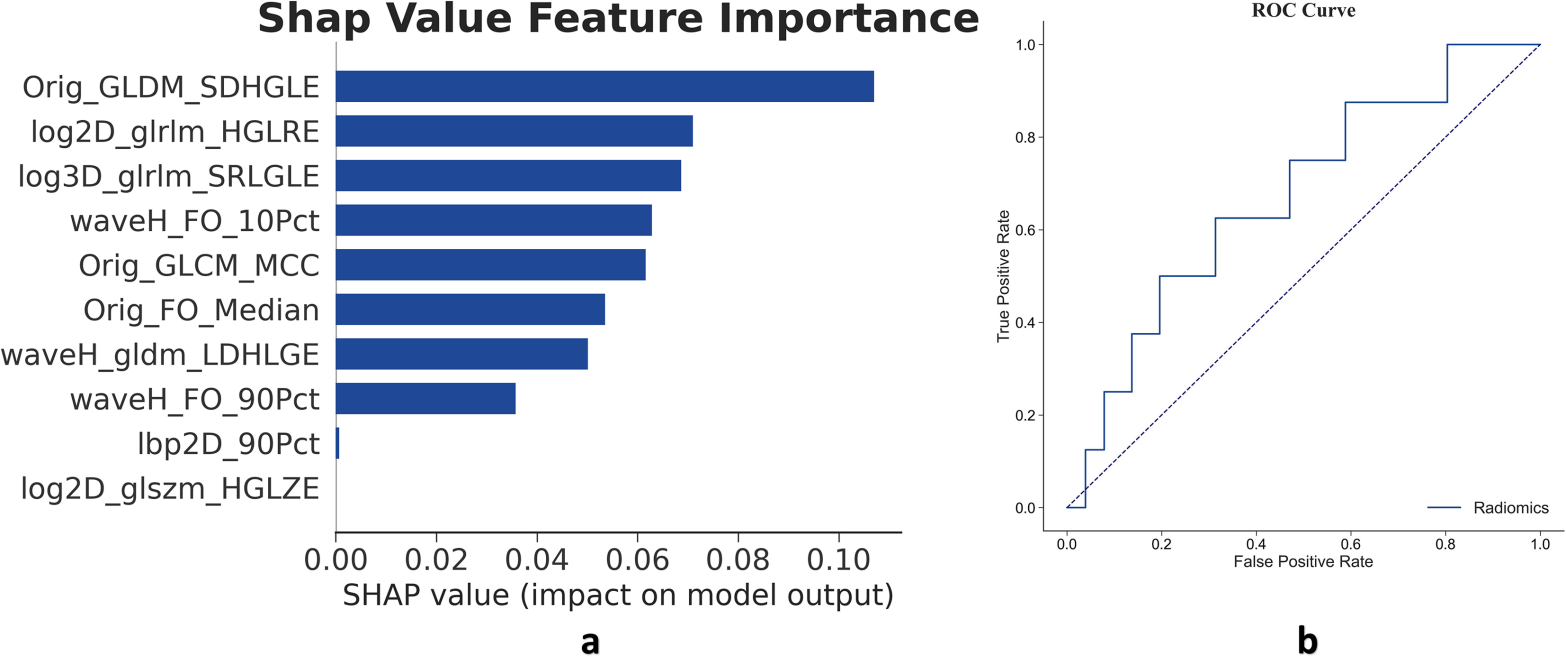
Features and validation of the radiomic unimodal. **(a)** The radiomic unimodal model constructed with 10 features selected from the 615 extracted from the tumor region on venous-phase CT images. Bar lengths represent the weight of each feature in the model. **(b)** The model achieved an AUC of 0.672 in the validation cohort.

A total of 615 radiomic features were extracted from the tumor region on the venous phase of the CT images. Univariate analysis identified 308 features with statistically significant differences (P-value < 0.05) between the pCR and non-pCR subgroups. Through binary LASSO regression (**Figure 2**), 10 features were selected and used to build a radiomic unimodal model (**Figure 2a**) employing a Light Gradient Boosting Machine (LGBM) algorithm. Model efficacy was tested in the validation cohort, yielding an AUC of 0.672 on the ROC curve (**Figure 2b**). The details of the radiomic features in the training and validation cohort were depicted in (**Supplementary Materials 1**, **3**).

### Pathomic unimodal model development and validation

**Figure 3:**
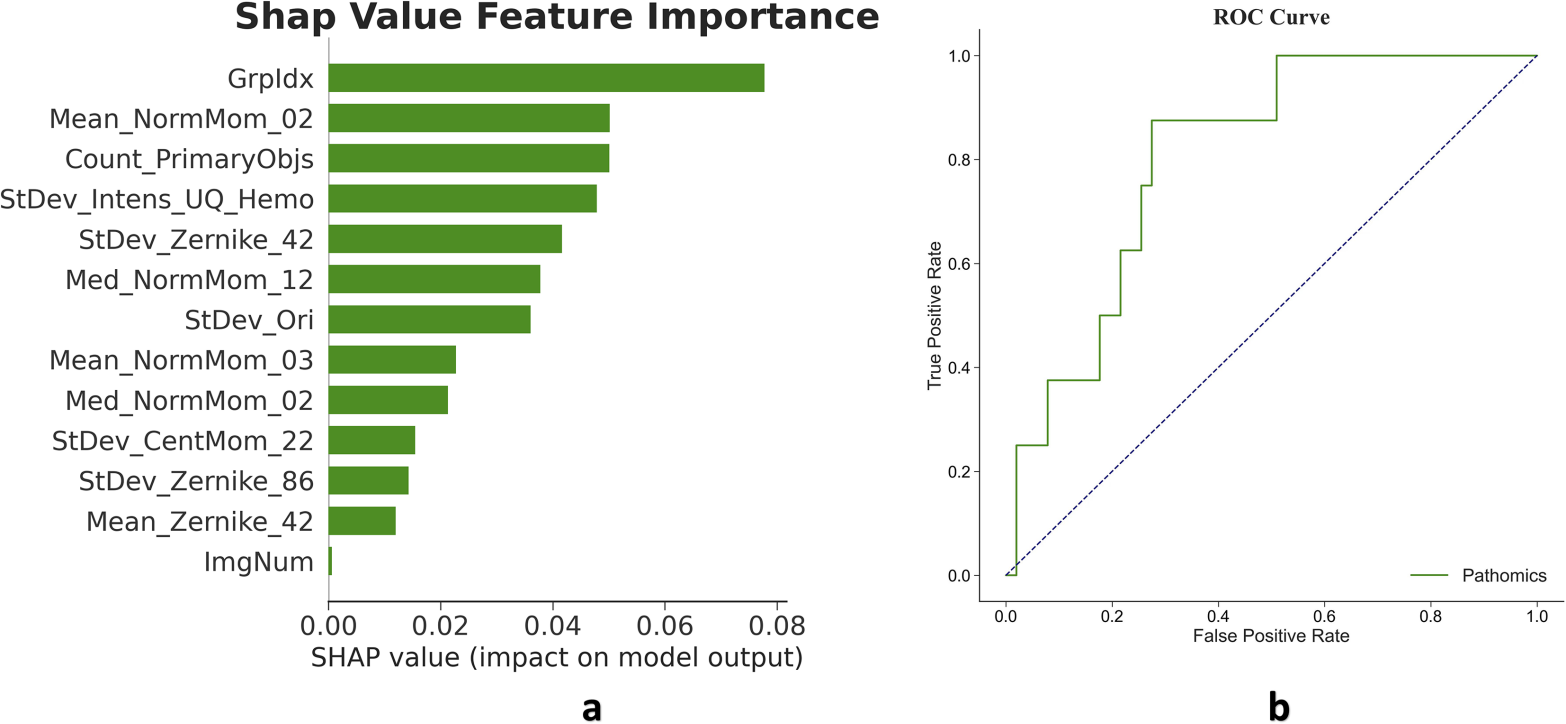
Features and validation of the pathomic unimodal. **(a)** The pathomic unimodal model includes 13 features of the total 548 extracted features from the tumor region on selected tumor region of the whole slide images. **(b)** The model achieved an AUC of 0.806 in the validation cohort.

The tumor region in whole-slide images was divided into 512x512 pixel patches. A total of 548 features were extracted from these patches, with 205 features showing statistically significant differences (P-value < 0.05) between pCR and non-pCR subgroups. Binary LASSO regression was used to select 13 features, which formed the basis of a pathomic unimodal model built with the LGBM algorithm (**Figure 3**). When tested in the validation cohort, this model achieved an AUC of 0.806 on the ROC curve, demonstrating stronger discriminative power compared to the radiomic model. The details of the pathomic features in the training and validation cohort were depict in (**Supplementary Materials 2**, **4**).

### Radio-pathomic multimodal model development and validation

**Figure 4:**
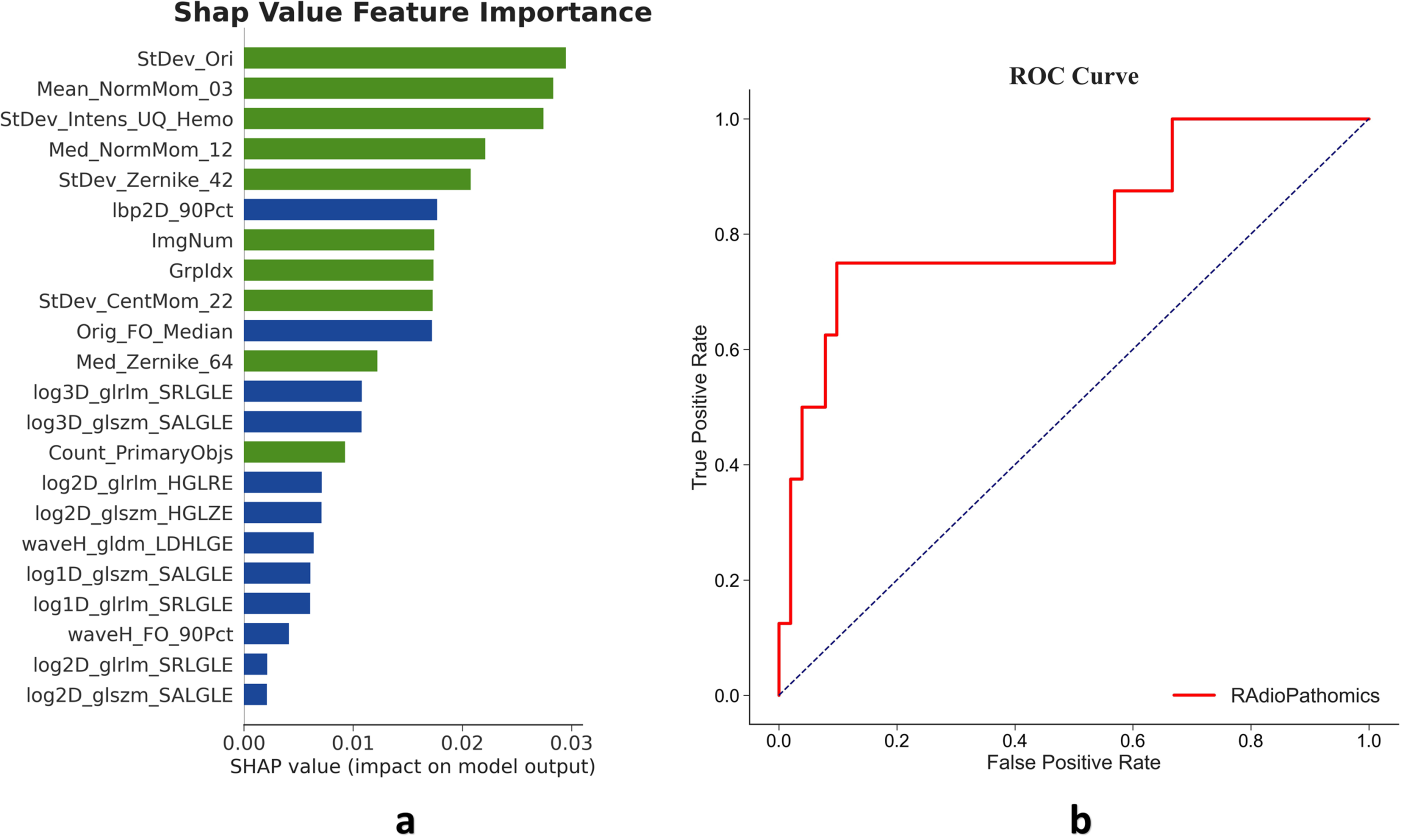
Features and validation of the Radio-Pathomic multimodal model. **(a)** The Radio-Pathomic multimodal model integrates 22 features of the total 1163 extracted features, including 12 radiomic features (the blue bars) and 10 pathomic features (the green bars). The greater length of green bars suggests pathomic features may contribute more substantially to the model’s predictions of chemosensitivity. **(b)** The model achieved an AUC of 0.814 in the validation cohort, exceeding the performance of the unimodal models. This indicates a potential complementary effect between radiomic and pathomic features.

Based on the 1163 features extracted from the CT images and pathological slide image above, and the 513 features that were significantly different in the pCR and non-PCR subgroups. As depicted in **Figure 4**, 22 features were selected in the binary LASSO regression to develop the Radio-Pathomic multimodal model, the ROC of the multimodal model tested in the validation cohort showed that the AUC is 0.814, higher than both the radiomic and pathomic single models mention above.

### Comparison of models

**Figure 5:**
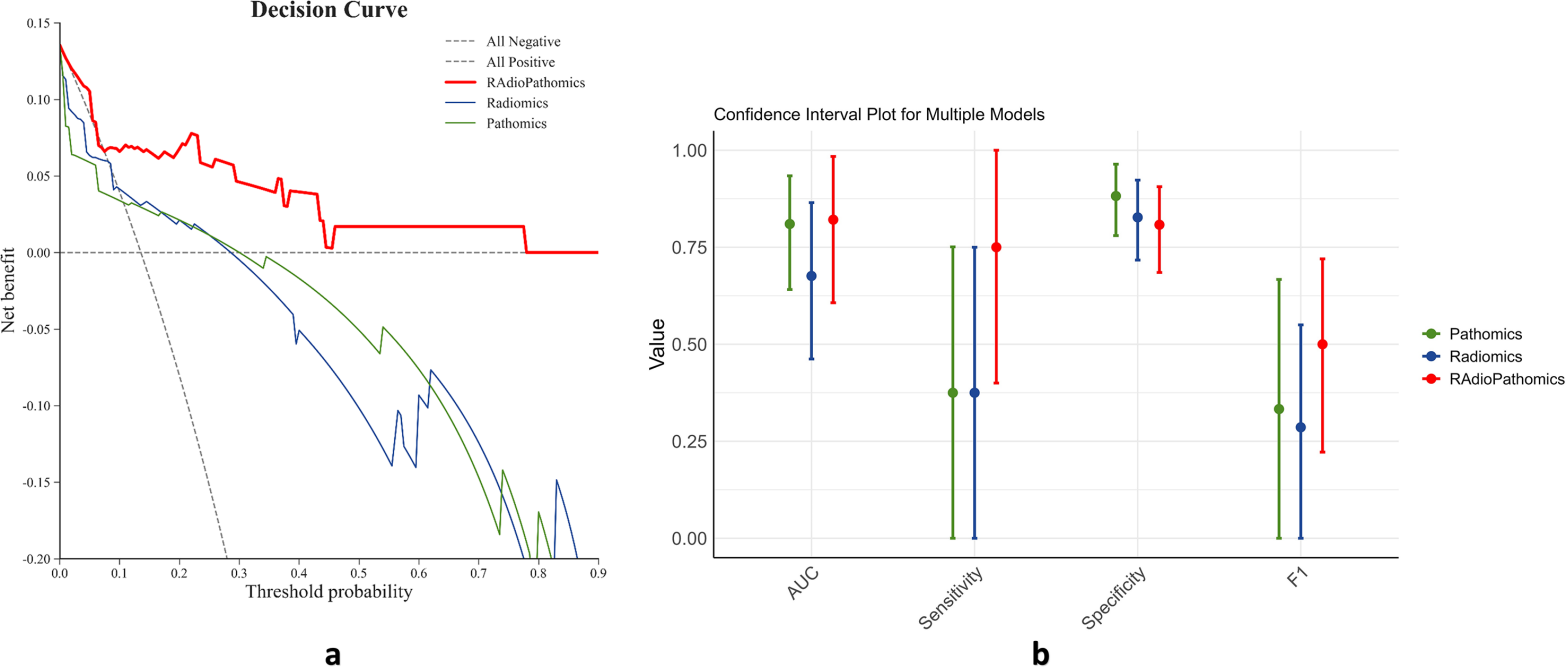
Comparison of performance between unimodal and the multimodal models. **(a)** In the decision curve analysis (DCA), where the Y-axis measures the net benefits, and the X-axis represents the threshold probability for “positive” (indicating higher potential of achieving pathological complete response after NAC), the multimodal model demonstrates superiority and more net benefit gains than the other two unimodal models. **(b)** The multimodal model also outperforms the unimodal models in terms of sensitivity and as measured by AUC and F1 score, metrics used to comprehensively evaluate the performance by combining precision and recall.

**Figure 5** presents the results of our decision curve analysis, comparing the predictive power of unimodal models with the pathomic multimodal model. While the multimodal model shows slightly lower specificity, it outperforms unimodal models in overall metrics such as AUC in the ROC test and F1-score. This improvement is particularly notable in terms of sensitivity. These results suggest that radiomic and pathomic features have a complementary effect, leading to a significantly improved prediction model for pathological complete responses to NAC.

## Discussion

In this study, we developed and validated a multimodal machine learning model for predicting pathological complete response (pCR) to neoadjuvant chemotherapy (NAC) in patients with advanced gastric cancer (AGC). Our model integrates multimodal features from both pre-treatment CT and pathological slide images, demonstrating satisfactory effectiveness in stratifying patients according to their sensitivity to NAC. This model holds the potential to serve as a powerful tool in personalizing treatment strategies for AGC patients.

Gastric cancer is a major health threat, particularly in Asia and less developed regions where over 70% of cases are diagnosed at advanced stages (1, 2). While radical resection of the primary tumor and perigastric lymph nodes remains the only potentially curative approach, the optimal comprehensive treatment strategy remains a subject of debate (25). A key controversy centers on the use of neoadjuvant chemotherapy (NAC). Currently, most scholars agree that the decision to employ NAC hinges on a patient’s sensitivity to chemotherapy (11, 26). For patients who are sensitive to NAC, the tumor will downstage or shrink, improving treatment outcomes such as R0 resection rates and survival. However, for those who are insensitive, postponing surgery may lead to tumor progression, potentially eliminating the possibility of surgical cure. Therefore, determining a patient’s chemotherapy sensitivity prior to selecting a treatment strategy is crucial.

Pathological complete response (pCR), defined as the absence of tumor cells in the primary tumor and surrounding lymph nodes, is a significant indicator of chemotherapy sensitivity. Studies have demonstrated that achieving pCR correlates with both short-term and long-term survival benefits from NAC (27–29). Unfortunately, pCR can only be definitively confirmed by postsurgical examination of the resected specimen. Thus, reliable biological markers for predicting pCR in advance of treatment are currently lacking.

Research efforts have focused on identifying biomarkers for predicting pCR. The most common approach is creating prediction models using pre-treatment information, including clinical, radiological, pathological, and molecular data. Regarding clinical data, Chen et al. identified tumor differentiation, peripheral lymphocyte ratio, and monocyte count as factors associated with pCR (30), Other studies also suggest a link between tumor differentiation and pathological response (31, 32). However, the assessment of tumor differentiation can be influenced by the pathologist’s experience, potentially introducing bias. Regarding radiological data, Chen et al. observed that better tumor differentiation might correlate with increased chemotherapy sensitivity. By combining machine learning with high-quality radiomic feature extraction, they established a model capable of predicting major complete response, achieving a C-index of 0.763 (12). Cui further demonstrated that integrating deep learning technology could enhance prediction accuracy (33). Regarding pathological data, Zhou et al. found the potential of developing prediction models using digital histopathological images of gastroscopic biopsy tissue (34). Similar research on breast tumors also indicates the feasibility of developing prediction models for pathological complete response after NAC using digital features from pathological slide images (35).

However, these existing models rely on unimodal information sources (such as CT or pathological slide images), this approach may limit their efficacy and robustness. Our hypothesis is that CT features represent the tumor’s macro-level characteristics, while pathological slide image features reflect micro-level details. Combining these features could enable the development of a prediction model that incorporates both macro and micro-level tumor bioinformation, potentially surpassing the capabilities of unimodal models. Previous studies, such as those focused on rectal cancer patients undergoing neoadjuvant therapy, have demonstrated the feasibility of establishing such multimodal models for pCR prediction. Our strategy involves the following steps: Feature Digitization: Digitize features from both CT and biopsy pathological images. Feature Selection: Identify features statistically associated with treatment outcomes. Feature Reduction: Employ Lasso regression to reduce feature dimensionality. Model Development: Explore various machine learning methods to establish the most suitable prediction model. As demonstrated in the results, the multimodal model outperformed unimodal models based solely on radiomic or pathomic features.

Interestingly, the unimodal pathomic model showed good performance, and adding radiomic features resulted in only marginal improvement. Pathomic features largely represent nuclear morphology, shape, and staining intensity (e.g., Zernike moments, area shape, hematoxylin intensity) (36), reflecting cellular atypia and proliferative activity that are closely linked to chemosensitivity. This may explain why the pathomic unimodal model performed well. However, biopsy specimens capture only a limited portion of the tumor and do not fully represent its heterogeneity. This limitation can be addressed by radiomic features, which provide a more comprehensive view of the tumor. The key radiomic features identified in our model mainly consisted of texture and wavelet-derived descriptors (e.g., GLRLM, GLSZM) (37), capturing intratumoral heterogeneity and density distribution. Although the improvement was modest, these features complemented the pathomic features and enhanced overall performance. Therefore, combining macro- and micro-level information remains a promising strategy.

To our knowledge, this is the first machine learning model for gastric cancer that integrates both pathomic and radiomic information. This multimodal approach has the potential to significantly improve personalized treatment strategies. While guidelines like NCCN (38), the Japanese gastric cancer treatment guideline (39), and the CSCO guideline (40) offer recommendations for NAC use, their variations can cause confusion in clinical practice. Our model, developed following TRIPOD, SPIRIT-AI, and CONSORT-AI guidelines for medical machine learning models (41–43), aims to address this by providing a tool that predicts pathological complete response (pCR) after NAC based on pre-treatment information. High predicted pCR would indicate a strong recommendation for NAC, while low pCR would suggest direct surgery. Furthermore, our model requires only readily obtainable data – biopsy pathology slides and pre-treatment CT scans – making it a highly practical tool for clinical application.

We acknowledge several limitations. First, genomic data such as microsatellite stability status, which may influence chemosensitivity, were not included, and we also failed to identify any clinical features (e.g., age, TNM stage, histologic grade) that were significantly associated with chemotherapy sensitivity to be incorporated into the model. Second, an independent prospective cohort is required to validate the generalizability of our model. Third, as with many high-capacity machine learning algorithms, our models showed near-perfect fit on the training set, indicating potential overfitting; therefore, we did not include training-set validation results in the main text, as they provide little meaningful information compared with the independent validation cohort. Nevertheless, by randomly dividing the cohort into training and validation sets at a reasonable ratio, we sought to minimize bias and enhance the reliability of our findings. Lastly, while pCR is an important indicator of chemosensitivity, many patients without pCR may still benefit from NAC through partial tumor downstaging, which warrants further investigation.

## Conclusion

In conclusion, we developed a novel multimodal model for predicting pCR to NAC in AGC by integrating radiomic and pathomic features. Our findings demonstrate the superior predictive performance of this multimodal approach compared to unimodal models, highlighting the value of combining diverse multimodal data. This model holds the potential for developing individualized treatment strategies for AGC patients.

## Data Availability

The raw data supporting the conclusions of this article will be made available by the authors, without undue reservation.
