# Supplementary material for: Integrating computed tomography and biopsy images to predict chemotherapy response in gastric cancer
Source: Front Oncol. 2025 Oct 21;15:1666358. doi: 10.3389/fonc.2025.1666358 (PMC12583031; doi:10.3389/fonc.2025.1666358)
Supplement: Supplementary file 1 [file DataSheet1.pdf]

Pathomic features of the validation cohort

	Radiomic Feature names	All cases in the Validation Cohort (n=59)	Non pathological complete response cases (n=51)	Pathological complete response cases (n=8)	p-value
1	Count_IdentifyPrimaryObjects_cell20*	2372.64(986.39)	2236.06(833.85)	3243.38(1446.65)	0.006
2	ExecutionTime_03NamesAndTypes_cell20	0.81(1.18)	0.78(1.15)	0.94(1.43)	0.346
3	ExecutionTime_05UnmixColors_cell20	0.56(0.17)	0.57(0.18)	0.54(0.08)	0.673
4	ExecutionTime_06IdentifyPrimaryObjects_cell20	3.38(1.21)	3.23(0.98)	4.36(2.03)	0.162
5	ExecutionTime_07MeasureObjectIntensity_cell20	0.86(0.46)	0.80(0.33)	1.27(0.88)	0.176
6	ExecutionTime_08MeasureObjectSizeShape_cell20*	15.45(5.54)	14.72(4.70)	20.13(8.23)	0.009
7	ExecutionTime_09MeasureTexture_cell20	67.41(24.62)	65.33(24.76)	80.70(20.32)	0.101
8	Group_Index_cell20	143.34(88.66)	141.14(85.23)	157.38(113.91)	0.634
9	Height_HE_cell20	1152.49(138.07)	1150.73(139.67)	1163.75(135.75)	0.825
10	ImageNumber_cell20	143.34(88.66)	141.14(85.23)	157.38(113.91)	0.634
11	Mean_IdentifyPrimaryObjects_AreaShape_Area_cell20	225.62(48.16)	222.72(48.49)	244.13(44.39)	0.246
12	Mean_IdentifyPrimaryObjects_AreaShape_BoundingBoxArea_cell20	424.56(107.26)	418.21(104.71)	465.04(121.85)	0.254
13	Mean_IdentifyPrimaryObjects_AreaShape_BoundingBoxMaximum_X_cell20	619.52(85.02)	616.89(85.72)	636.32(83.82)	0.26
14	Mean_IdentifyPrimaryObjects_AreaShape_BoundingBoxMaximum_Y_cell20	584.39(72.54)	583.60(73.27)	589.45(72.30)	0.64
15	Mean_IdentifyPrimaryObjects_AreaShape_BoundingBoxMinimum_X_cell20	598.92(85.49)	596.50(86.24)	614.36(84.35)	0.241
16	Mean_IdentifyPrimaryObjects_AreaShape_BoundingBoxMinimum_Y_cell20	565.31(72.62)	564.56(73.39)	570.11(72.04)	0.672
17	Mean_IdentifyPrimaryObjects_AreaShape_Center_X_cell20	608.72(85.24)	606.19(85.98)	624.84(84.06)	0.26
18	Mean_IdentifyPrimaryObjects_AreaShape_Center_Y_cell20	574.35(72.57)	573.57(73.32)	579.30(72.15)	0.64
19	Mean_IdentifyPrimaryObjects_AreaShape_CentralMoment_0_0_cell20	225.62(48.16)	222.72(48.49)	244.13(44.39)	0.246

20	Mean_IdentifyPrimaryObjects_AreaShape_CentralMoment_0_1_cell20	-0.00(0.00)	-0.00(0.00)	-0.00(0.00)	0.604
21	Mean_IdentifyPrimaryObjects_AreaShape_CentralMoment_0_2_cell20	9074.02(5133.49)	8590.10(4707.48)	12159.00(6890.60)	0.067
22	Mean_IdentifyPrimaryObjects_AreaShape_CentralMoment_0_3_cell20	-308.88(1758.12)	-196.96(1778.53)	-1022.43(1530.94)	0.22
23	Mean_IdentifyPrimaryObjects_AreaShape_CentralMoment_1_0_cell20	-0.00(0.00)	-0.00(0.00)	-0.00(0.00)	0.6
24	Mean_IdentifyPrimaryObjects_AreaShape_CentralMoment_1_1_cell20	-385.57(614.50)	-387.17(595.46)	-375.37(772.01)	0.96
25	Mean_IdentifyPrimaryObjects_AreaShape_CentralMoment_1_2_cell20	-187.42(907.42)	-173.39(918.07)	-276.92(889.67)	0.767
26	Mean_IdentifyPrimaryObjects_AreaShape_CentralMoment_1_3_cell20	-57589.26(109539.11)	-55753.78(102156.62)	-69290.45(157146.63)	0.957
27	Mean_IdentifyPrimaryObjects_AreaShape_CentralMoment_2_0_cell20	7633.23(3839.28)	7445.32(3819.01)	8831.17(4007.16)	0.347
28	Mean_IdentifyPrimaryObjects_AreaShape_CentralMoment_2_1_cell20*	99.44(900.37)	-53.36(793.57)	1073.55(981.89)	<0.001
29	Mean_IdentifyPrimaryObjects_AreaShape_CentralMoment_2_2_cell20	394770.36(335539.59)	362708.57(303037.87)	599164.27(470834.63)	0.206
30	Mean_IdentifyPrimaryObjects_AreaShape_CentralMoment_2_3_cell20	-35011.36(386560.14)	-67443.52(374376.25)	171743.67(424829.86)	0.06
31	Mean_IdentifyPrimaryObjects_AreaShape_Compactness_cell20	1.84(0.36)	1.83(0.36)	1.85(0.33)	0.898
32	Mean_IdentifyPrimaryObjects_AreaShape_ConvexArea_cell20	286.97(68.82)	282.81(67.64)	313.50(75.01)	0.244
33	Mean_IdentifyPrimaryObjects_AreaShape_Eccentricity_cell20	0.76(0.02)	0.76(0.02)	0.77(0.03)	0.7
34	Mean_IdentifyPrimaryObjects_AreaShape_EquivalentDiameter_cell20	16.26(1.59)	16.17(1.61)	16.86(1.41)	0.254
35	Mean_IdentifyPrimaryObjects_AreaShape_EulerNumber_cell20	1.00(0.00)	1.00(0.00)	1.00(0.00)	0.098
36	Mean_IdentifyPrimaryObjects_AreaShape_Extent_cell20	0.57(0.04)	0.57(0.05)	0.57(0.04)	0.972

37	Mean_IdentifyPrimaryObjects_AreaShape_FormFactor_cell20	0.62(0.09)	0.62(0.09)	0.61(0.09)	0.746
38	Mean_IdentifyPrimaryObjects_AreaShape_HuMoment_0_cell20	0.23(0.02)	0.23(0.02)	0.23(0.02)	0.892
39	Mean_IdentifyPrimaryObjects_AreaShape_HuMoment_1_cell20	0.02(0.01)	0.02(0.01)	0.02(0.01)	0.82
40	Mean_IdentifyPrimaryObjects_AreaShape_HuMoment_2_cell20	0.00(0.00)	0.00(0.00)	0.00(0.00)	0.57
41	Mean_IdentifyPrimaryObjects_AreaShape_HuMoment_3_cell20	0.00(0.00)	0.00(0.00)	0.00(0.00)	0.82
42	Mean_IdentifyPrimaryObjects_AreaShape_HuMoment_4_cell20	0.00(0.00)	0.00(0.00)	0.00(0.00)	0.353
43	Mean_IdentifyPrimaryObjects_AreaShape_HuMoment_5_cell20	0.00(0.00)	0.00(0.00)	0.00(0.00)	0.656
44	Mean_IdentifyPrimaryObjects_AreaShape_HuMoment_6_cell20	0.00(0.00)	0.00(0.00)	-0.00(0.00)	0.609
45	Mean_IdentifyPrimaryObjects_AreaShape_InertiaTensorEigenvalues_0_cell20	38.98(10.61)	38.19(9.88)	44.02(14.19)	0.15
46	Mean_IdentifyPrimaryObjects_AreaShape_InertiaTensorEigenvalues_1_cell20	12.63(2.66)	12.50(2.70)	13.46(2.40)	0.345
47	Mean_IdentifyPrimaryObjects_AreaShape_InertiaTensor_0_0_cell20	27.83(8.18)	27.04(7.41)	32.86(11.35)	0.061
48	Mean_IdentifyPrimaryObjects_AreaShape_InertiaTensor_0_1_cell20	1.15(1.60)	1.18(1.56)	1.01(1.93)	0.788
49	Mean_IdentifyPrimaryObjects_AreaShape_InertiaTensor_1_0_cell20	1.15(1.60)	1.18(1.56)	1.01(1.93)	0.788
50	Mean_IdentifyPrimaryObjects_AreaShape_InertiaTensor_1_1_cell20	23.78(5.95)	23.65(6.00)	24.62(5.98)	0.672
51	Mean_IdentifyPrimaryObjects_AreaShape_MajorAxisLength_cell20	23.41(2.91)	23.23(2.78)	24.58(3.59)	0.223
52	Mean_IdentifyPrimaryObjects_AreaShape_MaxFeretDiameter_cell20	23.54(3.04)	23.37(2.96)	24.62(3.55)	0.287
53	Mean_IdentifyPrimaryObjects_AreaShape_MaximumRadius_cell20	5.68(0.51)	5.66(0.54)	5.78(0.26)	0.542

54	Mean_IdentifyPrimaryObjects_AreaShape_MeanRadius_cell20	2.37(0.18)	2.36(0.20)	2.40(0.09)	0.555
55	Mean_IdentifyPrimaryObjects_AreaShape_MedianRadius_cell20	2.11(0.17)	2.11(0.18)	2.16(0.06)	0.133
56	Mean_IdentifyPrimaryObjects_AreaShape_MinFeretDiameter_cell20	13.68(1.57)	13.60(1.59)	14.18(1.45)	0.335
57	Mean_IdentifyPrimaryObjects_AreaShape_MinorAxisLength_cell20	13.46(1.30)	13.40(1.33)	13.84(1.10)	0.375
58	Mean_IdentifyPrimaryObjects_AreaShape_NormalizedMoment_0_2_cell20	0.12(0.02)	0.12(0.02)	0.13(0.02)	0.204
59	Mean_IdentifyPrimaryObjects_AreaShape_NormalizedMoment_0_3_cell20	-0.00(0.00)	0.00(0.00)	-0.00(0.00)	0.414
60	Mean_IdentifyPrimaryObjects_AreaShape_NormalizedMoment_1_1_cell20	-0.00(0.01)	-0.00(0.01)	-0.00(0.01)	0.675
61	Mean_IdentifyPrimaryObjects_AreaShape_NormalizedMoment_1_2_cell20	-0.00(0.00)	-0.00(0.00)	-0.00(0.00)	0.422
62	Mean_IdentifyPrimaryObjects_AreaShape_NormalizedMoment_1_3_cell20	-0.00(0.00)	-0.00(0.00)	-0.00(0.00)	0.377
63	Mean_IdentifyPrimaryObjects_AreaShape_NormalizedMoment_2_0_cell20	0.10(0.01)	0.11(0.01)	0.10(0.01)	0.205
64	Mean_IdentifyPrimaryObjects_AreaShape_NormalizedMoment_2_1_cell20	0.00(0.00)	0.00(0.00)	0.00(0.00)	0.455
65	Mean_IdentifyPrimaryObjects_AreaShape_NormalizedMoment_2_2_cell20	0.01(0.00)	0.01(0.00)	0.01(0.00)	0.782
66	Mean_IdentifyPrimaryObjects_AreaShape_NormalizedMoment_2_3_cell20	0.00(0.00)	0.00(0.00)	0.00(0.00)	0.769
67	Mean_IdentifyPrimaryObjects_AreaShape_NormalizedMoment_3_0_cell20	0.00(0.00)	0.00(0.00)	-0.00(0.00)	0.313
68	Mean_IdentifyPrimaryObjects_AreaShape_NormalizedMoment_3_1_cell20	-0.00(0.00)	-0.00(0.00)	-0.00(0.00)	0.465
69	Mean_IdentifyPrimaryObjects_AreaShape_NormalizedMoment_3_2_cell20	-0.00(0.00)	0.00(0.00)	-0.00(0.00)	0.145
70	Mean_IdentifyPrimaryObjects_AreaShape_NormalizedMoment_3_3_cell20	-0.00(0.00)	-0.00(0.00)	-0.00(0.00)	0.377

71	Mean_IdentifyPrimaryObjects_AreaShape_Orientation_cell20	-3.70(5.37)	-3.81(5.33)	-3.00(5.91)	0.694
72	Mean_IdentifyPrimaryObjects_AreaShape_Perimeter_cell20	69.59(11.03)	69.03(10.78)	73.10(12.71)	0.336
73	Mean_IdentifyPrimaryObjects_AreaShape_Solidity_cell20	0.82(0.05)	0.82(0.05)	0.82(0.04)	0.939
74	Mean_IdentifyPrimaryObjects_AreaShape_SpatialMoment_0_0_cell20	225.62(48.16)	222.72(48.49)	244.13(44.39)	0.246
75	Mean_IdentifyPrimaryObjects_AreaShape_SpatialMoment_0_1_cell20	2695.78(1031.24)	2617.44(999.53)	3195.18(1160.01)	0.142
76	Mean_IdentifyPrimaryObjects_AreaShape_SpatialMoment_0_2_cell20	47616.13(27224.43)	45183.19(25295.21)	63126.15(35393.90)	0.083
77	Mean_IdentifyPrimaryObjects_AreaShape_SpatialMoment_0_3_cell20*	1102422.13(866618.89)	1013941.69(764279.68)	1666484.97(1276651.15)	0.047
78	Mean_IdentifyPrimaryObjects_AreaShape_SpatialMoment_1_0_cell20	2468.60(886.09)	2422.83(885.97)	2760.35(886.70)	0.321
79	Mean_IdentifyPrimaryObjects_AreaShape_SpatialMoment_1_1_cell20	31864.58(16792.09)	30663.25(16231.09)	39523.07(19417.53)	0.167
80	Mean_IdentifyPrimaryObjects_AreaShape_SpatialMoment_1_2_cell20	602784.53(427651.44)	565353.54(397241.22)	841407.12(559382.76)	0.09
81	Mean_IdentifyPrimaryObjects_AreaShape_SpatialMoment_1_3_cell20	14792349.92(13489893.19)	13428975.60(11974893.48)	23483861.26(19587305.44)	0.156
82	Mean_IdentifyPrimaryObjects_AreaShape_SpatialMoment_2_0_cell20	39552.64(20452.56)	38547.35(20339.79)	45961.31(21368.71)	0.345
83	Mean_IdentifyPrimaryObjects_AreaShape_SpatialMoment_2_1_cell20	546755.77(369990.24)	520866.33(355885.16)	711800.94(439961.98)	0.177
84	Mean_IdentifyPrimaryObjects_AreaShape_SpatialMoment_2_2_cell20	10955645.49(9295833.62)	10145219.23(8599573.79)	16122112.89(12347685.89)	0.091
85	Mean_IdentifyPrimaryObjects_AreaShape_SpatialMoment_2_3_cell20	281979536.03(292643769.17)	252343195.76(259869613.20)	470911205.30(424293638.60)	0.177
86	Mean_IdentifyPrimaryObjects_AreaShape_Zernike_0_0_cell20	0.52(0.05)	0.52(0.05)	0.52(0.06)	0.843
87	Mean_IdentifyPrimaryObjects_AreaShape_Zernike_1_1_cell20	0.06(0.00)	0.06(0.00)	0.06(0.00)	0.299

88	Mean_IdentifyPrimaryObjects_AreaShape_Zernike_2_0_cell20	0.14(0.01)	0.14(0.01)	0.14(0.00)	0.933
89	Mean_IdentifyPrimaryObjects_AreaShape_Zernike_2_2_cell20	0.07(0.00)	0.07(0.00)	0.07(0.00)	0.785
90	Mean_IdentifyPrimaryObjects_AreaShape_Zernike_3_1_cell20	0.03(0.00)	0.03(0.00)	0.03(0.00)	0.79
91	Mean_IdentifyPrimaryObjects_AreaShape_Zernike_3_3_cell20	0.02(0.00)	0.02(0.00)	0.02(0.00)	0.497
92	Mean_IdentifyPrimaryObjects_AreaShape_Zernike_4_0_cell20	0.02(0.00)	0.02(0.00)	0.02(0.00)	0.332
93	Mean_IdentifyPrimaryObjects_AreaShape_Zernike_4_2_cell20	0.03(0.00)	0.03(0.00)	0.03(0.00)	0.258
94	Mean_IdentifyPrimaryObjects_AreaShape_Zernike_4_4_cell20	0.03(0.00)	0.03(0.00)	0.03(0.00)	0.303
95	Mean_IdentifyPrimaryObjects_AreaShape_Zernike_5_1_cell20	0.02(0.00)	0.02(0.00)	0.02(0.00)	1
96	Mean_IdentifyPrimaryObjects_AreaShape_Zernike_5_3_cell20	0.02(0.00)	0.02(0.00)	0.02(0.00)	0.563
97	Mean_IdentifyPrimaryObjects_AreaShape_Zernike_5_5_cell20	0.01(0.00)	0.01(0.00)	0.01(0.00)	0.367
98	Mean_IdentifyPrimaryObjects_AreaShape_Zernike_6_0_cell20	0.01(0.00)	0.01(0.00)	0.01(0.00)	0.842
99	Mean_IdentifyPrimaryObjects_AreaShape_Zernike_6_2_cell20	0.01(0.00)	0.01(0.00)	0.01(0.00)	0.92
100	Mean_IdentifyPrimaryObjects_AreaShape_Zernike_6_4_cell20	0.01(0.00)	0.01(0.00)	0.01(0.00)	0.174
101	Mean_IdentifyPrimaryObjects_AreaShape_Zernike_6_6_cell20	0.02(0.00)	0.02(0.00)	0.02(0.00)	0.808
102	Mean_IdentifyPrimaryObjects_AreaShape_Zernike_7_1_cell20	0.01(0.00)	0.01(0.00)	0.01(0.00)	0.243
103	Mean_IdentifyPrimaryObjects_AreaShape_Zernike_7_3_cell20	0.01(0.00)	0.01(0.00)	0.01(0.00)	0.995
104	Mean_IdentifyPrimaryObjects_AreaShape_Zernike_7_5_cell20	0.01(0.00)	0.01(0.00)	0.01(0.00)	0.442

105	Mean_IdentifyPrimaryObjects_AreaShape_Zernike_7_7_cell20	0.01(0.00)	0.01(0.00)	0.01(0.00)	0.901
106	Mean_IdentifyPrimaryObjects_AreaShape_Zernike_8_0_cell20	0.01(0.00)	0.01(0.00)	0.01(0.00)	0.751
107	Mean_IdentifyPrimaryObjects_AreaShape_Zernike_8_2_cell20	0.01(0.00)	0.01(0.00)	0.01(0.00)	0.325
108	Mean_IdentifyPrimaryObjects_AreaShape_Zernike_8_4_cell20	0.01(0.00)	0.01(0.00)	0.01(0.00)	0.683
109	Mean_IdentifyPrimaryObjects_AreaShape_Zernike_8_6_cell20	0.01(0.00)	0.01(0.00)	0.01(0.00)	0.47
110	Mean_IdentifyPrimaryObjects_AreaShape_Zernike_8_8_cell20	0.01(0.00)	0.01(0.00)	0.01(0.00)	0.562
111	Mean_IdentifyPrimaryObjects_AreaShape_Zernike_9_1_cell20	0.01(0.00)	0.01(0.00)	0.01(0.00)	0.136
112	Mean_IdentifyPrimaryObjects_AreaShape_Zernike_9_3_cell20	0.01(0.00)	0.01(0.00)	0.01(0.00)	0.429
113	Mean_IdentifyPrimaryObjects_AreaShape_Zernike_9_5_cell20	0.01(0.00)	0.01(0.00)	0.01(0.00)	0.658
114	Mean_IdentifyPrimaryObjects_AreaShape_Zernike_9_7_cell20	0.01(0.00)	0.01(0.00)	0.01(0.00)	0.957
115	Mean_IdentifyPrimaryObjects_AreaShape_Zernike_9_9_cell20	0.01(0.00)	0.01(0.00)	0.01(0.00)	0.699
116	Mean_IdentifyPrimaryObjects_Intensity_IntegratedIntensityEdge_Hematoxylin_cell20	24.09(7.84)	24.72(7.06)	20.04(11.50)	0.297
117	Mean_IdentifyPrimaryObjects_Intensity_IntegratedIntensity_Hematoxylin_cell20	116.51(37.33)	118.43(36.02)	104.23(45.63)	0.321
118	Mean_IdentifyPrimaryObjects_Intensity_LowerQuartileIntensity_Hematoxylin_cell20	0.43(0.15)	0.44(0.13)	0.35(0.23)	0.493
119	Mean_IdentifyPrimaryObjects_Intensity_MADIntensity_Hematoxylin_cell20	0.10(0.02)	0.10(0.03)	0.10(0.01)	0.895
120	Mean_IdentifyPrimaryObjects_Intensity_MassDisplacement_Hematoxylin_cell20	0.38(0.20)	0.37(0.19)	0.49(0.28)	0.199
121	Mean_IdentifyPrimaryObjects_Intensity_MaxIntensityEdge_Hematoxylin_cell20	0.59(0.13)	0.60(0.11)	0.53(0.20)	0.14

122	Mean_IdentifyPrimaryObjects_Intensity_MaxIntensity_Hematoxylin_cell20	0.79(0.11)	0.79(0.10)	0.73(0.16)	0.16
123	Mean_IdentifyPrimaryObjects_Intensity_MeanIntensityEdge_Hematoxylin_cell20	0.41(0.14)	0.42(0.12)	0.34(0.21)	0.507
124	Mean_IdentifyPrimaryObjects_Intensity_MeanIntensity_Hematoxylin_cell20	0.53(0.14)	0.54(0.13)	0.46(0.23)	0.64
125	Mean_IdentifyPrimaryObjects_Intensity_MedianIntensity_Hematoxylin_cell20	0.53(0.15)	0.54(0.13)	0.46(0.24)	0.64
126	Mean_IdentifyPrimaryObjects_Intensity_MinIntensityEdge_Hematoxylin_cell20	0.25(0.14)	0.26(0.14)	0.21(0.18)	0.363
127	Mean_IdentifyPrimaryObjects_Intensity_MinIntensity_Hematoxylin_cell20	0.24(0.15)	0.25(0.14)	0.21(0.17)	0.435
128	Mean_IdentifyPrimaryObjects_Intensity_StdIntensityEdge_Hematoxylin_cell20	0.08(0.02)	0.08(0.02)	0.08(0.01)	0.721
129	Mean_IdentifyPrimaryObjects_Intensity_StdIntensity_Hematoxylin_cell20	0.13(0.03)	0.13(0.04)	0.13(0.02)	0.893
130	Mean_IdentifyPrimaryObjects_Intensity_UpperQuartileIntensity_Hematoxylin_cell20	0.63(0.15)	0.64(0.13)	0.56(0.24)	0.342
131	Mean_IdentifyPrimaryObjects_Location_CenterMassIntensity_X_Hematoxylin_cell20	608.71(85.24)	606.18(85.97)	624.84(84.05)	0.26
132	Mean_IdentifyPrimaryObjects_Location_CenterMassIntensity_Y_Hematoxylin_cell20	574.35(72.57)	573.58(73.32)	579.30(72.13)	0.656
133	Mean_IdentifyPrimaryObjects_Location_Center_X_cell20	608.72(85.24)	606.19(85.98)	624.84(84.06)	0.26
134	Mean_IdentifyPrimaryObjects_Location_Center_Y_cell20	574.35(72.57)	573.57(73.32)	579.30(72.15)	0.64
135	Mean_IdentifyPrimaryObjects_Location_MaxIntensity_X_Hematoxylin_cell20	608.87(85.26)	606.35(85.99)	624.93(84.09)	0.26
136	Mean_IdentifyPrimaryObjects_Location_MaxIntensity_Y_Hematoxylin_cell20	574.21(72.57)	573.43(73.31)	579.23(72.20)	0.625
137	Mean_IdentifyPrimaryObjects_Texture_AngularSecondMoment_Hematoxylin_3_00_256_cell20	0.01(0.00)	0.01(0.00)	0.01(0.00)	0.838
138	Mean_IdentifyPrimaryObjects_Texture_AngularSecondMoment_Hematoxylin_3_01_256_cell20	0.01(0.00)	0.01(0.00)	0.01(0.00)	0.873

139	Mean_IdentifyPrimaryObjects_Texture_AngularSecondMoment_Hematoxylin_3_02_256_cell20	0.01(0.00)	0.01(0.00)	0.01(0.00)	0.868
140	Mean_IdentifyPrimaryObjects_Texture_AngularSecondMoment_Hematoxylin_3_03_256_cell20	0.01(0.00)	0.01(0.00)	0.01(0.00)	0.815
141	Mean_IdentifyPrimaryObjects_Texture_Contrast_Hematoxylin_3_00_256_cell20	1884.45(1077.20)	1910.73(1143.32)	1716.89(490.35)	0.64
142	Mean_IdentifyPrimaryObjects_Texture_Contrast_Hematoxylin_3_01_256_cell20	2359.95(1236.39)	2385.72(1312.11)	2195.67(576.94)	0.69
143	Mean_IdentifyPrimaryObjects_Texture_Contrast_Hematoxylin_3_02_256_cell20	1957.52(1088.94)	1967.45(1153.05)	1894.19(568.46)	0.861
144	Mean_IdentifyPrimaryObjects_Texture_Contrast_Hematoxylin_3_03_256_cell20	2331.14(1234.01)	2357.11(1306.94)	2165.57(616.79)	0.854
145	Mean_IdentifyPrimaryObjects_Texture_Correlation_Hematoxylin_3_00_256_cell20	0.20(0.10)	0.20(0.10)	0.18(0.13)	0.617
146	Mean_IdentifyPrimaryObjects_Texture_Correlation_Hematoxylin_3_01_256_cell20	0.02(0.06)	0.02(0.06)	-0.00(0.06)	0.335
147	Mean_IdentifyPrimaryObjects_Texture_Correlation_Hematoxylin_3_02_256_cell20	0.16(0.10)	0.17(0.09)	0.11(0.13)	0.091
148	Mean_IdentifyPrimaryObjects_Texture_Correlation_Hematoxylin_3_03_256_cell20	0.03(0.07)	0.03(0.07)	0.01(0.07)	0.501
149	Mean_IdentifyPrimaryObjects_Texture_DifferenceEntropy_Hematoxylin_3_00_256_cell20	5.49(0.22)	5.49(0.23)	5.51(0.11)	0.786
150	Mean_IdentifyPrimaryObjects_Texture_DifferenceEntropy_Hematoxylin_3_01_256_cell20	5.46(0.20)	5.46(0.21)	5.47(0.14)	0.879
151	Mean_IdentifyPrimaryObjects_Texture_DifferenceEntropy_Hematoxylin_3_02_256_cell20	5.49(0.21)	5.49(0.23)	5.52(0.12)	0.696
152	Mean_IdentifyPrimaryObjects_Texture_DifferenceEntropy_Hematoxylin_3_03_256_cell20	5.47(0.19)	5.47(0.20)	5.48(0.14)	0.912
153	Mean_IdentifyPrimaryObjects_Texture_DifferenceVariance_Hematoxylin_3_00_256_cell20	0.00(0.00)	0.00(0.00)	0.00(0.00)	0.769
154	Mean_IdentifyPrimaryObjects_Texture_DifferenceVariance_Hematoxylin_3_01_256_cell20	0.00(0.00)	0.00(0.00)	0.00(0.00)	1
155	Mean_IdentifyPrimaryObjects_Texture_DifferenceVariance_Hematoxylin_3_02_256_cell20	0.00(0.00)	0.00(0.00)	0.00(0.00)	0.905

156	Mean_IdentifyPrimaryObjects_Texture_DifferenceVariance_Hematoxylin_3_03_256_cell20	0.00(0.00)	0.00(0.00)	0.00(0.00)	0.974
157	Mean_IdentifyPrimaryObjects_Texture_Entropy_Hematoxylin_3_00_256_cell20	7.91(0.28)	7.90(0.30)	7.94(0.21)	0.702
158	Mean_IdentifyPrimaryObjects_Texture_Entropy_Hematoxylin_3_01_256_cell20	7.61(0.31)	7.61(0.33)	7.63(0.21)	0.832
159	Mean_IdentifyPrimaryObjects_Texture_Entropy_Hematoxylin_3_02_256_cell20	7.86(0.28)	7.86(0.30)	7.87(0.21)	0.915
160	Mean_IdentifyPrimaryObjects_Texture_Entropy_Hematoxylin_3_03_256_cell20	7.65(0.31)	7.65(0.33)	7.67(0.24)	0.869
161	Mean_IdentifyPrimaryObjects_Texture_InfoMeas1_Hematoxylin_3_00_256_cell20	-0.68(0.05)	-0.68(0.05)	-0.68(0.02)	0.88
162	Mean_IdentifyPrimaryObjects_Texture_InfoMeas1_Hematoxylin_3_01_256_cell20	-0.72(0.05)	-0.72(0.05)	-0.72(0.03)	0.952
163	Mean_IdentifyPrimaryObjects_Texture_InfoMeas1_Hematoxylin_3_02_256_cell20	-0.69(0.05)	-0.69(0.05)	-0.69(0.03)	0.911
164	Mean_IdentifyPrimaryObjects_Texture_InfoMeas1_Hematoxylin_3_03_256_cell20	-0.72(0.05)	-0.72(0.06)	-0.72(0.03)	0.991
165	Mean_IdentifyPrimaryObjects_Texture_InfoMeas2_Hematoxylin_3_00_256_cell20	1.00(0.00)	1.00(0.00)	1.00(0.00)	0.224
166	Mean_IdentifyPrimaryObjects_Texture_InfoMeas2_Hematoxylin_3_01_256_cell20	1.00(0.00)	1.00(0.00)	1.00(0.00)	0.207
167	Mean_IdentifyPrimaryObjects_Texture_InfoMeas2_Hematoxylin_3_02_256_cell20	1.00(0.00)	1.00(0.00)	1.00(0.00)	0.224
168	Mean_IdentifyPrimaryObjects_Texture_InfoMeas2_Hematoxylin_3_03_256_cell20	1.00(0.00)	1.00(0.00)	1.00(0.00)	0.177
169	Mean_IdentifyPrimaryObjects_Texture_InverseDifferenceMoment_Hematoxylin_3_00_256_cell20	0.04(0.01)	0.04(0.01)	0.04(0.01)	0.653
170	Mean_IdentifyPrimaryObjects_Texture_InverseDifferenceMoment_Hematoxylin_3_01_256_cell20	0.04(0.01)	0.04(0.01)	0.04(0.00)	0.692
171	Mean_IdentifyPrimaryObjects_Texture_InverseDifferenceMoment_Hematoxylin_3_02_256_cell20	0.04(0.01)	0.04(0.01)	0.04(0.01)	0.398

172	Mean_IdentifyPrimaryObjects_Texture_InverseDifferenceMoment_Hematoxylin_3_03_256_cell20	0.04(0.01)	0.04(0.01)	0.04(0.01)	0.68
173	Mean_IdentifyPrimaryObjects_Texture_SumAverage_Hematoxylin_3_00_256_cell20	283.32(69.85)	288.66(61.11)	249.28(110.55)	0.14
174	Mean_IdentifyPrimaryObjects_Texture_SumAverage_Hematoxylin_3_01_256_cell20	281.27(70.37)	286.66(61.50)	246.92(111.61)	0.656
175	Mean_IdentifyPrimaryObjects_Texture_SumAverage_Hematoxylin_3_02_256_cell20	282.93(70.09)	288.31(61.26)	248.58(111.11)	0.137
176	Mean_IdentifyPrimaryObjects_Texture_SumAverage_Hematoxylin_3_03_256_cell20	281.69(70.23)	287.06(61.41)	247.43(111.28)	0.656
177	Mean_IdentifyPrimaryObjects_Texture_SumEntropy_Hematoxylin_3_00_256_cell20	6.19(0.22)	6.19(0.22)	6.20(0.19)	0.919
178	Mean_IdentifyPrimaryObjects_Texture_SumEntropy_Hematoxylin_3_01_256_cell20	5.95(0.23)	5.95(0.24)	5.95(0.18)	0.989
179	Mean_IdentifyPrimaryObjects_Texture_SumEntropy_Hematoxylin_3_02_256_cell20	6.16(0.21)	6.16(0.22)	6.14(0.18)	0.781
180	Mean_IdentifyPrimaryObjects_Texture_SumEntropy_Hematoxylin_3_03_256_cell20	5.98(0.23)	5.98(0.24)	5.98(0.20)	0.958
181	Mean_IdentifyPrimaryObjects_Texture_SumVariance_Hematoxylin_3_00_256_cell20	2939.97(1479.08)	2972.97(1555.09)	2729.58(891.35)	0.669
182	Mean_IdentifyPrimaryObjects_Texture_SumVariance_Hematoxylin_3_01_256_cell20	2490.21(1250.32)	2524.27(1315.54)	2273.05(727.28)	0.602
183	Mean_IdentifyPrimaryObjects_Texture_SumVariance_Hematoxylin_3_02_256_cell20	2868.13(1460.61)	2916.64(1535.08)	2558.94(847.60)	0.524
184	Mean_IdentifyPrimaryObjects_Texture_SumVariance_Hematoxylin_3_03_256_cell20	2520.06(1260.41)	2551.95(1328.33)	2316.73(710.77)	0.628
185	Mean_IdentifyPrimaryObjects_Texture_Variance_Hematoxylin_3_00_256_cell20	1206.10(626.19)	1220.93(662.51)	1111.62(319.39)	0.65
186	Mean_IdentifyPrimaryObjects_Texture_Variance_Hematoxylin_3_01_256_cell20	1212.54(615.47)	1227.50(650.82)	1117.18(318.06)	0.641
187	Mean_IdentifyPrimaryObjects_Texture_Variance_Hematoxylin_3_02_256_cell20	1206.41(623.71)	1221.02(659.74)	1113.28(320.61)	0.654
188	Mean_IdentifyPrimaryObjects_Texture_Variance_Hematoxylin_3_03_256_cell20	1212.80(615.21)	1227.27(650.59)	1120.58(318.39)	0.652

189	Median_IdentifyPrimaryObjects_AreaShape_Area_cell20	189.92(37.66)	188.16(38.81)	201.19(28.71)	0.367
190	Median_IdentifyPrimaryObjects_AreaShape_BoundingBoxArea_cell20	341.34(77.34)	338.04(77.78)	362.38(75.86)	0.413
191	Median_IdentifyPrimaryObjects_AreaShape_BoundingBoxMaximum_X_cell20	620.81(93.87)	617.81(95.95)	639.94(82.22)	0.54
192	Median_IdentifyPrimaryObjects_AreaShape_BoundingBoxMaximum_Y_cell20	583.14(79.37)	582.07(80.14)	590.00(79.10)	0.795
193	Median_IdentifyPrimaryObjects_AreaShape_BoundingBoxMinimum_X_cell20	600.04(94.58)	597.25(96.47)	617.81(85.07)	0.572
194	Median_IdentifyPrimaryObjects_AreaShape_BoundingBoxMinimum_Y_cell20	564.14(79.22)	563.04(80.14)	571.19(77.82)	0.789
195	Median_IdentifyPrimaryObjects_AreaShape_Center_X_cell20	609.88(94.03)	606.93(95.98)	628.68(83.67)	0.548
196	Median_IdentifyPrimaryObjects_AreaShape_Center_Y_cell20	572.99(79.24)	571.92(80.11)	579.78(78.31)	0.797
197	Median_IdentifyPrimaryObjects_AreaShape_CentralMoment_0_0_cell20	189.92(37.66)	188.16(38.81)	201.19(28.71)	0.367
198	Median_IdentifyPrimaryObjects_AreaShape_CentralMoment_0_1_cell20	-0.00(0.00)	-0.00(0.00)	0.00(0.00)	0.839
199	Median_IdentifyPrimaryObjects_AreaShape_CentralMoment_0_2_cell20	4123.53(1834.26)	4016.42(1827.14)	4806.33(1847.96)	0.261
200	Median_IdentifyPrimaryObjects_AreaShape_CentralMoment_0_3_cell20	8.42(44.53)	9.58(45.32)	1.05(41.11)	0.619
201	Median_IdentifyPrimaryObjects_AreaShape_CentralMoment_1_0_cell20	0.00(0.00)	0.00(0.00)	0.00(0.00)	1
202	Median_IdentifyPrimaryObjects_AreaShape_CentralMoment_1_1_cell20	-92.04(133.89)	-93.25(133.33)	-84.32(146.49)	0.863
203	Median_IdentifyPrimaryObjects_AreaShape_CentralMoment_1_2_cell20	0.73(41.27)	-1.91(41.20)	17.54(40.19)	0.218
204	Median_IdentifyPrimaryObjects_AreaShape_CentralMoment_1_3_cell20	-2594.53(3945.26)	-2581.71(3915.06)	-2676.28(4413.05)	0.95
205	Median_IdentifyPrimaryObjects_AreaShape_CentralMoment_2_0_cell20	3532.99(1411.52)	3503.54(1462.30)	3720.75(1089.94)	0.689

206	Median_IdentifyPrimaryObjects_AreaShape_CentralMoment_2_1_cell20	6.56(32.24)	7.50(34.36)	0.63(11.39)	0.282
207	Median_IdentifyPrimaryObjects_AreaShape_CentralMoment_2_2_cell20	70078.88(44746.05)	68395.46(45372.97)	80810.71(41622.25)	0.47
208	Median_IdentifyPrimaryObjects_AreaShape_CentralMoment_2_3_cell20	187.82(566.28)	166.37(589.48)	324.59(387.52)	0.467
209	Median_IdentifyPrimaryObjects_AreaShape_Compactness_cell20	1.67(0.30)	1.67(0.30)	1.69(0.29)	0.856
210	Median_IdentifyPrimaryObjects_AreaShape_ConvexArea_cell20	234.71(51.05)	232.55(51.87)	248.50(46.12)	0.416
211	Median_IdentifyPrimaryObjects_AreaShape_Eccentricity_cell20	0.79(0.02)	0.79(0.02)	0.80(0.04)	0.73
212	Median_IdentifyPrimaryObjects_AreaShape_EquivalentDiameter_cell20	15.48(1.51)	15.40(1.55)	15.97(1.17)	0.329
213	Median_IdentifyPrimaryObjects_AreaShape_Extent_cell20	0.58(0.05)	0.58(0.05)	0.58(0.04)	0.936
214	Median_IdentifyPrimaryObjects_AreaShape_FormFactor_cell20	0.62(0.10)	0.62(0.11)	0.61(0.10)	0.798
215	Median_IdentifyPrimaryObjects_AreaShape_HuMoment_0_cell20	0.21(0.02)	0.21(0.02)	0.21(0.02)	0.753
216	Median_IdentifyPrimaryObjects_AreaShape_HuMoment_1_cell20	0.01(0.00)	0.01(0.00)	0.01(0.00)	0.492
217	Median_IdentifyPrimaryObjects_AreaShape_HuMoment_2_cell20	0.00(0.00)	0.00(0.00)	0.00(0.00)	0.915
218	Median_IdentifyPrimaryObjects_AreaShape_HuMoment_3_cell20	0.00(0.00)	0.00(0.00)	0.00(0.00)	0.922
219	Median_IdentifyPrimaryObjects_AreaShape_HuMoment_4_cell20	0.00(0.00)	0.00(0.00)	0.00(0.00)	0.991
220	Median_IdentifyPrimaryObjects_AreaShape_HuMoment_5_cell20	0.00(0.00)	0.00(0.00)	0.00(0.00)	0.991
221	Median_IdentifyPrimaryObjects_AreaShape_HuMoment_6_cell20	0.00(0.00)	-0.00(0.00)	0.00(0.00)	0.17
222	Median_IdentifyPrimaryObjects_AreaShape_InertiaTensorEigenvalues_0_cell20	30.47(7.03)	30.09(6.85)	32.90(8.19)	0.298

223	Median_IdentifyPrimaryObjects_AreaShape_InertiaTensorEigenvalues_1_cell20	10.12(1.92)	10.07(1.99)	10.45(1.42)	0.607
224	Median_IdentifyPrimaryObjects_AreaShape_InertiaTensor_0_0_cell20	20.83(5.21)	20.44(5.00)	23.34(6.16)	0.144
225	Median_IdentifyPrimaryObjects_AreaShape_InertiaTensor_0_1_cell20	0.64(0.93)	0.65(0.92)	0.59(1.00)	0.881
226	Median_IdentifyPrimaryObjects_AreaShape_InertiaTensor_1_0_cell20	0.64(0.93)	0.65(0.92)	0.59(1.00)	0.881
227	Median_IdentifyPrimaryObjects_AreaShape_InertiaTensor_1_1_cell20	17.92(3.77)	17.89(3.87)	18.09(3.26)	0.89
228	Median_IdentifyPrimaryObjects_AreaShape_MajorAxisLength_cell20	21.94(2.54)	21.80(2.49)	22.78(2.89)	0.315
229	Median_IdentifyPrimaryObjects_AreaShape_MaxFeretDiameter_cell20	22.03(2.72)	21.91(2.69)	22.80(2.95)	0.393
230	Median_IdentifyPrimaryObjects_AreaShape_MaximumRadius_cell20	5.43(0.50)	5.43(0.52)	5.47(0.36)	0.847
231	Median_IdentifyPrimaryObjects_AreaShape_MeanRadius_cell20	2.29(0.17)	2.29(0.18)	2.31(0.09)	0.792
232	Median_IdentifyPrimaryObjects_AreaShape_MedianRadius_cell20	2.03(0.12)	2.03(0.13)	2.03(0.08)	0.739
233	Median_IdentifyPrimaryObjects_AreaShape_MinFeretDiameter_cell20	12.79(1.43)	12.73(1.47)	13.12(1.21)	0.484
234	Median_IdentifyPrimaryObjects_AreaShape_MinorAxisLength_cell20	12.67(1.19)	12.63(1.23)	12.90(0.89)	0.556
235	Median_IdentifyPrimaryObjects_AreaShape_NormalizedMoment_0_2_cell20	0.11(0.01)	0.10(0.01)	0.11(0.02)	0.1
236	Median_IdentifyPrimaryObjects_AreaShape_NormalizedMoment_0_3_cell20	0.00(0.00)	0.00(0.00)	0.00(0.00)	0.762
237	Median_IdentifyPrimaryObjects_AreaShape_NormalizedMoment_1_1_cell20	-0.00(0.01)	-0.00(0.01)	-0.00(0.01)	0.818
238	Median_IdentifyPrimaryObjects_AreaShape_NormalizedMoment_1_2_cell20	0.00(0.00)	-0.00(0.00)	0.00(0.00)	0.211
239	Median_IdentifyPrimaryObjects_AreaShape_NormalizedMoment_1_3_cell20	-0.00(0.00)	-0.00(0.00)	-0.00(0.00)	0.933

240	Median_IdentifyPrimaryObjects_AreaShape_NormalizedMoment_2_0_cell20	0.09(0.01)	0.09(0.01)	0.09(0.01)	0.167
241	Median_IdentifyPrimaryObjects_AreaShape_NormalizedMoment_2_1_cell20	0.00(0.00)	0.00(0.00)	0.00(0.00)	0.128
242	Median_IdentifyPrimaryObjects_AreaShape_NormalizedMoment_2_2_cell20	0.01(0.00)	0.01(0.00)	0.01(0.00)	0.925
243	Median_IdentifyPrimaryObjects_AreaShape_NormalizedMoment_2_3_cell20	0.00(0.00)	0.00(0.00)	0.00(0.00)	0.405
244	Median_IdentifyPrimaryObjects_AreaShape_NormalizedMoment_3_0_cell20*	-0.00(0.00)	0.00(0.00)	-0.00(0.00)	0.023
245	Median_IdentifyPrimaryObjects_AreaShape_NormalizedMoment_3_1_cell20	-0.00(0.00)	-0.00(0.00)	-0.00(0.00)	0.808
246	Median_IdentifyPrimaryObjects_AreaShape_NormalizedMoment_3_2_cell20	-0.00(0.00)	-0.00(0.00)	-0.00(0.00)	0.97
247	Median_IdentifyPrimaryObjects_AreaShape_NormalizedMoment_3_3_cell20	-0.00(0.00)	-0.00(0.00)	-0.00(0.00)	0.874
248	Median_IdentifyPrimaryObjects_AreaShape_Orientation_cell20	-8.09(10.82)	-8.18(10.59)	-7.53(13.00)	0.875
249	Median_IdentifyPrimaryObjects_AreaShape_Perimeter_cell20	63.53(9.43)	63.11(9.39)	66.17(9.94)	0.398
250	Median_IdentifyPrimaryObjects_AreaShape_Solidity_cell20	0.83(0.05)	0.83(0.05)	0.83(0.05)	0.926
251	Median_IdentifyPrimaryObjects_AreaShape_SpatialMoment_0_0_cell20	189.92(37.66)	188.16(38.81)	201.19(28.71)	0.367
252	Median_IdentifyPrimaryObjects_AreaShape_SpatialMoment_0_1_cell20	1748.62(582.33)	1718.76(591.46)	1938.94(512.93)	0.324
253	Median_IdentifyPrimaryObjects_AreaShape_SpatialMoment_0_2_cell20	20647.03(9625.79)	20157.44(9664.95)	23768.19(9360.44)	0.328
254	Median_IdentifyPrimaryObjects_AreaShape_SpatialMoment_0_3_cell20	277841.42(167273.84)	268844.18(166103.24)	335198.81(174400.77)	0.301
255	Median_IdentifyPrimaryObjects_AreaShape_SpatialMoment_1_0_cell20	1611.94(495.28)	1595.74(511.61)	1715.25(385.77)	0.53
256	Median_IdentifyPrimaryObjects_AreaShape_SpatialMoment_1_1_cell20	14690.96(6454.19)	14438.29(6602.39)	16301.69(5509.61)	0.453

257	Median_IdentifyPrimaryObjects_AreaShape_SpatialMoment_1_2_cell20	171829.16(97983.57)	167868.61(99738.93)	197077.69(87466.47)	0.438
258	Median_IdentifyPrimaryObjects_AreaShape_SpatialMoment_1_3_cell20	2310446.91(1634732.65)	2240903.09(1650624.88)	2753788.75(1556055.21)	0.414
259	Median_IdentifyPrimaryObjects_AreaShape_SpatialMoment_2_0_cell20	17463.97(7277.91)	17293.17(7528.64)	18552.88(5696.39)	0.653
260	Median_IdentifyPrimaryObjects_AreaShape_SpatialMoment_2_1_cell20	158292.03(86351.13)	155787.53(89019.50)	174258.25(69585.69)	0.578
261	Median_IdentifyPrimaryObjects_AreaShape_SpatialMoment_2_2_cell20	1825420.38(1224906.07)	1784137.05(1254284.08)	2088601.62(1050199.99)	0.518
262	Median_IdentifyPrimaryObjects_AreaShape_SpatialMoment_2_3_cell20	24125378.97(19513173.90)	23470182.44(19935014.21)	28302256.81(17123943.06)	0.52
263	Median_IdentifyPrimaryObjects_AreaShape_Zernike_0_0_cell20	0.51(0.05)	0.52(0.05)	0.51(0.06)	0.829
264	Median_IdentifyPrimaryObjects_AreaShape_Zernike_1_1_cell20	0.06(0.00)	0.06(0.00)	0.06(0.01)	0.292
265	Median_IdentifyPrimaryObjects_AreaShape_Zernike_2_0_cell20	0.14(0.01)	0.14(0.01)	0.14(0.01)	0.943
266	Median_IdentifyPrimaryObjects_AreaShape_Zernike_2_2_cell20	0.07(0.01)	0.07(0.01)	0.07(0.00)	0.778
267	Median_IdentifyPrimaryObjects_AreaShape_Zernike_3_1_cell20	0.03(0.00)	0.03(0.00)	0.03(0.00)	0.833
268	Median_IdentifyPrimaryObjects_AreaShape_Zernike_3_3_cell20	0.02(0.00)	0.02(0.00)	0.02(0.00)	0.849
269	Median_IdentifyPrimaryObjects_AreaShape_Zernike_4_0_cell20	0.02(0.00)	0.02(0.00)	0.02(0.00)	0.444
270	Median_IdentifyPrimaryObjects_AreaShape_Zernike_4_2_cell20	0.03(0.00)	0.03(0.00)	0.03(0.00)	0.297
271	Median_IdentifyPrimaryObjects_AreaShape_Zernike_4_4_cell20	0.03(0.00)	0.03(0.00)	0.03(0.00)	0.401
272	Median_IdentifyPrimaryObjects_AreaShape_Zernike_5_1_cell20	0.01(0.00)	0.01(0.00)	0.01(0.00)	0.964
273	Median_IdentifyPrimaryObjects_AreaShape_Zernike_5_3_cell20	0.01(0.00)	0.01(0.00)	0.01(0.00)	0.667

274	Median_IdentifyPrimaryObjects_AreaShape_Zernike_5_5_cell20	0.01(0.00)	0.01(0.00)	0.01(0.00)	0.538
275	Median_IdentifyPrimaryObjects_AreaShape_Zernike_6_0_cell20	0.01(0.00)	0.01(0.00)	0.01(0.00)	0.846
276	Median_IdentifyPrimaryObjects_AreaShape_Zernike_6_2_cell20	0.01(0.00)	0.01(0.00)	0.01(0.00)	0.873
277	Median_IdentifyPrimaryObjects_AreaShape_Zernike_6_4_cell20	0.01(0.00)	0.01(0.00)	0.01(0.00)	0.124
278	Median_IdentifyPrimaryObjects_AreaShape_Zernike_6_6_cell20	0.02(0.00)	0.02(0.00)	0.02(0.00)	0.841
279	Median_IdentifyPrimaryObjects_AreaShape_Zernike_7_1_cell20	0.01(0.00)	0.01(0.00)	0.01(0.00)	0.282
280	Median_IdentifyPrimaryObjects_AreaShape_Zernike_7_3_cell20	0.01(0.00)	0.01(0.00)	0.01(0.00)	0.991
281	Median_IdentifyPrimaryObjects_AreaShape_Zernike_7_5_cell20	0.01(0.00)	0.01(0.00)	0.01(0.00)	0.292
282	Median_IdentifyPrimaryObjects_AreaShape_Zernike_7_7_cell20	0.01(0.00)	0.01(0.00)	0.01(0.00)	0.812
283	Median_IdentifyPrimaryObjects_AreaShape_Zernike_8_0_cell20	0.01(0.00)	0.01(0.00)	0.01(0.00)	0.826
284	Median_IdentifyPrimaryObjects_AreaShape_Zernike_8_2_cell20	0.01(0.00)	0.01(0.00)	0.01(0.00)	0.208
285	Median_IdentifyPrimaryObjects_AreaShape_Zernike_8_4_cell20	0.01(0.00)	0.01(0.00)	0.01(0.00)	0.976
286	Median_IdentifyPrimaryObjects_AreaShape_Zernike_8_6_cell20	0.01(0.00)	0.01(0.00)	0.01(0.00)	0.147
287	Median_IdentifyPrimaryObjects_AreaShape_Zernike_8_8_cell20	0.01(0.00)	0.01(0.00)	0.01(0.00)	0.581
288	Median_IdentifyPrimaryObjects_AreaShape_Zernike_9_1_cell20*	0.01(0.00)	0.01(0.00)	0.01(0.00)	0.031
289	Median_IdentifyPrimaryObjects_AreaShape_Zernike_9_3_cell20	0.01(0.00)	0.01(0.00)	0.01(0.00)	0.334
290	Median_IdentifyPrimaryObjects_AreaShape_Zernike_9_5_cell20	0.01(0.00)	0.01(0.00)	0.01(0.00)	0.67

291	Median_IdentifyPrimaryObjects_AreaShape_Zernike_9_7_cell20	0.01(0.00)	0.01(0.00)	0.01(0.00)	0.737
292	Median_IdentifyPrimaryObjects_AreaShape_Zernike_9_9_cell20	0.01(0.00)	0.01(0.00)	0.01(0.00)	0.858
293	Median_IdentifyPrimaryObjects_Intensity_IntegratedIntensityEdge_Hematoxylin_cell20	22.08(7.37)	22.67(6.61)	18.34(10.92)	0.308
294	Median_IdentifyPrimaryObjects_Intensity_IntegratedIntensity_Hematoxylin_cell20	99.16(32.01)	100.94(30.39)	87.81(41.52)	0.284
295	Median_IdentifyPrimaryObjects_Intensity_LowerQuartileIntensity_Hematoxylin_cell20	0.43(0.15)	0.44(0.13)	0.35(0.23)	0.625
296	Median_IdentifyPrimaryObjects_Intensity_MADIntensity_Hematoxylin_cell20	0.09(0.02)	0.09(0.03)	0.09(0.01)	0.936
297	Median_IdentifyPrimaryObjects_Intensity_MassDisplacement_Hematoxylin_cell20	0.29(0.14)	0.28(0.13)	0.37(0.20)	0.096
298	Median_IdentifyPrimaryObjects_Intensity_MaxIntensityEdge_Hematoxylin_cell20	0.58(0.13)	0.59(0.12)	0.52(0.20)	0.329
299	Median_IdentifyPrimaryObjects_Intensity_MaxIntensity_Hematoxylin_cell20	0.80(0.12)	0.81(0.12)	0.74(0.17)	0.119
300	Median_IdentifyPrimaryObjects_Intensity_MeanIntensityEdge_Hematoxylin_cell20	0.41(0.14)	0.42(0.12)	0.34(0.22)	0.55
301	Median_IdentifyPrimaryObjects_Intensity_MeanIntensity_Hematoxylin_cell20	0.53(0.15)	0.54(0.13)	0.45(0.23)	0.609
302	Median_IdentifyPrimaryObjects_Intensity_MedianIntensity_Hematoxylin_cell20	0.52(0.15)	0.53(0.14)	0.45(0.23)	0.594
303	Median_IdentifyPrimaryObjects_Intensity_MinIntensityEdge_Hematoxylin_cell20	0.26(0.15)	0.27(0.15)	0.22(0.19)	0.527
304	Median_IdentifyPrimaryObjects_Intensity_MinIntensity_Hematoxylin_cell20	0.25(0.16)	0.26(0.16)	0.22(0.19)	0.541
305	Median_IdentifyPrimaryObjects_Intensity_StdIntensityEdge_Hematoxylin_cell20	0.07(0.02)	0.07(0.02)	0.07(0.01)	0.761
306	Median_IdentifyPrimaryObjects_Intensity_StdIntensity_Hematoxylin_cell20	0.13(0.03)	0.13(0.04)	0.13(0.01)	0.8
307	Median_IdentifyPrimaryObjects_Intensity_UpperQuartileIntensity_Hematoxylin_cell20	0.63(0.16)	0.64(0.14)	0.55(0.25)	0.137

308	Median_IdentifyPrimaryObjects_Location_CenterMassIntensity_X_Hematoxylin_cell20	609.89(94.00)	606.94(95.95)	628.72(83.57)	0.547
309	Median_IdentifyPrimaryObjects_Location_CenterMassIntensity_Y_Hematoxylin_cell20	572.98(79.23)	571.91(80.10)	579.80(78.18)	0.796
310	Median_IdentifyPrimaryObjects_Location_Center_X_cell20	609.88(94.03)	606.93(95.98)	628.68(83.67)	0.548
311	Median_IdentifyPrimaryObjects_Location_Center_Y_cell20	572.99(79.24)	571.92(80.11)	579.78(78.31)	0.797
312	Median_IdentifyPrimaryObjects_Location_MaxIntensity_X_Hematoxylin_cell20	610.13(94.29)	607.23(96.24)	628.62(84.00)	0.555
313	Median_IdentifyPrimaryObjects_Location_MaxIntensity_Y_Hematoxylin_cell20	573.03(79.32)	571.88(80.22)	580.31(78.13)	0.783
314	Median_IdentifyPrimaryObjects_Texture_AngularSecondMoment_Hematoxylin_3_00_256_cell20	0.00(0.00)	0.00(0.00)	0.00(0.00)	0.576
315	Median_IdentifyPrimaryObjects_Texture_AngularSecondMoment_Hematoxylin_3_01_256_cell20	0.01(0.00)	0.01(0.00)	0.01(0.00)	0.679
316	Median_IdentifyPrimaryObjects_Texture_AngularSecondMoment_Hematoxylin_3_02_256_cell20	0.00(0.00)	0.00(0.00)	0.00(0.00)	0.783
317	Median_IdentifyPrimaryObjects_Texture_AngularSecondMoment_Hematoxylin_3_03_256_cell20	0.01(0.00)	0.01(0.00)	0.01(0.00)	0.696
318	Median_IdentifyPrimaryObjects_Texture_Contrast_Hematoxylin_3_00_256_cell20	1607.11(927.70)	1629.80(986.24)	1462.44(394.48)	0.639
319	Median_IdentifyPrimaryObjects_Texture_Contrast_Hematoxylin_3_01_256_cell20	1995.72(1036.64)	2022.18(1102.12)	1827.04(436.11)	0.625
320	Median_IdentifyPrimaryObjects_Texture_Contrast_Hematoxylin_3_02_256_cell20	1668.23(929.25)	1679.59(985.10)	1595.83(465.02)	0.815
321	Median_IdentifyPrimaryObjects_Texture_Contrast_Hematoxylin_3_03_256_cell20	1973.70(1027.48)	2000.58(1090.29)	1802.31(466.55)	0.616
322	Median_IdentifyPrimaryObjects_Texture_Correlation_Hematoxylin_3_00_256_cell20	0.21(0.11)	0.22(0.11)	0.19(0.14)	0.548

323	Median_IdentifyPrimaryObjects_Texture_Correlation_Hematoxylin_3_01_256_cell20	0.03(0.07)	0.03(0.07)	0.01(0.06)	0.378
324	Median_IdentifyPrimaryObjects_Texture_Correlation_Hematoxylin_3_02_256_cell20	0.18(0.11)	0.19(0.10)	0.12(0.14)	0.109
325	Median_IdentifyPrimaryObjects_Texture_Correlation_Hematoxylin_3_03_256_cell20	0.04(0.07)	0.04(0.07)	0.02(0.08)	0.49
326	Median_IdentifyPrimaryObjects_Texture_DifferenceEntropy_Hematoxylin_3_00_256_cell20	5.55(0.23)	5.55(0.25)	5.56(0.11)	0.747
327	Median_IdentifyPrimaryObjects_Texture_DifferenceEntropy_Hematoxylin_3_01_256_cell20	5.54(0.21)	5.54(0.23)	5.54(0.14)	0.967
328	Median_IdentifyPrimaryObjects_Texture_DifferenceEntropy_Hematoxylin_3_02_256_cell20	5.54(0.23)	5.54(0.24)	5.57(0.12)	0.784
329	Median_IdentifyPrimaryObjects_Texture_DifferenceEntropy_Hematoxylin_3_03_256_cell20	5.55(0.21)	5.55(0.22)	5.55(0.14)	0.982
330	Median_IdentifyPrimaryObjects_Texture_DifferenceVariance_Hematoxylin_3_00_256_cell20	0.00(0.00)	0.00(0.00)	0.00(0.00)	0.401
331	Median_IdentifyPrimaryObjects_Texture_DifferenceVariance_Hematoxylin_3_01_256_cell20	0.00(0.00)	0.00(0.00)	0.00(0.00)	0.493
332	Median_IdentifyPrimaryObjects_Texture_DifferenceVariance_Hematoxylin_3_02_256_cell20	0.00(0.00)	0.00(0.00)	0.00(0.00)	0.594
333	Median_IdentifyPrimaryObjects_Texture_DifferenceVariance_Hematoxylin_3_03_256_cell20	0.00(0.00)	0.00(0.00)	0.00(0.00)	0.465
334	Median_IdentifyPrimaryObjects_Texture_Entropy_Hematoxylin_3_00_256_cell20	7.89(0.31)	7.89(0.32)	7.93(0.21)	0.718
335	Median_IdentifyPrimaryObjects_Texture_Entropy_Hematoxylin_3_01_256_cell20	7.62(0.34)	7.62(0.35)	7.64(0.22)	0.849
336	Median_IdentifyPrimaryObjects_Texture_Entropy_Hematoxylin_3_02_256_cell20	7.86(0.31)	7.86(0.33)	7.87(0.22)	0.929
337	Median_IdentifyPrimaryObjects_Texture_Entropy_Hematoxylin_3_03_256_cell20	7.66(0.34)	7.66(0.35)	7.68(0.24)	0.863
338	Median_IdentifyPrimaryObjects_Texture_InfoMeas1_Hematoxylin_3_00_256_cell20	-0.69(0.06)	-0.69(0.06)	-0.70(0.03)	0.794
339	Median_IdentifyPrimaryObjects_Texture_InfoMeas1_Hematoxylin_3_01_256_cell20	-0.73(0.06)	-0.73(0.06)	-0.74(0.03)	0.773

340	Median_IdentifyPrimaryObjects_Texture_InfoMeas1_Hematoxylin_3_02_256_cell20	-0.70(0.05)	-0.70(0.06)	-0.71(0.04)	0.661
341	Median_IdentifyPrimaryObjects_Texture_InfoMeas1_Hematoxylin_3_03_256_cell20	-0.73(0.06)	-0.72(0.06)	-0.73(0.04)	0.74
342	Median_IdentifyPrimaryObjects_Texture_InfoMeas2_Hematoxylin_3_00_256_cell20	1.00(0.00)	1.00(0.00)	1.00(0.00)	0.974
343	Median_IdentifyPrimaryObjects_Texture_InfoMeas2_Hematoxylin_3_01_256_cell20	1.00(0.00)	1.00(0.00)	1.00(0.00)	0.974
344	Median_IdentifyPrimaryObjects_Texture_InfoMeas2_Hematoxylin_3_02_256_cell20	1.00(0.00)	1.00(0.00)	1.00(0.00)	0.888
345	Median_IdentifyPrimaryObjects_Texture_InfoMeas2_Hematoxylin_3_03_256_cell20	1.00(0.00)	1.00(0.00)	1.00(0.00)	0.957
346	Median_IdentifyPrimaryObjects_Texture_InverseDifferenceMoment_Hematoxylin_3_00_256_cell20	0.04(0.01)	0.04(0.01)	0.04(0.01)	0.622
347	Median_IdentifyPrimaryObjects_Texture_InverseDifferenceMoment_Hematoxylin_3_01_256_cell20	0.03(0.01)	0.03(0.01)	0.03(0.00)	0.682
348	Median_IdentifyPrimaryObjects_Texture_InverseDifferenceMoment_Hematoxylin_3_02_256_cell20	0.04(0.01)	0.04(0.01)	0.04(0.01)	0.399
349	Median_IdentifyPrimaryObjects_Texture_InverseDifferenceMoment_Hematoxylin_3_03_256_cell20	0.03(0.01)	0.03(0.01)	0.03(0.00)	0.674
350	Median_IdentifyPrimaryObjects_Texture_SumAverage_Hematoxylin_3_00_256_cell20	281.90(73.21)	287.52(64.25)	246.08(115.00)	0.625
351	Median_IdentifyPrimaryObjects_Texture_SumAverage_Hematoxylin_3_01_256_cell20	280.06(73.88)	285.72(64.84)	243.98(116.05)	0.609
352	Median_IdentifyPrimaryObjects_Texture_SumAverage_Hematoxylin_3_02_256_cell20	281.61(73.52)	287.27(64.50)	245.53(115.56)	0.609
353	Median_IdentifyPrimaryObjects_Texture_SumAverage_Hematoxylin_3_03_256_cell20	280.61(73.69)	286.24(64.66)	244.69(115.86)	0.625
354	Median_IdentifyPrimaryObjects_Texture_SumEntropy_Hematoxylin_3_00_256_cell20	6.23(0.23)	6.23(0.24)	6.23(0.19)	0.985

355	Median_IdentifyPrimaryObjects_Texture_SumEntropy_Hematoxylin_3_01_256_cell20	6.01(0.25)	6.01(0.26)	6.01(0.17)	0.969
356	Median_IdentifyPrimaryObjects_Texture_SumEntropy_Hematoxylin_3_02_256_cell20	6.20(0.24)	6.20(0.25)	6.17(0.18)	0.782
357	Median_IdentifyPrimaryObjects_Texture_SumEntropy_Hematoxylin_3_03_256_cell20	6.04(0.25)	6.04(0.26)	6.04(0.19)	0.985
358	Median_IdentifyPrimaryObjects_Texture_SumVariance_Hematoxylin_3_00_256_cell20	2556.81(1259.37)	2610.21(1324.97)	2216.39(669.69)	0.416
359	Median_IdentifyPrimaryObjects_Texture_SumVariance_Hematoxylin_3_01_256_cell20	2145.96(1037.24)	2193.47(1093.84)	1843.04(496.60)	0.379
360	Median_IdentifyPrimaryObjects_Texture_SumVariance_Hematoxylin_3_02_256_cell20	2490.05(1235.10)	2554.89(1297.93)	2076.72(617.10)	0.313
361	Median_IdentifyPrimaryObjects_Texture_SumVariance_Hematoxylin_3_03_256_cell20	2169.33(1054.00)	2214.90(1112.91)	1878.80(496.19)	0.406
362	Median_IdentifyPrimaryObjects_Texture_Variance_Hematoxylin_3_00_256_cell20	1095.15(556.88)	1115.47(591.10)	965.60(227.28)	0.484
363	Median_IdentifyPrimaryObjects_Texture_Variance_Hematoxylin_3_01_256_cell20	1095.01(542.50)	1114.69(575.03)	969.57(236.55)	0.487
364	Median_IdentifyPrimaryObjects_Texture_Variance_Hematoxylin_3_02_256_cell20	1092.81(552.19)	1112.92(585.81)	964.59(231.38)	0.485
365	Median_IdentifyPrimaryObjects_Texture_Variance_Hematoxylin_3_03_256_cell20	1096.64(543.46)	1116.39(575.88)	970.75(239.43)	0.486
366	StDev_IdentifyPrimaryObjects_AreaShape_Area_cell20	130.73(41.87)	127.61(40.64)	150.61(46.93)	0.15
367	StDev_IdentifyPrimaryObjects_AreaShape_BoundingBoxArea_cell20	289.67(100.96)	281.68(93.93)	340.61(134.22)	0.126
368	StDev_IdentifyPrimaryObjects_AreaShape_BoundingBoxMaximum_X_cell20	338.41(47.00)	338.29(46.90)	339.22(50.92)	0.579
369	StDev_IdentifyPrimaryObjects_AreaShape_BoundingBoxMaximum_Y_cell20	321.66(43.24)	321.27(43.91)	324.15(41.42)	0.888
370	StDev_IdentifyPrimaryObjects_AreaShape_BoundingBoxMinimum_X_cell20	338.35(46.90)	338.22(46.89)	339.23(50.20)	0.594
371	StDev_IdentifyPrimaryObjects_AreaShape_BoundingBoxMinimum_Y_cell20	321.75(43.24)	321.34(43.94)	324.35(41.13)	0.939

372	StDev_IdentifyPrimaryObjects_AreaShape_Center_X_cell20	338.36(46.95)	338.23(46.89)	339.18(50.57)	0.594
373	StDev_IdentifyPrimaryObjects_AreaShape_Center_Y_cell20	321.69(43.24)	321.29(43.92)	324.23(41.27)	0.922
374	StDev_IdentifyPrimaryObjects_AreaShape_CentralMoment_0_0_cell20	130.73(41.87)	127.61(40.64)	150.61(46.93)	0.15
375	StDev_IdentifyPrimaryObjects_AreaShape_CentralMoment_0_1_cell20	0.00(0.00)	0.00(0.00)	0.00(0.00)	0.057
376	StDev_IdentifyPrimaryObjects_AreaShape_CentralMoment_0_2_cell20	14858.22(9759.99)	13786.68(8411.14)	21689.29(14907.84)	0.183
377	StDev_IdentifyPrimaryObjects_AreaShape_CentralMoment_0_3_cell20	64312.73(64032.89)	55632.22(41968.11)	119651.00(131694.80)	0.214
378	StDev_IdentifyPrimaryObjects_AreaShape_CentralMoment_1_0_cell20	0.00(0.00)	0.00(0.00)	0.00(0.00)	0.374
379	StDev_IdentifyPrimaryObjects_AreaShape_CentralMoment_1_1_cell20	6008.68(3652.17)	5632.08(3232.60)	8409.49(5315.65)	0.19
380	StDev_IdentifyPrimaryObjects_AreaShape_CentralMoment_1_2_cell20*	34806.50(26886.54)	31849.57(23653.98)	53656.95(39029.67)	0.032
381	StDev_IdentifyPrimaryObjects_AreaShape_CentralMoment_1_3_cell20	1731959.84(1711193.29)	1488805.93(1369509.98)	3282065.99(2772795.34)	0.113
382	StDev_IdentifyPrimaryObjects_AreaShape_CentralMoment_2_0_cell20	12341.28(7287.43)	11875.23(6969.37)	15312.35(9022.82)	0.218
383	StDev_IdentifyPrimaryObjects_AreaShape_CentralMoment_2_1_cell20	32690.83(24590.06)	30506.10(22445.62)	46618.52(33986.03)	0.085
384	StDev_IdentifyPrimaryObjects_AreaShape_CentralMoment_2_2_cell20	1359287.30(1192828.76)	1199376.41(983937.35)	2378719.22(1870829.98)	0.121
385	StDev_IdentifyPrimaryObjects_AreaShape_CentralMoment_2_3_cell20	12100276.75(13582455.11)	10081375.66(10482399.04)	24970771.24(22906373.07)	0.192
386	StDev_IdentifyPrimaryObjects_AreaShape_Compactness_cell20	0.66(0.25)	0.66(0.26)	0.65(0.21)	0.912
387	StDev_IdentifyPrimaryObjects_AreaShape_ConvexArea_cell20	183.01(63.08)	178.14(59.41)	214.01(80.56)	0.136
388	StDev_IdentifyPrimaryObjects_AreaShape_Eccentricity_cell20	0.14(0.00)	0.14(0.00)	0.14(0.01)	0.834

389	StDev_IdentifyPrimaryObjects_AreaShape_EquivalentDiameter_cell20	4.41(0.98)	4.33(0.95)	4.87(1.08)	0.148
390	StDev_IdentifyPrimaryObjects_AreaShape_EulerNumber_cell20	0.04(0.03)	0.04(0.03)	0.06(0.03)	0.052
391	StDev_IdentifyPrimaryObjects_AreaShape_Extent_cell20	0.11(0.01)	0.11(0.01)	0.11(0.01)	0.347
392	StDev_IdentifyPrimaryObjects_AreaShape_FormFactor_cell20	0.17(0.01)	0.17(0.01)	0.17(0.01)	0.39
393	StDev_IdentifyPrimaryObjects_AreaShape_HuMoment_0_cell20	0.07(0.02)	0.07(0.03)	0.07(0.02)	0.769
394	StDev_IdentifyPrimaryObjects_AreaShape_HuMoment_1_cell20	0.04(0.03)	0.04(0.03)	0.04(0.02)	0.521
395	StDev_IdentifyPrimaryObjects_AreaShape_HuMoment_2_cell20	0.01(0.01)	0.01(0.01)	0.01(0.00)	0.888
396	StDev_IdentifyPrimaryObjects_AreaShape_HuMoment_3_cell20	0.00(0.00)	0.00(0.00)	0.00(0.00)	0.401
397	StDev_IdentifyPrimaryObjects_AreaShape_HuMoment_4_cell20	0.00(0.00)	0.00(0.00)	0.00(0.00)	0.269
398	StDev_IdentifyPrimaryObjects_AreaShape_HuMoment_5_cell20	0.00(0.00)	0.00(0.00)	0.00(0.00)	0.64
399	StDev_IdentifyPrimaryObjects_AreaShape_HuMoment_6_cell20	0.00(0.00)	0.00(0.00)	0.00(0.00)	0.871
400	StDev_IdentifyPrimaryObjects_AreaShape_InertiaTensorEigenvalues_0_cell20	29.83(11.62)	28.69(10.14)	37.05(17.75)	0.231
401	StDev_IdentifyPrimaryObjects_AreaShape_InertiaTensorEigenvalues_1_cell20	8.73(2.69)	8.54(2.61)	9.90(3.08)	0.186
402	StDev_IdentifyPrimaryObjects_AreaShape_InertiaTensor_0_0_cell20	23.74(9.82)	22.62(8.28)	30.82(15.58)	0.185
403	StDev_IdentifyPrimaryObjects_AreaShape_InertiaTensor_0_1_cell20	12.62(4.19)	12.26(3.72)	14.94(6.28)	0.274
404	StDev_IdentifyPrimaryObjects_AreaShape_InertiaTensor_1_0_cell20	12.62(4.19)	12.26(3.72)	14.94(6.28)	0.274
405	StDev_IdentifyPrimaryObjects_AreaShape_InertiaTensor_1_1_cell20	19.80(7.16)	19.50(6.95)	21.73(8.67)	0.417

406	StDev_IdentifyPrimaryObjects_AreaShape_MajorAxisLength_cell20	7.99(1.89)	7.83(1.70)	9.06(2.75)	0.252
407	StDev_IdentifyPrimaryObjects_AreaShape_MaxFeretDiameter_cell20	8.18(1.86)	8.02(1.71)	9.15(2.56)	0.111
408	StDev_IdentifyPrimaryObjects_AreaShape_MaximumRadius_cell20	1.57(0.29)	1.56(0.29)	1.66(0.25)	0.39
409	StDev_IdentifyPrimaryObjects_AreaShape_MeanRadius_cell20	0.50(0.09)	0.50(0.09)	0.52(0.07)	0.543
410	StDev_IdentifyPrimaryObjects_AreaShape_MedianRadius_cell20	0.45(0.08)	0.45(0.08)	0.46(0.07)	0.983
411	StDev_IdentifyPrimaryObjects_AreaShape_MinFeretDiameter_cell20	4.71(1.00)	4.64(0.97)	5.16(1.14)	0.178
412	StDev_IdentifyPrimaryObjects_AreaShape_MinorAxisLength_cell20	4.30(0.87)	4.24(0.85)	4.68(0.96)	0.184
413	StDev_IdentifyPrimaryObjects_AreaShape_NormalizedMoment_0_2_cell20	0.07(0.02)	0.06(0.02)	0.07(0.02)	0.539
414	StDev_IdentifyPrimaryObjects_AreaShape_NormalizedMoment_0_3_cell20	0.02(0.01)	0.02(0.01)	0.02(0.01)	0.948
415	StDev_IdentifyPrimaryObjects_AreaShape_NormalizedMoment_1_1_cell20	0.05(0.01)	0.05(0.01)	0.05(0.01)	0.983
416	StDev_IdentifyPrimaryObjects_AreaShape_NormalizedMoment_1_2_cell20	0.01(0.00)	0.01(0.00)	0.01(0.00)	0.784
417	StDev_IdentifyPrimaryObjects_AreaShape_NormalizedMoment_1_3_cell20	0.02(0.01)	0.02(0.01)	0.02(0.01)	0.452
418	StDev_IdentifyPrimaryObjects_AreaShape_NormalizedMoment_2_0_cell20	0.05(0.01)	0.06(0.02)	0.05(0.01)	0.381
419	StDev_IdentifyPrimaryObjects_AreaShape_NormalizedMoment_2_1_cell20	0.01(0.00)	0.01(0.00)	0.01(0.00)	0.437
420	StDev_IdentifyPrimaryObjects_AreaShape_NormalizedMoment_2_2_cell20	0.01(0.01)	0.01(0.01)	0.01(0.00)	0.957
421	StDev_IdentifyPrimaryObjects_AreaShape_NormalizedMoment_2_3_cell20	0.01(0.00)	0.01(0.00)	0.01(0.00)	0.854
422	StDev_IdentifyPrimaryObjects_AreaShape_NormalizedMoment_3_0_cell20	0.02(0.01)	0.02(0.01)	0.01(0.00)	0.211

423	StDev_IdentifyPrimaryObjects_AreaShape_NormalizedMoment_3_1_cell20	0.02(0.01)	0.02(0.01)	0.02(0.00)	0.704
424	StDev_IdentifyPrimaryObjects_AreaShape_NormalizedMoment_3_2_cell20	0.01(0.00)	0.01(0.00)	0.00(0.00)	0.736
425	StDev_IdentifyPrimaryObjects_AreaShape_NormalizedMoment_3_3_cell20	0.01(0.01)	0.01(0.01)	0.01(0.00)	0.991
426	StDev_IdentifyPrimaryObjects_AreaShape_Orientation_cell20	54.94(4.02)	54.57(4.05)	57.27(3.15)	0.078
427	StDev_IdentifyPrimaryObjects_AreaShape_Perimeter_cell20	27.26(7.55)	26.77(7.11)	30.38(9.89)	0.211
428	StDev_IdentifyPrimaryObjects_AreaShape_Solidity_cell20	0.09(0.02)	0.09(0.02)	0.09(0.01)	0.564
429	StDev_IdentifyPrimaryObjects_AreaShape_SpatialMoment_0_0_cell20	130.73(41.87)	127.61(40.64)	150.61(46.93)	0.15
430	StDev_IdentifyPrimaryObjects_AreaShape_SpatialMoment_0_1_cell20	2828.44(1302.75)	2710.03(1208.35)	3583.26(1696.89)	0.078
431	StDev_IdentifyPrimaryObjects_AreaShape_SpatialMoment_0_2_cell20	80875.25(51328.54)	75397.52(44749.40)	115795.80(76901.28)	0.187
432	StDev_IdentifyPrimaryObjects_AreaShape_SpatialMoment_0_3_cell20	2912686.04(2567091.83)	2591343.88(1983872.18)	4961242.31(4576200.90)	0.19
433	StDev_IdentifyPrimaryObjects_AreaShape_SpatialMoment_1_0_cell20	2555.21(1121.51)	2481.81(1082.00)	3023.14(1330.35)	0.207
434	StDev_IdentifyPrimaryObjects_AreaShape_SpatialMoment_1_1_cell20	49705.57(28847.03)	47285.03(26805.63)	65136.53(38048.79)	0.104
435	StDev_IdentifyPrimaryObjects_AreaShape_SpatialMoment_1_2_cell20	1387194.85(1020267.89)	1286826.09(911754.22)	2027045.70(1464877.93)	0.203
436	StDev_IdentifyPrimaryObjects_AreaShape_SpatialMoment_1_3_cell20	49475387.26(45920706.22)	44406468.42(39407243.82)	81789744.89(70705200.08)	0.184
437	StDev_IdentifyPrimaryObjects_AreaShape_SpatialMoment_2_0_cell20	65636.55(38220.02)	63253.08(36631.05)	80831.17(47050.50)	0.23
438	StDev_IdentifyPrimaryObjects_AreaShape_SpatialMoment_2_1_cell20	1249433.68(904021.91)	1173612.91(832304.01)	1732791.14(1230682.92)	0.104
439	StDev_IdentifyPrimaryObjects_AreaShape_SpatialMoment_2_2_cell20	34945268.71(30806148.06)	31852152.46(27294438.89)	54663884.82(45032118.56)	0.202

440	StDev_IdentifyPrimaryObjects_AreaShape_SpatialMoment_2_3_cell20	1254943964.09(1345578715.93)	1105511023.66(1166106406.66)	2207578959.31(2022184294.30)	0.26
441	StDev_IdentifyPrimaryObjects_AreaShape_Zernike_0_0_cell20	0.15(0.01)	0.15(0.01)	0.14(0.01)	0.28
442	StDev_IdentifyPrimaryObjects_AreaShape_Zernike_1_1_cell20	0.03(0.00)	0.03(0.00)	0.03(0.00)	0.384
443	StDev_IdentifyPrimaryObjects_AreaShape_Zernike_2_0_cell20	0.04(0.00)	0.04(0.00)	0.03(0.00)	0.559
444	StDev_IdentifyPrimaryObjects_AreaShape_Zernike_2_2_cell20	0.02(0.00)	0.02(0.00)	0.02(0.00)	0.929
445	StDev_IdentifyPrimaryObjects_AreaShape_Zernike_3_1_cell20	0.02(0.00)	0.02(0.00)	0.02(0.00)	0.785
446	StDev_IdentifyPrimaryObjects_AreaShape_Zernike_3_3_cell20	0.02(0.00)	0.02(0.00)	0.02(0.00)	0.105
447	StDev_IdentifyPrimaryObjects_AreaShape_Zernike_4_0_cell20	0.02(0.00)	0.02(0.00)	0.02(0.00)	0.373
448	StDev_IdentifyPrimaryObjects_AreaShape_Zernike_4_2_cell20	0.01(0.00)	0.01(0.00)	0.01(0.00)	0.06
449	StDev_IdentifyPrimaryObjects_AreaShape_Zernike_4_4_cell20	0.01(0.00)	0.01(0.00)	0.01(0.00)	0.63
450	StDev_IdentifyPrimaryObjects_AreaShape_Zernike_5_1_cell20	0.01(0.00)	0.01(0.00)	0.01(0.00)	0.526
451	StDev_IdentifyPrimaryObjects_AreaShape_Zernike_5_3_cell20	0.01(0.00)	0.01(0.00)	0.01(0.00)	0.464
452	StDev_IdentifyPrimaryObjects_AreaShape_Zernike_5_5_cell20	0.01(0.00)	0.01(0.00)	0.01(0.00)	0.24
453	StDev_IdentifyPrimaryObjects_AreaShape_Zernike_6_0_cell20	0.01(0.00)	0.01(0.00)	0.01(0.00)	0.412
454	StDev_IdentifyPrimaryObjects_AreaShape_Zernike_6_2_cell20	0.01(0.00)	0.01(0.00)	0.01(0.00)	0.759
455	StDev_IdentifyPrimaryObjects_AreaShape_Zernike_6_4_cell20	0.01(0.00)	0.01(0.00)	0.01(0.00)	0.74
456	StDev_IdentifyPrimaryObjects_AreaShape_Zernike_6_6_cell20	0.01(0.00)	0.01(0.00)	0.01(0.00)	0.871

457	StDev_IdentifyPrimaryObjects_AreaShape_Zernike_7_1_cell20	0.01(0.00)	0.01(0.00)	0.01(0.00)	0.394
458	StDev_IdentifyPrimaryObjects_AreaShape_Zernike_7_3_cell20	0.01(0.00)	0.01(0.00)	0.01(0.00)	0.606
459	StDev_IdentifyPrimaryObjects_AreaShape_Zernike_7_5_cell20	0.01(0.00)	0.01(0.00)	0.01(0.00)	0.877
460	StDev_IdentifyPrimaryObjects_AreaShape_Zernike_7_7_cell20	0.01(0.00)	0.01(0.00)	0.01(0.00)	0.935
461	StDev_IdentifyPrimaryObjects_AreaShape_Zernike_8_0_cell20	0.01(0.00)	0.01(0.00)	0.01(0.00)	0.872
462	StDev_IdentifyPrimaryObjects_AreaShape_Zernike_8_2_cell20	0.01(0.00)	0.01(0.00)	0.01(0.00)	0.535
463	StDev_IdentifyPrimaryObjects_AreaShape_Zernike_8_4_cell20	0.00(0.00)	0.00(0.00)	0.00(0.00)	0.357
464	StDev_IdentifyPrimaryObjects_AreaShape_Zernike_8_6_cell20	0.00(0.00)	0.00(0.00)	0.00(0.00)	0.773
465	StDev_IdentifyPrimaryObjects_AreaShape_Zernike_8_8_cell20	0.00(0.00)	0.00(0.00)	0.00(0.00)	0.446
466	StDev_IdentifyPrimaryObjects_AreaShape_Zernike_9_1_cell20	0.00(0.00)	0.00(0.00)	0.00(0.00)	0.179
467	StDev_IdentifyPrimaryObjects_AreaShape_Zernike_9_3_cell20	0.00(0.00)	0.00(0.00)	0.00(0.00)	0.747
468	StDev_IdentifyPrimaryObjects_AreaShape_Zernike_9_5_cell20	0.00(0.00)	0.00(0.00)	0.00(0.00)	0.474
469	StDev_IdentifyPrimaryObjects_AreaShape_Zernike_9_7_cell20	0.00(0.00)	0.00(0.00)	0.00(0.00)	0.888
470	StDev_IdentifyPrimaryObjects_AreaShape_Zernike_9_9_cell20	0.00(0.00)	0.00(0.00)	0.00(0.00)	0.438
471	StDev_IdentifyPrimaryObjects_Intensity_IntegratedIntensityEdge_Hematoxylin_cell20	9.28(2.89)	9.48(2.75)	8.02(3.60)	0.187
472	StDev_IdentifyPrimaryObjects_Intensity_IntegratedIntensity_Hematoxylin_cell20	66.58(24.23)	67.44(24.58)	61.12(22.61)	0.498
473	StDev_IdentifyPrimaryObjects_Intensity_LowerQuartileIntensity_Hematoxylin_cell20	0.05(0.01)	0.04(0.01)	0.05(0.01)	0.211

474	StDev_IdentifyPrimaryObjects_Intensity_MADIntensity_Hematoxylin_cell20	0.04(0.02)	0.04(0.02)	0.04(0.01)	0.786
475	StDev_IdentifyPrimaryObjects_Intensity_MassDisplacement_Hematoxylin_cell20	0.33(0.19)	0.31(0.18)	0.43(0.25)	0.17
476	StDev_IdentifyPrimaryObjects_Intensity_MaxIntensityEdge_Hematoxylin_cell20	0.07(0.02)	0.07(0.02)	0.08(0.02)	0.353
477	StDev_IdentifyPrimaryObjects_Intensity_MaxIntensity_Hematoxylin_cell20	0.10(0.04)	0.10(0.04)	0.11(0.05)	0.331
478	StDev_IdentifyPrimaryObjects_Intensity_MeanIntensityEdge_Hematoxylin_cell20	0.04(0.01)	0.03(0.01)	0.04(0.01)	0.318
479	StDev_IdentifyPrimaryObjects_Intensity_MeanIntensity_Hematoxylin_cell20	0.06(0.02)	0.06(0.02)	0.06(0.02)	0.629
480	StDev_IdentifyPrimaryObjects_Intensity_MedianIntensity_Hematoxylin_cell20	0.07(0.02)	0.07(0.02)	0.07(0.02)	0.68
481	StDev_IdentifyPrimaryObjects_Intensity_MinIntensityEdge_Hematoxylin_cell20	0.07(0.03)	0.07(0.03)	0.06(0.04)	0.346
482	StDev_IdentifyPrimaryObjects_Intensity_MinIntensity_Hematoxylin_cell20	0.07(0.03)	0.07(0.03)	0.06(0.04)	0.374
483	StDev_IdentifyPrimaryObjects_Intensity_StdIntensityEdge_Hematoxylin_cell20	0.03(0.01)	0.03(0.01)	0.03(0.01)	0.936
484	StDev_IdentifyPrimaryObjects_Intensity_StdIntensity_Hematoxylin_cell20	0.04(0.02)	0.04(0.02)	0.04(0.02)	0.82
485	StDev_IdentifyPrimaryObjects_Intensity_UpperQuartileIntensity_Hematoxylin_cell20	0.09(0.03)	0.09(0.03)	0.09(0.03)	0.905
486	StDev_IdentifyPrimaryObjects_Location_CenterMassIntensity_X_Hematoxylin_cell20	338.36(46.95)	338.23(46.89)	339.19(50.57)	0.594
487	StDev_IdentifyPrimaryObjects_Location_CenterMassIntensity_Y_Hematoxylin_cell20	321.69(43.24)	321.29(43.92)	324.23(41.26)	0.922
488	StDev_IdentifyPrimaryObjects_Location_Center_X_cell20	338.36(46.95)	338.23(46.89)	339.18(50.57)	0.594
489	StDev_IdentifyPrimaryObjects_Location_Center_Y_cell20	321.69(43.24)	321.29(43.92)	324.23(41.27)	0.922
490	StDev_IdentifyPrimaryObjects_Location_MaxIntensity_X_Hematoxylin_cell20	338.37(46.97)	338.24(46.91)	339.24(50.54)	0.594

491	StDev_IdentifyPrimaryObjects_Location_MaxIntensity_Y_Hematoxylin_cell20	321.71(43.23)	321.31(43.91)	324.25(41.25)	0.905
492	StDev_IdentifyPrimaryObjects_Texture_AngularSecondMoment_Hematoxylin_3_00_256_cell20	0.00(0.00)	0.00(0.00)	0.00(0.00)	0.672
493	StDev_IdentifyPrimaryObjects_Texture_AngularSecondMoment_Hematoxylin_3_01_256_cell20	0.01(0.00)	0.01(0.00)	0.01(0.01)	0.579
494	StDev_IdentifyPrimaryObjects_Texture_AngularSecondMoment_Hematoxylin_3_02_256_cell20	0.00(0.00)	0.00(0.00)	0.00(0.00)	0.203
495	StDev_IdentifyPrimaryObjects_Texture_AngularSecondMoment_Hematoxylin_3_03_256_cell20	0.01(0.00)	0.01(0.00)	0.01(0.00)	0.414
496	StDev_IdentifyPrimaryObjects_Texture_Contrast_Hematoxylin_3_00_256_cell20	1223.02(784.65)	1235.38(820.63)	1144.23(531.87)	0.939
497	StDev_IdentifyPrimaryObjects_Texture_Contrast_Hematoxylin_3_01_256_cell20	1594.99(992.49)	1601.00(1030.87)	1556.69(754.39)	0.908
498	StDev_IdentifyPrimaryObjects_Texture_Contrast_Hematoxylin_3_02_256_cell20	1285.12(819.94)	1284.46(856.29)	1289.31(577.09)	0.535
499	StDev_IdentifyPrimaryObjects_Texture_Contrast_Hematoxylin_3_03_256_cell20	1566.90(998.16)	1573.56(1033.96)	1524.42(785.27)	0.898
500	StDev_IdentifyPrimaryObjects_Texture_Correlation_Hematoxylin_3_00_256_cell20	0.24(0.03)	0.24(0.03)	0.24(0.02)	0.401
501	StDev_IdentifyPrimaryObjects_Texture_Correlation_Hematoxylin_3_01_256_cell20	0.25(0.03)	0.25(0.03)	0.24(0.04)	0.492
502	StDev_IdentifyPrimaryObjects_Texture_Correlation_Hematoxylin_3_02_256_cell20	0.25(0.03)	0.25(0.03)	0.24(0.03)	0.628
503	StDev_IdentifyPrimaryObjects_Texture_Correlation_Hematoxylin_3_03_256_cell20	0.25(0.03)	0.25(0.03)	0.24(0.04)	0.48
504	StDev_IdentifyPrimaryObjects_Texture_DifferenceEntropy_Hematoxylin_3_00_256_cell20	0.45(0.11)	0.44(0.11)	0.46(0.11)	0.479
505	StDev_IdentifyPrimaryObjects_Texture_DifferenceEntropy_Hematoxylin_3_01_256_cell20	0.56(0.12)	0.55(0.12)	0.57(0.13)	0.658
506	StDev_IdentifyPrimaryObjects_Texture_DifferenceEntropy_Hematoxylin_3_02_256_cell20	0.46(0.11)	0.46(0.11)	0.48(0.12)	0.32
507	StDev_IdentifyPrimaryObjects_Texture_DifferenceEntropy_Hematoxylin_3_03_256_cell20	0.54(0.12)	0.54(0.12)	0.56(0.13)	0.644

508	StDev_IdentifyPrimaryObjects_Texture_DifferenceVariance_Hematoxylin_3_00_256_cell20	0.00(0.00)	0.00(0.00)	0.00(0.00)	0.753
509	StDev_IdentifyPrimaryObjects_Texture_DifferenceVariance_Hematoxylin_3_01_256_cell20	0.00(0.00)	0.00(0.00)	0.00(0.00)	0.535
510	StDev_IdentifyPrimaryObjects_Texture_DifferenceVariance_Hematoxylin_3_02_256_cell20	0.00(0.00)	0.00(0.00)	0.00(0.00)	0.594
511	StDev_IdentifyPrimaryObjects_Texture_DifferenceVariance_Hematoxylin_3_03_256_cell20	0.00(0.00)	0.00(0.00)	0.00(0.00)	0.609
512	StDev_IdentifyPrimaryObjects_Texture_Entropy_Hematoxylin_3_00_256_cell20	0.86(0.12)	0.85(0.12)	0.91(0.14)	0.151
513	StDev_IdentifyPrimaryObjects_Texture_Entropy_Hematoxylin_3_01_256_cell20	0.98(0.13)	0.97(0.13)	1.05(0.16)	0.153
514	StDev_IdentifyPrimaryObjects_Texture_Entropy_Hematoxylin_3_02_256_cell20	0.87(0.12)	0.86(0.12)	0.94(0.14)	0.117
515	StDev_IdentifyPrimaryObjects_Texture_Entropy_Hematoxylin_3_03_256_cell20	0.97(0.13)	0.96(0.12)	1.03(0.16)	0.127
516	StDev_IdentifyPrimaryObjects_Texture_InfoMeas1_Hematoxylin_3_00_256_cell20	0.09(0.03)	0.09(0.03)	0.10(0.04)	0.25
517	StDev_IdentifyPrimaryObjects_Texture_InfoMeas1_Hematoxylin_3_01_256_cell20	0.10(0.03)	0.10(0.03)	0.12(0.03)	0.247
518	StDev_IdentifyPrimaryObjects_Texture_InfoMeas1_Hematoxylin_3_02_256_cell20	0.09(0.03)	0.09(0.03)	0.11(0.04)	0.208
519	StDev_IdentifyPrimaryObjects_Texture_InfoMeas1_Hematoxylin_3_03_256_cell20	0.10(0.03)	0.10(0.03)	0.11(0.03)	0.255
520	StDev_IdentifyPrimaryObjects_Texture_InfoMeas2_Hematoxylin_3_00_256_cell20	0.01(0.01)	0.01(0.01)	0.01(0.01)	0.143
521	StDev_IdentifyPrimaryObjects_Texture_InfoMeas2_Hematoxylin_3_01_256_cell20	0.01(0.01)	0.01(0.01)	0.01(0.01)	0.17
522	StDev_IdentifyPrimaryObjects_Texture_InfoMeas2_Hematoxylin_3_02_256_cell20	0.01(0.01)	0.01(0.01)	0.01(0.01)	0.163
523	StDev_IdentifyPrimaryObjects_Texture_InfoMeas2_Hematoxylin_3_03_256_cell20	0.01(0.01)	0.01(0.01)	0.01(0.01)	0.177
524	StDev_IdentifyPrimaryObjects_Texture_InverseDifferenceMoment_Hematoxylin_3_00_256_cell20	0.02(0.01)	0.02(0.01)	0.02(0.01)	0.656

525	StDev_IdentifyPrimaryObjects_Texture_InverseDifferenceMoment_Hematoxylin_3_01_256_cell20	0.02(0.01)	0.02(0.01)	0.02(0.01)	0.507
526	StDev_IdentifyPrimaryObjects_Texture_InverseDifferenceMoment_Hematoxylin_3_02_256_cell20	0.02(0.01)	0.02(0.01)	0.02(0.01)	0.414
527	StDev_IdentifyPrimaryObjects_Texture_InverseDifferenceMoment_Hematoxylin_3_03_256_cell20	0.02(0.01)	0.02(0.01)	0.02(0.01)	0.389
528	StDev_IdentifyPrimaryObjects_Texture_SumAverage_Hematoxylin_3_00_256_cell20	36.08(13.17)	36.36(13.18)	34.27(13.88)	0.68
529	StDev_IdentifyPrimaryObjects_Texture_SumAverage_Hematoxylin_3_01_256_cell20	35.90(12.82)	36.18(12.79)	34.10(13.74)	0.674
530	StDev_IdentifyPrimaryObjects_Texture_SumAverage_Hematoxylin_3_02_256_cell20	36.11(13.21)	36.40(13.19)	34.21(14.10)	0.666
531	StDev_IdentifyPrimaryObjects_Texture_SumAverage_Hematoxylin_3_03_256_cell20	35.83(12.75)	36.10(12.72)	34.11(13.67)	0.685
532	StDev_IdentifyPrimaryObjects_Texture_SumEntropy_Hematoxylin_3_00_256_cell20	0.62(0.10)	0.62(0.10)	0.63(0.10)	0.815
533	StDev_IdentifyPrimaryObjects_Texture_SumEntropy_Hematoxylin_3_01_256_cell20	0.73(0.11)	0.73(0.11)	0.75(0.12)	0.615
534	StDev_IdentifyPrimaryObjects_Texture_SumEntropy_Hematoxylin_3_02_256_cell20	0.64(0.10)	0.63(0.10)	0.65(0.10)	0.679
535	StDev_IdentifyPrimaryObjects_Texture_SumEntropy_Hematoxylin_3_03_256_cell20	0.72(0.10)	0.71(0.10)	0.73(0.12)	0.594
536	StDev_IdentifyPrimaryObjects_Texture_SumVariance_Hematoxylin_3_00_256_cell20	1968.28(1257.00)	1958.66(1264.30)	2029.61(1291.95)	0.609
537	StDev_IdentifyPrimaryObjects_Texture_SumVariance_Hematoxylin_3_01_256_cell20	1688.82(1054.13)	1687.17(1061.17)	1699.38(1078.56)	0.736
538	StDev_IdentifyPrimaryObjects_Texture_SumVariance_Hematoxylin_3_02_256_cell20	1929.46(1244.45)	1931.46(1253.16)	1916.75(1270.58)	0.888
539	StDev_IdentifyPrimaryObjects_Texture_SumVariance_Hematoxylin_3_03_256_cell20	1712.94(1060.85)	1708.90(1068.58)	1738.71(1080.67)	0.786
540	StDev_IdentifyPrimaryObjects_Texture_Variance_Hematoxylin_3_00_256_cell20	711.17(469.35)	710.48(478.45)	715.59(436.06)	0.977

541	StDev_IdentifyPrimaryObjects_Texture_Variance_Hematoxylin_3_01_256_cell20	725.55(467.79)	725.25(476.48)	727.51(437.55)	0.99
542	StDev_IdentifyPrimaryObjects_Texture_Variance_Hematoxylin_3_02_256_cell20	714.99(471.58)	713.99(480.49)	721.37(439.85)	0.968
543	StDev_IdentifyPrimaryObjects_Texture_Variance_Hematoxylin_3_03_256_cell20	722.74(467.22)	721.93(475.35)	727.93(441.30)	0.973
544	Threshold_FinalThreshold_IdentifyPrimaryObjects_cell20	0.37(0.13)	0.38(0.11)	0.32(0.19)	0.672
545	Threshold_OrigThreshold_IdentifyPrimaryObjects_cell20	0.37(0.13)	0.38(0.11)	0.32(0.19)	0.672
546	Threshold_SumOfEntropies_IdentifyPrimaryObjects_cell20	-10.94(1.25)	-10.82(1.16)	-11.76(1.57)	0.086
547	Threshold_WeightedVariance_IdentifyPrimaryObjects_cell20	0.73(1.07)	0.68(1.03)	1.07(1.32)	0.177
548	Width_HE_cell20	1208.73(155.81)	1205.04(156.37)	1232.25(160.47)	0.241
