# Supplementary material for: Integrating computed tomography and biopsy images to predict chemotherapy response in gastric cancer
Source: Front Oncol. 2025 Oct 21;15:1666358. doi: 10.3389/fonc.2025.1666358 (PMC12583031; doi:10.3389/fonc.2025.1666358)
Supplement: Supplementary file 2 [file DataSheet2.pdf]

Radiomic features of the validation cohort

	Radiomic Feature names	All cases in the Validation Cohort (n=59)	Non pathological complete response cases (n=51)	Pathological complete response cases (n=8)	p-value
1	diagnostics_Image-original_Mean_py	0.64(5.45)	0.73(5.58)	0.06(4.86)	0.749
2	diagnostics_Mask-original_VoxelNum_py	15561.58(11615.29)	16319.39(12154.58)	10730.50(5636.32)	0.26
3	diagnostics_Mask-original_VolumeNum_py	1.07(0.25)	1.06(0.24)	1.12(0.35)	0.509
4	original_shape2D_Elongation_py	0.55(0.17)	0.56(0.18)	0.52(0.14)	0.58
5	original_shape2D_MajorAxisLength_py	229.64(90.13)	236.43(92.71)	186.38(58.60)	0.146
6	original_shape2D_MaximumDiameter_py	227.62(90.97)	233.42(93.40)	190.69(66.83)	0.22
7	original_shape2D_MeshSurface_py	15560.94(11615.28)	16318.83(12154.62)	10729.38(5634.98)	0.26
8	original_shape2D_MinorAxisLength_py	122.47(55.01)	126.86(57.08)	94.49(27.68)	0.123
9	original_shape2D_Perimeter_py	814.20(411.19)	844.72(416.05)	619.67(338.39)	0.152
10	original_shape2D_PerimeterSurfaceRatio_py	0.06(0.02)	0.06(0.02)	0.06(0.03)	0.929
11	original_shape2D_PixelSurface_py	15561.58(11615.29)	16319.39(12154.58)	10730.50(5636.32)	0.26
12	original_shape2D_Sphericity_py	0.55(0.12)	0.54(0.11)	0.62(0.14)	0.07
13	original_firstorder_10Percentile_py	-113.27(33.56)	-112.14(36.00)	-120.50(1.77)	0.403
14	original_firstorder_90Percentile_py	95.80(56.89)	93.12(60.47)	112.91(17.60)	0.563
15	original_firstorder_Energy_py	164263308.19(120728552.44)	170648872.65(126153197.34)	123555334.75(70004199.71)	0.535
16	original_firstorder_Entropy_py	2.62(0.35)	2.62(0.36)	2.59(0.32)	0.844
17	original_firstorder_InterquartileRange_py	126.50(88.28)	126.31(88.05)	127.66(95.88)	0.868
18	original_firstorder_Kurtosis_py	3.68(3.74)	3.82(3.93)	2.76(2.10)	0.493
19	original_firstorder_MeanAbsoluteDeviation_py	75.07(25.22)	74.11(25.67)	81.21(22.62)	0.464
20	original_firstorder_Mean_py	-16.87(47.73)	-18.51(47.50)	-6.40(51.10)	0.509
21	original_firstorder_Median_py	-24.95(87.35)	-29.10(85.80)	1.50(98.54)	0.595
22	original_firstorder_RobustMeanAbsoluteDeviation_py	59.62(31.17)	58.72(31.20)	65.35(32.45)	0.581
23	original_firstorder_RootMeanSquared_py	103.02(7.93)	102.56(8.32)	106.00(3.77)	0.065
24	original_firstorder_Skewness_py	0.53(1.43)	0.57(1.45)	0.23(1.33)	0.53
25	original_firstorder_TotalEnergy_py	164263308.19(120728552.44)	170648872.65(126153197.34)	123555334.75(70004199.71)	0.535
26	original_firstorder_Uniformity_py	0.20(0.06)	0.20(0.06)	0.21(0.06)	0.72
27	original_firstorder_Variance_py	8151.89(2940.28)	8030.86(3010.94)	8923.45(2467.65)	0.429

28	original_glcm_Autocorrelation_py	65.64(37.33)	64.39(37.30)	73.59(39.09)	0.522
29	original_glcm_ClusterProminence_py	13280.77(3861.26)	13164.39(4058.75)	14022.63(2267.04)	0.563
30	original_glcm_ClusterShade_py	138.44(596.32)	161.10(590.05)	-6.01(657.23)	0.609
31	original_glcm_ClusterTendency_py	72.62(27.15)	71.80(28.03)	77.82(21.38)	0.564
32	original_glcm_Contrast_py*	9.69(3.96)	9.27(3.70)	12.40(4.78)	0.037
33	original_glcm_Correlation_py	0.76(0.06)	0.76(0.06)	0.73(0.04)	0.107
34	original_glcm_DifferenceAverage_py*	0.98(0.35)	0.95(0.32)	1.24(0.42)	0.027
35	original_glcm_DifferenceEntropy_py*	1.14(0.21)	1.12(0.20)	1.29(0.20)	0.033
36	original_glcm_DifferenceVariance_py*	8.60(3.26)	8.27(3.10)	10.72(3.69)	0.048
37	original_glcm_Id_py*	0.83(0.05)	0.83(0.05)	0.79(0.05)	0.03
38	original_glcm_Idm_py*	0.82(0.05)	0.83(0.05)	0.79(0.05)	0.03
39	original_glcm_Idmn_py*	0.97(0.01)	0.97(0.01)	0.96(0.01)	0.033
40	original_glcm_Idn_py*	0.96(0.01)	0.96(0.01)	0.95(0.02)	0.025
41	original_glcm_Imc1_py*	-0.55(0.07)	-0.56(0.07)	-0.49(0.05)	0.011
42	original_glcm_Imc2_py	0.97(0.02)	0.97(0.02)	0.96(0.02)	0.142
43	original_glcm_InverseVariance_py	0.24(0.06)	0.23(0.06)	0.27(0.04)	0.077
44	original_glcm_JointAverage_py	6.65(2.40)	6.57(2.39)	7.17(2.58)	0.515
45	original_glcm_JointEnergy_py	0.13(0.05)	0.13(0.05)	0.12(0.05)	0.669
46	original_glcm_JointEntropy_py	3.77(0.52)	3.76(0.52)	3.89(0.51)	0.5
47	original_glcm_MCC_py	0.94(0.03)	0.94(0.03)	0.92(0.02)	0.117
48	original_glcm_MaximumProbability_py	0.25(0.09)	0.25(0.09)	0.25(0.08)	0.939
49	original_glcm_SumAverage_py	13.30(4.81)	13.14(4.78)	14.34(5.15)	0.515
50	original_glcm_SumEntropy_py	3.44(0.43)	3.44(0.44)	3.50(0.42)	0.685
51	original_glcm_SumSquares_py	20.58(7.56)	20.27(7.73)	22.55(6.40)	0.431
52	original_gldm_DependenceEntropy_py	3.86(0.44)	3.86(0.44)	3.91(0.41)	0.754
53	original_gldm_DependenceNonUniformity_py	6956.39(6043.92)	7370.38(6345.55)	4317.18(2417.68)	0.186
54	original_gldm_DependenceNonUniformityNormalized_py	0.43(0.05)	0.43(0.05)	0.40(0.03)	0.057
55	original_gldm_DependenceVariance_py	0.43(0.07)	0.42(0.07)	0.47(0.05)	0.053
56	original_gldm_GrayLevelNonUniformity_py	3210.87(2743.03)	3350.78(2867.54)	2318.96(1599.23)	0.327
57	original_gldm_GrayLevelVariance_py	20.72(7.44)	20.41(7.62)	22.65(6.21)	0.433
58	original_gldm_HighGrayLevelEmphasis_py	70.95(37.92)	69.49(37.77)	80.27(40.10)	0.46
59	original_gldm_LargeDependenceEmphasis_py*	6.13(0.66)	6.20(0.65)	5.69(0.59)	0.046
60	original_gldm_LargeDependenceHighGrayLevelEmphasis_py	417.44(257.61)	413.85(259.25)	440.34(262.87)	0.789

61	original_gldm_LargeDependenceLowGrayLevelEmphasis_py	0.98(0.32)	0.99(0.33)	0.90(0.26)	0.489
62	original_gldm_LowGrayLevelEmphasis_py	0.18(0.05)	0.18(0.05)	0.18(0.03)	0.839
63	original_gldm_SmallDependenceEmphasis_py*	0.26(0.06)	0.26(0.05)	0.30(0.05)	0.046
64	original_gldm_SmallDependenceHighGrayLevelEmphasis_py	20.26(9.31)	19.40(8.97)	25.76(10.22)	0.072
65	original_gldm_SmallDependenceLowGrayLevelEmphasis_py	0.06(0.02)	0.06(0.02)	0.07(0.02)	0.07
66	original_glrlm_GrayLevelNonUniformity_py	762.30(545.76)	778.50(568.84)	659.06(377.62)	0.569
67	original_glrlm_GrayLevelNonUniformityNormalized_py	0.17(0.04)	0.17(0.04)	0.17(0.03)	0.994
68	original_glrlm_GrayLevelVariance_py	25.16(6.42)	24.84(6.71)	27.15(3.84)	0.349
69	original_glrlm_HighGrayLevelRunEmphasis_py	76.65(31.04)	75.36(31.25)	84.85(30.30)	0.426
70	original_glrlm_LongRunEmphasis_py	24.38(15.72)	25.77(16.28)	15.52(7.01)	0.086
71	original_glrlm_LongRunHighGrayLevelEmphasis_py	1530.47(1425.78)	1593.70(1484.48)	1127.34(942.47)	0.493
72	original_glrlm_LongRunLowGrayLevelEmphasis_py	3.53(2.69)	3.72(2.79)	2.31(1.60)	0.06
73	original_glrlm_LowGrayLevelRunEmphasis_py	0.23(0.05)	0.23(0.06)	0.23(0.03)	0.973
74	original_glrlm_RunEntropy_py*	5.50(0.40)	5.54(0.40)	5.20(0.24)	0.025
75	original_glrlm_RunLengthNonUniformity_py	848.09(550.23)	843.15(568.10)	879.64(449.38)	0.863
76	original_glrlm_RunLengthNonUniformityNormalized_py	0.20(0.06)	0.19(0.06)	0.23(0.06)	0.053
77	original_glrlm_RunPercentage_py*	0.31(0.08)	0.30(0.08)	0.36(0.07)	0.046
78	original_glrlm_RunVariance_py	11.48(8.13)	12.20(8.43)	6.89(3.40)	0.085
79	original_glrlm_ShortRunEmphasis_py	0.40(0.10)	0.39(0.10)	0.46(0.08)	0.052
80	original_glrlm_ShortRunHighGrayLevelEmphasis_py*	34.33(12.01)	33.01(11.67)	42.77(11.33)	0.031
81	original_glrlm_ShortRunLowGrayLevelEmphasis_py	0.12(0.03)	0.12(0.03)	0.13(0.02)	0.102
82	original_glszm_GrayLevelNonUniformity_py	762.30(545.76)	778.50(568.84)	659.06(377.62)	0.569
83	original_glszm_GrayLevelNonUniformityNormalized_py	0.17(0.04)	0.17(0.04)	0.17(0.03)	0.994
84	original_glszm_GrayLevelVariance_py	25.16(6.42)	24.84(6.71)	27.15(3.84)	0.349
85	original_glszm_HighGrayLevelZoneEmphasis_py	76.65(31.04)	75.36(31.25)	84.85(30.30)	0.426

86	original_glszm_LargeAreaEmphasis_py	24.38(15.72)	25.77(16.28)	15.52(7.01)	0.086
87	original_glszm_LargeAreaHighGrayLevelEmphasis_py	1530.47(1425.78)	1593.70(1484.48)	1127.34(942.47)	0.493
88	original_glszm_LargeAreaLowGrayLevelEmphasis_py	3.53(2.69)	3.72(2.79)	2.31(1.60)	0.06
89	original_glszm_LowGrayLevelZoneEmphasis_py	0.23(0.05)	0.23(0.06)	0.23(0.03)	0.973
90	original_glszm_SizeZoneNonUniformity_py	848.09(550.23)	843.15(568.10)	879.64(449.38)	0.863
91	original_glszm_SizeZoneNonUniformityNormalized_py	0.20(0.06)	0.19(0.06)	0.23(0.06)	0.053
92	original_glszm_SmallAreaEmphasis_py	0.40(0.10)	0.39(0.10)	0.46(0.08)	0.052
93	original_glszm_SmallAreaHighGrayLevelEmphasis_py*	34.33(12.01)	33.01(11.67)	42.77(11.33)	0.031
94	original_glszm_SmallAreaLowGrayLevelEmphasis_py	0.12(0.03)	0.12(0.03)	0.13(0.02)	0.102
95	original_glszm_ZoneEntropy_py*	5.50(0.40)	5.54(0.40)	5.20(0.24)	0.025
96	original_glszm_ZonePercentage_py*	0.31(0.08)	0.30(0.08)	0.36(0.07)	0.046
97	original_glszm_ZoneVariance_py	11.48(8.13)	12.20(8.43)	6.89(3.40)	0.085
98	original_ngtdm_Busyness_py	20.46(14.41)	20.87(15.11)	17.84(8.92)	0.584
99	original_ngtdm_Coarseness_py	0.00(0.00)	0.00(0.00)	0.00(0.00)	0.656
100	original_ngtdm_Complexity_py*	93.37(39.39)	89.33(36.48)	119.10(49.80)	0.046
101	original_ngtdm_Contrast_py	0.41(0.30)	0.39(0.29)	0.53(0.36)	0.209
102	original_ngtdm_Strength_py	0.13(0.16)	0.13(0.17)	0.14(0.13)	0.939
103	log-sigma-1-mm-3D_firstorder_10Percentile_py	-34.29(15.41)	-33.29(15.48)	-40.66(14.25)	0.163
104	log-sigma-1-mm-3D_firstorder_90Percentile_py	37.65(14.14)	36.32(14.35)	46.15(9.40)	0.071
105	log-sigma-1-mm-3D_firstorder_Energy_py	14059322.33(11055516.35)	14132306.90(11502436.35)	13594045.72(8208984.64)	0.899
106	log-sigma-1-mm-3D_firstorder_Entropy_py	2.48(0.39)	2.45(0.39)	2.69(0.34)	0.096
107	log-sigma-1-mm-3D_firstorder_InterquartileRange_py*	17.62(12.69)	16.15(11.26)	26.99(17.66)	0.023
108	log-sigma-1-mm-3D_firstorder_Kurtosis_py	6.67(3.92)	6.95(4.11)	4.83(1.52)	0.1
109	log-sigma-1-mm-3D_firstorder_Maximum_py	151.30(21.66)	150.87(20.93)	154.02(27.37)	0.705
110	log-sigma-1-mm-3D_firstorder_MeanAbsoluteDeviation_py	19.27(6.32)	18.65(6.10)	23.27(6.65)	0.054

111	log-sigma-1-mm-3D_firstorder_Mean_py	0.67(0.77)	0.69(0.80)	0.51(0.62)	0.507
112	log-sigma-1-mm-3D_firstorder_Median_py	0.02(0.67)	0.04(0.63)	-0.14(0.90)	0.474
113	log-sigma-1-mm-3D_firstorder_Minimum_py	-154.60(17.79)	-155.76(18.01)	-147.23(15.37)	0.211
114	log-sigma-1-mm-3D_firstorder_Range_py	305.90(29.85)	306.63(28.95)	301.26(37.00)	0.64
115	log-sigma-1-mm-3D_firstorder_RobustMeanAbsoluteDeviation_py*	9.60(5.13)	9.08(4.82)	12.93(6.14)	0.048
116	log-sigma-1-mm-3D_firstorder_RootMeanSquared_py	30.57(6.50)	29.96(6.38)	34.44(6.26)	0.069
117	log-sigma-1-mm-3D_firstorder_Skewness_py	-0.09(0.67)	-0.11(0.69)	-0.01(0.58)	0.696
118	log-sigma-1-mm-3D_firstorder_TotalEnergy_py	14059322.33(11055516.35)	14132306.90(11502436.35)	13594045.72(8208984.64)	0.899
119	log-sigma-1-mm-3D_firstorder_Uniformity_py	0.25(0.07)	0.26(0.07)	0.21(0.06)	0.098
120	log-sigma-1-mm-3D_firstorder_Variance_py	974.90(385.43)	936.46(366.30)	1219.97(439.41)	0.052
121	log-sigma-1-mm-3D_glcm_Autocorrelation_py	78.33(14.75)	79.19(15.18)	72.89(10.88)	0.414
122	log-sigma-1-mm-3D_glcm_ClusterProminence_py	374.96(172.50)	359.04(162.24)	476.46(211.98)	0.073
123	log-sigma-1-mm-3D_glcm_ClusterShade_py	0.69(12.31)	0.33(11.84)	3.01(15.74)	0.572
124	log-sigma-1-mm-3D_glcm_ClusterTendency_py	8.43(3.09)	8.13(2.95)	10.32(3.50)	0.062
125	log-sigma-1-mm-3D_glcm_Contrast_py*	1.87(0.74)	1.79(0.68)	2.40(0.94)	0.029
126	log-sigma-1-mm-3D_glcm_Correlation_py	0.64(0.04)	0.64(0.04)	0.63(0.04)	0.328
127	log-sigma-1-mm-3D_glcm_DifferenceAverage_py*	0.72(0.22)	0.69(0.21)	0.86(0.25)	0.041
128	log-sigma-1-mm-3D_glcm_DifferenceEntropy_py	1.61(0.28)	1.58(0.28)	1.78(0.27)	0.063
129	log-sigma-1-mm-3D_glcm_DifferenceVariance_py*	1.31(0.42)	1.26(0.40)	1.60(0.48)	0.035
130	log-sigma-1-mm-3D_glcm_Id_py	0.75(0.06)	0.76(0.06)	0.71(0.06)	0.053
131	log-sigma-1-mm-3D_glcm_Idm_py	0.74(0.07)	0.74(0.07)	0.69(0.07)	0.053
132	log-sigma-1-mm-3D_glcm_Idmn_py*	0.99(0.00)	0.99(0.00)	0.99(0.00)	0.014
133	log-sigma-1-mm-3D_glcm_Idn_py*	0.96(0.01)	0.96(0.01)	0.95(0.01)	0.024
134	log-sigma-1-mm-3D_glcm_Imc1_py	-0.31(0.05)	-0.32(0.05)	-0.28(0.05)	0.067
135	log-sigma-1-mm-3D_glcm_Imc2_py	0.88(0.02)	0.88(0.02)	0.88(0.02)	0.751
136	log-sigma-1-mm-3D_glcm_InverseVariance_py	0.32(0.05)	0.31(0.05)	0.35(0.04)	0.076
137	log-sigma-1-mm-3D_glcm_JointAverage_py	8.71(0.88)	8.76(0.90)	8.40(0.64)	0.137
138	log-sigma-1-mm-3D_glcm_JointEnergy_py	0.14(0.06)	0.14(0.06)	0.10(0.05)	0.084
139	log-sigma-1-mm-3D_glcm_JointEntropy_py	4.19(0.76)	4.12(0.75)	4.64(0.73)	0.074

140	log-sigma-1-mm-3D_glcm_MCC_py	0.76(0.05)	0.76(0.04)	0.73(0.07)	0.116
141	log-sigma-1-mm-3D_glcm_MaximumProbability_py*	0.26(0.07)	0.26(0.06)	0.21(0.07)	0.047
142	log-sigma-1-mm-3D_glcm_SumAverage_py	17.43(1.75)	17.53(1.80)	16.80(1.27)	0.137
143	log-sigma-1-mm-3D_glcm_SumEntropy_py	3.19(0.46)	3.15(0.46)	3.45(0.40)	0.091
144	log-sigma-1-mm-3D_glcm_SumSquares_py	2.57(0.95)	2.48(0.90)	3.18(1.10)	0.052
145	log-sigma-1-mm-3D_gldm_DependenceEntropy_py	3.77(0.46)	3.73(0.46)	4.02(0.37)	0.093
146	log-sigma-1-mm-3D_gldm_DependenceNonUniformity_py	5823.49(5002.28)	6142.67(5269.16)	3788.70(1882.27)	0.219
147	log-sigma-1-mm-3D_gldm_DependenceNonUniformityNormalized_py	0.36(0.04)	0.36(0.04)	0.36(0.02)	0.837
148	log-sigma-1-mm-3D_gldm_DependenceVariance_py	0.57(0.06)	0.57(0.06)	0.57(0.03)	0.401
149	log-sigma-1-mm-3D_gldm_GrayLevelNonUniformity_py	4181.31(4192.57)	4486.28(4411.53)	2237.16(1278.33)	0.16
150	log-sigma-1-mm-3D_gldm_GrayLevelVariance_py	2.59(0.94)	2.49(0.89)	3.18(1.08)	0.053
151	log-sigma-1-mm-3D_gldm_HighGrayLevelEmphasis_py	79.27(14.81)	80.09(15.25)	74.05(11.00)	0.452
152	log-sigma-1-mm-3D_gldm_LargeDependenceEmphasis_py	5.10(0.79)	5.18(0.78)	4.61(0.75)	0.057
153	log-sigma-1-mm-3D_gldm_LargeDependenceHighGrayLevelEmphasis_py*	395.32(94.70)	405.19(94.95)	332.36(68.35)	0.042
154	log-sigma-1-mm-3D_gldm_LargeDependenceLowGrayLevelEmphasis_py	0.08(0.02)	0.08(0.02)	0.07(0.02)	0.827
155	log-sigma-1-mm-3D_gldm_LowGrayLevelEmphasis_py*	0.02(0.00)	0.02(0.00)	0.02(0.00)	0.044
156	log-sigma-1-mm-3D_gldm_SmallDependenceEmphasis_py	0.39(0.08)	0.38(0.08)	0.43(0.08)	0.074
157	log-sigma-1-mm-3D_gldm_SmallDependenceHighGrayLevelEmphasis_py	31.55(9.45)	31.31(9.62)	33.05(8.68)	0.632

158	log-sigma-1-mm-3D_gldm_SmallDependenceLowGrayLevelEmphasis_py*	0.01(0.00)	0.01(0.00)	0.01(0.00)	0.023
159	log-sigma-1-mm-3D_glrlm_GrayLevelNonUniformity_py	1024.99(736.36)	1067.65(770.03)	753.02(399.53)	0.265
160	log-sigma-1-mm-3D_glrlm_GrayLevelNonUniformityNormalized_py	0.16(0.04)	0.16(0.04)	0.14(0.02)	0.26
161	log-sigma-1-mm-3D_glrlm_GrayLevelVariance_py	4.22(0.91)	4.14(0.90)	4.71(0.94)	0.106
162	log-sigma-1-mm-3D_glrlm_HighGrayLevelRunEmphasis_py	81.51(14.63)	82.38(15.06)	75.95(10.70)	0.241
163	log-sigma-1-mm-3D_glrlm_LongRunEmphasis_py	12.50(8.22)	13.19(8.55)	8.14(3.52)	0.107
164	log-sigma-1-mm-3D_glrlm_LongRunHighGrayLevelEmphasis_py	962.65(699.96)	1022.66(729.15)	580.12(261.77)	0.097
165	log-sigma-1-mm-3D_glrlm_LongRunLowGrayLevelEmphasis_py	0.18(0.12)	0.19(0.12)	0.13(0.05)	0.241
166	log-sigma-1-mm-3D_glrlm_LowGrayLevelRunEmphasis_py*	0.02(0.01)	0.02(0.01)	0.02(0.00)	0.035
167	log-sigma-1-mm-3D_glrlm_RunEntropy_py	4.94(0.19)	4.95(0.19)	4.84(0.14)	0.129
168	log-sigma-1-mm-3D_glrlm_RunLengthNonUniformity_py	2293.67(1770.05)	2302.86(1841.47)	2235.09(1316.75)	0.888
169	log-sigma-1-mm-3D_glrlm_RunLengthNonUniformityNormalized_py	0.36(0.09)	0.35(0.09)	0.40(0.09)	0.146
170	log-sigma-1-mm-3D_glrlm_RunPercentage_py	0.44(0.10)	0.43(0.09)	0.50(0.09)	0.061
171	log-sigma-1-mm-3D_glrlm_RunVariance_py	6.30(4.57)	6.70(4.75)	3.72(1.79)	0.086
172	log-sigma-1-mm-3D_glrlm_ShortRunEmphasis_py	0.61(0.09)	0.60(0.09)	0.65(0.08)	0.224
173	log-sigma-1-mm-3D_glrlm_ShortRunHighGrayLevelEmphasis_py	50.67(12.08)	50.75(12.49)	50.20(9.71)	0.906
174	log-sigma-1-mm-3D_glrlm_ShortRunLowGrayLevelEmphasis_py*	0.01(0.00)	0.01(0.00)	0.01(0.00)	0.029
175	log-sigma-1-mm-3D_glszm_GrayLevelNonUniformity_py	1024.99(736.36)	1067.65(770.03)	753.02(399.53)	0.265

176	log-sigma-1-mm-3D_glszm_GrayLevelNonUniformityNormalized_py	0.16(0.04)	0.16(0.04)	0.14(0.02)	0.26
177	log-sigma-1-mm-3D_glszm_GrayLevelVariance_py	4.22(0.91)	4.14(0.90)	4.71(0.94)	0.106
178	log-sigma-1-mm-3D_glszm_HighGrayLevelZoneEmphasis_py	81.51(14.63)	82.38(15.06)	75.95(10.70)	0.241
179	log-sigma-1-mm-3D_glszm_LargeAreaEmphasis_py	12.50(8.22)	13.19(8.55)	8.14(3.52)	0.107
180	log-sigma-1-mm-3D_glszm_LargeAreaHighGrayLevelEmphasis_py	962.65(699.96)	1022.66(729.15)	580.12(261.77)	0.097
181	log-sigma-1-mm-3D_glszm_LargeAreaLowGrayLevelEmphasis_py	0.18(0.12)	0.19(0.12)	0.13(0.05)	0.241
182	log-sigma-1-mm-3D_glszm_LowGrayLevelZoneEmphasis_py*	0.02(0.01)	0.02(0.01)	0.02(0.00)	0.035
183	log-sigma-1-mm-3D_glszm_SizeZoneNonUniformity_py	2293.67(1770.05)	2302.86(1841.47)	2235.09(1316.75)	0.888
184	log-sigma-1-mm-3D_glszm_SizeZoneNonUniformityNormalized_py	0.36(0.09)	0.35(0.09)	0.40(0.09)	0.146
185	log-sigma-1-mm-3D_glszm_SmallAreaEmphasis_py	0.61(0.09)	0.60(0.09)	0.65(0.08)	0.224
186	log-sigma-1-mm-3D_glszm_SmallAreaHighGrayLevelEmphasis_py	50.67(12.08)	50.75(12.49)	50.20(9.71)	0.906
187	log-sigma-1-mm-3D_glszm_SmallAreaLowGrayLevelEmphasis_py*	0.01(0.00)	0.01(0.00)	0.01(0.00)	0.029
188	log-sigma-1-mm-3D_glszm_ZoneEntropy_py	4.94(0.19)	4.95(0.19)	4.84(0.14)	0.129
189	log-sigma-1-mm-3D_glszm_ZonePercentage_py	0.44(0.10)	0.43(0.09)	0.50(0.09)	0.061
190	log-sigma-1-mm-3D_glszm_ZoneVariance_py	6.30(4.57)	6.70(4.75)	3.72(1.79)	0.086
191	log-sigma-1-mm-3D_ngtdm_Busyness_py	5.72(3.69)	5.87(3.82)	4.81(2.71)	0.455
192	log-sigma-1-mm-3D_ngtdm_Coarseness_py	0.00(0.00)	0.00(0.00)	0.00(0.00)	0.377
193	log-sigma-1-mm-3D_ngtdm_Complexity_py	63.52(22.83)	62.03(22.11)	72.99(26.60)	0.209
194	log-sigma-1-mm-3D_ngtdm_Contrast_py*	0.01(0.01)	0.01(0.01)	0.02(0.01)	0.016
195	log-sigma-1-mm-3D_ngtdm_Strength_py	0.15(0.11)	0.15(0.11)	0.15(0.08)	0.966
196	log-sigma-2-mm-3D_firstorder_10Percentile_py	-39.14(14.59)	-38.22(14.71)	-45.05(13.12)	0.221

197	log-sigma-2-mm-3D_firstorder_90Percentile_py	45.99(12.87)	44.99(13.05)	52.41(10.16)	0.131
198	log-sigma-2-mm-3D_firstorder_Energy_py	19083959.70(15577551.41)	19414417.16(16306516.96)	16977293.45(10268452.73)	0.684
199	log-sigma-2-mm-3D_firstorder_Entropy_py	2.72(0.39)	2.69(0.40)	2.88(0.29)	0.192
200	log-sigma-2-mm-3D_firstorder_InterquartileRange_py	33.57(15.54)	32.48(15.14)	40.55(17.26)	0.174
201	log-sigma-2-mm-3D_firstorder_Kurtosis_py	5.22(2.85)	5.39(2.98)	4.11(1.48)	0.199
202	log-sigma-2-mm-3D_firstorder_Maximum_py	142.98(17.91)	143.51(18.18)	139.57(16.84)	0.567
203	log-sigma-2-mm-3D_firstorder_MeanAbsoluteDeviation_py	25.07(7.10)	24.51(7.07)	28.61(6.61)	0.131
204	log-sigma-2-mm-3D_firstorder_Mean_py	2.05(2.08)	2.12(2.13)	1.61(1.82)	0.523
205	log-sigma-2-mm-3D_firstorder_Median_py	1.12(4.00)	1.32(3.83)	-0.11(5.07)	0.353
206	log-sigma-2-mm-3D_firstorder_Minimum_py	-145.60(15.84)	-146.50(16.59)	-139.82(8.31)	0.271
207	log-sigma-2-mm-3D_firstorder_Range_py	288.57(25.84)	290.02(27.18)	279.39(11.99)	0.283
208	log-sigma-2-mm-3D_firstorder_RobustMeanAbsoluteDeviation_py	15.05(6.00)	14.58(5.89)	18.05(6.26)	0.129
209	log-sigma-2-mm-3D_firstorder_RootMeanSquared_py	35.25(7.19)	34.71(7.27)	38.68(5.92)	0.149
210	log-sigma-2-mm-3D_firstorder_Skewness_py	-0.11(0.68)	-0.13(0.68)	-0.03(0.69)	0.714
211	log-sigma-2-mm-3D_firstorder_TotalEnergy_py	19083959.70(15577551.41)	19414417.16(16306516.96)	16977293.45(10268452.73)	0.684
212	log-sigma-2-mm-3D_firstorder_Uniformity_py	0.21(0.07)	0.21(0.07)	0.18(0.05)	0.26
213	log-sigma-2-mm-3D_firstorder_Variance_py	1284.88(485.09)	1247.82(483.84)	1521.15(451.49)	0.14
214	log-sigma-2-mm-3D_glcm_Autocorrelation_py	73.28(13.47)	73.94(13.97)	69.08(9.33)	0.439
215	log-sigma-2-mm-3D_glcm_ClusterProminence_py	664.83(298.90)	645.16(303.51)	790.19(248.01)	0.205
216	log-sigma-2-mm-3D_glcm_ClusterShade_py	0.03(24.45)	-0.66(23.15)	4.48(33.15)	0.939
217	log-sigma-2-mm-3D_glcm_ClusterTendency_py	12.36(4.58)	12.02(4.58)	14.53(4.22)	0.151
218	log-sigma-2-mm-3D_glcm_Contrast_py*	0.86(0.32)	0.82(0.30)	1.06(0.36)	0.044
219	log-sigma-2-mm-3D_glcm_Correlation_py	0.87(0.02)	0.87(0.02)	0.86(0.02)	0.523
220	log-sigma-2-mm-3D_glcm_DifferenceAverage_py	0.54(0.16)	0.52(0.15)	0.64(0.17)	0.056
221	log-sigma-2-mm-3D_glcm_DifferenceEntropy_py	1.36(0.22)	1.34(0.22)	1.50(0.20)	0.071
222	log-sigma-2-mm-3D_glcm_DifferenceVariance_py	0.54(0.15)	0.53(0.14)	0.63(0.15)	0.056

223	log-sigma-2-mm-3D_glcm_Id_py	0.78(0.06)	0.78(0.06)	0.74(0.06)	0.07
224	log-sigma-2-mm-3D_glcm_Idm_py	0.76(0.06)	0.77(0.06)	0.72(0.06)	0.066
225	log-sigma-2-mm-3D_glcm_Idmn_py*	1.00(0.00)	1.00(0.00)	1.00(0.00)	0.034
226	log-sigma-2-mm-3D_glcm_Idn_py*	0.97(0.01)	0.97(0.01)	0.96(0.01)	0.045
227	log-sigma-2-mm-3D_glcm_Imc1_py*	-0.40(0.05)	-0.40(0.05)	-0.36(0.05)	0.048
228	log-sigma-2-mm-3D_glcm_Imc2_py	0.94(0.02)	0.94(0.02)	0.93(0.01)	0.765
229	log-sigma-2-mm-3D_glcm_InverseVariance_py	0.32(0.06)	0.32(0.07)	0.35(0.05)	0.15
230	log-sigma-2-mm-3D_glcm_JointAverage_py	8.35(0.83)	8.39(0.86)	8.09(0.59)	0.241
231	log-sigma-2-mm-3D_glcm_JointEnergy_py	0.11(0.07)	0.12(0.07)	0.08(0.05)	0.177
232	log-sigma-2-mm-3D_glcm_JointEntropy_py	4.36(0.73)	4.30(0.73)	4.72(0.61)	0.134
233	log-sigma-2-mm-3D_glcm_MCC_py	0.87(0.02)	0.87(0.02)	0.87(0.02)	0.391
234	log-sigma-2-mm-3D_glcm_MaximumProbability_py	0.23(0.08)	0.24(0.08)	0.20(0.08)	0.2
235	log-sigma-2-mm-3D_glcm_SumAverage_py	16.70(1.66)	16.78(1.72)	16.17(1.19)	0.241
236	log-sigma-2-mm-3D_glcm_SumEntropy_py	3.52(0.49)	3.49(0.50)	3.73(0.36)	0.184
237	log-sigma-2-mm-3D_glcm_SumSquares_py	3.30(1.22)	3.21(1.21)	3.90(1.13)	0.139
238	log-sigma-2-mm-3D_gldm_DependenceEntropy_py	4.11(0.51)	4.07(0.53)	4.32(0.37)	0.207
239	log-sigma-2-mm-3D_gldm_DependenceNonUniformity_py	5965.87(5317.47)	6312.98(5603.40)	3753.09(1880.56)	0.208
240	log-sigma-2-mm-3D_gldm_DependenceNonUniformityNormalized_py	0.37(0.05)	0.37(0.05)	0.35(0.02)	0.426
241	log-sigma-2-mm-3D_gldm_DependenceVariance_py	0.57(0.06)	0.56(0.07)	0.59(0.04)	0.439
242	log-sigma-2-mm-3D_gldm_GrayLevelNonUniformity_py	3505.84(3733.95)	3752.76(3939.27)	1931.68(1185.07)	0.156
243	log-sigma-2-mm-3D_gldm_GrayLevelVariance_py	3.31(1.20)	3.22(1.20)	3.90(1.11)	0.141
244	log-sigma-2-mm-3D_gldm_HighGrayLevelEmphasis_py	73.67(13.46)	74.32(13.96)	69.52(9.26)	0.452
245	log-sigma-2-mm-3D_gldm_LargeDependenceEmphasis_py	5.30(0.82)	5.37(0.81)	4.84(0.72)	0.09
246	log-sigma-2-mm-3D_gldm_LargeDependenceHighGrayLevelEmphasis_py	386.70(107.93)	395.44(109.77)	331.01(79.69)	0.117

**247**	log-sigma-2-mm-3D_gldm_LargeDependenceLowGrayLevelEmphasis_py	0.09(0.03)	0.09(0.03)	0.09(0.02)	0.974
**248**	log-sigma-2-mm-3D_gldm_LowGrayLevelEmphasis_py	0.02(0.01)	0.02(0.01)	0.02(0.00)	0.354
**249**	log-sigma-2-mm-3D_gldm_SmallDependenceEmphasis_py	0.37(0.08)	0.36(0.08)	0.41(0.08)	0.082
**250**	log-sigma-2-mm-3D_gldm_SmallDependenceHighGrayLevelEmphasis_py	27.43(6.74)	27.15(6.94)	29.25(5.28)	0.418
**251**	log-sigma-2-mm-3D_gldm_SmallDependenceLowGrayLevelEmphasis_py	0.01(0.00)	0.01(0.00)	0.01(0.00)	0.091
**252**	log-sigma-2-mm-3D_glrlm_GrayLevelNonUniformity_py	864.58(579.78)	897.07(605.63)	657.46(329.49)	0.281
**253**	log-sigma-2-mm-3D_glrlm_GrayLevelNonUniformityNormalized_py	0.15(0.03)	0.15(0.03)	0.13(0.02)	0.32
**254**	log-sigma-2-mm-3D_glrlm_GrayLevelVariance_py	4.63(1.02)	4.57(1.04)	4.98(0.81)	0.299
**255**	log-sigma-2-mm-3D_glrlm_HighGrayLevelRunEmphasis_py	75.35(12.93)	75.99(13.46)	71.29(8.25)	0.343
**256**	log-sigma-2-mm-3D_glrlm_LongRunEmphasis_py	15.70(11.69)	16.55(12.22)	10.31(5.25)	0.105
**257**	log-sigma-2-mm-3D_glrlm_LongRunHighGrayLevelEmphasis_py	1164.77(1046.84)	1237.99(1099.21)	698.00(401.90)	0.115
**258**	log-sigma-2-mm-3D_glrlm_LongRunLowGrayLevelEmphasis_py	0.25(0.16)	0.26(0.16)	0.19(0.08)	0.207
**259**	log-sigma-2-mm-3D_glrlm_LowGrayLevelRunEmphasis_py	0.02(0.01)	0.02(0.01)	0.02(0.00)	0.416
**260**	log-sigma-2-mm-3D_glrlm_RunEntropy_py	5.22(0.26)	5.24(0.27)	5.07(0.19)	0.088
**261**	log-sigma-2-mm-3D_glrlm_RunLengthNonUniformity_py	2127.88(1757.35)	2138.55(1839.77)	2059.86(1185.55)	0.786
**262**	log-sigma-2-mm-3D_glrlm_RunLengthNonUniformityNormalized_py	0.34(0.09)	0.34(0.09)	0.39(0.08)	0.096

263	log-sigma-2-mm-3D_glrlm_RunPercentage_py	0.42(0.10)	0.41(0.10)	0.47(0.09)	0.087
264	log-sigma-2-mm-3D_glrlm_RunVariance_py	8.51(6.84)	9.01(7.14)	5.33(3.08)	0.1
265	log-sigma-2-mm-3D_glrlm_ShortRunEmphasis_py	0.60(0.09)	0.59(0.09)	0.64(0.07)	0.115
266	log-sigma-2-mm-3D_glrlm_ShortRunHighGrayLevelEmphasis_py	45.17(9.45)	45.03(9.85)	46.09(6.73)	0.772
267	log-sigma-2-mm-3D_glrlm_ShortRunLowGrayLevelEmphasis_py	0.01(0.00)	0.01(0.00)	0.01(0.00)	0.21
268	log-sigma-2-mm-3D_glszm_GrayLevelNonUniformity_py	864.58(579.78)	897.07(605.63)	657.46(329.49)	0.281
269	log-sigma-2-mm-3D_glszm_GrayLevelNonUniformityNormalized_py	0.15(0.03)	0.15(0.03)	0.13(0.02)	0.32
270	log-sigma-2-mm-3D_glszm_GrayLevelVariance_py	4.63(1.02)	4.57(1.04)	4.98(0.81)	0.299
271	log-sigma-2-mm-3D_glszm_HighGrayLevelZoneEmphasis_py	75.35(12.93)	75.99(13.46)	71.29(8.25)	0.343
272	log-sigma-2-mm-3D_glszm_LargeAreaEmphasis_py	15.70(11.69)	16.55(12.22)	10.31(5.25)	0.105
273	log-sigma-2-mm-3D_glszm_LargeAreaHighGrayLevelEmphasis_py	1164.77(1046.84)	1237.99(1099.21)	698.00(401.90)	0.115
274	log-sigma-2-mm-3D_glszm_LargeAreaLowGrayLevelEmphasis_py	0.25(0.16)	0.26(0.16)	0.19(0.08)	0.207
275	log-sigma-2-mm-3D_glszm_LowGrayLevelZoneEmphasis_py	0.02(0.01)	0.02(0.01)	0.02(0.00)	0.416
276	log-sigma-2-mm-3D_glszm_SizeZoneNonUniformity_py	2127.88(1757.35)	2138.55(1839.77)	2059.86(1185.55)	0.786
277	log-sigma-2-mm-3D_glszm_SizeZoneNonUniformityNormalized_py	0.34(0.09)	0.34(0.09)	0.39(0.08)	0.096
278	log-sigma-2-mm-3D_glszm_SmallAreaEmphasis_py	0.60(0.09)	0.59(0.09)	0.64(0.07)	0.115
279	log-sigma-2-mm-3D_glszm_SmallAreaHighGrayLevelEmphasis_py	45.17(9.45)	45.03(9.85)	46.09(6.73)	0.772
280	log-sigma-2-mm-3D_glszm_SmallAreaLowGrayLevelEmphasis_py	0.01(0.00)	0.01(0.00)	0.01(0.00)	0.21
281	log-sigma-2-mm-3D_glszm_ZoneEntropy_py	5.22(0.26)	5.24(0.27)	5.07(0.19)	0.088

282	log-sigma-2-mm-3D_glszm_ZonePercentage_py	0.42(0.10)	0.41(0.10)	0.47(0.09)	0.087
283	log-sigma-2-mm-3D_glszm_ZoneVariance_py	8.51(6.84)	9.01(7.14)	5.33(3.08)	0.1
284	log-sigma-2-mm-3D_ngtdm_Busyness_py	3.97(2.36)	4.07(2.44)	3.34(1.73)	0.425
285	log-sigma-2-mm-3D_ngtdm_Coarseness_py	0.00(0.00)	0.00(0.00)	0.00(0.00)	0.224
286	log-sigma-2-mm-3D_ngtdm_Complexity_py	30.75(6.86)	30.54(7.20)	32.06(4.16)	0.565
287	log-sigma-2-mm-3D_ngtdm_Contrast_py	0.01(0.01)	0.01(0.01)	0.01(0.01)	0.068
288	log-sigma-2-mm-3D_ngtdm_Strength_py	0.23(0.14)	0.23(0.13)	0.26(0.19)	0.526
289	log-sigma-3-mm-3D_firstorder_10Percentile_py	-37.78(12.99)	-37.12(13.31)	-42.02(10.44)	0.325
290	log-sigma-3-mm-3D_firstorder_90Percentile_py	47.12(11.80)	46.56(12.36)	50.67(6.73)	0.364
291	log-sigma-3-mm-3D_firstorder_Energy_py	18233961.51(14729921.70)	18784032.78(15469927.51)	14727257.15(8489945.68)	0.82
292	log-sigma-3-mm-3D_firstorder_Entropy_py	2.71(0.36)	2.69(0.37)	2.81(0.22)	0.64
293	log-sigma-3-mm-3D_firstorder_InterquartileRange_py	38.13(14.87)	37.54(15.08)	41.94(13.73)	0.441
294	log-sigma-3-mm-3D_firstorder_Kurtosis_py	4.37(2.20)	4.45(2.30)	3.81(1.38)	0.656
295	log-sigma-3-mm-3D_firstorder_Maximum_py	122.07(19.52)	122.21(20.36)	121.16(13.93)	0.889
296	log-sigma-3-mm-3D_firstorder_MeanAbsoluteDeviation_py	25.58(6.67)	25.25(6.86)	27.69(5.18)	0.341
297	log-sigma-3-mm-3D_firstorder_Mean_py	3.55(3.57)	3.65(3.63)	2.87(3.30)	0.569
298	log-sigma-3-mm-3D_firstorder_Median_py	2.62(6.39)	2.81(6.27)	1.43(7.43)	0.575
299	log-sigma-3-mm-3D_firstorder_Minimum_py	-122.19(18.42)	-122.47(19.01)	-120.42(15.11)	0.773
300	log-sigma-3-mm-3D_firstorder_Range_py	244.26(28.53)	244.68(30.37)	241.58(12.07)	0.612
301	log-sigma-3-mm-3D_firstorder_RobustMeanAbsoluteDeviation_py	16.46(5.79)	16.19(5.89)	18.20(5.17)	0.367
302	log-sigma-3-mm-3D_firstorder_RootMeanSquared_py	34.50(6.77)	34.18(7.06)	36.55(4.21)	0.36
303	log-sigma-3-mm-3D_firstorder_Skewness_py	-0.10(0.64)	-0.10(0.63)	-0.10(0.77)	0.988
304	log-sigma-3-mm-3D_firstorder_TotalEnergy_py	18233961.51(14729921.70)	18784032.78(15469927.51)	14727257.15(8489945.68)	0.82
305	log-sigma-3-mm-3D_firstorder_Uniformity_py	0.20(0.06)	0.20(0.07)	0.18(0.04)	0.704
306	log-sigma-3-mm-3D_firstorder_Variance_py	1210.11(438.15)	1190.68(454.33)	1334.00(309.78)	0.394
307	log-sigma-3-mm-3D_glcm_Autocorrelation_py	56.51(14.15)	57.00(14.24)	53.41(14.10)	0.51

308	log-sigma-3-mm-3D_glcm_ClusterProminence_py	544.47(246.64)	534.09(259.48)	610.65(131.55)	0.419
309	log-sigma-3-mm-3D_glcm_ClusterShade_py	-0.93(22.81)	-0.82(21.38)	-1.64(32.36)	0.926
310	log-sigma-3-mm-3D_glcm_ClusterTendency_py	11.95(4.27)	11.76(4.43)	13.19(3.04)	0.385
311	log-sigma-3-mm-3D_glcm_Contrast_py	0.47(0.14)	0.46(0.15)	0.54(0.12)	0.158
312	log-sigma-3-mm-3D_glcm_Correlation_py	0.92(0.01)	0.92(0.01)	0.92(0.01)	0.963
313	log-sigma-3-mm-3D_glcm_DifferenceAverage_py	0.39(0.10)	0.39(0.11)	0.45(0.09)	0.125
314	log-sigma-3-mm-3D_glcm_DifferenceEntropy_py	1.09(0.16)	1.08(0.17)	1.16(0.11)	0.168
315	log-sigma-3-mm-3D_glcm_DifferenceVariance_py	0.31(0.07)	0.30(0.07)	0.34(0.05)	0.23
316	log-sigma-3-mm-3D_glcm_Id_py	0.82(0.05)	0.82(0.05)	0.79(0.04)	0.114
317	log-sigma-3-mm-3D_glcm_Idm_py	0.81(0.05)	0.82(0.05)	0.79(0.04)	0.118
318	log-sigma-3-mm-3D_glcm_Idmn_py	1.00(0.00)	1.00(0.00)	1.00(0.00)	0.11
319	log-sigma-3-mm-3D_glcm_Idn_py	0.97(0.01)	0.97(0.01)	0.97(0.01)	0.082
320	log-sigma-3-mm-3D_glcm_Imc1_py	-0.50(0.04)	-0.50(0.04)	-0.47(0.04)	0.09
321	log-sigma-3-mm-3D_glcm_Imc2_py	0.96(0.01)	0.96(0.01)	0.96(0.01)	0.342
322	log-sigma-3-mm-3D_glcm_InverseVariance_py	0.32(0.07)	0.32(0.07)	0.36(0.07)	0.097
323	log-sigma-3-mm-3D_glcm_JointAverage_py	7.26(0.98)	7.30(0.98)	7.03(1.00)	0.475
324	log-sigma-3-mm-3D_glcm_JointEnergy_py	0.12(0.06)	0.12(0.07)	0.09(0.04)	0.401
325	log-sigma-3-mm-3D_glcm_JointEntropy_py	4.08(0.61)	4.04(0.63)	4.30(0.41)	0.365
326	log-sigma-3-mm-3D_glcm_MCC_py	0.92(0.01)	0.92(0.01)	0.92(0.01)	0.762
327	log-sigma-3-mm-3D_glcm_MaximumProbability_py	0.23(0.09)	0.24(0.09)	0.21(0.08)	0.327
328	log-sigma-3-mm-3D_glcm_SumAverage_py	14.52(1.96)	14.59(1.97)	14.05(2.01)	0.475
329	log-sigma-3-mm-3D_glcm_SumEntropy_py	3.53(0.46)	3.51(0.48)	3.68(0.29)	0.521
330	log-sigma-3-mm-3D_glcm_SumSquares_py	3.11(1.10)	3.06(1.14)	3.43(0.78)	0.374
331	log-sigma-3-mm-3D_gldm_DependenceEntropy_py	4.09(0.50)	4.07(0.52)	4.25(0.31)	0.521
332	log-sigma-3-mm-3D_gldm_DependenceNonUniformity_py	6281.46(5587.82)	6659.25(5881.69)	3873.07(1984.97)	0.192
333	log-sigma-3-mm-3D_gldm_DependenceNonUniformityNormalized_py	0.39(0.06)	0.39(0.06)	0.36(0.03)	0.163
334	log-sigma-3-mm-3D_gldm_DependenceVariance_py	0.53(0.08)	0.53(0.08)	0.56(0.05)	0.215
335	log-sigma-3-mm-3D_gldm_GrayLevelNonUniformity_py	3347.98(3511.83)	3567.68(3707.33)	1947.40(1191.77)	0.228

336	log-sigma-3-mm-3D_gldm_GrayLevelVariance_py	3.12(1.09)	3.07(1.13)	3.43(0.76)	0.384
337	log-sigma-3-mm-3D_gldm_HighGrayLevelEmphasis_py	56.67(14.17)	57.16(14.26)	53.57(14.06)	0.51
338	log-sigma-3-mm-3D_gldm_LargeDependenceEmphasis_py	5.71(0.74)	5.77(0.74)	5.33(0.60)	0.116
339	log-sigma-3-mm-3D_gldm_LargeDependenceHighGrayLevelEmphasis_py	321.15(96.05)	326.50(93.17)	287.02(113.61)	0.284
340	log-sigma-3-mm-3D_gldm_LargeDependenceLowGrayLevelEmphasis_py	0.15(0.06)	0.15(0.06)	0.16(0.06)	0.592
341	log-sigma-3-mm-3D_gldm_LowGrayLevelEmphasis_py	0.03(0.01)	0.03(0.01)	0.03(0.01)	0.323
342	log-sigma-3-mm-3D_gldm_SmallDependenceEmphasis_py	0.32(0.07)	0.32(0.07)	0.36(0.06)	0.123
343	log-sigma-3-mm-3D_gldm_SmallDependenceHighGrayLevelEmphasis_py	18.45(6.04)	18.39(6.42)	18.84(2.69)	0.848
344	log-sigma-3-mm-3D_gldm_SmallDependenceLowGrayLevelEmphasis_py	0.01(0.00)	0.01(0.00)	0.01(0.00)	0.212
345	log-sigma-3-mm-3D_glrlm_GrayLevelNonUniformity_py	794.07(523.82)	820.19(546.54)	627.51(321.24)	0.338
346	log-sigma-3-mm-3D_glrlm_GrayLevelNonUniformityNormalized_py	0.15(0.03)	0.15(0.03)	0.15(0.01)	0.922
347	log-sigma-3-mm-3D_glrlm_GrayLevelVariance_py	3.90(0.86)	3.89(0.92)	4.01(0.35)	0.491
348	log-sigma-3-mm-3D_glrlm_HighGrayLevelRunEmphasis_py	57.41(13.58)	57.93(13.96)	54.12(11.06)	0.466
349	log-sigma-3-mm-3D_glrlm_LongRunEmphasis_py	21.33(14.87)	22.43(15.49)	14.33(7.28)	0.143
350	log-sigma-3-mm-3D_glrlm_LongRunHighGrayLevelEmphasis_py	1185.40(982.00)	1246.10(1013.70)	798.43(672.46)	0.054
351	log-sigma-3-mm-3D_glrlm_LongRunLowGrayLevelEmphasis_py	0.51(0.39)	0.53(0.41)	0.38(0.17)	0.401

352	log-sigma-3-mm-3D_glrlm_LowGrayLevelRunEmphasis_py	0.03(0.01)	0.03(0.01)	0.03(0.01)	0.532
353	log-sigma-3-mm-3D_glrlm_RunEntropy_py	5.37(0.30)	5.40(0.30)	5.19(0.23)	0.061
354	log-sigma-3-mm-3D_glrlm_RunLengthNonUniformity_py	1608.00(1385.53)	1623.62(1454.98)	1508.40(878.55)	0.82
355	log-sigma-3-mm-3D_glrlm_RunLengthNonUniformityNormalized_py	0.29(0.08)	0.28(0.08)	0.33(0.06)	0.147
356	log-sigma-3-mm-3D_glrlm_RunPercentage_py	0.37(0.09)	0.36(0.09)	0.41(0.07)	0.117
357	log-sigma-3-mm-3D_glrlm_RunVariance_py	11.94(8.73)	12.58(9.08)	7.81(4.55)	0.131
358	log-sigma-3-mm-3D_glrlm_ShortRunEmphasis_py	0.54(0.09)	0.54(0.09)	0.59(0.06)	0.161
359	log-sigma-3-mm-3D_glrlm_ShortRunHighGrayLevelEmphasis_py	30.99(9.62)	31.02(10.27)	30.80(3.55)	0.953
360	log-sigma-3-mm-3D_glrlm_ShortRunLowGrayLevelEmphasis_py	0.02(0.01)	0.02(0.01)	0.02(0.00)	0.424
361	log-sigma-3-mm-3D_glszm_GrayLevelNonUniformity_py	794.07(523.82)	820.19(546.54)	627.51(321.24)	0.338
362	log-sigma-3-mm-3D_glszm_GrayLevelNonUniformityNormalized_py	0.15(0.03)	0.15(0.03)	0.15(0.01)	0.922
363	log-sigma-3-mm-3D_glszm_GrayLevelVariance_py	3.90(0.86)	3.89(0.92)	4.01(0.35)	0.491
364	log-sigma-3-mm-3D_glszm_HighGrayLevelZoneEmphasis_py	57.41(13.58)	57.93(13.96)	54.12(11.06)	0.466
365	log-sigma-3-mm-3D_glszm_LargeAreaEmphasis_py	21.33(14.87)	22.43(15.49)	14.33(7.28)	0.143
366	log-sigma-3-mm-3D_glszm_LargeAreaHighGrayLevelEmphasis_py	1185.40(982.00)	1246.10(1013.70)	798.43(672.46)	0.054
367	log-sigma-3-mm-3D_glszm_LargeAreaLowGrayLevelEmphasis_py	0.51(0.39)	0.53(0.41)	0.38(0.17)	0.401
368	log-sigma-3-mm-3D_glszm_LowGrayLevelZoneEmphasis_py	0.03(0.01)	0.03(0.01)	0.03(0.01)	0.532
369	log-sigma-3-mm-3D_glszm_SizeZoneNonUniformity_py	1608.00(1385.53)	1623.62(1454.98)	1508.40(878.55)	0.82

370	log-sigma-3-mm-3D_glszm_SizeZoneNonUniformityNormalized_py	0.29(0.08)	0.28(0.08)	0.33(0.06)	0.147
371	log-sigma-3-mm-3D_glszm_SmallAreaEmphasis_py	0.54(0.09)	0.54(0.09)	0.59(0.06)	0.161
372	log-sigma-3-mm-3D_glszm_SmallAreaHighGrayLevelEmphasis_py	30.99(9.62)	31.02(10.27)	30.80(3.55)	0.953
373	log-sigma-3-mm-3D_glszm_SmallAreaLowGrayLevelEmphasis_py	0.02(0.01)	0.02(0.01)	0.02(0.00)	0.424
374	log-sigma-3-mm-3D_glszm_ZoneEntropy_py	5.37(0.30)	5.40(0.30)	5.19(0.23)	0.061
375	log-sigma-3-mm-3D_glszm_ZonePercentage_py	0.37(0.09)	0.36(0.09)	0.41(0.07)	0.117
376	log-sigma-3-mm-3D_glszm_ZoneVariance_py	11.94(8.73)	12.58(9.08)	7.81(4.55)	0.131
377	log-sigma-3-mm-3D_ngtdm_Busyness_py	4.75(3.00)	4.83(3.03)	4.23(2.89)	0.601
378	log-sigma-3-mm-3D_ngtdm_Coarseness_py	0.00(0.00)	0.00(0.00)	0.00(0.00)	0.269
379	log-sigma-3-mm-3D_ngtdm_Complexity_py	17.39(4.60)	17.38(4.94)	17.44(0.95)	0.941
380	log-sigma-3-mm-3D_ngtdm_Contrast_py	0.01(0.00)	0.01(0.00)	0.01(0.00)	0.183
381	log-sigma-3-mm-3D_ngtdm_Strength_py	0.20(0.12)	0.19(0.12)	0.23(0.15)	0.579
382	lbp-2D_firstorder_10Percentile_py	1.61(0.67)	1.65(0.69)	1.38(0.52)	0.326
383	lbp-2D_firstorder_90Percentile_py	8.51(0.68)	8.47(0.70)	8.75(0.46)	0.301
384	lbp-2D_firstorder_Energy_py	421436.14(299469.84)	437819.35(313036.58)	316993.12(169459.68)	0.293
385	lbp-2D_firstorder_InterquartileRange_py*	3.05(0.95)	2.92(0.91)	3.88(0.83)	0.011
386	lbp-2D_firstorder_Kurtosis_py	2.66(0.60)	2.72(0.61)	2.29(0.32)	0.059
387	lbp-2D_firstorder_MeanAbsoluteDeviation_py*	1.87(0.29)	1.84(0.28)	2.08(0.27)	0.025
388	lbp-2D_firstorder_Mean_py	4.67(0.14)	4.66(0.13)	4.75(0.16)	0.083
389	lbp-2D_firstorder_Median_py*	4.12(0.33)	4.08(0.27)	4.38(0.52)	0.018
390	lbp-2D_firstorder_RobustMeanAbsoluteDeviation_py*	1.57(0.44)	1.53(0.44)	1.81(0.40)	0.037
391	lbp-2D_firstorder_RootMeanSquared_py*	5.24(0.24)	5.21(0.23)	5.40(0.26)	0.043
392	lbp-2D_firstorder_Skewness_py	0.24(0.19)	0.26(0.18)	0.12(0.18)	0.05
393	lbp-2D_firstorder_TotalEnergy_py	421436.14(299469.84)	437819.35(313036.58)	316993.12(169459.68)	0.293
394	lbp-2D_firstorder_Variance_py*	5.67(1.27)	5.52(1.22)	6.61(1.26)	0.023
395	lbp-2D_gldm_DependenceEntropy_py	0.22(0.07)	0.23(0.07)	0.22(0.07)	0.878
396	lbp-2D_gldm_DependenceNonUniformity_py	14678.89(11250.03)	15399.80(11782.72)	10083.07(5392.59)	0.309

397	lbp-2D_gldm_DependenceNonUniformityNormalized_py	0.93(0.03)	0.93(0.03)	0.93(0.03)	0.858
398	lbp-2D_gldm_DependenceVariance_py	0.04(0.01)	0.04(0.02)	0.04(0.01)	0.89
399	lbp-2D_gldm_GrayLevelNonUniformity_py	15561.58(11615.29)	16319.39(12154.58)	10730.50(5636.32)	0.26
400	lbp-2D_gldm_LargeDependenceEmphasis_py	8.82(0.08)	8.82(0.08)	8.82(0.08)	0.862
401	lbp-2D_gldm_LargeDependenceHighGrayLevelEmphasis_py	8.82(0.08)	8.82(0.08)	8.82(0.08)	0.862
402	lbp-2D_gldm_LargeDependenceLowGrayLevelEmphasis_py	8.82(0.08)	8.82(0.08)	8.82(0.08)	0.862
403	lbp-2D_gldm_SmallDependenceEmphasis_py	0.12(0.00)	0.12(0.00)	0.12(0.00)	0.907
404	lbp-2D_gldm_SmallDependenceHighGrayLevelEmphasis_py	0.12(0.00)	0.12(0.00)	0.12(0.00)	0.907
405	lbp-2D_gldm_SmallDependenceLowGrayLevelEmphasis_py	0.12(0.00)	0.12(0.00)	0.12(0.00)	0.907
406	lbp-2D_glrlm_GrayLevelNonUniformity_py	230.02(107.47)	239.49(109.27)	169.62(74.78)	0.087
407	lbp-2D_glrlm_LongRunEmphasis_py	5943.19(4798.66)	5983.20(4995.70)	5688.15(3527.86)	0.873
408	lbp-2D_glrlm_LongRunHighGrayLevelEmphasis_py	5943.19(4798.66)	5983.20(4995.70)	5688.15(3527.86)	0.873
409	lbp-2D_glrlm_LongRunLowGrayLevelEmphasis_py	5943.19(4798.66)	5983.20(4995.70)	5688.15(3527.86)	0.873
410	lbp-2D_glrlm_RunEntropy_py	5.97(0.62)	5.97(0.64)	5.95(0.49)	0.959
411	lbp-2D_glrlm_RunLengthNonUniformity_py	4.85(2.20)	5.06(2.23)	3.52(1.47)	0.067
412	lbp-2D_glrlm_RunLengthNonUniformityNormalized_py	0.02(0.01)	0.02(0.01)	0.02(0.01)	0.991
413	lbp-2D_glrlm_RunPercentage_py	0.02(0.01)	0.02(0.01)	0.02(0.01)	0.866
414	lbp-2D_glrlm_RunVariance_py	1400.45(1442.48)	1422.47(1509.95)	1260.05(963.93)	0.991
415	lbp-2D_glrlm_ShortRunEmphasis_py	0.02(0.01)	0.02(0.01)	0.02(0.01)	0.15
416	lbp-2D_glrlm_ShortRunHighGrayLevelEmphasis_py	0.02(0.01)	0.02(0.01)	0.02(0.01)	0.15

417	lbp-2D_glrlm_ShortRunLowGrayLevelEmphasis_py	0.02(0.01)	0.02(0.01)	0.02(0.01)	0.15
418	lbp-2D_glszm_GrayLevelNonUniformity_py	230.02(107.47)	239.49(109.27)	169.62(74.78)	0.087
419	lbp-2D_glszm_LargeAreaEmphasis_py	5943.19(4798.66)	5983.20(4995.70)	5688.15(3527.86)	0.873
420	lbp-2D_glszm_LargeAreaHighGrayLevelEmphasis_py	5943.19(4798.66)	5983.20(4995.70)	5688.15(3527.86)	0.873
421	lbp-2D_glszm_LargeAreaLowGrayLevelEmphasis_py	5943.19(4798.66)	5983.20(4995.70)	5688.15(3527.86)	0.873
422	lbp-2D_glszm_SizeZoneNonUniformity_py	4.85(2.20)	5.06(2.23)	3.52(1.47)	0.067
423	lbp-2D_glszm_SizeZoneNonUniformityNormalized_py	0.02(0.01)	0.02(0.01)	0.02(0.01)	0.991
424	lbp-2D_glszm_SmallAreaEmphasis_py	0.02(0.01)	0.02(0.01)	0.02(0.01)	0.15
425	lbp-2D_glszm_SmallAreaHighGrayLevelEmphasis_py	0.02(0.01)	0.02(0.01)	0.02(0.01)	0.15
426	lbp-2D_glszm_SmallAreaLowGrayLevelEmphasis_py	0.02(0.01)	0.02(0.01)	0.02(0.01)	0.15
427	lbp-2D_glszm_ZoneEntropy_py	5.97(0.62)	5.97(0.64)	5.95(0.49)	0.959
428	lbp-2D_glszm_ZonePercentage_py	0.02(0.01)	0.02(0.01)	0.02(0.01)	0.866
429	lbp-2D_glszm_ZoneVariance_py	1400.45(1442.48)	1422.47(1509.95)	1260.05(963.93)	0.991
430	wavelet-H_firstorder_10Percentile_py	-15.83(22.62)	-13.00(17.26)	-33.90(40.99)	0.067
431	wavelet-H_firstorder_90Percentile_py	16.81(22.94)	14.10(18.25)	34.10(39.82)	0.067
432	wavelet-H_firstorder_Energy_py	21616964.69(17130202.49)	21632902.57(17823245.68)	21515360.72(12740501.71)	0.986
433	wavelet-H_firstorder_Entropy_py*	1.67(0.22)	1.65(0.21)	1.83(0.26)	0.031
434	wavelet-H_firstorder_InterquartileRange_py*	4.39(2.39)	4.07(1.98)	6.37(3.77)	0.01
435	wavelet-H_firstorder_Kurtosis_py	12.55(7.56)	13.13(7.88)	8.81(3.32)	0.095
436	wavelet-H_firstorder_Maximum_py	216.36(13.59)	216.16(14.59)	217.67(2.42)	0.715
437	wavelet-H_firstorder_MeanAbsoluteDeviation_py*	16.00(6.06)	15.33(5.65)	20.26(7.21)	0.031
438	wavelet-H_firstorder_Mean_py	0.21(0.29)	0.23(0.30)	0.14(0.16)	0.377
439	wavelet-H_firstorder_Median_py	-0.05(0.10)	-0.06(0.10)	-0.02(0.10)	0.3
440	wavelet-H_firstorder_Minimum_py	-217.85(2.03)	-217.84(2.10)	-217.88(1.62)	0.963
441	wavelet-H_firstorder_Range_py	434.21(14.32)	434.00(15.36)	435.55(3.16)	0.579
442	wavelet-H_firstorder_RobustMeanAbsoluteDeviation_py	2.56(1.86)	2.30(1.36)	4.23(3.41)	0.156

443	wavelet-H_firstorder_RootMeanSquared_py*	38.04(8.43)	37.19(8.14)	43.50(8.75)	0.048
444	wavelet-H_firstorder_Skewness_py	-0.00(0.27)	-0.00(0.28)	-0.01(0.26)	0.969
445	wavelet-H_firstorder_TotalEnergy_py	21616964.69(17130202.49)	21632902.57(17823245.68)	21515360.72(12740501.71)	0.986
446	wavelet-H_firstorder_Uniformity_py*	0.39(0.04)	0.39(0.04)	0.36(0.05)	0.032
447	wavelet-H_firstorder_Variance_py*	1516.92(627.95)	1447.55(580.15)	1959.19(777.51)	0.031
448	wavelet-H_glcm_Autocorrelation_py	136.21(9.99)	136.15(9.96)	136.55(10.87)	0.656
449	wavelet-H_glcm_ClusterProminence_py*	234.73(112.78)	221.21(99.49)	320.93(157.69)	0.019
450	wavelet-H_glcm_ClusterShade_py	0.97(2.34)	0.98(2.31)	0.96(2.69)	0.988
451	wavelet-H_glcm_ClusterTendency_py*	5.09(1.93)	4.87(1.76)	6.52(2.45)	0.023
452	wavelet-H_glcm_Contrast_py*	10.65(4.32)	10.16(3.98)	13.72(5.36)	0.029
453	wavelet-H_glcm_Correlation_py	-0.34(0.03)	-0.34(0.03)	-0.35(0.01)	0.385
454	wavelet-H_glcm_DifferenceAverage_py*	1.51(0.48)	1.46(0.44)	1.84(0.57)	0.03
455	wavelet-H_glcm_DifferenceEntropy_py*	1.78(0.27)	1.75(0.25)	1.97(0.30)	0.031
456	wavelet-H_glcm_DifferenceVariance_py*	8.15(2.84)	7.85(2.70)	10.04(3.15)	0.042
457	wavelet-H_glcm_Id_py*	0.69(0.05)	0.70(0.05)	0.65(0.06)	0.033
458	wavelet-H_glcm_Idm_py*	0.67(0.06)	0.68(0.06)	0.63(0.07)	0.033
459	wavelet-H_glcm_Idmn_py*	0.98(0.01)	0.98(0.01)	0.98(0.01)	0.029
460	wavelet-H_glcm_Idn_py*	0.95(0.02)	0.95(0.01)	0.94(0.02)	0.03
461	wavelet-H_glcm_Imc1_py	-0.22(0.03)	-0.22(0.03)	-0.23(0.03)	0.134
462	wavelet-H_glcm_Imc2_py	0.71(0.07)	0.71(0.07)	0.75(0.06)	0.089
463	wavelet-H_glcm_InverseVariance_py	0.34(0.02)	0.34(0.02)	0.33(0.01)	0.26
464	wavelet-H_glcm_JointAverage_py	11.72(0.42)	11.72(0.41)	11.75(0.46)	0.493
465	wavelet-H_glcm_JointEnergy_py*	0.19(0.03)	0.19(0.03)	0.16(0.03)	0.036
466	wavelet-H_glcm_JointEntropy_py*	2.97(0.35)	2.93(0.33)	3.22(0.41)	0.026
467	wavelet-H_glcm_MCC_py	0.65(0.03)	0.65(0.03)	0.64(0.03)	0.71
468	wavelet-H_glcm_MaximumProbability_py*	0.27(0.03)	0.27(0.03)	0.24(0.04)	0.037
469	wavelet-H_glcm_SumAverage_py	23.45(0.83)	23.44(0.83)	23.51(0.93)	0.493
470	wavelet-H_glcm_SumEntropy_py*	2.12(0.17)	2.10(0.16)	2.24(0.19)	0.027
471	wavelet-H_glcm_SumSquares_py*	3.93(1.56)	3.76(1.43)	5.06(1.95)	0.027
472	wavelet-H_gldm_DependenceEntropy_py	2.96(0.17)	2.95(0.16)	3.07(0.18)	0.05
473	wavelet-H_gldm_DependenceNonUniformity_py	5565.16(4254.28)	5836.64(4462.73)	3834.44(1935.32)	0.219
474	wavelet-H_gldm_DependenceNonUniformityNormalized_py	0.36(0.01)	0.36(0.01)	0.36(0.01)	0.55
475	wavelet-H_gldm_DependenceVariance_py	0.55(0.04)	0.55(0.04)	0.55(0.03)	0.786

476	wavelet-H_gldm_GrayLevelNonUniformity_py	6210.57(5091.48)	6581.40(5330.83)	3846.49(2102.04)	0.16
477	wavelet-H_gldm_GrayLevelVariance_py*	3.98(1.55)	3.80(1.43)	5.07(1.92)	0.03
478	wavelet-H_gldm_HighGrayLevelEmphasis_py	141.54(10.13)	141.25(9.90)	143.40(12.09)	0.414
479	wavelet-H_gldm_LargeDependenceEmphasis_py*	4.50(0.46)	4.55(0.44)	4.19(0.51)	0.039
480	wavelet-H_gldm_LargeDependenceHighGrayLevelEmphasis_py	622.20(75.35)	628.36(76.06)	582.91(60.73)	0.113
481	wavelet-H_gldm_LargeDependenceLowGrayLevelEmphasis_py	0.04(0.00)	0.04(0.00)	0.03(0.01)	0.12
482	wavelet-H_gldm_LowGrayLevelEmphasis_py	0.01(0.00)	0.01(0.00)	0.01(0.00)	0.523
483	wavelet-H_gldm_SmallDependenceEmphasis_py	0.43(0.06)	0.43(0.06)	0.47(0.07)	0.054
484	wavelet-H_gldm_SmallDependenceHighGrayLevelEmphasis_py	63.46(10.91)	62.39(10.05)	70.31(14.26)	0.056
485	wavelet-H_gldm_SmallDependenceLowGrayLevelEmphasis_py	0.01(0.00)	0.01(0.00)	0.01(0.00)	0.242
486	wavelet-H_glrlm_GrayLevelNonUniformity_py	2447.11(1956.39)	2577.61(2052.67)	1615.16(837.79)	0.232
487	wavelet-H_glrlm_GrayLevelNonUniformityNormalized_py	0.31(0.05)	0.32(0.05)	0.28(0.05)	0.054
488	wavelet-H_glrlm_GrayLevelVariance_py*	7.33(2.24)	7.10(2.15)	8.78(2.37)	0.048
489	wavelet-H_glrlm_HighGrayLevelRunEmphasis_py	145.43(10.11)	145.12(9.86)	147.39(12.16)	0.55
490	wavelet-H_glrlm_LongRunEmphasis_py	6.10(1.46)	6.24(1.44)	5.17(1.29)	0.052
491	wavelet-H_glrlm_LongRunHighGrayLevelEmphasis_py	843.96(205.57)	863.82(206.91)	717.35(152.13)	0.06
492	wavelet-H_glrlm_LongRunLowGrayLevelEmphasis_py	0.05(0.01)	0.05(0.01)	0.04(0.01)	0.07
493	wavelet-H_glrlm_LowGrayLevelRunEmphasis_py	0.01(0.00)	0.01(0.00)	0.01(0.00)	0.639
494	wavelet-H_glrlm_RunEntropy_py	3.74(0.09)	3.74(0.09)	3.73(0.05)	0.739
495	wavelet-H_glrlm_RunLengthNonUniformity_py	2870.48(2095.28)	2924.24(2191.27)	2527.76(1386.75)	0.888
496	wavelet-H_glrlm_RunLengthNonUniformityNormalized_py	0.38(0.07)	0.37(0.07)	0.42(0.08)	0.085
497	wavelet-H_glrlm_RunPercentage_py*	0.51(0.06)	0.50(0.06)	0.55(0.07)	0.043
498	wavelet-H_glrlm_RunVariance_py*	2.06(0.51)	2.11(0.49)	1.71(0.46)	0.035

499	wavelet-H_glrlm_ShortRunEmphasis_py	0.63(0.07)	0.62(0.07)	0.67(0.07)	0.143
500	wavelet-H_glrlm_ShortRunHighGrayLevelEmphasis_py	94.22(13.62)	93.11(13.06)	101.25(15.89)	0.117
501	wavelet-H_glrlm_ShortRunLowGrayLevelEmphasis_py	0.01(0.00)	0.01(0.00)	0.01(0.00)	0.424
502	wavelet-H_glszm_GrayLevelNonUniformity_py	2447.11(1956.39)	2577.61(2052.67)	1615.16(837.79)	0.232
503	wavelet-H_glszm_GrayLevelNonUniformityNormalized_py	0.31(0.05)	0.32(0.05)	0.28(0.05)	0.054
504	wavelet-H_glszm_GrayLevelVariance_py*	7.33(2.24)	7.10(2.15)	8.78(2.37)	0.048
505	wavelet-H_glszm_HighGrayLevelZoneEmphasis_py	145.43(10.11)	145.12(9.86)	147.39(12.16)	0.55
506	wavelet-H_glszm_LargeAreaEmphasis_py	6.10(1.46)	6.24(1.44)	5.17(1.29)	0.052
507	wavelet-H_glszm_LargeAreaHighGrayLevelEmphasis_py	843.96(205.57)	863.82(206.91)	717.35(152.13)	0.06
508	wavelet-H_glszm_LargeAreaLowGrayLevelEmphasis_py	0.05(0.01)	0.05(0.01)	0.04(0.01)	0.07
509	wavelet-H_glszm_LowGrayLevelZoneEmphasis_py	0.01(0.00)	0.01(0.00)	0.01(0.00)	0.639
510	wavelet-H_glszm_SizeZoneNonUniformity_py	2870.48(2095.28)	2924.24(2191.27)	2527.76(1386.75)	0.888
511	wavelet-H_glszm_SizeZoneNonUniformityNormalized_py	0.38(0.07)	0.37(0.07)	0.42(0.08)	0.085
512	wavelet-H_glszm_SmallAreaEmphasis_py	0.63(0.07)	0.62(0.07)	0.67(0.07)	0.143
513	wavelet-H_glszm_SmallAreaHighGrayLevelEmphasis_py	94.22(13.62)	93.11(13.06)	101.25(15.89)	0.117
514	wavelet-H_glszm_SmallAreaLowGrayLevelEmphasis_py	0.01(0.00)	0.01(0.00)	0.01(0.00)	0.424
515	wavelet-H_glszm_ZoneEntropy_py	3.74(0.09)	3.74(0.09)	3.73(0.05)	0.739
516	wavelet-H_glszm_ZonePercentage_py*	0.51(0.06)	0.50(0.06)	0.55(0.07)	0.043
517	wavelet-H_glszm_ZoneVariance_py*	2.06(0.51)	2.11(0.49)	1.71(0.46)	0.035
518	wavelet-H_ngtdm_Busyness_py	13.48(8.34)	13.85(8.70)	11.14(5.39)	0.398
519	wavelet-H_ngtdm_Coarseness_py	0.00(0.00)	0.00(0.00)	0.00(0.00)	0.672
520	wavelet-H_ngtdm_Complexity_py	219.98(84.21)	213.14(77.97)	263.58(113.25)	0.116
521	wavelet-H_ngtdm_Contrast_py*	0.07(0.05)	0.06(0.04)	0.10(0.05)	0.044
522	wavelet-H_ngtdm_Strength_py	0.09(0.06)	0.09(0.06)	0.10(0.07)	0.874
523	wavelet-L_firstorder_10Percentile_py	-156.86(45.98)	-155.41(49.31)	-166.11(5.76)	0.688
524	wavelet-L_firstorder_90Percentile_py	128.83(78.16)	125.27(82.53)	151.59(35.90)	0.465

525	wavelet-L_firstorder_Energy_py	306949332.43(228127595.53)	319687875.23(238339441.91)	225741122.07(129355065.47)	0.479
526	wavelet-L_firstorder_Entropy_py	3.32(0.35)	3.31(0.35)	3.36(0.37)	0.695
527	wavelet-L_firstorder_InterquartileRange_py	166.15(111.01)	166.30(111.07)	165.23(118.27)	0.905
528	wavelet-L_firstorder_Kurtosis_py	3.84(3.83)	3.98(4.03)	2.91(2.16)	0.564
529	wavelet-L_firstorder_Maximum_py	218.84(2.73)	218.89(2.89)	218.47(1.30)	0.097
530	wavelet-L_firstorder_MeanAbsoluteDeviation_py	100.18(33.54)	99.10(34.31)	107.06(29.08)	0.537
531	wavelet-L_firstorder_Mean_py	-23.83(67.48)	-26.15(67.17)	-9.03(72.25)	0.51
532	wavelet-L_firstorder_Median_py	-33.96(116.54)	-39.04(114.92)	-1.53(129.70)	0.72
533	wavelet-L_firstorder_Minimum_py	-219.69(3.60)	-219.96(3.11)	-217.94(5.87)	0.17
534	wavelet-L_firstorder_Range_py	438.53(5.18)	438.86(4.82)	436.41(7.11)	0.108
535	wavelet-L_firstorder_RobustMeanAbsoluteDeviation_py	76.15(39.74)	75.26(39.99)	81.84(40.25)	0.667
536	wavelet-L_firstorder_RootMeanSquared_py	140.46(10.41)	140.02(11.02)	143.28(4.47)	0.154
537	wavelet-L_firstorder_Skewness_py	0.52(1.42)	0.57(1.44)	0.22(1.33)	0.526
538	wavelet-L_firstorder_TotalEnergy_py	306949332.43(228127595.53)	319687875.23(238339441.91)	225741122.07(129355065.47)	0.479
539	wavelet-L_firstorder_Uniformity_py	0.13(0.04)	0.13(0.04)	0.13(0.05)	0.672
540	wavelet-L_firstorder_Variance_py	14792.10(5406.22)	14618.85(5579.92)	15896.56(4261.38)	0.539
541	wavelet-L_glcm_Autocorrelation_py	164.99(90.99)	162.67(90.94)	179.81(96.13)	0.625
542	wavelet-L_glcm_ClusterProminence_py	48225.84(14024.89)	47848.93(14766.40)	50628.66(8041.72)	0.607
543	wavelet-L_glcm_ClusterShade_py	372.56(1572.54)	431.60(1556.90)	-3.85(1728.70)	0.625
544	wavelet-L_glcm_ClusterTendency_py	138.71(52.30)	137.26(54.09)	147.95(40.67)	0.595
545	wavelet-L_glcm_Contrast_py*	8.17(3.26)	7.83(3.07)	10.36(3.81)	0.04
546	wavelet-L_glcm_Correlation_py	0.88(0.03)	0.89(0.03)	0.87(0.02)	0.125
547	wavelet-L_glcm_DifferenceAverage_py*	1.33(0.45)	1.28(0.43)	1.64(0.52)	0.035
548	wavelet-L_glcm_DifferenceEntropy_py*	1.91(0.38)	1.88(0.37)	2.16(0.36)	0.044
549	wavelet-L_glcm_DifferenceVariance_py	6.20(2.06)	6.01(2.01)	7.43(2.05)	0.069
550	wavelet-L_glcm_Id_py*	0.71(0.07)	0.72(0.07)	0.67(0.08)	0.042
551	wavelet-L_glcm_Idm_py*	0.69(0.08)	0.70(0.08)	0.64(0.09)	0.041
552	wavelet-L_glcm_Idmn_py*	0.99(0.01)	0.99(0.01)	0.98(0.01)	0.018
553	wavelet-L_glcm_Idn_py*	0.95(0.02)	0.96(0.01)	0.94(0.02)	0.024
554	wavelet-L_glcm_Imc1_py*	-0.48(0.08)	-0.49(0.08)	-0.43(0.05)	0.043
555	wavelet-L_glcm_Imc2_py*	0.98(0.01)	0.98(0.01)	0.97(0.01)	0.031

556	wavelet-L_glcm_InverseVariance_py	0.27(0.04)	0.27(0.05)	0.29(0.03)	0.302
557	wavelet-L_glcm_JointAverage_py	10.92(3.64)	10.83(3.63)	11.50(3.89)	0.636
558	wavelet-L_glcm_JointEnergy_py	0.07(0.04)	0.07(0.04)	0.06(0.04)	0.488
559	wavelet-L_glcm_JointEntropy_py	5.02(0.67)	4.98(0.66)	5.28(0.71)	0.254
560	wavelet-L_glcm_MCC_py	0.93(0.03)	0.93(0.03)	0.92(0.02)	0.116
561	wavelet-L_glcm_MaximumProbability_py	0.15(0.06)	0.15(0.06)	0.14(0.07)	0.762
562	wavelet-L_glcm_SumAverage_py	21.85(7.28)	21.67(7.26)	23.00(7.77)	0.636
563	wavelet-L_glcm_SumEntropy_py	4.00(0.41)	3.99(0.42)	4.06(0.42)	0.669
564	wavelet-L_glcm_SumSquares_py	36.72(13.69)	36.27(14.11)	39.58(10.98)	0.53
565	wavelet-L_gldm_DependenceEntropy_py	4.58(0.42)	4.57(0.43)	4.64(0.41)	0.66
566	wavelet-L_gldm_DependenceNonUniformity_py	5724.00(4859.94)	6031.56(5119.17)	3763.33(1852.78)	0.232
567	wavelet-L_gldm_DependenceNonUniformityNormalized_py	0.36(0.03)	0.36(0.03)	0.35(0.02)	0.82
568	wavelet-L_gldm_DependenceVariance_py	0.60(0.06)	0.59(0.07)	0.60(0.04)	0.769
569	wavelet-L_gldm_GrayLevelNonUniformity_py	2097.80(1890.63)	2206.21(1979.86)	1406.70(993.37)	0.27
570	wavelet-L_gldm_GrayLevelVariance_py	36.96(13.48)	36.53(13.91)	39.75(10.65)	0.534
571	wavelet-L_gldm_HighGrayLevelEmphasis_py	170.12(91.01)	167.62(90.89)	186.03(96.39)	0.599
572	wavelet-L_gldm_LargeDependenceEmphasis_py	4.96(0.82)	5.04(0.80)	4.44(0.78)	0.055
573	wavelet-L_gldm_LargeDependenceHighGrayLevelEmphasis_py	814.06(546.49)	814.66(552.87)	810.23(539.56)	0.939
574	wavelet-L_gldm_LargeDependenceLowGrayLevelEmphasis_py	0.14(0.07)	0.14(0.07)	0.14(0.07)	0.814
575	wavelet-L_gldm_LowGrayLevelEmphasis_py	0.03(0.01)	0.03(0.01)	0.03(0.01)	0.537
576	wavelet-L_gldm_SmallDependenceEmphasis_py	0.41(0.09)	0.40(0.09)	0.47(0.09)	0.063
577	wavelet-L_gldm_SmallDependenceHighGrayLevelEmphasis_py	74.15(32.35)	71.79(31.24)	89.18(37.47)	0.159
578	wavelet-L_gldm_SmallDependenceLowGrayLevelEmphasis_py	0.01(0.01)	0.01(0.01)	0.02(0.01)	0.192
579	wavelet-L_glrlm_GrayLevelNonUniformity_py	671.05(479.23)	694.41(501.32)	522.14(280.06)	0.349
580	wavelet-L_glrlm_GrayLevelNonUniformityNormalized_py	0.10(0.02)	0.10(0.02)	0.10(0.02)	0.535

581	wavelet-L_glrlm_GrayLevelVariance_py	42.79(10.25)	42.48(10.81)	44.74(5.57)	0.567
582	wavelet-L_glrlm_HighGrayLevelRunEmphasis_py	175.78(69.17)	174.09(69.62)	186.53(69.78)	0.64
583	wavelet-L_glrlm_LongRunEmphasis_py	11.42(7.31)	12.02(7.57)	7.62(3.74)	0.114
584	wavelet-L_glrlm_LongRunHighGrayLevelEmphasis_py	1787.53(1624.50)	1862.67(1693.59)	1308.51(1037.00)	0.594
585	wavelet-L_glrlm_LongRunLowGrayLevelEmphasis_py	0.33(0.27)	0.34(0.28)	0.24(0.17)	0.309
586	wavelet-L_glrlm_LowGrayLevelRunEmphasis_py	0.03(0.01)	0.03(0.01)	0.04(0.01)	0.546
587	wavelet-L_glrlm_RunEntropy_py	5.38(0.33)	5.41(0.34)	5.20(0.18)	0.096
588	wavelet-L_glrlm_RunLengthNonUniformity_py	2644.49(1917.61)	2653.47(1995.28)	2587.25(1423.78)	0.929
589	wavelet-L_glrlm_RunLengthNonUniformityNormalized_py	0.40(0.10)	0.39(0.10)	0.45(0.10)	0.116
590	wavelet-L_glrlm_RunPercentage_py	0.46(0.10)	0.45(0.10)	0.52(0.10)	0.056
591	wavelet-L_glrlm_RunVariance_py	5.82(4.16)	6.17(4.31)	3.59(2.03)	0.104
592	wavelet-L_glrlm_ShortRunEmphasis_py	0.64(0.10)	0.64(0.10)	0.69(0.08)	0.134
593	wavelet-L_glrlm_ShortRunHighGrayLevelEmphasis_py	119.49(38.19)	117.42(37.92)	132.68(39.77)	0.298
594	wavelet-L_glrlm_ShortRunLowGrayLevelEmphasis_py	0.02(0.01)	0.02(0.01)	0.03(0.01)	0.439
595	wavelet-L_glszm_GrayLevelNonUniformity_py	671.05(479.23)	694.41(501.32)	522.14(280.06)	0.349
596	wavelet-L_glszm_GrayLevelNonUniformityNormalized_py	0.10(0.02)	0.10(0.02)	0.10(0.02)	0.535
597	wavelet-L_glszm_GrayLevelVariance_py	42.79(10.25)	42.48(10.81)	44.74(5.57)	0.567
598	wavelet-L_glszm_HighGrayLevelZoneEmphasis_py	175.78(69.17)	174.09(69.62)	186.53(69.78)	0.64
599	wavelet-L_glszm_LargeAreaEmphasis_py	11.42(7.31)	12.02(7.57)	7.62(3.74)	0.114
600	wavelet-L_glszm_LargeAreaHighGrayLevelEmphasis_py	1787.53(1624.50)	1862.67(1693.59)	1308.51(1037.00)	0.594
601	wavelet-L_glszm_LargeAreaLowGrayLevelEmphasis_py	0.33(0.27)	0.34(0.28)	0.24(0.17)	0.309
602	wavelet-L_glszm_LowGrayLevelZoneEmphasis_py	0.03(0.01)	0.03(0.01)	0.04(0.01)	0.546
603	wavelet-L_glszm_SizeZoneNonUniformity_py	2644.49(1917.61)	2653.47(1995.28)	2587.25(1423.78)	0.929
604	wavelet-L_glszm_SizeZoneNonUniformityNormalized_py	0.40(0.10)	0.39(0.10)	0.45(0.10)	0.116
605	wavelet-L_glszm_SmallAreaEmphasis_py	0.64(0.10)	0.64(0.10)	0.69(0.08)	0.134

606	wavelet-L_glszm_SmallAreaHighGrayLevelEmphasis_py	119.49(38.19)	117.42(37.92)	132.68(39.77)	0.298
607	wavelet-L_glszm_SmallAreaLowGrayLevelEmphasis_py	0.02(0.01)	0.02(0.01)	0.03(0.01)	0.439
608	wavelet-L_glszm_ZoneEntropy_py	5.38(0.33)	5.41(0.34)	5.20(0.18)	0.096
609	wavelet-L_glszm_ZonePercentage_py	0.46(0.10)	0.45(0.10)	0.52(0.10)	0.056
610	wavelet-L_glszm_ZoneVariance_py	5.82(4.16)	6.17(4.31)	3.59(2.03)	0.104
611	wavelet-L_ngtdm_Busyness_py	3.36(2.50)	3.49(2.66)	2.54(0.67)	0.323
612	wavelet-L_ngtdm_Coarseness_py	0.00(0.00)	0.00(0.00)	0.00(0.00)	0.991
613	wavelet-L_ngtdm_Complexity_py	214.63(65.80)	208.99(63.52)	250.58(73.05)	0.097
614	wavelet-L_ngtdm_Contrast_py	0.15(0.08)	0.14(0.08)	0.19(0.08)	0.094
615	wavelet-L_ngtdm_Strength_py	0.53(0.56)	0.53(0.57)	0.55(0.46)	0.957
