# Supplementary material for: Integrating computed tomography and biopsy images to predict chemotherapy response in gastric cancer
Source: Front Oncol. 2025 Oct 21;15:1666358. doi: 10.3389/fonc.2025.1666358 (PMC12583031; doi:10.3389/fonc.2025.1666358)
Supplement: Supplementary file 3 [file DataSheet3.pdf]

Pathomic features of the training cohort

	Pathomic Feature names	All cases in the Training Cohort (n=236)	Non pathological complete response cases (n=202)	Pathological complete response cases (n=34)	p-value
1	Count_IdentifyPrimaryObjects_cell20*	2250.19(1047.33)	2176.92(1015.34)	2685.50(1141.92)	0.003
2	ExecutionTime_03NamesAndTypes_cell20	0.57(0.74)	0.55(0.71)	0.65(0.90)	0.649
3	ExecutionTime_05UnmixColors_cell20	0.59(0.24)	0.60(0.25)	0.56(0.17)	0.42
4	ExecutionTime_06IdentifyPrimaryObjects_cell20*	3.36(1.55)	3.27(1.51)	3.87(1.67)	0.026
5	ExecutionTime_07MeasureObjectIntensity_cell20*	0.87(0.59)	0.82(0.57)	1.12(0.62)	<0.001
6	ExecutionTime_08MeasureObjectSizeShape_cell20*	14.82(6.17)	14.43(5.99)	17.15(6.79)	0.014
7	ExecutionTime_09MeasureTexture_cell20	65.21(29.76)	63.94(29.65)	72.72(29.80)	0.112
8	Group_Index_cell20*	154.40(87.91)	149.39(84.85)	184.18(100.55)	0.032
9	Height_HE_cell20	1138.69(165.30)	1136.55(168.34)	1151.41(147.60)	0.697
10	ImageNumber_cell20*	154.40(87.91)	149.39(84.85)	184.18(100.55)	0.032
11	Mean_IdentifyPrimaryObjects_AreaShape_Area_cell20*	221.49(55.44)	215.71(52.35)	255.84(61.44)	<0.001
12	Mean_IdentifyPrimaryObjects_AreaShape_BoundingBoxArea_cell20*	414.22(121.09)	402.75(115.24)	482.35(133.95)	0.001
13	Mean_IdentifyPrimaryObjects_AreaShape_BoundingBoxMaximum_X_cell20	612.71(94.51)	611.39(96.31)	620.55(83.84)	0.846
14	Mean_IdentifyPrimaryObjects_AreaShape_BoundingBoxMaximum_Y_cell20	574.81(85.45)	573.35(86.71)	583.54(78.18)	0.707
15	Mean_IdentifyPrimaryObjects_AreaShape_BoundingBoxMinimum_X_cell20	592.37(94.60)	591.40(96.46)	598.11(83.74)	0.984
16	Mean_IdentifyPrimaryObjects_AreaShape_BoundingBoxMinimum_Y_cell20	555.99(85.46)	554.67(86.71)	563.86(78.34)	0.723
17	Mean_IdentifyPrimaryObjects_AreaShape_Center_X_cell20	602.04(94.54)	600.90(96.38)	608.83(83.77)	0.923
18	Mean_IdentifyPrimaryObjects_AreaShape_Center_Y_cell20	564.91(85.45)	563.51(86.71)	573.20(78.24)	0.707
19	Mean_IdentifyPrimaryObjects_AreaShape_CentralMoment_0_0_cell20*	221.49(55.44)	215.71(52.35)	255.84(61.44)	<0.001
20	Mean_IdentifyPrimaryObjects_AreaShape_CentralMoment_0_1_cell20	0.00(0.00)	0.00(0.00)	0.00(0.00)	0.2

21	Mean_IdentifyPrimaryObjects_AreaShape_CentralMoment_0_2_cell20*	8849.44(5767.28)	8152.47(5154.15)	12990.23(7376.73)	<0.001
22	Mean_IdentifyPrimaryObjects_AreaShape_CentralMoment_0_3_cell20	222.60(1999.93)	283.99(1924.18)	-142.14(2402.35)	0.34
23	Mean_IdentifyPrimaryObjects_AreaShape_CentralMoment_1_0_cell20	-0.00(0.00)	0.00(0.00)	-0.00(0.00)	0.227
24	Mean_IdentifyPrimaryObjects_AreaShape_CentralMoment_1_1_cell20	-218.89(582.66)	-205.63(564.30)	-297.65(685.99)	0.062
25	Mean_IdentifyPrimaryObjects_AreaShape_CentralMoment_1_2_cell20	-44.47(834.13)	-4.83(807.76)	-279.99(955.97)	0.134
26	Mean_IdentifyPrimaryObjects_AreaShape_CentralMoment_1_3_cell20	-33317.96(123044.05)	-31567.99(111208.90)	-43714.85(179875.12)	0.179
27	Mean_IdentifyPrimaryObjects_AreaShape_CentralMoment_2_0_cell20*	7389.63(4388.64)	7022.60(4149.57)	9570.22(5153.00)	0.003
28	Mean_IdentifyPrimaryObjects_AreaShape_CentralMoment_2_1_cell20	130.24(895.78)	105.49(820.45)	277.27(1260.25)	0.681
29	Mean_IdentifyPrimaryObjects_AreaShape_CentralMoment_2_2_cell20*	388858.18(392172.65)	349155.93(353686.01)	624736.22(516071.72)	<0.001
30	Mean_IdentifyPrimaryObjects_AreaShape_CentralMoment_2_3_cell20	84924.53(553106.81)	62498.36(463801.96)	218162.36(920311.95)	0.464
31	Mean_IdentifyPrimaryObjects_AreaShape_Compactness_cell20	1.79(0.33)	1.79(0.34)	1.84(0.28)	0.255
32	Mean_IdentifyPrimaryObjects_AreaShape_ConvexArea_cell20*	280.02(78.44)	272.34(74.21)	325.66(88.11)	<0.001
33	Mean_IdentifyPrimaryObjects_AreaShape_Eccentricity_cell20	0.76(0.02)	0.76(0.02)	0.77(0.02)	0.074
34	Mean_IdentifyPrimaryObjects_AreaShape_EquivalentDiameter_cell20*	16.10(1.83)	15.92(1.75)	17.22(1.95)	<0.001
35	Mean_IdentifyPrimaryObjects_AreaShape_EulerNumber_cell20*	1.00(0.00)	1.00(0.00)	1.00(0.00)	0.031
36	Mean_IdentifyPrimaryObjects_AreaShape_Extent_cell20	0.58(0.04)	0.58(0.05)	0.57(0.03)	0.391
37	Mean_IdentifyPrimaryObjects_AreaShape_FormFactor_cell20	0.63(0.09)	0.63(0.09)	0.61(0.08)	0.148

38	Mean_IdentifyPrimaryObjects_AreaShape_HuMoment_0_cell20	0.22(0.02)	0.22(0.02)	0.23(0.02)	0.308
39	Mean_IdentifyPrimaryObjects_AreaShape_HuMoment_1_cell20	0.02(0.01)	0.02(0.01)	0.02(0.01)	0.202
40	Mean_IdentifyPrimaryObjects_AreaShape_HuMoment_2_cell20	0.00(0.00)	0.00(0.00)	0.00(0.00)	0.546
41	Mean_IdentifyPrimaryObjects_AreaShape_HuMoment_3_cell20	0.00(0.00)	0.00(0.00)	0.00(0.00)	0.744
42	Mean_IdentifyPrimaryObjects_AreaShape_HuMoment_4_cell20	0.00(0.00)	0.00(0.00)	0.00(0.00)	0.789
43	Mean_IdentifyPrimaryObjects_AreaShape_HuMoment_5_cell20	0.00(0.00)	0.00(0.00)	0.00(0.00)	0.622
44	Mean_IdentifyPrimaryObjects_AreaShape_HuMoment_6_cell20	0.00(0.00)	0.00(0.00)	0.00(0.00)	0.53
45	Mean_IdentifyPrimaryObjects_AreaShape_InertiaTensorEigenvalues_0_cell20*	38.08(11.61)	36.90(10.95)	45.10(13.06)	<0.001
46	Mean_IdentifyPrimaryObjects_AreaShape_InertiaTensorEigenvalues_1_cell20*	12.30(3.10)	12.01(2.95)	14.01(3.44)	0.002
47	Mean_IdentifyPrimaryObjects_AreaShape_InertiaTensor_0_0_cell20*	27.22(8.75)	26.13(8.01)	33.67(10.22)	<0.001
48	Mean_IdentifyPrimaryObjects_AreaShape_InertiaTensor_0_1_cell20	0.71(1.56)	0.65(1.54)	1.02(1.67)	0.206
49	Mean_IdentifyPrimaryObjects_AreaShape_InertiaTensor_1_0_cell20	0.71(1.56)	0.65(1.54)	1.02(1.67)	0.206
50	Mean_IdentifyPrimaryObjects_AreaShape_InertiaTensor_1_1_cell20	23.16(6.50)	22.78(6.36)	25.44(6.95)	0.056
51	Mean_IdentifyPrimaryObjects_AreaShape_MajorAxisLength_cell20*	23.11(3.20)	22.79(3.06)	24.97(3.39)	<0.001
52	Mean_IdentifyPrimaryObjects_AreaShape_MaxFeretDiameter_cell20*	23.25(3.37)	22.93(3.24)	25.19(3.51)	<0.001
53	Mean_IdentifyPrimaryObjects_AreaShape_MaximumRadius_cell20*	5.66(0.57)	5.61(0.55)	5.95(0.57)	<0.001
54	Mean_IdentifyPrimaryObjects_AreaShape_MeanRadius_cell20*	2.36(0.20)	2.35(0.19)	2.46(0.19)	0.002

55	Mean_IdentifyPrimaryObjects_AreaShape_MedianRadius_cell20*	2.11(0.18)	2.10(0.18)	2.20(0.16)	0.001
56	Mean_IdentifyPrimaryObjects_AreaShape_MinFeretDiameter_cell20*	13.48(1.82)	13.31(1.75)	14.45(1.94)	<0.001
57	Mean_IdentifyPrimaryObjects_AreaShape_MinorAxisLength_cell20*	13.28(1.53)	13.14(1.47)	14.10(1.62)	<0.001
58	Mean_IdentifyPrimaryObjects_AreaShape_NormalizedMoment_0_2_cell20*	0.12(0.02)	0.12(0.01)	0.13(0.01)	0.003
59	Mean_IdentifyPrimaryObjects_AreaShape_NormalizedMoment_0_3_cell20*	-0.00(0.00)	0.00(0.00)	-0.00(0.00)	0.039
60	Mean_IdentifyPrimaryObjects_AreaShape_NormalizedMoment_1_1_cell20	-0.00(0.01)	-0.00(0.01)	-0.00(0.01)	0.201
61	Mean_IdentifyPrimaryObjects_AreaShape_NormalizedMoment_1_2_cell20	-0.00(0.00)	-0.00(0.00)	-0.00(0.00)	0.233
62	Mean_IdentifyPrimaryObjects_AreaShape_NormalizedMoment_1_3_cell20	-0.00(0.00)	-0.00(0.00)	-0.00(0.00)	0.127
63	Mean_IdentifyPrimaryObjects_AreaShape_NormalizedMoment_2_0_cell20*	0.10(0.01)	0.10(0.01)	0.10(0.01)	0.01
64	Mean_IdentifyPrimaryObjects_AreaShape_NormalizedMoment_2_1_cell20	0.00(0.00)	0.00(0.00)	0.00(0.00)	0.595
65	Mean_IdentifyPrimaryObjects_AreaShape_NormalizedMoment_2_2_cell20	0.01(0.00)	0.01(0.00)	0.01(0.00)	0.783
66	Mean_IdentifyPrimaryObjects_AreaShape_NormalizedMoment_2_3_cell20	0.00(0.00)	0.00(0.00)	0.00(0.00)	0.827
67	Mean_IdentifyPrimaryObjects_AreaShape_NormalizedMoment_3_0_cell20	-0.00(0.00)	-0.00(0.00)	0.00(0.00)	0.312
68	Mean_IdentifyPrimaryObjects_AreaShape_NormalizedMoment_3_1_cell20	-0.00(0.00)	-0.00(0.00)	-0.00(0.00)	0.263
69	Mean_IdentifyPrimaryObjects_AreaShape_NormalizedMoment_3_2_cell20	-0.00(0.00)	-0.00(0.00)	-0.00(0.00)	0.904
70	Mean_IdentifyPrimaryObjects_AreaShape_NormalizedMoment_3_3_cell20	-0.00(0.00)	-0.00(0.00)	-0.00(0.00)	0.237
71	Mean_IdentifyPrimaryObjects_AreaShape_Orientation_cell20	-2.78(5.66)	-2.60(5.65)	-3.80(5.68)	0.253

72	Mean_IdentifyPrimaryObjects_AreaShape_Perimeter_cell20*	68.19(12.01)	67.18(11.60)	74.18(12.86)	0.002
73	Mean_IdentifyPrimaryObjects_AreaShape_Solidity_cell20	0.82(0.05)	0.82(0.05)	0.82(0.04)	0.501
74	Mean_IdentifyPrimaryObjects_AreaShape_SpatialMoment_0_0_cell20*	221.49(55.44)	215.71(52.35)	255.84(61.44)	<0.001
75	Mean_IdentifyPrimaryObjects_AreaShape_SpatialMoment_0_1_cell20*	2626.58(1174.43)	2491.96(1079.05)	3426.40(1400.09)	<0.001
76	Mean_IdentifyPrimaryObjects_AreaShape_SpatialMoment_0_2_cell20*	46330.53(30646.01)	42664.91(27472.69)	68108.66(38988.09)	<0.001
77	Mean_IdentifyPrimaryObjects_AreaShape_SpatialMoment_0_3_cell20*	1076913.93(955089.23)	960294.91(836347.94)	1769768.09(1287493.21)	<0.001
78	Mean_IdentifyPrimaryObjects_AreaShape_SpatialMoment_1_0_cell20*	2403.02(1023.28)	2310.46(970.53)	2952.90(1164.21)	0.002
79	Mean_IdentifyPrimaryObjects_AreaShape_SpatialMoment_1_1_cell20*	31099.62(19618.50)	29090.22(18020.64)	43037.85(24316.92)	<0.001
80	Mean_IdentifyPrimaryObjects_AreaShape_SpatialMoment_1_2_cell20*	593051.32(500509.35)	538385.61(446939.41)	917829.95(663531.24)	<0.001
81	Mean_IdentifyPrimaryObjects_AreaShape_SpatialMoment_1_3_cell20*	14751510.24(15662746.23)	12983524.03(13638642.42)	25255428.29(21936098.63)	<0.001
82	Mean_IdentifyPrimaryObjects_AreaShape_SpatialMoment_2_0_cell20*	38429.57(23577.02)	36469.02(22298.25)	50077.50(27689.76)	0.003
83	Mean_IdentifyPrimaryObjects_AreaShape_SpatialMoment_2_1_cell20*	537662.19(439251.57)	496232.19(402413.26)	783805.11(560991.41)	0.002
84	Mean_IdentifyPrimaryObjects_AreaShape_SpatialMoment_2_2_cell20*	10948560.12(11198557.54)	9814434.52(9976517.33)	17686600.48(15237151.39)	<0.001
85	Mean_IdentifyPrimaryObjects_AreaShape_SpatialMoment_2_3_cell20*	288136617.37(354693118.48)	250784583.61(308258782.85)	510051641.44(507781161.08)	<0.001
86	Mean_IdentifyPrimaryObjects_AreaShape_Zernike_0_0_cell20	0.52(0.05)	0.52(0.05)	0.51(0.04)	0.078
87	Mean_IdentifyPrimaryObjects_AreaShape_Zernike_1_1_cell20*	0.06(0.00)	0.06(0.00)	0.06(0.00)	0.002
88	Mean_IdentifyPrimaryObjects_AreaShape_Zernike_2_0_cell20	0.14(0.01)	0.14(0.01)	0.14(0.01)	0.064

89	Mean_IdentifyPrimaryObjects_AreaShape_Zernike_2_2_cell20	0.07(0.00)	0.07(0.00)	0.07(0.00)	0.307
90	Mean_IdentifyPrimaryObjects_AreaShape_Zernike_3_1_cell20	0.03(0.00)	0.03(0.00)	0.03(0.00)	0.524
91	Mean_IdentifyPrimaryObjects_AreaShape_Zernike_3_3_cell20	0.02(0.00)	0.02(0.00)	0.02(0.00)	0.076
92	Mean_IdentifyPrimaryObjects_AreaShape_Zernike_4_0_cell20	0.02(0.00)	0.02(0.00)	0.02(0.00)	0.378
93	Mean_IdentifyPrimaryObjects_AreaShape_Zernike_4_2_cell20*	0.03(0.00)	0.03(0.00)	0.03(0.00)	<0.001
94	Mean_IdentifyPrimaryObjects_AreaShape_Zernike_4_4_cell20	0.03(0.00)	0.03(0.00)	0.03(0.00)	0.437
95	Mean_IdentifyPrimaryObjects_AreaShape_Zernike_5_1_cell20	0.02(0.00)	0.02(0.00)	0.02(0.00)	0.648
96	Mean_IdentifyPrimaryObjects_AreaShape_Zernike_5_3_cell20	0.02(0.00)	0.02(0.00)	0.02(0.00)	0.121
97	Mean_IdentifyPrimaryObjects_AreaShape_Zernike_5_5_cell20*	0.01(0.00)	0.01(0.00)	0.01(0.00)	0.035
98	Mean_IdentifyPrimaryObjects_AreaShape_Zernike_6_0_cell20*	0.01(0.00)	0.01(0.00)	0.01(0.00)	0.004
99	Mean_IdentifyPrimaryObjects_AreaShape_Zernike_6_2_cell20	0.01(0.00)	0.01(0.00)	0.01(0.00)	0.494
100	Mean_IdentifyPrimaryObjects_AreaShape_Zernike_6_4_cell20*	0.01(0.00)	0.01(0.00)	0.01(0.00)	0.043
101	Mean_IdentifyPrimaryObjects_AreaShape_Zernike_6_6_cell20	0.02(0.00)	0.02(0.00)	0.02(0.00)	0.216
102	Mean_IdentifyPrimaryObjects_AreaShape_Zernike_7_1_cell20*	0.01(0.00)	0.01(0.00)	0.01(0.00)	0.001
103	Mean_IdentifyPrimaryObjects_AreaShape_Zernike_7_3_cell20	0.01(0.00)	0.01(0.00)	0.01(0.00)	0.22
104	Mean_IdentifyPrimaryObjects_AreaShape_Zernike_7_5_cell20	0.01(0.00)	0.01(0.00)	0.01(0.00)	0.386
105	Mean_IdentifyPrimaryObjects_AreaShape_Zernike_7_7_cell20*	0.01(0.00)	0.01(0.00)	0.01(0.00)	0.004

106	Mean_IdentifyPrimaryObjects_AreaShape_Zernike_8_0_cell20*	0.01(0.00)	0.01(0.00)	0.01(0.00)	0.001
107	Mean_IdentifyPrimaryObjects_AreaShape_Zernike_8_2_cell20*	0.01(0.00)	0.01(0.00)	0.01(0.00)	0.002
108	Mean_IdentifyPrimaryObjects_AreaShape_Zernike_8_4_cell20*	0.01(0.00)	0.01(0.00)	0.01(0.00)	0.002
109	Mean_IdentifyPrimaryObjects_AreaShape_Zernike_8_6_cell20*	0.01(0.00)	0.01(0.00)	0.01(0.00)	0.046
110	Mean_IdentifyPrimaryObjects_AreaShape_Zernike_8_8_cell20*	0.01(0.00)	0.01(0.00)	0.01(0.00)	0.026
111	Mean_IdentifyPrimaryObjects_AreaShape_Zernike_9_1_cell20*	0.01(0.00)	0.01(0.00)	0.01(0.00)	<0.001
112	Mean_IdentifyPrimaryObjects_AreaShape_Zernike_9_3_cell20*	0.01(0.00)	0.01(0.00)	0.01(0.00)	<0.001
113	Mean_IdentifyPrimaryObjects_AreaShape_Zernike_9_5_cell20*	0.01(0.00)	0.01(0.00)	0.01(0.00)	<0.001
114	Mean_IdentifyPrimaryObjects_AreaShape_Zernike_9_7_cell20*	0.01(0.00)	0.01(0.00)	0.01(0.00)	0.002
115	Mean_IdentifyPrimaryObjects_AreaShape_Zernike_9_9_cell20*	0.01(0.00)	0.01(0.00)	0.01(0.00)	0.001
116	Mean_IdentifyPrimaryObjects_Intensity_IntegratedIntensityEdge_Hematoxylin_cell20	24.33(7.27)	24.29(6.67)	24.57(10.25)	0.876
117	Mean_IdentifyPrimaryObjects_Intensity_IntegratedIntensity_Hematoxylin_cell20	116.13(36.63)	114.59(33.64)	125.30(50.58)	0.241
118	Mean_IdentifyPrimaryObjects_Intensity_LowerQuartileIntensity_Hematoxylin_cell20	0.44(0.13)	0.44(0.12)	0.42(0.18)	0.88
119	Mean_IdentifyPrimaryObjects_Intensity_MADIntensity_Hematoxylin_cell20	0.09(0.02)	0.09(0.02)	0.09(0.01)	0.077
120	Mean_IdentifyPrimaryObjects_Intensity_MassDisplacement_Hematoxylin_cell20*	0.36(0.22)	0.35(0.21)	0.42(0.27)	0.018
121	Mean_IdentifyPrimaryObjects_Intensity_MaxIntensityEdge_Hematoxylin_cell20	0.60(0.11)	0.60(0.11)	0.58(0.15)	0.787
122	Mean_IdentifyPrimaryObjects_Intensity_MaxIntensity_Hematoxylin_cell20	0.79(0.10)	0.79(0.09)	0.76(0.12)	0.063

123	Mean_IdentifyPrimaryObjects_Intensity_MeanIntensityEdge_Hematoxylin_cell20	0.42(0.12)	0.43(0.11)	0.40(0.16)	0.821
124	Mean_IdentifyPrimaryObjects_Intensity_MeanIntensity_Hematoxylin_cell20	0.54(0.13)	0.54(0.12)	0.51(0.18)	0.823
125	Mean_IdentifyPrimaryObjects_Intensity_MedianIntensity_Hematoxylin_cell20	0.54(0.14)	0.55(0.12)	0.51(0.19)	0.945
126	Mean_IdentifyPrimaryObjects_Intensity_MinIntensityEdge_Hematoxylin_cell20	0.26(0.13)	0.27(0.13)	0.24(0.13)	0.098
127	Mean_IdentifyPrimaryObjects_Intensity_MinIntensity_Hematoxylin_cell20	0.25(0.14)	0.26(0.14)	0.24(0.13)	0.114
128	Mean_IdentifyPrimaryObjects_Intensity_StdIntensityEdge_Hematoxylin_cell20	0.08(0.03)	0.08(0.03)	0.08(0.02)	0.26
129	Mean_IdentifyPrimaryObjects_Intensity_StdIntensity_Hematoxylin_cell20	0.13(0.03)	0.13(0.03)	0.12(0.02)	0.844
130	Mean_IdentifyPrimaryObjects_Intensity_UpperQuartileIntensity_Hematoxylin_cell20	0.64(0.13)	0.65(0.12)	0.61(0.19)	0.923
131	Mean_IdentifyPrimaryObjects_Location_CenterMassIntensity_X_Hematoxylin_cell20	602.04(94.54)	600.89(96.37)	608.83(83.77)	0.923
132	Mean_IdentifyPrimaryObjects_Location_CenterMassIntensity_Y_Hematoxylin_cell20	564.91(85.45)	563.51(86.71)	573.20(78.23)	0.707
133	Mean_IdentifyPrimaryObjects_Location_Center_X_cell20	602.04(94.54)	600.90(96.38)	608.83(83.77)	0.923
134	Mean_IdentifyPrimaryObjects_Location_Center_Y_cell20	564.91(85.45)	563.51(86.71)	573.20(78.24)	0.707
135	Mean_IdentifyPrimaryObjects_Location_MaxIntensity_X_Hematoxylin_cell20	602.19(94.53)	601.04(96.37)	608.98(83.73)	0.921
136	Mean_IdentifyPrimaryObjects_Location_MaxIntensity_Y_Hematoxylin_cell20	564.75(85.48)	563.35(86.75)	573.06(78.23)	0.699
137	Mean_IdentifyPrimaryObjects_Texture_AngularSecondMoment_Hematoxylin_3_00_256_cell20*	0.01(0.00)	0.01(0.00)	0.00(0.00)	0.011
138	Mean_IdentifyPrimaryObjects_Texture_AngularSecondMoment_Hematoxylin_3_01_256_cell20	0.01(0.00)	0.01(0.00)	0.01(0.00)	0.126
139	Mean_IdentifyPrimaryObjects_Texture_AngularSecondMoment_Hematoxylin_3_02_256_cell20	0.01(0.00)	0.01(0.00)	0.01(0.00)	0.067

140	Mean_IdentifyPrimaryObjects_Texture_AngularSecondMoment_Hematoxylin_3_03_256_cell20	0.01(0.00)	0.01(0.00)	0.01(0.00)	0.066
141	Mean_IdentifyPrimaryObjects_Texture_Contrast_Hematoxylin_3_00_256_cell20	1798.22(970.34)	1841.99(1011.64)	1538.12(624.57)	0.214
142	Mean_IdentifyPrimaryObjects_Texture_Contrast_Hematoxylin_3_01_256_cell20	2256.22(1142.22)	2303.02(1195.14)	1978.15(705.51)	0.387
143	Mean_IdentifyPrimaryObjects_Texture_Contrast_Hematoxylin_3_02_256_cell20	1897.51(1017.32)	1931.58(1065.18)	1695.07(640.23)	0.659
144	Mean_IdentifyPrimaryObjects_Texture_Contrast_Hematoxylin_3_03_256_cell20	2239.79(1123.52)	2288.83(1176.67)	1948.42(673.59)	0.308
145	Mean_IdentifyPrimaryObjects_Texture_Correlation_Hematoxylin_3_00_256_cell20	0.19(0.10)	0.19(0.09)	0.22(0.15)	0.168
146	Mean_IdentifyPrimaryObjects_Texture_Correlation_Hematoxylin_3_01_256_cell20	0.01(0.07)	0.01(0.06)	0.03(0.08)	0.24
147	Mean_IdentifyPrimaryObjects_Texture_Correlation_Hematoxylin_3_02_256_cell20	0.15(0.09)	0.15(0.09)	0.15(0.12)	0.676
148	Mean_IdentifyPrimaryObjects_Texture_Correlation_Hematoxylin_3_03_256_cell20	0.02(0.07)	0.01(0.06)	0.03(0.08)	0.06
149	Mean_IdentifyPrimaryObjects_Texture_DifferenceEntropy_Hematoxylin_3_00_256_cell20	5.49(0.22)	5.49(0.22)	5.49(0.17)	0.902
150	Mean_IdentifyPrimaryObjects_Texture_DifferenceEntropy_Hematoxylin_3_01_256_cell20	5.46(0.20)	5.45(0.21)	5.48(0.17)	0.378
151	Mean_IdentifyPrimaryObjects_Texture_DifferenceEntropy_Hematoxylin_3_02_256_cell20	5.49(0.22)	5.49(0.23)	5.51(0.16)	0.456
152	Mean_IdentifyPrimaryObjects_Texture_DifferenceEntropy_Hematoxylin_3_03_256_cell20	5.47(0.20)	5.47(0.20)	5.50(0.16)	0.363
153	Mean_IdentifyPrimaryObjects_Texture_DifferenceVariance_Hematoxylin_3_00_256_cell20	0.00(0.00)	0.00(0.00)	0.00(0.00)	0.325
154	Mean_IdentifyPrimaryObjects_Texture_DifferenceVariance_Hematoxylin_3_01_256_cell20	0.00(0.00)	0.00(0.00)	0.00(0.00)	0.852
155	Mean_IdentifyPrimaryObjects_Texture_DifferenceVariance_Hematoxylin_3_02_256_cell20	0.00(0.00)	0.00(0.00)	0.00(0.00)	0.601
156	Mean_IdentifyPrimaryObjects_Texture_DifferenceVariance_Hematoxylin_3_03_256_cell20	0.00(0.00)	0.00(0.00)	0.00(0.00)	0.756

157	Mean_IdentifyPrimaryObjects_Texture_Entropy_Hematoxylin_3_00_256_cell20*	7.89(0.31)	7.86(0.30)	8.05(0.32)	0.001
158	Mean_IdentifyPrimaryObjects_Texture_Entropy_Hematoxylin_3_01_256_cell20*	7.60(0.34)	7.57(0.33)	7.75(0.37)	0.004
159	Mean_IdentifyPrimaryObjects_Texture_Entropy_Hematoxylin_3_02_256_cell20*	7.85(0.31)	7.83(0.30)	7.98(0.33)	0.006
160	Mean_IdentifyPrimaryObjects_Texture_Entropy_Hematoxylin_3_03_256_cell20*	7.63(0.34)	7.60(0.33)	7.79(0.35)	0.002
161	Mean_IdentifyPrimaryObjects_Texture_InfoMeas1_Hematoxylin_3_00_256_cell20*	-0.69(0.05)	-0.69(0.05)	-0.67(0.04)	0.028
162	Mean_IdentifyPrimaryObjects_Texture_InfoMeas1_Hematoxylin_3_01_256_cell20*	-0.72(0.05)	-0.73(0.05)	-0.71(0.05)	0.033
163	Mean_IdentifyPrimaryObjects_Texture_InfoMeas1_Hematoxylin_3_02_256_cell20	-0.69(0.05)	-0.69(0.05)	-0.68(0.04)	0.073
164	Mean_IdentifyPrimaryObjects_Texture_InfoMeas1_Hematoxylin_3_03_256_cell20*	-0.72(0.05)	-0.72(0.05)	-0.70(0.04)	0.022
165	Mean_IdentifyPrimaryObjects_Texture_InfoMeas2_Hematoxylin_3_00_256_cell20	1.00(0.00)	1.00(0.00)	1.00(0.00)	0.679
166	Mean_IdentifyPrimaryObjects_Texture_InfoMeas2_Hematoxylin_3_01_256_cell20	1.00(0.00)	1.00(0.00)	1.00(0.00)	0.754
167	Mean_IdentifyPrimaryObjects_Texture_InfoMeas2_Hematoxylin_3_02_256_cell20	1.00(0.00)	1.00(0.00)	1.00(0.00)	0.814
168	Mean_IdentifyPrimaryObjects_Texture_InfoMeas2_Hematoxylin_3_03_256_cell20	1.00(0.00)	1.00(0.00)	1.00(0.00)	0.683
169	Mean_IdentifyPrimaryObjects_Texture_InverseDifferenceMoment_Hematoxylin_3_00_256_cell20	0.04(0.01)	0.04(0.01)	0.05(0.01)	0.437
170	Mean_IdentifyPrimaryObjects_Texture_InverseDifferenceMoment_Hematoxylin_3_01_256_cell20	0.04(0.01)	0.04(0.01)	0.04(0.01)	0.658
171	Mean_IdentifyPrimaryObjects_Texture_InverseDifferenceMoment_Hematoxylin_3_02_256_cell20	0.04(0.01)	0.04(0.01)	0.04(0.01)	0.844
172	Mean_IdentifyPrimaryObjects_Texture_InverseDifferenceMoment_Hematoxylin_3_03_256_cell20	0.04(0.01)	0.04(0.01)	0.04(0.01)	0.576
173	Mean_IdentifyPrimaryObjects_Texture_SumAverage_Hematoxylin_3_00_256_cell20	287.82(62.41)	290.23(57.53)	273.51(85.63)	0.795

174	Mean_IdentifyPrimaryObjects_Texture_SumAverage_Hematoxylin_3_01_256_cell20	285.86(62.63)	288.21(57.68)	271.86(86.20)	0.842
175	Mean_IdentifyPrimaryObjects_Texture_SumAverage_Hematoxylin_3_02_256_cell20	287.50(62.52)	289.91(57.54)	273.17(86.14)	0.819
176	Mean_IdentifyPrimaryObjects_Texture_SumAverage_Hematoxylin_3_03_256_cell20	286.17(62.58)	288.52(57.63)	272.25(86.19)	0.857
177	Mean_IdentifyPrimaryObjects_Texture_SumEntropy_Hematoxylin_3_00_256_cell20*	6.18(0.22)	6.17(0.22)	6.26(0.22)	0.027
178	Mean_IdentifyPrimaryObjects_Texture_SumEntropy_Hematoxylin_3_01_256_cell20	5.95(0.24)	5.94(0.24)	6.02(0.25)	0.059
179	Mean_IdentifyPrimaryObjects_Texture_SumEntropy_Hematoxylin_3_02_256_cell20	6.15(0.22)	6.14(0.22)	6.20(0.23)	0.122
180	Mean_IdentifyPrimaryObjects_Texture_SumEntropy_Hematoxylin_3_03_256_cell20*	5.97(0.24)	5.95(0.24)	6.05(0.24)	0.033
181	Mean_IdentifyPrimaryObjects_Texture_SumVariance_Hematoxylin_3_00_256_cell20	2762.85(1347.24)	2807.20(1415.84)	2499.36(794.96)	0.727
182	Mean_IdentifyPrimaryObjects_Texture_SumVariance_Hematoxylin_3_01_256_cell20	2340.10(1133.51)	2381.67(1192.44)	2093.09(644.61)	0.609
183	Mean_IdentifyPrimaryObjects_Texture_SumVariance_Hematoxylin_3_02_256_cell20	2670.16(1301.85)	2725.09(1366.74)	2343.79(750.41)	0.398
184	Mean_IdentifyPrimaryObjects_Texture_SumVariance_Hematoxylin_3_03_256_cell20	2351.91(1154.15)	2390.24(1213.61)	2124.17(672.65)	0.785
185	Mean_IdentifyPrimaryObjects_Texture_Variance_Hematoxylin_3_00_256_cell20	1140.27(566.28)	1162.30(595.42)	1009.37(321.84)	0.62
186	Mean_IdentifyPrimaryObjects_Texture_Variance_Hematoxylin_3_01_256_cell20	1149.08(562.19)	1171.17(590.74)	1017.81(323.09)	0.564
187	Mean_IdentifyPrimaryObjects_Texture_Variance_Hematoxylin_3_02_256_cell20	1141.92(567.84)	1164.17(597.02)	1009.72(322.68)	0.605
188	Mean_IdentifyPrimaryObjects_Texture_Variance_Hematoxylin_3_03_256_cell20	1147.92(560.41)	1169.77(588.90)	1018.15(322.19)	0.575
189	Median_IdentifyPrimaryObjects_AreaShape_Area_cell20*	187.29(44.00)	182.97(41.60)	213.00(49.51)	<0.001
190	Median_IdentifyPrimaryObjects_AreaShape_BoundingBoxArea_cell20*	333.81(89.80)	325.71(85.75)	381.91(99.27)	0.003

191	Median_IdentifyPrimaryObjects_AreaShape_BoundingBoxMaximum_X_cell20	612.14(98.71)	611.09(101.11)	618.38(84.09)	0.981
192	Median_IdentifyPrimaryObjects_AreaShape_BoundingBoxMaximum_Y_cell20	573.42(88.72)	572.15(90.18)	581.01(80.32)	0.703
193	Median_IdentifyPrimaryObjects_AreaShape_BoundingBoxMinimum_X_cell20	591.67(98.83)	590.87(101.25)	596.43(84.23)	0.918
194	Median_IdentifyPrimaryObjects_AreaShape_BoundingBoxMinimum_Y_cell20	554.50(88.61)	553.37(90.04)	561.22(80.52)	0.736
195	Median_IdentifyPrimaryObjects_AreaShape_Center_X_cell20	601.40(98.73)	600.48(101.14)	606.87(84.14)	0.945
196	Median_IdentifyPrimaryObjects_AreaShape_Center_Y_cell20	563.50(88.65)	562.33(90.12)	570.46(80.27)	0.741
197	Median_IdentifyPrimaryObjects_AreaShape_CentralMoment_0_0_cell20*	187.29(44.00)	182.97(41.60)	213.00(49.51)	<0.001
198	Median_IdentifyPrimaryObjects_AreaShape_CentralMoment_0_1_cell20	-0.00(0.00)	-0.00(0.00)	0.00(0.00)	0.415
199	Median_IdentifyPrimaryObjects_AreaShape_CentralMoment_0_2_cell20*	4034.11(2150.71)	3784.22(1934.48)	5518.74(2737.36)	<0.001
200	Median_IdentifyPrimaryObjects_AreaShape_CentralMoment_0_3_cell20	-3.35(49.73)	-2.67(52.16)	-7.42(31.92)	0.156
201	Median_IdentifyPrimaryObjects_AreaShape_CentralMoment_1_0_cell20	-0.00(0.00)	-0.00(0.00)	-0.00(0.00)	0.424
202	Median_IdentifyPrimaryObjects_AreaShape_CentralMoment_1_1_cell20	-62.46(138.26)	-58.75(138.55)	-84.52(136.42)	0.316
203	Median_IdentifyPrimaryObjects_AreaShape_CentralMoment_1_2_cell20*	-3.63(45.48)	-0.99(45.10)	-19.30(45.24)	0.03
204	Median_IdentifyPrimaryObjects_AreaShape_CentralMoment_1_3_cell20	-1655.55(4009.98)	-1563.73(4003.79)	-2201.10(4063.40)	0.159
205	Median_IdentifyPrimaryObjects_AreaShape_CentralMoment_2_0_cell20*	3448.10(1678.88)	3322.76(1608.48)	4192.77(1909.51)	0.012
206	Median_IdentifyPrimaryObjects_AreaShape_CentralMoment_2_1_cell20	6.19(46.66)	4.62(47.06)	15.53(43.71)	0.145
207	Median_IdentifyPrimaryObjects_AreaShape_CentralMoment_2_2_cell20*	69894.93(56390.03)	64588.07(51749.94)	101423.96(71665.14)	0.002

208	Median_IdentifyPrimaryObjects_AreaShape_CentralMoment_2_3_cell20	66.94(878.88)	33.85(825.19)	263.55(1143.07)	0.334
209	Median_IdentifyPrimaryObjects_AreaShape_Compactness_cell20	1.63(0.28)	1.62(0.28)	1.68(0.25)	0.148
210	Median_IdentifyPrimaryObjects_AreaShape_ConvexArea_cell20*	229.73(59.42)	224.16(56.42)	262.82(66.58)	0.001
211	Median_IdentifyPrimaryObjects_AreaShape_Eccentricity_cell20	0.79(0.03)	0.79(0.03)	0.80(0.02)	0.075
212	Median_IdentifyPrimaryObjects_AreaShape_EquivalentDiameter_cell20*	15.34(1.76)	15.17(1.68)	16.36(1.91)	<0.001
213	Median_IdentifyPrimaryObjects_AreaShape_Extent_cell20	0.58(0.05)	0.58(0.05)	0.58(0.04)	0.338
214	Median_IdentifyPrimaryObjects_AreaShape_FormFactor_cell20	0.63(0.10)	0.63(0.11)	0.61(0.09)	0.163
215	Median_IdentifyPrimaryObjects_AreaShape_HuMoment_0_cell20	0.21(0.02)	0.20(0.02)	0.21(0.01)	0.258
216	Median_IdentifyPrimaryObjects_AreaShape_HuMoment_1_cell20	0.01(0.00)	0.01(0.00)	0.01(0.00)	0.128
217	Median_IdentifyPrimaryObjects_AreaShape_HuMoment_2_cell20	0.00(0.00)	0.00(0.00)	0.00(0.00)	0.26
218	Median_IdentifyPrimaryObjects_AreaShape_HuMoment_3_cell20	0.00(0.00)	0.00(0.00)	0.00(0.00)	0.451
219	Median_IdentifyPrimaryObjects_AreaShape_HuMoment_4_cell20	0.00(0.00)	0.00(0.00)	0.00(0.00)	0.459
220	Median_IdentifyPrimaryObjects_AreaShape_HuMoment_5_cell20	0.00(0.00)	0.00(0.00)	0.00(0.00)	0.354
221	Median_IdentifyPrimaryObjects_AreaShape_HuMoment_6_cell20	-0.00(0.00)	-0.00(0.00)	0.00(0.00)	0.3
222	Median_IdentifyPrimaryObjects_AreaShape_InertiaTensorEigenvalues_0_cell20*	29.73(8.00)	28.97(7.56)	34.25(9.09)	0.003
223	Median_IdentifyPrimaryObjects_AreaShape_InertiaTensorEigenvalues_1_cell20*	9.91(2.29)	9.71(2.18)	11.09(2.62)	0.005
224	Median_IdentifyPrimaryObjects_AreaShape_InertiaTensor_0_0_cell20*	20.28(5.64)	19.59(5.15)	24.36(6.73)	<0.001

225	Median_IdentifyPrimaryObjects_AreaShape_InertiaTensor_0_1_cell20	0.44(0.93)	0.41(0.93)	0.60(0.93)	0.274
226	Median_IdentifyPrimaryObjects_AreaShape_InertiaTensor_1_0_cell20	0.44(0.93)	0.41(0.93)	0.60(0.93)	0.274
227	Median_IdentifyPrimaryObjects_AreaShape_InertiaTensor_1_1_cell20	17.47(4.23)	17.25(4.16)	18.75(4.47)	0.086
228	Median_IdentifyPrimaryObjects_AreaShape_MajorAxisLength_cell20*	21.62(2.86)	21.36(2.74)	23.21(3.11)	<0.001
229	Median_IdentifyPrimaryObjects_AreaShape_MaxFeretDiameter_cell20*	21.76(3.07)	21.47(2.95)	23.44(3.29)	<0.001
230	Median_IdentifyPrimaryObjects_AreaShape_MaximumRadius_cell20*	5.44(0.53)	5.40(0.51)	5.68(0.59)	0.008
231	Median_IdentifyPrimaryObjects_AreaShape_MeanRadius_cell20*	2.30(0.19)	2.28(0.18)	2.38(0.20)	0.003
232	Median_IdentifyPrimaryObjects_AreaShape_MedianRadius_cell20*	2.04(0.11)	2.03(0.11)	2.09(0.12)	0.007
233	Median_IdentifyPrimaryObjects_AreaShape_MinFeretDiameter_cell20*	12.60(1.68)	12.46(1.61)	13.49(1.83)	<0.001
234	Median_IdentifyPrimaryObjects_AreaShape_MinorAxisLength_cell20*	12.51(1.41)	12.39(1.35)	13.23(1.56)	0.001
235	Median_IdentifyPrimaryObjects_AreaShape_NormalizedMoment_0_2_cell20*	0.10(0.01)	0.10(0.01)	0.11(0.01)	<0.001
236	Median_IdentifyPrimaryObjects_AreaShape_NormalizedMoment_0_3_cell20	-0.00(0.00)	-0.00(0.00)	-0.00(0.00)	0.385
237	Median_IdentifyPrimaryObjects_AreaShape_NormalizedMoment_1_1_cell20	-0.00(0.01)	-0.00(0.01)	-0.00(0.01)	0.304
238	Median_IdentifyPrimaryObjects_AreaShape_NormalizedMoment_1_2_cell20*	-0.00(0.00)	0.00(0.00)	-0.00(0.00)	0.009
239	Median_IdentifyPrimaryObjects_AreaShape_NormalizedMoment_1_3_cell20	-0.00(0.00)	-0.00(0.00)	-0.00(0.00)	0.237
240	Median_IdentifyPrimaryObjects_AreaShape_NormalizedMoment_2_0_cell20*	0.09(0.01)	0.09(0.01)	0.09(0.01)	0.001
241	Median_IdentifyPrimaryObjects_AreaShape_NormalizedMoment_2_1_cell20	0.00(0.00)	0.00(0.00)	0.00(0.00)	0.525

242	Median_IdentifyPrimaryObjects_AreaShape_NormalizedMoment_2_2_cell20	0.01(0.00)	0.01(0.00)	0.01(0.00)	0.476
243	Median_IdentifyPrimaryObjects_AreaShape_NormalizedMoment_2_3_cell20	0.00(0.00)	0.00(0.00)	0.00(0.00)	0.508
244	Median_IdentifyPrimaryObjects_AreaShape_NormalizedMoment_3_0_cell20	0.00(0.00)	-0.00(0.00)	0.00(0.00)	0.298
245	Median_IdentifyPrimaryObjects_AreaShape_NormalizedMoment_3_1_cell20	-0.00(0.00)	-0.00(0.00)	-0.00(0.00)	0.395
246	Median_IdentifyPrimaryObjects_AreaShape_NormalizedMoment_3_2_cell20	-0.00(0.00)	-0.00(0.00)	-0.00(0.00)	0.272
247	Median_IdentifyPrimaryObjects_AreaShape_NormalizedMoment_3_3_cell20	-0.00(0.00)	-0.00(0.00)	-0.00(0.00)	0.273
248	Median_IdentifyPrimaryObjects_AreaShape_Orientation_cell20	-6.09(12.28)	-5.67(12.11)	-8.61(13.12)	0.197
249	Median_IdentifyPrimaryObjects_AreaShape_Perimeter_cell20*	62.33(10.53)	61.46(10.16)	67.51(11.36)	0.002
250	Median_IdentifyPrimaryObjects_AreaShape_Solidity_cell20	0.84(0.05)	0.84(0.05)	0.83(0.04)	0.291
251	Median_IdentifyPrimaryObjects_AreaShape_SpatialMoment_0_0_cell20*	187.29(44.00)	182.97(41.60)	213.00(49.51)	<0.001
252	Median_IdentifyPrimaryObjects_AreaShape_SpatialMoment_0_1_cell20*	1714.90(675.14)	1641.69(623.25)	2149.81(807.65)	<0.001
253	Median_IdentifyPrimaryObjects_AreaShape_SpatialMoment_0_2_cell20*	20253.57(11303.71)	18975.60(10245.39)	27846.21(14183.16)	<0.001
254	Median_IdentifyPrimaryObjects_AreaShape_SpatialMoment_0_3_cell20*	274129.16(201033.54)	250925.63(178496.76)	411985.43(266197.59)	<0.001
255	Median_IdentifyPrimaryObjects_AreaShape_SpatialMoment_1_0_cell20*	1580.51(591.26)	1531.17(564.65)	1873.63(666.43)	0.006
256	Median_IdentifyPrimaryObjects_AreaShape_SpatialMoment_1_1_cell20*	14498.26(7811.25)	13766.15(7293.26)	18847.88(9367.30)	0.002
257	Median_IdentifyPrimaryObjects_AreaShape_SpatialMoment_1_2_cell20*	171314.20(121193.37)	159084.48(110495.40)	243973.12(154515.46)	<0.001
258	Median_IdentifyPrimaryObjects_AreaShape_SpatialMoment_1_3_cell20*	2320155.76(2053025.68)	2106312.94(1838648.16)	3590633.74(2735190.18)	<0.001

259	Median_IdentifyPrimaryObjects_AreaShape_SpatialMoment_2_0_cell20*	17181.21(8861.14)	16533.20(8515.82)	21031.15(9980.34)	0.014
260	Median_IdentifyPrimaryObjects_AreaShape_SpatialMoment_2_1_cell20*	158098.73(108549.03)	148979.93(101698.96)	212275.10(131829.98)	0.005
261	Median_IdentifyPrimaryObjects_AreaShape_SpatialMoment_2_2_cell20*	1856263.88(1584420.58)	1709914.64(1451369.32)	2725750.56(2033876.45)	0.002
262	Median_IdentifyPrimaryObjects_AreaShape_SpatialMoment_2_3_cell20*	25012096.05(25851182.32)	22512745.78(23109685.48)	39861177.04(35223271.14)	0.002
263	Median_IdentifyPrimaryObjects_AreaShape_Zernike_0_0_cell20	0.52(0.06)	0.52(0.06)	0.51(0.04)	0.096
264	Median_IdentifyPrimaryObjects_AreaShape_Zernike_1_1_cell20*	0.06(0.00)	0.06(0.00)	0.06(0.00)	0.006
265	Median_IdentifyPrimaryObjects_AreaShape_Zernike_2_0_cell20	0.14(0.01)	0.14(0.01)	0.14(0.01)	0.095
266	Median_IdentifyPrimaryObjects_AreaShape_Zernike_2_2_cell20	0.07(0.01)	0.07(0.01)	0.07(0.00)	0.221
267	Median_IdentifyPrimaryObjects_AreaShape_Zernike_3_1_cell20	0.03(0.00)	0.03(0.00)	0.03(0.00)	0.721
268	Median_IdentifyPrimaryObjects_AreaShape_Zernike_3_3_cell20	0.02(0.00)	0.02(0.00)	0.02(0.00)	0.069
269	Median_IdentifyPrimaryObjects_AreaShape_Zernike_4_0_cell20	0.02(0.00)	0.02(0.00)	0.02(0.00)	0.675
270	Median_IdentifyPrimaryObjects_AreaShape_Zernike_4_2_cell20*	0.03(0.00)	0.03(0.00)	0.03(0.00)	<0.001
271	Median_IdentifyPrimaryObjects_AreaShape_Zernike_4_4_cell20	0.03(0.00)	0.03(0.00)	0.03(0.00)	0.469
272	Median_IdentifyPrimaryObjects_AreaShape_Zernike_5_1_cell20	0.01(0.00)	0.01(0.00)	0.01(0.00)	0.937
273	Median_IdentifyPrimaryObjects_AreaShape_Zernike_5_3_cell20	0.01(0.00)	0.01(0.00)	0.01(0.00)	0.097
274	Median_IdentifyPrimaryObjects_AreaShape_Zernike_5_5_cell20*	0.01(0.00)	0.01(0.00)	0.01(0.00)	0.033
275	Median_IdentifyPrimaryObjects_AreaShape_Zernike_6_0_cell20*	0.01(0.00)	0.01(0.00)	0.01(0.00)	0.017

276	Median_IdentifyPrimaryObjects_AreaShape_Zernike_6_2_cell20	0.01(0.00)	0.01(0.00)	0.01(0.00)	0.667
277	Median_IdentifyPrimaryObjects_AreaShape_Zernike_6_4_cell20*	0.01(0.00)	0.01(0.00)	0.01(0.00)	0.005
278	Median_IdentifyPrimaryObjects_AreaShape_Zernike_6_6_cell20	0.02(0.00)	0.02(0.00)	0.02(0.00)	0.457
279	Median_IdentifyPrimaryObjects_AreaShape_Zernike_7_1_cell20*	0.01(0.00)	0.01(0.00)	0.01(0.00)	0.006
280	Median_IdentifyPrimaryObjects_AreaShape_Zernike_7_3_cell20	0.01(0.00)	0.01(0.00)	0.01(0.00)	0.283
281	Median_IdentifyPrimaryObjects_AreaShape_Zernike_7_5_cell20	0.01(0.00)	0.01(0.00)	0.01(0.00)	0.821
282	Median_IdentifyPrimaryObjects_AreaShape_Zernike_7_7_cell20*	0.01(0.00)	0.01(0.00)	0.01(0.00)	0.001
283	Median_IdentifyPrimaryObjects_AreaShape_Zernike_8_0_cell20*	0.01(0.00)	0.01(0.00)	0.01(0.00)	<0.001
284	Median_IdentifyPrimaryObjects_AreaShape_Zernike_8_2_cell20*	0.01(0.00)	0.01(0.00)	0.01(0.00)	0.003
285	Median_IdentifyPrimaryObjects_AreaShape_Zernike_8_4_cell20*	0.01(0.00)	0.01(0.00)	0.01(0.00)	0.003
286	Median_IdentifyPrimaryObjects_AreaShape_Zernike_8_6_cell20	0.01(0.00)	0.01(0.00)	0.01(0.00)	0.285
287	Median_IdentifyPrimaryObjects_AreaShape_Zernike_8_8_cell20	0.01(0.00)	0.01(0.00)	0.01(0.00)	0.067
288	Median_IdentifyPrimaryObjects_AreaShape_Zernike_9_1_cell20*	0.01(0.00)	0.01(0.00)	0.01(0.00)	0.002
289	Median_IdentifyPrimaryObjects_AreaShape_Zernike_9_3_cell20*	0.01(0.00)	0.01(0.00)	0.01(0.00)	<0.001
290	Median_IdentifyPrimaryObjects_AreaShape_Zernike_9_5_cell20*	0.01(0.00)	0.01(0.00)	0.01(0.00)	0.002
291	Median_IdentifyPrimaryObjects_AreaShape_Zernike_9_7_cell20*	0.01(0.00)	0.01(0.00)	0.01(0.00)	0.003
292	Median_IdentifyPrimaryObjects_AreaShape_Zernike_9_9_cell20*	0.01(0.00)	0.01(0.00)	0.01(0.00)	0.003

293	Median_IdentifyPrimaryObjects_Intensity_IntegratedIntensityEdge_Hematoxylin_cell20	22.34(6.82)	22.32(6.22)	22.51(9.78)	0.91
294	Median_IdentifyPrimaryObjects_Intensity_IntegratedIntensity_Hematoxylin_cell20	99.30(31.26)	98.21(28.23)	105.82(45.35)	0.349
295	Median_IdentifyPrimaryObjects_Intensity_LowerQuartileIntensity_Hematoxylin_cell20	0.44(0.13)	0.44(0.12)	0.41(0.18)	0.891
296	Median_IdentifyPrimaryObjects_Intensity_MADIntensity_Hematoxylin_cell20	0.09(0.02)	0.09(0.02)	0.09(0.01)	0.339
297	Median_IdentifyPrimaryObjects_Intensity_MassDisplacement_Hematoxylin_cell20*	0.28(0.17)	0.27(0.16)	0.33(0.22)	0.019
298	Median_IdentifyPrimaryObjects_Intensity_MaxIntensityEdge_Hematoxylin_cell20	0.59(0.11)	0.60(0.11)	0.57(0.15)	0.8
299	Median_IdentifyPrimaryObjects_Intensity_MaxIntensity_Hematoxylin_cell20	0.80(0.11)	0.80(0.11)	0.77(0.13)	0.062
300	Median_IdentifyPrimaryObjects_Intensity_MeanIntensityEdge_Hematoxylin_cell20	0.42(0.12)	0.43(0.11)	0.40(0.17)	0.876
301	Median_IdentifyPrimaryObjects_Intensity_MeanIntensity_Hematoxylin_cell20	0.54(0.13)	0.54(0.12)	0.51(0.18)	0.863
302	Median_IdentifyPrimaryObjects_Intensity_MedianIntensity_Hematoxylin_cell20	0.53(0.14)	0.54(0.13)	0.51(0.18)	0.999
303	Median_IdentifyPrimaryObjects_Intensity_MinIntensityEdge_Hematoxylin_cell20	0.27(0.14)	0.28(0.14)	0.25(0.15)	0.1
304	Median_IdentifyPrimaryObjects_Intensity_MinIntensity_Hematoxylin_cell20	0.26(0.15)	0.26(0.15)	0.24(0.15)	0.123
305	Median_IdentifyPrimaryObjects_Intensity_StdIntensityEdge_Hematoxylin_cell20	0.07(0.03)	0.07(0.03)	0.08(0.02)	0.199
306	Median_IdentifyPrimaryObjects_Intensity_StdIntensity_Hematoxylin_cell20	0.13(0.03)	0.13(0.03)	0.12(0.02)	0.816
307	Median_IdentifyPrimaryObjects_Intensity_UpperQuartileIntensity_Hematoxylin_cell20	0.64(0.14)	0.64(0.13)	0.61(0.19)	0.958
308	Median_IdentifyPrimaryObjects_Location_CenterMassIntensity_X_Hematoxylin_cell20	601.42(98.70)	600.50(101.11)	606.88(84.04)	0.958
309	Median_IdentifyPrimaryObjects_Location_CenterMassIntensity_Y_Hematoxylin_cell20	563.52(88.66)	562.34(90.13)	570.53(80.26)	0.739

310	Median_IdentifyPrimaryObjects_Location_Center_X_cell20	601.40(98.73)	600.48(101.14)	606.87(84.14)	0.945
311	Median_IdentifyPrimaryObjects_Location_Center_Y_cell20	563.50(88.65)	562.33(90.12)	570.46(80.27)	0.741
312	Median_IdentifyPrimaryObjects_Location_MaxIntensity_X_Hematoxylin_cell20	601.65(98.89)	600.79(101.33)	606.79(84.02)	0.922
313	Median_IdentifyPrimaryObjects_Location_MaxIntensity_Y_Hematoxylin_cell20	563.38(88.61)	562.16(90.08)	570.66(80.14)	0.71
314	Median_IdentifyPrimaryObjects_Texture_AngularSecondMoment_Hematoxylin_3_00_256_cell20*	0.00(0.00)	0.00(0.00)	0.00(0.00)	0.002
315	Median_IdentifyPrimaryObjects_Texture_AngularSecondMoment_Hematoxylin_3_01_256_cell20*	0.01(0.00)	0.01(0.00)	0.00(0.00)	0.008
316	Median_IdentifyPrimaryObjects_Texture_AngularSecondMoment_Hematoxylin_3_02_256_cell20*	0.00(0.00)	0.00(0.00)	0.00(0.00)	0.008
317	Median_IdentifyPrimaryObjects_Texture_AngularSecondMoment_Hematoxylin_3_03_256_cell20*	0.01(0.00)	0.01(0.00)	0.00(0.00)	0.003
318	Median_IdentifyPrimaryObjects_Texture_Contrast_Hematoxylin_3_00_256_cell20	1527.62(862.66)	1564.05(899.14)	1311.22(564.68)	0.274
319	Median_IdentifyPrimaryObjects_Texture_Contrast_Hematoxylin_3_01_256_cell20	1901.41(1004.77)	1937.93(1050.64)	1684.42(639.69)	0.453
320	Median_IdentifyPrimaryObjects_Texture_Contrast_Hematoxylin_3_02_256_cell20	1608.87(904.86)	1636.20(946.63)	1446.48(583.77)	0.758
321	Median_IdentifyPrimaryObjects_Texture_Contrast_Hematoxylin_3_03_256_cell20	1890.53(988.21)	1929.07(1034.90)	1661.58(606.34)	0.37
322	Median_IdentifyPrimaryObjects_Texture_Correlation_Hematoxylin_3_00_256_cell20	0.21(0.11)	0.20(0.09)	0.24(0.16)	0.143
323	Median_IdentifyPrimaryObjects_Texture_Correlation_Hematoxylin_3_01_256_cell20	0.02(0.07)	0.02(0.07)	0.04(0.09)	0.193
324	Median_IdentifyPrimaryObjects_Texture_Correlation_Hematoxylin_3_02_256_cell20	0.17(0.10)	0.17(0.10)	0.16(0.13)	0.752
325	Median_IdentifyPrimaryObjects_Texture_Correlation_Hematoxylin_3_03_256_cell20*	0.02(0.07)	0.02(0.07)	0.05(0.08)	0.036
326	Median_IdentifyPrimaryObjects_Texture_DifferenceEntropy_Hematoxylin_3_00_256_cell20	5.54(0.23)	5.54(0.24)	5.53(0.17)	0.995

327	Median_IdentifyPrimaryObjects_Texture_DifferenceEntropy_Hematoxylin_3_01_256_cell20	5.52(0.22)	5.52(0.23)	5.56(0.16)	0.174
328	Median_IdentifyPrimaryObjects_Texture_DifferenceEntropy_Hematoxylin_3_02_256_cell20	5.54(0.23)	5.53(0.24)	5.56(0.15)	0.263
329	Median_IdentifyPrimaryObjects_Texture_DifferenceEntropy_Hematoxylin_3_03_256_cell20	5.54(0.21)	5.53(0.22)	5.57(0.15)	0.174
330	Median_IdentifyPrimaryObjects_Texture_DifferenceVariance_Hematoxylin_3_00_256_cell20	0.00(0.00)	0.00(0.00)	0.00(0.00)	0.229
331	Median_IdentifyPrimaryObjects_Texture_DifferenceVariance_Hematoxylin_3_01_256_cell20	0.00(0.00)	0.00(0.00)	0.00(0.00)	0.885
332	Median_IdentifyPrimaryObjects_Texture_DifferenceVariance_Hematoxylin_3_02_256_cell20	0.00(0.00)	0.00(0.00)	0.00(0.00)	0.889
333	Median_IdentifyPrimaryObjects_Texture_DifferenceVariance_Hematoxylin_3_03_256_cell20	0.00(0.00)	0.00(0.00)	0.00(0.00)	0.806
334	Median_IdentifyPrimaryObjects_Texture_Entropy_Hematoxylin_3_00_256_cell20*	7.88(0.34)	7.85(0.33)	8.04(0.36)	0.002
335	Median_IdentifyPrimaryObjects_Texture_Entropy_Hematoxylin_3_01_256_cell20*	7.61(0.37)	7.58(0.36)	7.77(0.40)	0.006
336	Median_IdentifyPrimaryObjects_Texture_Entropy_Hematoxylin_3_02_256_cell20*	7.84(0.34)	7.81(0.33)	7.98(0.36)	0.007
337	Median_IdentifyPrimaryObjects_Texture_Entropy_Hematoxylin_3_03_256_cell20*	7.63(0.37)	7.60(0.36)	7.81(0.39)	0.003
338	Median_IdentifyPrimaryObjects_Texture_InfoMeas1_Hematoxylin_3_00_256_cell20*	-0.69(0.05)	-0.70(0.05)	-0.68(0.05)	0.048
339	Median_IdentifyPrimaryObjects_Texture_InfoMeas1_Hematoxylin_3_01_256_cell20	-0.73(0.05)	-0.73(0.05)	-0.72(0.05)	0.066
340	Median_IdentifyPrimaryObjects_Texture_InfoMeas1_Hematoxylin_3_02_256_cell20	-0.70(0.05)	-0.70(0.05)	-0.69(0.05)	0.119
341	Median_IdentifyPrimaryObjects_Texture_InfoMeas1_Hematoxylin_3_03_256_cell20*	-0.73(0.05)	-0.73(0.05)	-0.71(0.05)	0.044
342	Median_IdentifyPrimaryObjects_Texture_InfoMeas2_Hematoxylin_3_00_256_cell20	1.00(0.00)	1.00(0.00)	1.00(0.00)	0.254
343	Median_IdentifyPrimaryObjects_Texture_InfoMeas2_Hematoxylin_3_01_256_cell20	1.00(0.00)	1.00(0.00)	1.00(0.00)	0.237

344	Median_IdentifyPrimaryObjects_Texture_InfoMeas2_Hematoxylin_3_02_256_cell20	1.00(0.00)	1.00(0.00)	1.00(0.00)	0.329
345	Median_IdentifyPrimaryObjects_Texture_InfoMeas2_Hematoxylin_3_03_256_cell20	1.00(0.00)	1.00(0.00)	1.00(0.00)	0.191
346	Median_IdentifyPrimaryObjects_Texture_InverseDifferenceMoment_Hematoxylin_3_00_256_cell20	0.04(0.01)	0.04(0.01)	0.04(0.01)	0.339
347	Median_IdentifyPrimaryObjects_Texture_InverseDifferenceMoment_Hematoxylin_3_01_256_cell20	0.03(0.01)	0.03(0.01)	0.03(0.01)	0.61
348	Median_IdentifyPrimaryObjects_Texture_InverseDifferenceMoment_Hematoxylin_3_02_256_cell20	0.04(0.01)	0.04(0.01)	0.04(0.01)	0.877
349	Median_IdentifyPrimaryObjects_Texture_InverseDifferenceMoment_Hematoxylin_3_03_256_cell20	0.03(0.01)	0.03(0.01)	0.03(0.01)	0.465
350	Median_IdentifyPrimaryObjects_Texture_SumAverage_Hematoxylin_3_00_256_cell20	286.71(64.90)	289.01(60.26)	273.07(87.52)	0.814
351	Median_IdentifyPrimaryObjects_Texture_SumAverage_Hematoxylin_3_01_256_cell20	285.04(65.10)	287.28(60.41)	271.72(87.97)	0.882
352	Median_IdentifyPrimaryObjects_Texture_SumAverage_Hematoxylin_3_02_256_cell20	286.49(65.08)	288.78(60.34)	272.86(88.12)	0.861
353	Median_IdentifyPrimaryObjects_Texture_SumAverage_Hematoxylin_3_03_256_cell20	285.34(65.06)	287.57(60.35)	272.06(87.98)	0.867
354	Median_IdentifyPrimaryObjects_Texture_SumEntropy_Hematoxylin_3_00_256_cell20*	6.22(0.25)	6.20(0.25)	6.31(0.23)	0.018
355	Median_IdentifyPrimaryObjects_Texture_SumEntropy_Hematoxylin_3_01_256_cell20*	6.00(0.27)	5.99(0.27)	6.09(0.26)	0.039
356	Median_IdentifyPrimaryObjects_Texture_SumEntropy_Hematoxylin_3_02_256_cell20	6.18(0.24)	6.17(0.24)	6.25(0.23)	0.075
357	Median_IdentifyPrimaryObjects_Texture_SumEntropy_Hematoxylin_3_03_256_cell20*	6.02(0.27)	6.00(0.27)	6.12(0.25)	0.019
358	Median_IdentifyPrimaryObjects_Texture_SumVariance_Hematoxylin_3_00_256_cell20	2398.58(1224.44)	2432.94(1283.51)	2194.46(769.62)	0.768
359	Median_IdentifyPrimaryObjects_Texture_SumVariance_Hematoxylin_3_01_256_cell20	2014.77(1007.46)	2046.64(1057.11)	1825.43(615.00)	0.701
360	Median_IdentifyPrimaryObjects_Texture_SumVariance_Hematoxylin_3_02_256_cell20	2315.17(1178.49)	2360.16(1234.16)	2047.87(725.80)	0.437

361	Median_IdentifyPrimaryObjects_Texture_SumVariance_Hematoxylin_3_03_256_cell20	2025.31(1029.69)	2054.79(1080.68)	1850.16(632.46)	0.861
362	Median_IdentifyPrimaryObjects_Texture_Variance_Hematoxylin_3_00_256_cell20	1027.16(534.47)	1045.95(561.28)	915.56(316.90)	0.607
363	Median_IdentifyPrimaryObjects_Texture_Variance_Hematoxylin_3_01_256_cell20	1031.34(527.30)	1049.51(553.30)	923.41(318.23)	0.586
364	Median_IdentifyPrimaryObjects_Texture_Variance_Hematoxylin_3_02_256_cell20	1027.11(535.19)	1045.98(562.02)	915.01(317.32)	0.575
365	Median_IdentifyPrimaryObjects_Texture_Variance_Hematoxylin_3_03_256_cell20	1030.56(525.71)	1048.81(551.76)	922.13(315.65)	0.566
366	StDev_IdentifyPrimaryObjects_AreaShape_Area_cell20*	125.94(46.15)	121.09(43.70)	154.76(50.34)	<0.001
367	StDev_IdentifyPrimaryObjects_AreaShape_BoundingBoxArea_cell20*	279.63(109.47)	269.09(103.92)	342.26(121.86)	<0.001
368	StDev_IdentifyPrimaryObjects_AreaShape_BoundingBoxMaximum_X_cell20	335.68(52.14)	334.66(52.99)	341.77(46.98)	0.526
369	StDev_IdentifyPrimaryObjects_AreaShape_BoundingBoxMaximum_Y_cell20	317.67(49.40)	316.78(50.38)	322.94(43.36)	0.725
370	StDev_IdentifyPrimaryObjects_AreaShape_BoundingBoxMinimum_X_cell20	335.70(52.12)	334.67(52.96)	341.78(47.06)	0.526
371	StDev_IdentifyPrimaryObjects_AreaShape_BoundingBoxMinimum_Y_cell20	317.74(49.44)	316.85(50.43)	322.99(43.41)	0.689
372	StDev_IdentifyPrimaryObjects_AreaShape_Center_X_cell20	335.67(52.13)	334.65(52.99)	341.75(47.02)	0.528
373	StDev_IdentifyPrimaryObjects_AreaShape_Center_Y_cell20	317.69(49.42)	316.80(50.41)	322.95(43.39)	0.709
374	StDev_IdentifyPrimaryObjects_AreaShape_CentralMoment_0_0_cell20*	125.94(46.15)	121.09(43.70)	154.76(50.34)	<0.001
375	StDev_IdentifyPrimaryObjects_AreaShape_CentralMoment_0_1_cell20*	0.00(0.00)	0.00(0.00)	0.00(0.00)	<0.001
376	StDev_IdentifyPrimaryObjects_AreaShape_CentralMoment_0_2_cell20*	14512.92(10480.06)	13259.15(9425.53)	21961.82(13199.41)	<0.001
377	StDev_IdentifyPrimaryObjects_AreaShape_CentralMoment_0_3_cell20*	63899.01(63686.14)	57428.49(59589.52)	102341.53(73999.53)	<0.001

378	StDev_IdentifyPrimaryObjects_AreaShape_CentralMoment_1_0_cell20*	0.00(0.00)	0.00(0.00)	0.00(0.00)	0.002
379	StDev_IdentifyPrimaryObjects_AreaShape_CentralMoment_1_1_cell20*	5790.14(4075.05)	5374.50(3751.88)	8259.54(5017.52)	<0.001
380	StDev_IdentifyPrimaryObjects_AreaShape_CentralMoment_1_2_cell20*	35292.62(32478.10)	32062.44(29717.68)	54483.69(41099.77)	<0.001
381	StDev_IdentifyPrimaryObjects_AreaShape_CentralMoment_1_3_cell20*	1764515.49(2128206.03)	1541394.55(1886692.60)	3090116.38(2908156.53)	<0.001
382	StDev_IdentifyPrimaryObjects_AreaShape_CentralMoment_2_0_cell20*	11899.45(7938.85)	11234.05(7492.20)	15852.74(9386.29)	0.003
383	StDev_IdentifyPrimaryObjects_AreaShape_CentralMoment_2_1_cell20*	30899.21(26834.84)	28484.53(24775.49)	45245.20(33786.98)	0.003
384	StDev_IdentifyPrimaryObjects_AreaShape_CentralMoment_2_2_cell20*	1347540.34(1489338.96)	1189842.58(1255593.15)	2284450.57(2266439.25)	0.001
385	StDev_IdentifyPrimaryObjects_AreaShape_CentralMoment_2_3_cell20*	14260755.15(23097171.24)	12314797.11(19472886.42)	25822035.24(36448516.13)	<0.001
386	StDev_IdentifyPrimaryObjects_AreaShape_Compactness_cell20	0.64(0.23)	0.64(0.24)	0.64(0.18)	0.632
387	StDev_IdentifyPrimaryObjects_AreaShape_ConvexArea_cell20*	176.17(68.65)	169.42(64.91)	216.27(77.26)	<0.001
388	StDev_IdentifyPrimaryObjects_AreaShape_Eccentricity_cell20	0.14(0.01)	0.14(0.01)	0.14(0.00)	0.26
389	StDev_IdentifyPrimaryObjects_AreaShape_EquivalentDiameter_cell20*	4.26(1.08)	4.15(1.03)	4.92(1.12)	<0.001
390	StDev_IdentifyPrimaryObjects_AreaShape_EulerNumber_cell20*	0.03(0.03)	0.03(0.03)	0.04(0.03)	0.041
391	StDev_IdentifyPrimaryObjects_AreaShape_Extent_cell20	0.11(0.01)	0.11(0.01)	0.11(0.01)	0.181
392	StDev_IdentifyPrimaryObjects_AreaShape_FormFactor_cell20	0.17(0.01)	0.17(0.02)	0.17(0.01)	0.08
393	StDev_IdentifyPrimaryObjects_AreaShape_HuMoment_0_cell20	0.06(0.02)	0.06(0.02)	0.07(0.02)	0.422
394	StDev_IdentifyPrimaryObjects_AreaShape_HuMoment_1_cell20	0.04(0.02)	0.04(0.02)	0.04(0.02)	0.352

395	StDev_IdentifyPrimaryObjects_AreaShape_HuMoment_2_cell20	0.01(0.01)	0.01(0.01)	0.01(0.01)	0.81
396	StDev_IdentifyPrimaryObjects_AreaShape_HuMoment_3_cell20	0.00(0.00)	0.00(0.00)	0.00(0.01)	0.844
397	StDev_IdentifyPrimaryObjects_AreaShape_HuMoment_4_cell20	0.00(0.00)	0.00(0.00)	0.00(0.00)	0.785
398	StDev_IdentifyPrimaryObjects_AreaShape_HuMoment_5_cell20	0.00(0.00)	0.00(0.00)	0.00(0.01)	0.721
399	StDev_IdentifyPrimaryObjects_AreaShape_HuMoment_6_cell20	0.00(0.00)	0.00(0.00)	0.00(0.00)	0.756
400	StDev_IdentifyPrimaryObjects_AreaShape_InertiaTensorEigenvalues_0_cell20*	29.08(12.22)	27.80(11.46)	36.69(13.94)	<0.001
401	StDev_IdentifyPrimaryObjects_AreaShape_InertiaTensorEigenvalues_1_cell20*	8.34(2.90)	8.06(2.75)	10.00(3.23)	<0.001
402	StDev_IdentifyPrimaryObjects_AreaShape_InertiaTensor_0_0_cell20*	23.26(10.12)	22.01(9.26)	30.64(11.88)	<0.001
403	StDev_IdentifyPrimaryObjects_AreaShape_InertiaTensor_0_1_cell20*	12.33(4.56)	11.93(4.34)	14.75(5.13)	0.002
404	StDev_IdentifyPrimaryObjects_AreaShape_InertiaTensor_1_0_cell20*	12.33(4.56)	11.93(4.34)	14.75(5.13)	0.002
405	StDev_IdentifyPrimaryObjects_AreaShape_InertiaTensor_1_1_cell20*	19.24(7.57)	18.78(7.35)	22.03(8.36)	0.036
406	StDev_IdentifyPrimaryObjects_AreaShape_MajorAxisLength_cell20*	7.82(2.00)	7.61(1.89)	9.07(2.15)	<0.001
407	StDev_IdentifyPrimaryObjects_AreaShape_MaxFeretDiameter_cell20*	8.00(1.99)	7.79(1.90)	9.24(2.07)	<0.001
408	StDev_IdentifyPrimaryObjects_AreaShape_MaximumRadius_cell20*	1.52(0.31)	1.49(0.31)	1.69(0.30)	<0.001
409	StDev_IdentifyPrimaryObjects_AreaShape_MeanRadius_cell20*	0.49(0.10)	0.48(0.10)	0.54(0.09)	<0.001
410	StDev_IdentifyPrimaryObjects_AreaShape_MedianRadius_cell20*	0.44(0.08)	0.44(0.08)	0.48(0.08)	0.004
411	StDev_IdentifyPrimaryObjects_AreaShape_MinFeretDiameter_cell20*	4.56(1.08)	4.46(1.04)	5.14(1.17)	<0.001

412	StDev_IdentifyPrimaryObjects_AreaShape_MinorAxisLength_cell20*	4.15(0.93)	4.07(0.89)	4.67(1.00)	<0.001
413	StDev_IdentifyPrimaryObjects_AreaShape_NormalizedMoment_0_2_cell20*	0.06(0.02)	0.06(0.02)	0.07(0.01)	0.036
414	StDev_IdentifyPrimaryObjects_AreaShape_NormalizedMoment_0_3_cell20	0.02(0.01)	0.02(0.01)	0.02(0.01)	0.061
415	StDev_IdentifyPrimaryObjects_AreaShape_NormalizedMoment_1_1_cell20	0.05(0.01)	0.05(0.01)	0.05(0.01)	0.973
416	StDev_IdentifyPrimaryObjects_AreaShape_NormalizedMoment_1_2_cell20	0.01(0.00)	0.01(0.00)	0.01(0.00)	0.605
417	StDev_IdentifyPrimaryObjects_AreaShape_NormalizedMoment_1_3_cell20	0.02(0.01)	0.02(0.01)	0.02(0.01)	0.378
418	StDev_IdentifyPrimaryObjects_AreaShape_NormalizedMoment_2_0_cell20	0.05(0.01)	0.05(0.01)	0.05(0.01)	0.273
419	StDev_IdentifyPrimaryObjects_AreaShape_NormalizedMoment_2_1_cell20	0.01(0.00)	0.01(0.00)	0.01(0.00)	0.643
420	StDev_IdentifyPrimaryObjects_AreaShape_NormalizedMoment_2_2_cell20	0.01(0.01)	0.01(0.01)	0.01(0.01)	0.997
421	StDev_IdentifyPrimaryObjects_AreaShape_NormalizedMoment_2_3_cell20	0.01(0.00)	0.01(0.00)	0.01(0.00)	0.681
422	StDev_IdentifyPrimaryObjects_AreaShape_NormalizedMoment_3_0_cell20	0.01(0.01)	0.02(0.01)	0.01(0.00)	0.108
423	StDev_IdentifyPrimaryObjects_AreaShape_NormalizedMoment_3_1_cell20	0.02(0.01)	0.02(0.01)	0.02(0.01)	0.41
424	StDev_IdentifyPrimaryObjects_AreaShape_NormalizedMoment_3_2_cell20	0.01(0.00)	0.01(0.00)	0.01(0.00)	0.479
425	StDev_IdentifyPrimaryObjects_AreaShape_NormalizedMoment_3_3_cell20	0.01(0.01)	0.01(0.01)	0.01(0.01)	0.917
426	StDev_IdentifyPrimaryObjects_AreaShape_Orientation_cell20*	55.06(3.60)	54.64(3.44)	57.51(3.60)	<0.001
427	StDev_IdentifyPrimaryObjects_AreaShape_Perimeter_cell20*	26.25(7.88)	25.62(7.60)	30.00(8.56)	0.003
428	StDev_IdentifyPrimaryObjects_AreaShape_Solidity_cell20	0.09(0.02)	0.09(0.02)	0.09(0.01)	0.359

429	StDev_IdentifyPrimaryObjects_AreaShape_SpatialMoment_0_0_cell20*	125.94(46.15)	121.09(43.70)	154.76(50.34)	<0.001
430	StDev_IdentifyPrimaryObjects_AreaShape_SpatialMoment_0_1_cell20*	2714.58(1417.78)	2550.71(1307.66)	3688.18(1660.74)	<0.001
431	StDev_IdentifyPrimaryObjects_AreaShape_SpatialMoment_0_2_cell20*	77704.40(54281.10)	71124.13(49002.59)	116798.96(67206.01)	<0.001
432	StDev_IdentifyPrimaryObjects_AreaShape_SpatialMoment_0_3_cell20*	2804464.79(2512771.36)	2496344.79(2231413.00)	4635060.13(3256045.78)	<0.001
433	StDev_IdentifyPrimaryObjects_AreaShape_SpatialMoment_1_0_cell20*	2463.03(1233.51)	2349.56(1172.04)	3137.20(1386.04)	0.001
434	StDev_IdentifyPrimaryObjects_AreaShape_SpatialMoment_1_1_cell20*	47921.60(31745.04)	44687.19(29401.46)	67137.82(38296.64)	<0.001
435	StDev_IdentifyPrimaryObjects_AreaShape_SpatialMoment_1_2_cell20*	1357314.67(1136719.99)	1234495.63(1028631.32)	2087004.24(1454629.73)	<0.001
436	StDev_IdentifyPrimaryObjects_AreaShape_SpatialMoment_1_3_cell20*	49912718.26(52300318.26)	44204270.85(46468400.20)	83827611.74(70300069.06)	<0.001
437	StDev_IdentifyPrimaryObjects_AreaShape_SpatialMoment_2_0_cell20*	63777.79(42123.86)	60277.33(39941.42)	84574.67(48974.18)	0.003
438	StDev_IdentifyPrimaryObjects_AreaShape_SpatialMoment_2_1_cell20*	1224029.13(1001795.99)	1130529.35(918986.68)	1779527.81(1277253.59)	0.002
439	StDev_IdentifyPrimaryObjects_AreaShape_SpatialMoment_2_2_cell20*	35226158.53(35823256.86)	31620807.48(31107980.83)	56646185.32(51886338.89)	0.001
440	StDev_IdentifyPrimaryObjects_AreaShape_SpatialMoment_2_3_cell20*	1331818042.86(1719343753.54)	1157727236.10(1404860827.39)	2366122247.73(2782512137.98)	0.001
441	StDev_IdentifyPrimaryObjects_AreaShape_Zernike_0_0_cell20	0.15(0.01)	0.15(0.01)	0.15(0.01)	0.115
442	StDev_IdentifyPrimaryObjects_AreaShape_Zernike_1_1_cell20*	0.03(0.00)	0.03(0.00)	0.03(0.00)	0.001
443	StDev_IdentifyPrimaryObjects_AreaShape_Zernike_2_0_cell20	0.04(0.00)	0.04(0.00)	0.03(0.00)	0.304
444	StDev_IdentifyPrimaryObjects_AreaShape_Zernike_2_2_cell20*	0.02(0.00)	0.02(0.00)	0.02(0.00)	0.005
445	StDev_IdentifyPrimaryObjects_AreaShape_Zernike_3_1_cell20	0.02(0.00)	0.02(0.00)	0.02(0.00)	0.821

446	StDev_IdentifyPrimaryObjects_AreaShape_Zernike_3_3_cell20	0.02(0.00)	0.02(0.00)	0.02(0.00)	0.155
447	StDev_IdentifyPrimaryObjects_AreaShape_Zernike_4_0_cell20	0.02(0.00)	0.02(0.00)	0.02(0.00)	0.066
448	StDev_IdentifyPrimaryObjects_AreaShape_Zernike_4_2_cell20*	0.01(0.00)	0.01(0.00)	0.01(0.00)	<0.001
449	StDev_IdentifyPrimaryObjects_AreaShape_Zernike_4_4_cell20*	0.01(0.00)	0.01(0.00)	0.01(0.00)	0.004
450	StDev_IdentifyPrimaryObjects_AreaShape_Zernike_5_1_cell20	0.01(0.00)	0.01(0.00)	0.01(0.00)	0.47
451	StDev_IdentifyPrimaryObjects_AreaShape_Zernike_5_3_cell20	0.01(0.00)	0.01(0.00)	0.01(0.00)	0.2
452	StDev_IdentifyPrimaryObjects_AreaShape_Zernike_5_5_cell20	0.01(0.00)	0.01(0.00)	0.01(0.00)	0.34
453	StDev_IdentifyPrimaryObjects_AreaShape_Zernike_6_0_cell20*	0.01(0.00)	0.01(0.00)	0.01(0.00)	0.004
454	StDev_IdentifyPrimaryObjects_AreaShape_Zernike_6_2_cell20	0.01(0.00)	0.01(0.00)	0.01(0.00)	0.153
455	StDev_IdentifyPrimaryObjects_AreaShape_Zernike_6_4_cell20	0.01(0.00)	0.01(0.00)	0.01(0.00)	0.906
456	StDev_IdentifyPrimaryObjects_AreaShape_Zernike_6_6_cell20*	0.01(0.00)	0.01(0.00)	0.01(0.00)	0.012
457	StDev_IdentifyPrimaryObjects_AreaShape_Zernike_7_1_cell20*	0.01(0.00)	0.01(0.00)	0.01(0.00)	<0.001
458	StDev_IdentifyPrimaryObjects_AreaShape_Zernike_7_3_cell20	0.01(0.00)	0.01(0.00)	0.01(0.00)	0.064
459	StDev_IdentifyPrimaryObjects_AreaShape_Zernike_7_5_cell20	0.01(0.00)	0.01(0.00)	0.01(0.00)	0.096
460	StDev_IdentifyPrimaryObjects_AreaShape_Zernike_7_7_cell20*	0.01(0.00)	0.01(0.00)	0.01(0.00)	0.026
461	StDev_IdentifyPrimaryObjects_AreaShape_Zernike_8_0_cell20*	0.01(0.00)	0.01(0.00)	0.01(0.00)	0.003
462	StDev_IdentifyPrimaryObjects_AreaShape_Zernike_8_2_cell20*	0.01(0.00)	0.01(0.00)	0.01(0.00)	0.004

463	StDev_IdentifyPrimaryObjects_AreaShape_Zernike_8_4_cell20*	0.00(0.00)	0.00(0.00)	0.00(0.00)	0.005
464	StDev_IdentifyPrimaryObjects_AreaShape_Zernike_8_6_cell20*	0.00(0.00)	0.00(0.00)	0.00(0.00)	0.001
465	StDev_IdentifyPrimaryObjects_AreaShape_Zernike_8_8_cell20*	0.00(0.00)	0.00(0.00)	0.00(0.00)	0.002
466	StDev_IdentifyPrimaryObjects_AreaShape_Zernike_9_1_cell20*	0.00(0.00)	0.00(0.00)	0.00(0.00)	<0.001
467	StDev_IdentifyPrimaryObjects_AreaShape_Zernike_9_3_cell20*	0.00(0.00)	0.00(0.00)	0.00(0.00)	0.001
468	StDev_IdentifyPrimaryObjects_AreaShape_Zernike_9_5_cell20*	0.00(0.00)	0.00(0.00)	0.00(0.00)	<0.001
469	StDev_IdentifyPrimaryObjects_AreaShape_Zernike_9_7_cell20*	0.00(0.00)	0.00(0.00)	0.00(0.00)	0.017
470	StDev_IdentifyPrimaryObjects_AreaShape_Zernike_9_9_cell20*	0.00(0.00)	0.00(0.00)	0.00(0.00)	0.011
471	StDev_IdentifyPrimaryObjects_Intensity_IntegratedIntensityEdge_Hematoxylin_cell20	9.20(2.88)	9.12(2.77)	9.68(3.45)	0.291
472	StDev_IdentifyPrimaryObjects_Intensity_IntegratedIntensity_Hematoxylin_cell20	65.17(24.80)	63.63(23.64)	74.32(29.58)	0.055
473	StDev_IdentifyPrimaryObjects_Intensity_LowerQuartileIntensity_Hematoxylin_cell20*	0.04(0.02)	0.04(0.02)	0.05(0.01)	0.034
474	StDev_IdentifyPrimaryObjects_Intensity_MADIntensity_Hematoxylin_cell20	0.04(0.01)	0.04(0.01)	0.03(0.01)	0.384
475	StDev_IdentifyPrimaryObjects_Intensity_MassDisplacement_Hematoxylin_cell20*	0.30(0.18)	0.29(0.17)	0.35(0.20)	0.015
476	StDev_IdentifyPrimaryObjects_Intensity_MaxIntensityEdge_Hematoxylin_cell20	0.07(0.02)	0.07(0.02)	0.07(0.01)	0.218
477	StDev_IdentifyPrimaryObjects_Intensity_MaxIntensity_Hematoxylin_cell20	0.10(0.03)	0.10(0.03)	0.09(0.02)	0.814
478	StDev_IdentifyPrimaryObjects_Intensity_MeanIntensityEdge_Hematoxylin_cell20*	0.03(0.02)	0.03(0.02)	0.04(0.01)	0.017
479	StDev_IdentifyPrimaryObjects_Intensity_MeanIntensity_Hematoxylin_cell20	0.06(0.01)	0.06(0.01)	0.05(0.01)	0.276

480	StDev_IdentifyPrimaryObjects_Intensity_MedianIntensity_Hematoxylin_cell20	0.07(0.02)	0.07(0.02)	0.06(0.02)	0.271
481	StDev_IdentifyPrimaryObjects_Intensity_MinIntensityEdge_Hematoxylin_cell20	0.06(0.03)	0.06(0.03)	0.07(0.04)	0.142
482	StDev_IdentifyPrimaryObjects_Intensity_MinIntensity_Hematoxylin_cell20	0.07(0.03)	0.06(0.03)	0.07(0.04)	0.159
483	StDev_IdentifyPrimaryObjects_Intensity_StdIntensityEdge_Hematoxylin_cell20	0.02(0.01)	0.02(0.01)	0.03(0.01)	0.078
484	StDev_IdentifyPrimaryObjects_Intensity_StdIntensity_Hematoxylin_cell20	0.04(0.01)	0.04(0.01)	0.04(0.01)	0.512
485	StDev_IdentifyPrimaryObjects_Intensity_UpperQuartileIntensity_Hematoxylin_cell20*	0.09(0.02)	0.09(0.02)	0.08(0.02)	0.015
486	StDev_IdentifyPrimaryObjects_Location_CenterMassIntensity_X_Hematoxylin_cell20	335.67(52.13)	334.65(52.98)	341.76(47.02)	0.528
487	StDev_IdentifyPrimaryObjects_Location_CenterMassIntensity_Y_Hematoxylin_cell20	317.69(49.42)	316.81(50.41)	322.96(43.39)	0.709
488	StDev_IdentifyPrimaryObjects_Location_Center_X_cell20	335.67(52.13)	334.65(52.99)	341.75(47.02)	0.528
489	StDev_IdentifyPrimaryObjects_Location_Center_Y_cell20	317.69(49.42)	316.80(50.41)	322.95(43.39)	0.709
490	StDev_IdentifyPrimaryObjects_Location_MaxIntensity_X_Hematoxylin_cell20	335.70(52.14)	334.67(52.99)	341.79(47.04)	0.526
491	StDev_IdentifyPrimaryObjects_Location_MaxIntensity_Y_Hematoxylin_cell20	317.71(49.42)	316.83(50.41)	322.99(43.39)	0.703
492	StDev_IdentifyPrimaryObjects_Texture_AngularSecondMoment_Hematoxylin_3_00_256_cell20	0.00(0.00)	0.00(0.00)	0.00(0.00)	0.148
493	StDev_IdentifyPrimaryObjects_Texture_AngularSecondMoment_Hematoxylin_3_01_256_cell20	0.01(0.00)	0.01(0.00)	0.01(0.01)	0.9
494	StDev_IdentifyPrimaryObjects_Texture_AngularSecondMoment_Hematoxylin_3_02_256_cell20	0.00(0.00)	0.00(0.00)	0.00(0.00)	0.56
495	StDev_IdentifyPrimaryObjects_Texture_AngularSecondMoment_Hematoxylin_3_03_256_cell20	0.01(0.00)	0.01(0.00)	0.01(0.00)	0.44
496	StDev_IdentifyPrimaryObjects_Texture_Contrast_Hematoxylin_3_00_256_cell20	1148.26(605.80)	1175.31(632.67)	987.55(379.89)	0.239

497	StDev_IdentifyPrimaryObjects_Texture_Contrast_Hematoxylin_3_01_256_cell20	1496.16(760.43)	1529.43(798.70)	1298.50(430.51)	0.367
498	StDev_IdentifyPrimaryObjects_Texture_Contrast_Hematoxylin_3_02_256_cell20	1224.85(632.97)	1247.13(664.57)	1092.46(376.88)	0.586
499	StDev_IdentifyPrimaryObjects_Texture_Contrast_Hematoxylin_3_03_256_cell20	1475.82(753.91)	1510.50(792.15)	1269.74(417.56)	0.341
500	StDev_IdentifyPrimaryObjects_Texture_Correlation_Hematoxylin_3_00_256_cell20	0.24(0.03)	0.24(0.02)	0.24(0.03)	0.382
501	StDev_IdentifyPrimaryObjects_Texture_Correlation_Hematoxylin_3_01_256_cell20	0.25(0.03)	0.25(0.03)	0.25(0.04)	0.977
502	StDev_IdentifyPrimaryObjects_Texture_Correlation_Hematoxylin_3_02_256_cell20	0.24(0.03)	0.24(0.03)	0.25(0.04)	0.361
503	StDev_IdentifyPrimaryObjects_Texture_Correlation_Hematoxylin_3_03_256_cell20	0.25(0.03)	0.25(0.03)	0.25(0.04)	0.851
504	StDev_IdentifyPrimaryObjects_Texture_DifferenceEntropy_Hematoxylin_3_00_256_cell20	0.42(0.07)	0.42(0.07)	0.43(0.10)	0.731
505	StDev_IdentifyPrimaryObjects_Texture_DifferenceEntropy_Hematoxylin_3_01_256_cell20	0.53(0.09)	0.53(0.09)	0.54(0.12)	0.699
506	StDev_IdentifyPrimaryObjects_Texture_DifferenceEntropy_Hematoxylin_3_02_256_cell20	0.44(0.08)	0.43(0.07)	0.45(0.11)	0.451
507	StDev_IdentifyPrimaryObjects_Texture_DifferenceEntropy_Hematoxylin_3_03_256_cell20	0.52(0.09)	0.52(0.09)	0.52(0.12)	0.99
508	StDev_IdentifyPrimaryObjects_Texture_DifferenceVariance_Hematoxylin_3_00_256_cell20	0.00(0.00)	0.00(0.00)	0.00(0.00)	0.8
509	StDev_IdentifyPrimaryObjects_Texture_DifferenceVariance_Hematoxylin_3_01_256_cell20	0.00(0.00)	0.00(0.00)	0.00(0.00)	0.77
510	StDev_IdentifyPrimaryObjects_Texture_DifferenceVariance_Hematoxylin_3_02_256_cell20	0.00(0.00)	0.00(0.00)	0.00(0.00)	0.481
511	StDev_IdentifyPrimaryObjects_Texture_DifferenceVariance_Hematoxylin_3_03_256_cell20	0.00(0.00)	0.00(0.00)	0.00(0.00)	0.548
512	StDev_IdentifyPrimaryObjects_Texture_Entropy_Hematoxylin_3_00_256_cell20*	0.83(0.13)	0.82(0.13)	0.90(0.16)	0.002
513	StDev_IdentifyPrimaryObjects_Texture_Entropy_Hematoxylin_3_01_256_cell20*	0.95(0.14)	0.94(0.13)	1.03(0.17)	0.002

514	StDev_IdentifyPrimaryObjects_Texture_Entropy_Hematoxylin_3_02_256_cell20*	0.85(0.14)	0.84(0.13)	0.92(0.16)	<0.001
515	StDev_IdentifyPrimaryObjects_Texture_Entropy_Hematoxylin_3_03_256_cell20*	0.94(0.14)	0.93(0.13)	1.01(0.17)	0.002
516	StDev_IdentifyPrimaryObjects_Texture_InfoMeas1_Hematoxylin_3_00_256_cell20	0.09(0.02)	0.09(0.02)	0.10(0.03)	0.13
517	StDev_IdentifyPrimaryObjects_Texture_InfoMeas1_Hematoxylin_3_01_256_cell20	0.10(0.02)	0.10(0.02)	0.11(0.03)	0.07
518	StDev_IdentifyPrimaryObjects_Texture_InfoMeas1_Hematoxylin_3_02_256_cell20	0.09(0.02)	0.09(0.02)	0.10(0.03)	0.068
519	StDev_IdentifyPrimaryObjects_Texture_InfoMeas1_Hematoxylin_3_03_256_cell20	0.10(0.02)	0.10(0.02)	0.11(0.03)	0.094
520	StDev_IdentifyPrimaryObjects_Texture_InfoMeas2_Hematoxylin_3_00_256_cell20	0.01(0.01)	0.01(0.01)	0.01(0.01)	0.87
521	StDev_IdentifyPrimaryObjects_Texture_InfoMeas2_Hematoxylin_3_01_256_cell20	0.01(0.01)	0.01(0.01)	0.01(0.01)	0.831
522	StDev_IdentifyPrimaryObjects_Texture_InfoMeas2_Hematoxylin_3_02_256_cell20	0.01(0.01)	0.01(0.01)	0.01(0.01)	0.825
523	StDev_IdentifyPrimaryObjects_Texture_InfoMeas2_Hematoxylin_3_03_256_cell20	0.01(0.01)	0.01(0.01)	0.01(0.01)	0.787
524	StDev_IdentifyPrimaryObjects_Texture_InverseDifferenceMoment_Hematoxylin_3_00_256_cell20	0.02(0.01)	0.02(0.01)	0.02(0.01)	0.28
525	StDev_IdentifyPrimaryObjects_Texture_InverseDifferenceMoment_Hematoxylin_3_01_256_cell20	0.02(0.01)	0.02(0.01)	0.02(0.01)	0.153
526	StDev_IdentifyPrimaryObjects_Texture_InverseDifferenceMoment_Hematoxylin_3_02_256_cell20	0.02(0.01)	0.02(0.01)	0.02(0.01)	0.122
527	StDev_IdentifyPrimaryObjects_Texture_InverseDifferenceMoment_Hematoxylin_3_03_256_cell20	0.02(0.01)	0.02(0.01)	0.02(0.01)	0.162
528	StDev_IdentifyPrimaryObjects_Texture_SumAverage_Hematoxylin_3_00_256_cell20	34.40(8.98)	34.92(9.12)	31.33(7.51)	0.105
529	StDev_IdentifyPrimaryObjects_Texture_SumAverage_Hematoxylin_3_01_256_cell20	34.16(8.76)	34.64(8.90)	31.26(7.33)	0.164
530	StDev_IdentifyPrimaryObjects_Texture_SumAverage_Hematoxylin_3_02_256_cell20	34.46(9.09)	34.98(9.23)	31.35(7.61)	0.111

531	StDev_IdentifyPrimaryObjects_Texture_SumAverage_Hematoxylin_3_03_256_cell20	34.18(8.73)	34.68(8.85)	31.22(7.39)	0.137
532	StDev_IdentifyPrimaryObjects_Texture_SumEntropy_Hematoxylin_3_00_256_cell20	0.60(0.09)	0.59(0.09)	0.62(0.09)	0.138
533	StDev_IdentifyPrimaryObjects_Texture_SumEntropy_Hematoxylin_3_01_256_cell20	0.70(0.10)	0.70(0.10)	0.73(0.12)	0.108
534	StDev_IdentifyPrimaryObjects_Texture_SumEntropy_Hematoxylin_3_02_256_cell20	0.61(0.09)	0.60(0.09)	0.63(0.10)	0.062
535	StDev_IdentifyPrimaryObjects_Texture_SumEntropy_Hematoxylin_3_03_256_cell20	0.69(0.10)	0.68(0.10)	0.71(0.11)	0.129
536	StDev_IdentifyPrimaryObjects_Texture_SumVariance_Hematoxylin_3_00_256_cell20	1798.26(907.13)	1830.38(961.77)	1607.46(426.60)	0.859
537	StDev_IdentifyPrimaryObjects_Texture_SumVariance_Hematoxylin_3_01_256_cell20	1537.35(764.73)	1566.59(810.73)	1363.65(353.20)	0.99
538	StDev_IdentifyPrimaryObjects_Texture_SumVariance_Hematoxylin_3_02_256_cell20	1743.94(890.12)	1781.10(943.26)	1523.14(405.22)	0.9
539	StDev_IdentifyPrimaryObjects_Texture_SumVariance_Hematoxylin_3_03_256_cell20	1548.87(774.03)	1575.47(819.59)	1390.86(380.79)	0.87
540	StDev_IdentifyPrimaryObjects_Texture_Variance_Hematoxylin_3_00_256_cell20	656.71(338.46)	670.46(358.12)	575.00(163.02)	0.912
541	StDev_IdentifyPrimaryObjects_Texture_Variance_Hematoxylin_3_01_256_cell20	670.87(340.19)	685.19(359.71)	585.80(165.35)	0.863
542	StDev_IdentifyPrimaryObjects_Texture_Variance_Hematoxylin_3_02_256_cell20	660.70(340.62)	674.68(360.37)	577.69(164.12)	0.906
543	StDev_IdentifyPrimaryObjects_Texture_Variance_Hematoxylin_3_03_256_cell20	668.61(338.97)	682.84(358.42)	584.08(164.92)	0.831
544	Threshold_FinalThreshold_IdentifyPrimaryObjects_cell20	0.38(0.11)	0.39(0.10)	0.37(0.14)	0.88
545	Threshold_OrigThreshold_IdentifyPrimaryObjects_cell20	0.38(0.11)	0.39(0.10)	0.37(0.14)	0.88
546	Threshold_SumOfEntropies_IdentifyPrimaryObjects_cell20	-10.78(1.16)	-10.77(1.13)	-10.85(1.36)	0.713
547	Threshold_WeightedVariance_IdentifyPrimaryObjects_cell20	0.64(0.98)	0.63(0.97)	0.71(1.08)	0.058
548	Width_HE_cell20	1205.03(179.10)	1201.10(181.11)	1228.41(167.22)	0.523
