# Supplementary material for: Integrating computed tomography and biopsy images to predict chemotherapy response in gastric cancer
Source: Front Oncol. 2025 Oct 21;15:1666358. doi: 10.3389/fonc.2025.1666358 (PMC12583031; doi:10.3389/fonc.2025.1666358)
Supplement: Supplementary file 4 [file DataSheet4.pdf]

Radiomic features of the training cohort

	Radiomic Feature names	All cases in the Training Cohort (n=236)	Non pathological complete response cases (n=202)	Pathological complete response cases (n=34)	p-value
1	diagnostics_Image-original_Mean_py	0.34(4.95)	0.29(4.78)	0.58(5.93)	0.757
2	diagnostics_Mask-original_VoxelNum_py	13353.00(8556.68)	13696.30(8859.18)	11313.41(6191.92)	0.245
3	diagnostics_Mask-original_VolumeNum_py	1.12(0.40)	1.13(0.42)	1.06(0.24)	0.301
4	original_shape2D_Elongation_py	0.54(0.18)	0.54(0.18)	0.55(0.16)	0.713
5	original_shape2D_MajorAxisLength_py*	216.77(72.03)	220.03(74.14)	197.42(54.88)	0.04
6	original_shape2D_MaximumDiameter_py*	220.81(70.66)	223.76(73.10)	203.28(51.34)	0.049
7	original_shape2D_MeshSurface_py	13352.46(8556.80)	13695.75(8859.31)	11312.88(6191.93)	0.246
8	original_shape2D_MinorAxisLength_py	113.65(44.99)	114.64(45.75)	107.74(40.27)	0.409
9	original_shape2D_Perimeter_py	765.10(316.71)	776.94(323.54)	694.75(265.99)	0.198
10	original_shape2D_PerimeterSurfaceRatio_py	0.07(0.03)	0.07(0.03)	0.07(0.03)	0.347
11	original_shape2D_PixelSurface_py	13353.00(8556.68)	13696.30(8859.18)	11313.41(6191.92)	0.245
12	original_shape2D_Sphericity_py	0.54(0.11)	0.54(0.11)	0.55(0.11)	0.644
13	original_firstorder_10Percentile_py	-116.11(23.23)	-117.24(17.27)	-109.44(44.40)	0.304
14	original_firstorder_90Percentile_py	96.11(55.38)	93.20(57.86)	113.35(33.00)	0.058
15	original_firstorder_Energy_py	142464680.69(97376386.51)	145157362.99(100992265.63)	126466980.00(71362214.00)	0.521
16	original_firstorder_Entropy_py	2.60(0.32)	2.60(0.31)	2.61(0.33)	0.852
17	original_firstorder_InterquartileRange_py	133.80(88.56)	129.68(88.95)	158.32(83.29)	0.296
18	original_firstorder_Kurtosis_py	3.81(4.83)	4.01(5.11)	2.64(2.41)	0.116
19	original_firstorder_MeanAbsoluteDeviation_py	76.68(27.03)	75.43(27.60)	84.11(22.27)	0.122
20	original_firstorder_Mean_py*	-24.73(41.60)	-28.18(40.12)	-4.21(44.85)	0.003

21	original_firstorder_Median_py*	-41.19(80.60)	-47.92(76.50)	-1.21(93.22)	0.047
22	original_firstorder_RobustMeanAbsoluteDeviation_py	62.50(32.59)	61.15(32.98)	70.47(29.30)	0.148
23	original_firstorder_RootMeanSquared_py	102.77(10.23)	102.27(10.59)	105.75(7.19)	0.106
24	original_firstorder_Skewness_py*	0.72(1.30)	0.82(1.28)	0.08(1.22)	0.002
25	original_firstorder_TotalEnergy_py	142464680.69(97376386.51)	145157362.99(100992265.63)	126466980.00(71362214.00)	0.521
26	original_firstorder_Uniformity_py	0.21(0.05)	0.21(0.05)	0.20(0.05)	0.844
27	original_firstorder_Variance_py	8330.57(3274.69)	8173.65(3353.04)	9262.91(2615.16)	0.133
28	original_glcm_Autocorrelation_py*	59.53(32.42)	56.82(31.42)	75.63(34.06)	0.002
29	original_glcm_ClusterProminence_py	13096.06(4362.63)	12917.16(4519.37)	14158.98(3125.64)	0.24
30	original_glcm_ClusterShade_py	253.81(524.97)	287.71(503.39)	52.37(608.46)	0.094
31	original_glcm_ClusterTendency_py	74.15(30.07)	72.82(30.87)	82.07(23.65)	0.183
32	original_glcm_Contrast_py*	10.01(4.48)	9.69(4.46)	11.92(4.17)	0.007
33	original_glcm_Correlation_py	0.76(0.06)	0.76(0.06)	0.74(0.06)	0.178
34	original_glcm_DifferenceAverage_py*	1.01(0.38)	0.98(0.37)	1.18(0.35)	0.005
35	original_glcm_DifferenceEntropy_py*	1.15(0.21)	1.14(0.21)	1.23(0.20)	0.012
36	original_glcm_DifferenceVariance_py*	8.84(3.71)	8.58(3.71)	10.41(3.35)	0.008
37	original_glcm_Id_py*	0.82(0.05)	0.83(0.05)	0.80(0.05)	0.009
38	original_glcm_Idm_py*	0.82(0.05)	0.82(0.05)	0.80(0.05)	0.009
39	original_glcm_Idmn_py*	0.97(0.01)	0.97(0.01)	0.97(0.01)	0.006
40	original_glcm_Idn_py*	0.95(0.02)	0.96(0.01)	0.95(0.01)	0.005
41	original_glcm_Imc1_py*	-0.54(0.07)	-0.55(0.06)	-0.51(0.07)	0.006
42	original_glcm_Imc2_py	0.97(0.02)	0.97(0.02)	0.96(0.02)	0.053
43	original_glcm_InverseVariance_py	0.24(0.06)	0.24(0.06)	0.25(0.06)	0.088
44	original_glcm_JointAverage_py*	6.26(2.09)	6.08(2.02)	7.29(2.26)	0.003
45	original_glcm_JointEnergy_py	0.12(0.05)	0.13(0.05)	0.12(0.05)	0.257
46	original_glcm_JointEntropy_py	3.77(0.48)	3.76(0.48)	3.86(0.49)	0.257
47	original_glcm_MCC_py*	0.93(0.03)	0.94(0.02)	0.92(0.03)	0.007

48	original_glcm_MaximumProbability_py	0.25(0.08)	0.25(0.08)	0.24(0.08)	0.644
49	original_glcm_SumAverage_py*	12.51(4.18)	12.16(4.03)	14.58(4.51)	0.003
50	original_glcm_SumEntropy_py	3.44(0.40)	3.43(0.40)	3.49(0.41)	0.377
51	original_glcm_SumSquares_py	21.04(8.41)	20.63(8.60)	23.50(6.72)	0.132
52	original_gldm_DependenceEntropy_py	3.86(0.41)	3.85(0.41)	3.92(0.41)	0.389
53	original_gldm_DependenceNonUniformity_py	5819.54(4180.62)	6020.53(4348.92)	4625.40(2747.17)	0.112
54	original_gldm_DependenceNonUniformityNormalized_py*	0.42(0.05)	0.43(0.05)	0.41(0.05)	0.009
55	original_gldm_DependenceVariance_py*	0.43(0.07)	0.43(0.07)	0.46(0.06)	0.026
56	original_gldm_GrayLevelNonUniformity_py	2815.06(2233.28)	2903.81(2325.57)	2287.76(1497.17)	0.238
57	original_gldm_GrayLevelVariance_py	21.16(8.30)	20.76(8.50)	23.54(6.63)	0.139
58	original_gldm_HighGrayLevelEmphasis_py*	65.01(33.22)	62.16(32.29)	81.92(34.11)	0.002
59	original_gldm_LargeDependenceEmphasis_py*	6.08(0.64)	6.12(0.63)	5.83(0.61)	0.016
60	original_gldm_LargeDependenceHighGrayLevelEmphasis_py*	373.94(215.27)	358.25(209.55)	467.15(228.22)	0.006
61	original_gldm_LargeDependenceLowGrayLevelEmphasis_py	1.06(0.41)	1.07(0.41)	0.98(0.41)	0.252
62	original_gldm_LowGrayLevelEmphasis_py	0.19(0.06)	0.19(0.06)	0.19(0.06)	0.774
63	original_gldm_SmallDependenceEmphasis_py*	0.27(0.05)	0.26(0.05)	0.29(0.05)	0.016
64	original_gldm_SmallDependenceHighGrayLevelEmphasis_py*	19.24(8.94)	18.36(8.69)	24.50(8.72)	<0.001
65	original_gldm_SmallDependenceLowGrayLevelEmphasis_py	0.06(0.02)	0.06(0.02)	0.07(0.02)	0.141
66	original_glrlm_GrayLevelNonUniformity_py	689.95(435.93)	695.63(445.16)	656.20(380.65)	0.819

67	original_glrlm_GrayLevelNonUniformityNormalized_py	0.17(0.03)	0.17(0.03)	0.17(0.03)	0.856
68	original_glrlm_GrayLevelVariance_py	25.19(7.65)	24.86(7.90)	27.15(5.63)	0.259
69	original_glrlm_HighGrayLevelRunEmphasis_py*	71.76(28.18)	69.45(27.71)	85.44(27.40)	0.002
70	original_glrlm_LongRunEmphasis_py*	23.70(17.58)	24.47(17.91)	19.12(14.95)	0.01
71	original_glrlm_LongRunHighGrayLevelEmphasis_py	1256.53(1055.01)	1229.92(1034.24)	1414.63(1175.13)	0.236
72	original_glrlm_LongRunLowGrayLevelEmphasis_py*	3.94(4.01)	4.09(4.12)	3.03(3.13)	0.017
73	original_glrlm_LowGrayLevelRunEmphasis_py	0.24(0.07)	0.24(0.07)	0.24(0.07)	0.844
74	original_glrlm_RunEntropy_py*	5.45(0.36)	5.47(0.35)	5.29(0.37)	0.007
75	original_glrlm_RunLengthNonUniformity_py	809.33(531.33)	791.81(500.66)	913.44(686.01)	0.542
76	original_glrlm_RunLengthNonUniformityNormalized_py*	0.20(0.06)	0.20(0.06)	0.22(0.06)	0.023
77	original_glrlm_RunPercentage_py*	0.31(0.07)	0.31(0.07)	0.34(0.07)	0.016
78	original_glrlm_RunVariance_py*	11.33(10.01)	11.75(10.31)	8.81(7.62)	0.011
79	original_glrlm_ShortRunEmphasis_py*	0.41(0.09)	0.41(0.09)	0.44(0.09)	0.022
80	original_glrlm_ShortRunHighGrayLevelEmphasis_py*	33.34(12.13)	32.22(11.97)	39.97(11.09)	<0.001
81	original_glrlm_ShortRunLowGrayLevelEmphasis_py	0.12(0.04)	0.12(0.04)	0.13(0.04)	0.204
82	original_glszm_GrayLevelNonUniformity_py	689.95(435.93)	695.63(445.16)	656.20(380.65)	0.819
83	original_glszm_GrayLevelNonUniformityNormalized_py	0.17(0.03)	0.17(0.03)	0.17(0.03)	0.856
84	original_glszm_GrayLevelVariance_py	25.19(7.65)	24.86(7.90)	27.15(5.63)	0.259
85	original_glszm_HighGrayLevelZoneEmphasis_py*	71.76(28.18)	69.45(27.71)	85.44(27.40)	0.002

86	original_glszm_LargeAreaEmphasis_py*	23.70(17.58)	24.47(17.91)	19.12(14.95)	0.01
87	original_glszm_LargeAreaHighGrayLevelEmphasis_py	1256.53(1055.01)	1229.92(1034.24)	1414.63(1175.13)	0.236
88	original_glszm_LargeAreaLowGrayLevelEmphasis_py*	3.94(4.01)	4.09(4.12)	3.03(3.13)	0.017
89	original_glszm_LowGrayLevelZoneEmphasis_py	0.24(0.07)	0.24(0.07)	0.24(0.07)	0.844
90	original_glszm_SizeZoneNonUniformity_py	809.33(531.33)	791.81(500.66)	913.44(686.01)	0.542
91	original_glszm_SizeZoneNonUniformityNormalized_py*	0.20(0.06)	0.20(0.06)	0.22(0.06)	0.023
92	original_glszm_SmallAreaEmphasis_py*	0.41(0.09)	0.41(0.09)	0.44(0.09)	0.022
93	original_glszm_SmallAreaHighGrayLevelEmphasis_py*	33.34(12.13)	32.22(11.97)	39.97(11.09)	<0.001
94	original_glszm_SmallAreaLowGrayLevelEmphasis_py	0.12(0.04)	0.12(0.04)	0.13(0.04)	0.204
95	original_glszm_ZoneEntropy_py*	5.45(0.36)	5.47(0.35)	5.29(0.37)	0.007
96	original_glszm_ZonePercentage_py*	0.31(0.07)	0.31(0.07)	0.34(0.07)	0.016
97	original_glszm_ZoneVariance_py*	11.33(10.01)	11.75(10.31)	8.81(7.62)	0.011
98	original_ngtdm_Busyness_py	21.58(16.42)	21.83(16.61)	20.09(15.40)	0.451
99	original_ngtdm_Coarseness_py	0.00(0.00)	0.00(0.00)	0.00(0.00)	0.483
100	original_ngtdm_Complexity_py*	95.07(41.96)	92.64(41.88)	109.52(40.01)	0.03
101	original_ngtdm_Contrast_py*	0.45(0.38)	0.43(0.35)	0.58(0.48)	0.049
102	original_ngtdm_Strength_py	0.12(0.08)	0.12(0.08)	0.12(0.09)	0.825
103	log-sigma-1-mm-3D_firstorder_10Percentile_py*	-35.30(16.35)	-34.24(16.74)	-41.62(12.21)	0.018
104	log-sigma-1-mm-3D_firstorder_90Percentile_py*	38.19(13.78)	37.49(13.64)	42.35(14.06)	0.015
105	log-sigma-1-mm-3D_firstorder_Energy_py	12762640.19(9389664.89)	12645328.25(9477862.92)	13459611.13(8950810.95)	0.448
106	log-sigma-1-mm-3D_firstorder_Entropy_py*	2.49(0.43)	2.46(0.44)	2.67(0.36)	0.009

107	log-sigma-1-mm-3D_firstorder_InterquartileRange_py*	19.65(15.25)	18.55(14.74)	26.16(16.77)	0.003
108	log-sigma-1-mm-3D_firstorder_Kurtosis_py*	6.93(6.81)	7.23(7.26)	5.20(2.23)	0.016
109	log-sigma-1-mm-3D_firstorder_Maximum_py*	146.69(22.92)	145.44(23.37)	154.11(18.70)	0.041
110	log-sigma-1-mm-3D_firstorder_MeanAbsoluteDeviation_py*	19.82(7.08)	19.30(7.04)	22.89(6.62)	0.006
111	log-sigma-1-mm-3D_firstorder_Mean_py	0.67(0.62)	0.68(0.62)	0.64(0.60)	0.605
112	log-sigma-1-mm-3D_firstorder_Median_py	0.13(0.85)	0.16(0.82)	-0.01(1.05)	0.109
113	log-sigma-1-mm-3D_firstorder_Minimum_py	-154.46(18.75)	-154.65(19.24)	-153.34(15.73)	0.381
114	log-sigma-1-mm-3D_firstorder_Range_py	301.15(31.99)	300.09(32.80)	307.45(26.21)	0.215
115	log-sigma-1-mm-3D_firstorder_RobustMeanAbsoluteDeviation_py*	10.21(5.80)	9.80(5.69)	12.65(5.97)	0.008
116	log-sigma-1-mm-3D_firstorder_RootMeanSquared_py*	30.85(7.47)	30.30(7.53)	34.11(6.28)	0.006
117	log-sigma-1-mm-3D_firstorder_Skewness_py*	-0.19(0.63)	-0.23(0.63)	0.04(0.61)	0.007
118	log-sigma-1-mm-3D_firstorder_TotalEnergy_py	12762640.19(9389664.89)	12645328.25(9477862.92)	13459611.13(8950810.95)	0.448
119	log-sigma-1-mm-3D_firstorder_Uniformity_py*	0.25(0.08)	0.25(0.08)	0.22(0.06)	0.011
120	log-sigma-1-mm-3D_firstorder_Variance_py*	1006.66(441.17)	973.94(437.86)	1201.02(415.63)	0.005
121	log-sigma-1-mm-3D_glcm_Autocorrelation_py	78.59(15.58)	78.96(15.78)	76.43(14.33)	0.539
122	log-sigma-1-mm-3D_glcm_ClusterProminence_py*	387.73(195.35)	372.53(192.38)	478.06(191.09)	0.003

123	log-sigma-1-mm-3D_glcm_ClusterShade_py	-2.68(11.99)	-3.27(11.51)	0.80(14.18)	0.052
124	log-sigma-1-mm-3D_glcm_ClusterTendency_py*	8.69(3.57)	8.43(3.54)	10.27(3.40)	0.005
125	log-sigma-1-mm-3D_glcm_Contrast_py*	1.93(0.82)	1.87(0.81)	2.30(0.78)	0.005
126	log-sigma-1-mm-3D_glcm_Correlation_py	0.64(0.03)	0.64(0.03)	0.63(0.04)	0.556
127	log-sigma-1-mm-3D_glcm_DifferenceAverage_py*	0.73(0.24)	0.72(0.24)	0.84(0.22)	0.007
128	log-sigma-1-mm-3D_glcm_DifferenceEntropy_py*	1.62(0.31)	1.60(0.31)	1.75(0.25)	0.007
129	log-sigma-1-mm-3D_glcm_DifferenceVariance_py*	1.34(0.47)	1.30(0.47)	1.55(0.41)	0.004
130	log-sigma-1-mm-3D_glcm_Id_py*	0.75(0.07)	0.75(0.07)	0.72(0.06)	0.007
131	log-sigma-1-mm-3D_glcm_Idm_py*	0.73(0.08)	0.74(0.08)	0.70(0.07)	0.007
132	log-sigma-1-mm-3D_glcm_Idmn_py*	0.99(0.00)	0.99(0.00)	0.99(0.00)	0.002
133	log-sigma-1-mm-3D_glcm_Idn_py*	0.96(0.01)	0.96(0.01)	0.96(0.01)	0.003
134	log-sigma-1-mm-3D_glcm_Imc1_py*	-0.31(0.05)	-0.31(0.05)	-0.29(0.05)	0.008
135	log-sigma-1-mm-3D_glcm_Imc2_py	0.88(0.03)	0.88(0.03)	0.88(0.02)	0.915
136	log-sigma-1-mm-3D_glcm_InverseVariance_py*	0.32(0.05)	0.31(0.05)	0.34(0.05)	0.007
137	log-sigma-1-mm-3D_glcm_JointAverage_py	8.72(0.95)	8.74(0.97)	8.59(0.85)	0.169
138	log-sigma-1-mm-3D_glcm_JointEnergy_py*	0.14(0.07)	0.14(0.07)	0.11(0.05)	0.009
139	log-sigma-1-mm-3D_glcm_JointEntropy_py*	4.22(0.84)	4.16(0.85)	4.59(0.72)	0.006
140	log-sigma-1-mm-3D_glcm_MCC_py*	0.76(0.05)	0.76(0.05)	0.73(0.05)	0.006
141	log-sigma-1-mm-3D_glcm_MaximumProbability_py*	0.25(0.08)	0.26(0.08)	0.22(0.07)	0.017
142	log-sigma-1-mm-3D_glcm_SumAverage_py	17.44(1.90)	17.48(1.93)	17.17(1.71)	0.169
143	log-sigma-1-mm-3D_glcm_SumEntropy_py*	3.21(0.51)	3.17(0.52)	3.43(0.42)	0.007

144	log-sigma-1-mm-3D_glcm_SumSquares_py*	2.66(1.09)	2.58(1.08)	3.14(1.03)	0.005
145	log-sigma-1-mm-3D_gldm_DependenceEntropy_py*	3.79(0.50)	3.75(0.51)	4.00(0.40)	0.008
146	log-sigma-1-mm-3D_gldm_DependenceNonUniformity_py	4949.16(3375.33)	5101.60(3501.83)	4043.53(2334.89)	0.158
147	log-sigma-1-mm-3D_gldm_DependenceNonUniformityNormalized_py	0.37(0.04)	0.37(0.04)	0.36(0.02)	0.163
148	log-sigma-1-mm-3D_gldm_DependenceVariance_py	0.56(0.06)	0.56(0.06)	0.57(0.04)	0.413
149	log-sigma-1-mm-3D_gldm_GrayLevelNonUniformity_py*	3451.01(2844.52)	3615.76(2967.14)	2472.22(1686.05)	0.042
150	log-sigma-1-mm-3D_gldm_GrayLevelVariance_py*	2.67(1.08)	2.59(1.07)	3.14(1.02)	0.005
151	log-sigma-1-mm-3D_gldm_HighGrayLevelEmphasis_py	79.56(15.72)	79.89(15.93)	77.57(14.48)	0.699
152	log-sigma-1-mm-3D_gldm_LargeDependenceEmphasis_py*	5.05(0.86)	5.11(0.86)	4.68(0.75)	0.007
153	log-sigma-1-mm-3D_gldm_LargeDependenceHighGrayLevelEmphasis_py*	389.77(90.17)	396.20(91.36)	351.54(72.80)	0.007
154	log-sigma-1-mm-3D_gldm_LargeDependenceLowGrayLevelEmphasis_py	0.08(0.04)	0.08(0.04)	0.07(0.02)	0.787
155	log-sigma-1-mm-3D_gldm_LowGrayLevelEmphasis_py*	0.02(0.01)	0.02(0.01)	0.02(0.00)	0.041
156	log-sigma-1-mm-3D_gldm_SmallDependenceEmphasis_py*	0.39(0.09)	0.38(0.09)	0.43(0.08)	0.007

157	log-sigma-1-mm-3D_gldm_SmallDependenceHighGrayLevelEmphasis_py	32.29(10.47)	31.92(10.55)	34.45(9.82)	0.193
158	log-sigma-1-mm-3D_gldm_SmallDependenceLowGrayLevelEmphasis_py*	0.01(0.00)	0.01(0.00)	0.01(0.00)	0.008
159	log-sigma-1-mm-3D_glrlm_GrayLevelNonUniformity_py	897.78(562.97)	919.02(583.39)	771.56(406.00)	0.293
160	log-sigma-1-mm-3D_glrlm_GrayLevelNonUniformityNormalized_py*	0.16(0.05)	0.17(0.05)	0.15(0.03)	0.011
161	log-sigma-1-mm-3D_glrlm_GrayLevelVariance_py*	4.23(1.09)	4.15(1.10)	4.70(0.89)	0.006
162	log-sigma-1-mm-3D_glrlm_HighGrayLevelRunEmphasis_py	81.62(15.44)	81.96(15.66)	79.61(14.13)	0.649
163	log-sigma-1-mm-3D_glrlm_LongRunEmphasis_py*	12.24(7.97)	12.75(8.21)	9.18(5.55)	0.006
164	log-sigma-1-mm-3D_glrlm_LongRunHighGrayLevelEmphasis_py*	905.04(545.57)	944.56(558.29)	670.26(393.45)	0.002
165	log-sigma-1-mm-3D_glrlm_LongRunLowGrayLevelEmphasis_py	0.20(0.24)	0.21(0.25)	0.14(0.09)	0.055
166	log-sigma-1-mm-3D_glrlm_LowGrayLevelRunEmphasis_py	0.02(0.01)	0.02(0.01)	0.02(0.01)	0.097
167	log-sigma-1-mm-3D_glrlm_RunEntropy_py*	4.92(0.17)	4.93(0.17)	4.87(0.14)	0.045
168	log-sigma-1-mm-3D_glrlm_RunLengthNonUniformity_py	2114.67(1555.19)	2091.19(1566.09)	2254.20(1503.73)	0.407

169	log-sigma-1-mm-3D_glrlm_RunLengthNonUniformityNormalized_py*	0.36(0.09)	0.35(0.10)	0.40(0.08)	0.01
170	log-sigma-1-mm-3D_glrlm_RunPercentage_py*	0.45(0.10)	0.44(0.11)	0.49(0.09)	0.007
171	log-sigma-1-mm-3D_glrlm_RunVariance_py*	6.03(4.05)	6.29(4.13)	4.46(3.18)	0.005
172	log-sigma-1-mm-3D_glrlm_ShortRunEmphasis_py*	0.61(0.10)	0.60(0.10)	0.65(0.07)	0.009
173	log-sigma-1-mm-3D_glrlm_ShortRunHighGrayLevelEmphasis_py	51.07(13.37)	50.77(13.59)	52.88(11.98)	0.396
174	log-sigma-1-mm-3D_glrlm_ShortRunLowGrayLevelEmphasis_py*	0.01(0.00)	0.01(0.00)	0.01(0.00)	0.01
175	log-sigma-1-mm-3D_glszm_GrayLevelNonUniformity_py	897.78(562.97)	919.02(583.39)	771.56(406.00)	0.293
176	log-sigma-1-mm-3D_glszm_GrayLevelNonUniformityNormalized_py*	0.16(0.05)	0.17(0.05)	0.15(0.03)	0.011
177	log-sigma-1-mm-3D_glszm_GrayLevelVariance_py*	4.23(1.09)	4.15(1.10)	4.70(0.89)	0.006
178	log-sigma-1-mm-3D_glszm_HighGrayLevelZoneEmphasis_py	81.62(15.44)	81.96(15.66)	79.61(14.13)	0.649
179	log-sigma-1-mm-3D_glszm_LargeAreaEmphasis_py*	12.24(7.97)	12.75(8.21)	9.18(5.55)	0.006
180	log-sigma-1-mm-3D_glszm_LargeAreaHighGrayLevelEmphasis_py*	905.04(545.57)	944.56(558.29)	670.26(393.45)	0.002
181	log-sigma-1-mm-3D_glszm_LargeAreaLowGrayLevelEmphasis_py	0.20(0.24)	0.21(0.25)	0.14(0.09)	0.055

182	log-sigma-1-mm-3D_glszm_LowGrayLevelZoneEmphasis_py	0.02(0.01)	0.02(0.01)	0.02(0.01)	0.097
183	log-sigma-1-mm-3D_glszm_SizeZoneNonUniformity_py	2114.67(1555.19)	2091.19(1566.09)	2254.20(1503.73)	0.407
184	log-sigma-1-mm-3D_glszm_SizeZoneNonUniformityNormalized_py*	0.36(0.09)	0.35(0.10)	0.40(0.08)	0.01
185	log-sigma-1-mm-3D_glszm_SmallAreaEmphasis_py*	0.61(0.10)	0.60(0.10)	0.65(0.07)	0.009
186	log-sigma-1-mm-3D_glszm_SmallAreaHighGrayLevelEmphasis_py	51.07(13.37)	50.77(13.59)	52.88(11.98)	0.396
187	log-sigma-1-mm-3D_glszm_SmallAreaLowGrayLevelEmphasis_py*	0.01(0.00)	0.01(0.00)	0.01(0.00)	0.01
188	log-sigma-1-mm-3D_glszm_ZoneEntropy_py*	4.92(0.17)	4.93(0.17)	4.87(0.14)	0.045
189	log-sigma-1-mm-3D_glszm_ZonePercentage_py*	0.45(0.10)	0.44(0.11)	0.49(0.09)	0.007
190	log-sigma-1-mm-3D_glszm_ZoneVariance_py*	6.03(4.05)	6.29(4.13)	4.46(3.18)	0.005
191	log-sigma-1-mm-3D_ngtdm_Busyness_py	5.17(3.26)	5.27(3.40)	4.59(2.19)	0.432
192	log-sigma-1-mm-3D_ngtdm_Coarseness_py	0.00(0.00)	0.00(0.00)	0.00(0.00)	0.316
193	log-sigma-1-mm-3D_ngtdm_Complexity_py*	64.03(25.13)	62.69(25.17)	72.00(23.70)	0.045
194	log-sigma-1-mm-3D_ngtdm_Contrast_py*	0.01(0.01)	0.01(0.01)	0.02(0.01)	0.004
195	log-sigma-1-mm-3D_ngtdm_Strength_py	0.15(0.08)	0.15(0.08)	0.16(0.09)	0.596
196	log-sigma-2-mm-3D_firstorder_10Percentile_py*	-41.30(15.80)	-40.46(16.22)	-46.26(12.04)	0.041

197	log-sigma-2-mm-3D_firstorder_90Percentile_py*	46.22(12.88)	45.47(12.64)	50.72(13.60)	0.028
198	log-sigma-2-mm-3D_firstorder_Energy_py	17255780.65(13482827.13)	17284021.30(13943203.57)	17087997.91(10505036.48)	0.675
199	log-sigma-2-mm-3D_firstorder_Entropy_py*	2.74(0.42)	2.71(0.43)	2.88(0.35)	0.03
200	log-sigma-2-mm-3D_firstorder_InterquartileRange_py*	36.03(17.29)	35.01(16.99)	42.07(18.05)	0.027
201	log-sigma-2-mm-3D_firstorder_Kurtosis_py*	5.22(4.01)	5.38(4.24)	4.25(2.01)	0.025
202	log-sigma-2-mm-3D_firstorder_Maximum_py	136.33(20.81)	135.66(21.43)	140.31(16.30)	0.352
203	log-sigma-2-mm-3D_firstorder_MeanAbsoluteDeviation_py*	25.79(7.94)	25.30(7.94)	28.72(7.42)	0.02
204	log-sigma-2-mm-3D_firstorder_Mean_py	2.16(1.83)	2.20(1.82)	1.93(1.86)	0.28
205	log-sigma-2-mm-3D_firstorder_Median_py	1.85(3.95)	2.00(3.82)	0.95(4.59)	0.143
206	log-sigma-2-mm-3D_firstorder_Minimum_py*	-145.16(18.35)	-146.09(18.26)	-139.63(18.22)	0.017
207	log-sigma-2-mm-3D_firstorder_Range_py	281.49(29.30)	281.75(30.31)	279.94(22.62)	0.503
208	log-sigma-2-mm-3D_firstorder_RobustMeanAbsoluteDeviation_py*	15.90(6.66)	15.49(6.56)	18.32(6.81)	0.022
209	log-sigma-2-mm-3D_firstorder_RootMeanSquared_py*	35.66(8.29)	35.15(8.39)	38.65(7.04)	0.022
210	log-sigma-2-mm-3D_firstorder_Skewness_py*	-0.20(0.65)	-0.24(0.63)	0.02(0.72)	0.015
211	log-sigma-2-mm-3D_firstorder_TotalEnergy_py	17255780.65(13482827.13)	17284021.30(13943203.57)	17087997.91(10505036.48)	0.675
212	log-sigma-2-mm-3D_firstorder_Uniformity_py*	0.20(0.07)	0.21(0.07)	0.18(0.06)	0.029

213	log-sigma-2-mm-3D_firstorder_Variance_py*	1331.92(567.79)	1297.72(567.40)	1535.10(533.99)	0.024
214	log-sigma-2-mm-3D_glcm_Autocorrelation_py*	74.48(15.17)	75.50(15.03)	68.45(14.80)	0.015
215	log-sigma-2-mm-3D_glcm_ClusterProminence_py*	690.89(353.32)	671.79(356.11)	804.36(317.93)	0.043
216	log-sigma-2-mm-3D_glcm_ClusterShade_py	-6.66(24.29)	-7.82(23.66)	0.18(27.10)	0.097
217	log-sigma-2-mm-3D_glcm_ClusterTendency_py*	12.80(5.36)	12.47(5.36)	14.74(5.05)	0.022
218	log-sigma-2-mm-3D_glcm_Contrast_py*	0.89(0.36)	0.87(0.35)	1.02(0.36)	0.024
219	log-sigma-2-mm-3D_glcm_Correlation_py	0.87(0.02)	0.87(0.02)	0.87(0.02)	0.334
220	log-sigma-2-mm-3D_glcm_DifferenceAverage_py*	0.55(0.17)	0.54(0.17)	0.62(0.16)	0.02
221	log-sigma-2-mm-3D_glcm_DifferenceEntropy_py*	1.38(0.25)	1.37(0.25)	1.47(0.20)	0.023
222	log-sigma-2-mm-3D_glcm_DifferenceVariance_py*	0.55(0.17)	0.54(0.17)	0.61(0.15)	0.032
223	log-sigma-2-mm-3D_glcm_Id_py*	0.77(0.06)	0.78(0.06)	0.75(0.06)	0.019
224	log-sigma-2-mm-3D_glcm_Idm_py*	0.76(0.07)	0.76(0.07)	0.73(0.06)	0.019
225	log-sigma-2-mm-3D_glcm_Idmn_py*	1.00(0.00)	1.00(0.00)	1.00(0.00)	0.006
226	log-sigma-2-mm-3D_glcm_Idn_py*	0.97(0.01)	0.97(0.01)	0.96(0.01)	0.007
227	log-sigma-2-mm-3D_glcm_Imc1_py*	-0.39(0.05)	-0.39(0.05)	-0.38(0.04)	0.03
228	log-sigma-2-mm-3D_glcm_Imc2_py	0.94(0.02)	0.93(0.02)	0.94(0.01)	0.768
229	log-sigma-2-mm-3D_glcm_InverseVariance_py*	0.33(0.06)	0.32(0.06)	0.35(0.05)	0.019
230	log-sigma-2-mm-3D_glcm_JointAverage_py*	8.40(0.96)	8.47(0.94)	8.01(0.95)	0.003
231	log-sigma-2-mm-3D_glcm_JointEnergy_py*	0.11(0.07)	0.11(0.07)	0.09(0.05)	0.022
232	log-sigma-2-mm-3D_glcm_JointEntropy_py*	4.41(0.79)	4.36(0.80)	4.69(0.66)	0.024
233	log-sigma-2-mm-3D_glcm_MCC_py	0.87(0.02)	0.87(0.02)	0.87(0.02)	0.612

234	log-sigma-2-mm-3D_glcm_MaximumProbability_py	0.23(0.08)	0.23(0.08)	0.20(0.09)	0.088
235	log-sigma-2-mm-3D_glcm_SumAverage_py*	16.80(1.91)	16.94(1.89)	16.02(1.90)	0.003
236	log-sigma-2-mm-3D_glcm_SumEntropy_py*	3.55(0.52)	3.52(0.53)	3.73(0.42)	0.022
237	log-sigma-2-mm-3D_glcm_SumSquares_py*	3.42(1.43)	3.33(1.42)	3.94(1.34)	0.022
238	log-sigma-2-mm-3D_gldm_DependenceEntropy_py*	4.13(0.54)	4.10(0.55)	4.32(0.43)	0.021
239	log-sigma-2-mm-3D_gldm_DependenceNonUniformity_py	5012.03(3507.57)	5177.96(3646.86)	4026.19(2331.58)	0.135
240	log-sigma-2-mm-3D_gldm_DependenceNonUniformityNormalized_py	0.37(0.04)	0.37(0.05)	0.36(0.03)	0.079
241	log-sigma-2-mm-3D_gldm_DependenceVariance_py	0.56(0.07)	0.56(0.07)	0.59(0.04)	0.107
242	log-sigma-2-mm-3D_gldm_GrayLevelNonUniformity_py	2853.84(2486.16)	2986.62(2599.65)	2064.98(1450.36)	0.076
243	log-sigma-2-mm-3D_gldm_GrayLevelVariance_py*	3.43(1.41)	3.35(1.41)	3.93(1.33)	0.024
244	log-sigma-2-mm-3D_gldm_HighGrayLevelEmphasis_py*	74.90(15.20)	75.91(15.07)	68.93(14.84)	0.015
245	log-sigma-2-mm-3D_gldm_LargeDependenceEmphasis_py*	5.22(0.85)	5.28(0.85)	4.90(0.74)	0.017
246	log-sigma-2-mm-3D_gldm_LargeDependenceHighGrayLevelEmphasis_py*	387.14(103.26)	396.51(102.11)	331.49(93.21)	<0.001
247	log-sigma-2-mm-3D_gldm_LargeDependenceLowGrayLevelEmphasis_py*	0.09(0.04)	0.09(0.05)	0.10(0.04)	0.02
248	log-sigma-2-mm-3D_gldm_LowGrayLevelEmphasis_py*	0.02(0.01)	0.02(0.01)	0.02(0.01)	<0.001

249	log-sigma-2-mm-3D_gldm_SmallDependenceEmphasis_py*	0.38(0.09)	0.37(0.09)	0.41(0.08)	0.016
250	log-sigma-2-mm-3D_gldm_SmallDependenceHighGrayLevelEmphasis_py	28.38(8.37)	28.32(8.47)	28.74(7.86)	0.783
251	log-sigma-2-mm-3D_gldm_SmallDependenceLowGrayLevelEmphasis_py*	0.01(0.00)	0.01(0.00)	0.01(0.00)	<0.001
252	log-sigma-2-mm-3D_glrlm_GrayLevelNonUniformity_py	771.93(464.06)	786.91(477.23)	682.95(369.95)	0.364
253	log-sigma-2-mm-3D_glrlm_GrayLevelNonUniformityNormalized_py	0.15(0.04)	0.15(0.04)	0.14(0.02)	0.061
254	log-sigma-2-mm-3D_glrlm_GrayLevelVariance_py	4.64(1.25)	4.58(1.27)	5.03(1.02)	0.052
255	log-sigma-2-mm-3D_glrlm_HighGrayLevelRunEmphasis_py*	75.68(14.19)	76.60(14.17)	70.24(13.25)	0.007
256	log-sigma-2-mm-3D_glrlm_LongRunEmphasis_py*	14.78(10.63)	15.39(11.03)	11.18(6.87)	0.019
257	log-sigma-2-mm-3D_glrlm_LongRunHighGrayLevelEmphasis_py*	1065.65(776.21)	1119.47(791.52)	745.87(592.80)	<0.001
258	log-sigma-2-mm-3D_glrlm_LongRunLowGrayLevelEmphasis_py	0.26(0.33)	0.27(0.35)	0.22(0.16)	0.495
259	log-sigma-2-mm-3D_glrlm_LowGrayLevelRunEmphasis_py*	0.02(0.01)	0.02(0.01)	0.02(0.01)	<0.001
260	log-sigma-2-mm-3D_glrlm_RunEntropy_py*	5.19(0.23)	5.20(0.23)	5.11(0.19)	0.029

261	log-sigma-2-mm-3D_glrlm_RunLengthNonUniformity_py	1973.58(1480.46)	1956.29(1497.98)	2076.27(1388.40)	0.484
262	log-sigma-2-mm-3D_glrlm_RunLengthNonUniformityNormalized_py*	0.35(0.10)	0.34(0.10)	0.38(0.08)	0.019
263	log-sigma-2-mm-3D_glrlm_RunPercentage_py*	0.43(0.10)	0.42(0.10)	0.46(0.09)	0.017
264	log-sigma-2-mm-3D_glrlm_RunVariance_py*	7.74(5.47)	8.06(5.61)	5.86(4.11)	0.019
265	log-sigma-2-mm-3D_glrlm_ShortRunEmphasis_py*	0.60(0.10)	0.59(0.11)	0.64(0.07)	0.029
266	log-sigma-2-mm-3D_glrlm_ShortRunHighGrayLevelEmphasis_py	45.45(11.31)	45.48(11.59)	45.26(9.59)	0.915
267	log-sigma-2-mm-3D_glrlm_ShortRunLowGrayLevelEmphasis_py*	0.01(0.00)	0.01(0.00)	0.02(0.00)	<0.001
268	log-sigma-2-mm-3D_glszm_GrayLevelNonUniformity_py	771.93(464.06)	786.91(477.23)	682.95(369.95)	0.364
269	log-sigma-2-mm-3D_glszm_GrayLevelNonUniformityNormalized_py	0.15(0.04)	0.15(0.04)	0.14(0.02)	0.061
270	log-sigma-2-mm-3D_glszm_GrayLevelVariance_py	4.64(1.25)	4.58(1.27)	5.03(1.02)	0.052
271	log-sigma-2-mm-3D_glszm_HighGrayLevelZoneEmphasis_py*	75.68(14.19)	76.60(14.17)	70.24(13.25)	0.007
272	log-sigma-2-mm-3D_glszm_LargeAreaEmphasis_py*	14.78(10.63)	15.39(11.03)	11.18(6.87)	0.019
273	log-sigma-2-mm-3D_glszm_LargeAreaHighGrayLevelEmphasis_py*	1065.65(776.21)	1119.47(791.52)	745.87(592.80)	<0.001

274	log-sigma-2-mm-3D_glszm_LargeAreaLowGrayLevelEmphasis_py	0.26(0.33)	0.27(0.35)	0.22(0.16)	0.495
275	log-sigma-2-mm-3D_glszm_LowGrayLevelZoneEmphasis_py*	0.02(0.01)	0.02(0.01)	0.02(0.01)	<0.001
276	log-sigma-2-mm-3D_glszm_SizeZoneNonUniformity_py	1973.58(1480.46)	1956.29(1497.98)	2076.27(1388.40)	0.484
277	log-sigma-2-mm-3D_glszm_SizeZoneNonUniformityNormalized_py*	0.35(0.10)	0.34(0.10)	0.38(0.08)	0.019
278	log-sigma-2-mm-3D_glszm_SmallAreaEmphasis_py*	0.60(0.10)	0.59(0.11)	0.64(0.07)	0.029
279	log-sigma-2-mm-3D_glszm_SmallAreaHighGrayLevelEmphasis_py	45.45(11.31)	45.48(11.59)	45.26(9.59)	0.915
280	log-sigma-2-mm-3D_glszm_SmallAreaLowGrayLevelEmphasis_py*	0.01(0.00)	0.01(0.00)	0.02(0.00)	<0.001
281	log-sigma-2-mm-3D_glszm_ZoneEntropy_py*	5.19(0.23)	5.20(0.23)	5.11(0.19)	0.029
282	log-sigma-2-mm-3D_glszm_ZonePercentage_py*	0.43(0.10)	0.42(0.10)	0.46(0.09)	0.017
283	log-sigma-2-mm-3D_glszm_ZoneVariance_py*	7.74(5.47)	8.06(5.61)	5.86(4.11)	0.019
284	log-sigma-2-mm-3D_ngtdm_Busyness_py	3.64(2.26)	3.68(2.35)	3.36(1.66)	0.679
285	log-sigma-2-mm-3D_ngtdm_Coarseness_py	0.00(0.00)	0.00(0.00)	0.00(0.00)	0.145
286	log-sigma-2-mm-3D_ngtdm_Complexity_py	30.82(8.46)	30.71(8.60)	31.43(7.64)	0.65
287	log-sigma-2-mm-3D_ngtdm_Contrast_py*	0.01(0.01)	0.01(0.01)	0.01(0.01)	0.008
288	log-sigma-2-mm-3D_ngtdm_Strength_py	0.24(0.14)	0.23(0.14)	0.25(0.15)	0.577

289	log-sigma-3-mm-3D_firstorder_10Percentile_py	-39.90(14.26)	-39.27(14.80)	-43.60(9.94)	0.132
290	log-sigma-3-mm-3D_firstorder_90Percentile_py	47.49(11.36)	46.95(11.27)	50.72(11.47)	0.073
291	log-sigma-3-mm-3D_firstorder_Energy_py	16644058.30(13699708.71)	16828564.90(14359471.69)	15547872.07(8897501.73)	0.835
292	log-sigma-3-mm-3D_firstorder_Entropy_py	2.74(0.38)	2.72(0.39)	2.84(0.31)	0.093
293	log-sigma-3-mm-3D_firstorder_InterquartileRange_py	40.56(16.12)	39.80(16.11)	45.07(15.64)	0.078
294	log-sigma-3-mm-3D_firstorder_Kurtosis_py	4.26(2.90)	4.35(3.03)	3.74(1.85)	0.059
295	log-sigma-3-mm-3D_firstorder_Maximum_py	119.85(19.13)	119.44(19.63)	122.34(15.90)	0.413
296	log-sigma-3-mm-3D_firstorder_MeanAbsoluteDeviation_py	26.37(7.29)	26.00(7.39)	28.58(6.38)	0.056
297	log-sigma-3-mm-3D_firstorder_Mean_py	3.75(3.11)	3.84(3.07)	3.19(3.34)	0.182
298	log-sigma-3-mm-3D_firstorder_Median_py	3.51(5.77)	3.73(5.50)	2.17(7.17)	0.119
299	log-sigma-3-mm-3D_firstorder_Minimum_py	-124.16(20.87)	-125.17(20.72)	-118.17(21.07)	0.07
300	log-sigma-3-mm-3D_firstorder_Range_py	244.01(30.77)	244.60(31.75)	240.52(24.21)	0.475
301	log-sigma-3-mm-3D_firstorder_RobustMeanAbsoluteDeviation_py	17.37(6.24)	17.06(6.25)	19.20(5.97)	0.065
302	log-sigma-3-mm-3D_firstorder_RootMeanSquared_py	34.98(7.55)	34.61(7.73)	37.20(6.04)	0.065
303	log-sigma-3-mm-3D_firstorder_Skewness_py	-0.17(0.60)	-0.20(0.56)	0.02(0.74)	0.071
304	log-sigma-3-mm-3D_firstorder_TotalEnergy_py	16644058.30(13699708.71)	16828564.90(14359471.69)	15547872.07(8897501.73)	0.835

305	log-sigma-3-mm-3D_firstorder_Uniformity_py	0.20(0.07)	0.20(0.07)	0.18(0.06)	0.103
306	log-sigma-3-mm-3D_firstorder_Variance_py	1256.96(504.32)	1233.21(512.28)	1398.04(434.44)	0.078
307	log-sigma-3-mm-3D_glcm_Autocorrelation_py	59.53(15.67)	60.33(15.55)	54.73(15.72)	0.095
308	log-sigma-3-mm-3D_glcm_ClusterProminence_py	565.53(290.75)	554.22(298.54)	632.72(231.71)	0.146
309	log-sigma-3-mm-3D_glcm_ClusterShade_py	-5.92(21.67)	-6.87(20.96)	-0.27(25.08)	0.132
310	log-sigma-3-mm-3D_glcm_ClusterTendency_py	12.40(4.92)	12.17(5.00)	13.80(4.24)	0.074
311	log-sigma-3-mm-3D_glcm_Contrast_py*	0.50(0.17)	0.49(0.17)	0.55(0.16)	0.045
312	log-sigma-3-mm-3D_glcm_Correlation_py	0.92(0.01)	0.92(0.02)	0.92(0.01)	0.569
313	log-sigma-3-mm-3D_glcm_DifferenceAverage_py*	0.41(0.12)	0.40(0.12)	0.44(0.11)	0.04
314	log-sigma-3-mm-3D_glcm_DifferenceEntropy_py*	1.11(0.18)	1.10(0.18)	1.16(0.14)	0.041
315	log-sigma-3-mm-3D_glcm_DifferenceVariance_py	0.32(0.07)	0.31(0.08)	0.34(0.06)	0.05
316	log-sigma-3-mm-3D_glcm_Id_py*	0.81(0.05)	0.81(0.05)	0.79(0.05)	0.039
317	log-sigma-3-mm-3D_glcm_Idm_py*	0.81(0.05)	0.81(0.05)	0.79(0.05)	0.039
318	log-sigma-3-mm-3D_glcm_Idmn_py*	1.00(0.00)	1.00(0.00)	1.00(0.00)	0.008
319	log-sigma-3-mm-3D_glcm_Idn_py*	0.97(0.01)	0.97(0.01)	0.97(0.01)	0.012
320	log-sigma-3-mm-3D_glcm_Imc1_py*	-0.49(0.04)	-0.49(0.04)	-0.48(0.04)	0.044
321	log-sigma-3-mm-3D_glcm_Imc2_py	0.96(0.01)	0.96(0.01)	0.96(0.01)	0.735
322	log-sigma-3-mm-3D_glcm_InverseVariance_py*	0.33(0.08)	0.33(0.08)	0.36(0.07)	0.038
323	log-sigma-3-mm-3D_glcm_JointAverage_py*	7.44(1.10)	7.50(1.08)	7.07(1.19)	0.042
324	log-sigma-3-mm-3D_glcm_JointEnergy_py	0.11(0.06)	0.11(0.06)	0.09(0.05)	0.079

325	log-sigma-3-mm-3D_glcm_JointEntropy_py	4.13(0.66)	4.10(0.67)	4.33(0.54)	0.069
326	log-sigma-3-mm-3D_glcm_MCC_py	0.92(0.01)	0.92(0.01)	0.92(0.01)	0.318
327	log-sigma-3-mm-3D_glcm_MaximumProbability_py	0.22(0.09)	0.23(0.09)	0.21(0.10)	0.224
328	log-sigma-3-mm-3D_glcm_SumAverage_py*	14.88(2.21)	15.00(2.16)	14.15(2.38)	0.042
329	log-sigma-3-mm-3D_glcm_SumEntropy_py	3.57(0.49)	3.54(0.50)	3.70(0.39)	0.076
330	log-sigma-3-mm-3D_glcm_SumSquares_py	3.22(1.27)	3.16(1.29)	3.59(1.10)	0.072
331	log-sigma-3-mm-3D_gldm_DependenceEntropy_py	4.13(0.52)	4.10(0.54)	4.27(0.42)	0.085
332	log-sigma-3-mm-3D_gldm_DependenceNonUniformity_py	5259.92(3764.84)	5442.64(3921.54)	4174.33(2418.39)	0.135
333	log-sigma-3-mm-3D_gldm_DependenceNonUniformityNormalized_py	0.39(0.06)	0.39(0.06)	0.37(0.04)	0.056
334	log-sigma-3-mm-3D_gldm_DependenceVariance_py	0.54(0.08)	0.53(0.09)	0.56(0.05)	0.147
335	log-sigma-3-mm-3D_gldm_GrayLevelNonUniformity_py	2709.28(2332.36)	2822.02(2439.53)	2039.50(1396.44)	0.112
336	log-sigma-3-mm-3D_gldm_GrayLevelVariance_py	3.23(1.26)	3.17(1.27)	3.58(1.08)	0.078
337	log-sigma-3-mm-3D_gldm_HighGrayLevelEmphasis_py	59.71(15.69)	60.51(15.58)	54.95(15.73)	0.095
338	log-sigma-3-mm-3D_gldm_LargeDependenceEmphasis_py*	5.63(0.78)	5.68(0.79)	5.37(0.69)	0.034
339	log-sigma-3-mm-3D_gldm_LargeDependenceHighGrayLevelEmphasis_py*	335.17(102.67)	342.44(100.97)	291.99(103.55)	0.008

340	log-sigma-3-mm-3D_gldm_LargeDependenceLowGrayLevelEmphasis_py	0.15(0.10)	0.14(0.09)	0.17(0.14)	0.078
341	log-sigma-3-mm-3D_gldm_LowGrayLevelEmphasis_py*	0.03(0.01)	0.03(0.01)	0.03(0.02)	0.004
342	log-sigma-3-mm-3D_gldm_SmallDependenceEmphasis_py*	0.33(0.07)	0.33(0.07)	0.36(0.07)	0.04
343	log-sigma-3-mm-3D_gldm_SmallDependenceHighGrayLevelEmphasis_py	19.76(6.63)	19.76(6.78)	19.73(5.80)	0.979
344	log-sigma-3-mm-3D_gldm_SmallDependenceLowGrayLevelEmphasis_py*	0.01(0.00)	0.01(0.00)	0.01(0.00)	<0.001
345	log-sigma-3-mm-3D_glrlm_GrayLevelNonUniformity_py	705.77(415.47)	717.13(424.32)	638.31(356.53)	0.41
346	log-sigma-3-mm-3D_glrlm_GrayLevelNonUniformityNormalized_py	0.15(0.04)	0.15(0.04)	0.14(0.02)	0.141
347	log-sigma-3-mm-3D_glrlm_GrayLevelVariance_py	3.94(1.01)	3.90(1.05)	4.19(0.71)	0.12
348	log-sigma-3-mm-3D_glrlm_HighGrayLevelRunEmphasis_py	59.54(14.38)	60.24(14.35)	55.44(14.08)	0.096
349	log-sigma-3-mm-3D_glrlm_LongRunEmphasis_py*	20.30(14.95)	21.08(15.54)	15.68(9.75)	0.035
350	log-sigma-3-mm-3D_glrlm_LongRunHighGrayLevelEmphasis_py*	1160.70(865.71)	1217.90(883.19)	820.85(668.52)	<0.001
351	log-sigma-3-mm-3D_glrlm_LongRunLowGrayLevelEmphasis_py	0.52(0.85)	0.52(0.86)	0.52(0.78)	0.278

352	log-sigma-3-mm-3D_glrlm_LowGrayLevelRunEmphasis_py*	0.03(0.01)	0.03(0.01)	0.03(0.02)	0.014
353	log-sigma-3-mm-3D_glrlm_RunEntropy_py*	5.34(0.28)	5.36(0.28)	5.25(0.24)	0.036
354	log-sigma-3-mm-3D_glrlm_RunLengthNonUniformity_py	1518.16(1251.26)	1514.22(1287.89)	1541.61(1022.66)	0.616
355	log-sigma-3-mm-3D_glrlm_RunLengthNonUniformityNormalized_py*	0.30(0.09)	0.29(0.09)	0.33(0.07)	0.048
356	log-sigma-3-mm-3D_glrlm_RunPercentage_py*	0.38(0.09)	0.37(0.09)	0.41(0.08)	0.035
357	log-sigma-3-mm-3D_glrlm_RunVariance_py*	11.05(7.81)	11.45(7.99)	8.68(6.19)	0.038
358	log-sigma-3-mm-3D_glrlm_ShortRunEmphasis_py	0.55(0.11)	0.54(0.11)	0.58(0.07)	0.076
359	log-sigma-3-mm-3D_glrlm_ShortRunHighGrayLevelEmphasis_py	32.36(10.08)	32.37(10.37)	32.26(8.33)	0.95
360	log-sigma-3-mm-3D_glrlm_ShortRunLowGrayLevelEmphasis_py*	0.02(0.01)	0.02(0.01)	0.02(0.01)	0.002
361	log-sigma-3-mm-3D_glszm_GrayLevelNonUniformity_py	705.77(415.47)	717.13(424.32)	638.31(356.53)	0.41
362	log-sigma-3-mm-3D_glszm_GrayLevelNonUniformityNormalized_py	0.15(0.04)	0.15(0.04)	0.14(0.02)	0.141
363	log-sigma-3-mm-3D_glszm_GrayLevelVariance_py	3.94(1.01)	3.90(1.05)	4.19(0.71)	0.12
364	log-sigma-3-mm-3D_glszm_HighGrayLevelZoneEmphasis_py	59.54(14.38)	60.24(14.35)	55.44(14.08)	0.096

365	log-sigma-3-mm-3D_glszm_LargeAreaEmphasis_py*	20.30(14.95)	21.08(15.54)	15.68(9.75)	0.035
366	log-sigma-3-mm-3D_glszm_LargeAreaHighGrayLevelEmphasis_py*	1160.70(865.71)	1217.90(883.19)	820.85(668.52)	<0.001
367	log-sigma-3-mm-3D_glszm_LargeAreaLowGrayLevelEmphasis_py	0.52(0.85)	0.52(0.86)	0.52(0.78)	0.278
368	log-sigma-3-mm-3D_glszm_LowGrayLevelZoneEmphasis_py*	0.03(0.01)	0.03(0.01)	0.03(0.02)	0.014
369	log-sigma-3-mm-3D_glszm_SizeZoneNonUniformity_py	1518.16(1251.26)	1514.22(1287.89)	1541.61(1022.66)	0.616
370	log-sigma-3-mm-3D_glszm_SizeZoneNonUniformityNormalized_py*	0.30(0.09)	0.29(0.09)	0.33(0.07)	0.048
371	log-sigma-3-mm-3D_glszm_SmallAreaEmphasis_py	0.55(0.11)	0.54(0.11)	0.58(0.07)	0.076
372	log-sigma-3-mm-3D_glszm_SmallAreaHighGrayLevelEmphasis_py	32.36(10.08)	32.37(10.37)	32.26(8.33)	0.95
373	log-sigma-3-mm-3D_glszm_SmallAreaLowGrayLevelEmphasis_py*	0.02(0.01)	0.02(0.01)	0.02(0.01)	0.002
374	log-sigma-3-mm-3D_glszm_ZoneEntropy_py*	5.34(0.28)	5.36(0.28)	5.25(0.24)	0.036
375	log-sigma-3-mm-3D_glszm_ZonePercentage_py*	0.38(0.09)	0.37(0.09)	0.41(0.08)	0.035
376	log-sigma-3-mm-3D_glszm_ZoneVariance_py*	11.05(7.81)	11.45(7.99)	8.68(6.19)	0.038
377	log-sigma-3-mm-3D_ngtdm_Busyness_py	4.06(2.44)	4.09(2.53)	3.88(1.87)	0.99
378	log-sigma-3-mm-3D_ngtdm_Coarseness_py	0.00(0.00)	0.00(0.00)	0.00(0.00)	0.167

379	log-sigma-3-mm-3D_ngtdm_Complexity_py	18.19(5.02)	18.20(5.16)	18.13(4.16)	0.938
380	log-sigma-3-mm-3D_ngtdm_Contrast_py*	0.01(0.00)	0.01(0.00)	0.01(0.00)	0.015
381	log-sigma-3-mm-3D_ngtdm_Strength_py	0.21(0.13)	0.21(0.12)	0.24(0.16)	0.458
382	lbp-2D_firstorder_10Percentile_py*	1.60(0.72)	1.65(0.73)	1.29(0.58)	0.006
383	lbp-2D_firstorder_90Percentile_py*	8.49(0.67)	8.44(0.69)	8.79(0.41)	0.004
384	lbp-2D_firstorder_Energy_py	365739.89(229794.38)	372330.27(236581.25)	326585.29(182431.03)	0.427
385	lbp-2D_firstorder_InterquartileRange_py*	3.10(1.09)	3.03(1.07)	3.53(1.11)	0.01
386	lbp-2D_firstorder_Kurtosis_py*	2.64(0.63)	2.69(0.64)	2.38(0.49)	0.004
387	lbp-2D_firstorder_MeanAbsoluteDeviation_py*	1.89(0.32)	1.86(0.32)	2.04(0.31)	0.003
388	lbp-2D_firstorder_Mean_py*	4.68(0.16)	4.67(0.16)	4.74(0.15)	0.016
389	lbp-2D_firstorder_Median_py*	4.12(0.33)	4.10(0.31)	4.24(0.43)	0.031
390	lbp-2D_firstorder_RobustMeanAbsoluteDeviation_py*	1.59(0.48)	1.55(0.48)	1.82(0.41)	0.005
391	lbp-2D_firstorder_RootMeanSquared_py*	5.25(0.28)	5.23(0.27)	5.37(0.26)	0.006
392	lbp-2D_firstorder_Skewness_py*	0.24(0.20)	0.26(0.20)	0.15(0.18)	0.004
393	lbp-2D_firstorder_TotalEnergy_py	365739.89(229794.38)	372330.27(236581.25)	326585.29(182431.03)	0.427
394	lbp-2D_firstorder_Variance_py*	5.75(1.43)	5.64(1.42)	6.43(1.38)	0.003
395	lbp-2D_gldm_DependenceEntropy_py	0.23(0.07)	0.23(0.08)	0.25(0.07)	0.154
396	lbp-2D_gldm_DependenceNonUniformity_py	12555.27(8327.81)	12894.16(8629.95)	10541.88(5947.02)	0.241
397	lbp-2D_gldm_DependenceNonUniformityNormalized_py	0.93(0.03)	0.93(0.03)	0.92(0.03)	0.158

398	lbp-2D_gldm_DependenceVariance_py	0.04(0.02)	0.04(0.02)	0.04(0.01)	0.15
399	lbp-2D_gldm_GrayLevelNonUniformity_py	13353.00(8556.68)	13696.30(8859.18)	11313.41(6191.92)	0.245
400	lbp-2D_gldm_LargeDependenceEmphasis_py	8.81(0.08)	8.81(0.08)	8.79(0.08)	0.157
401	lbp-2D_gldm_LargeDependenceHighGrayLevelEmphasis_py	8.81(0.08)	8.81(0.08)	8.79(0.08)	0.157
402	lbp-2D_gldm_LargeDependenceLowGrayLevelEmphasis_py	8.81(0.08)	8.81(0.08)	8.79(0.08)	0.157
403	lbp-2D_gldm_SmallDependenceEmphasis_py	0.12(0.00)	0.12(0.00)	0.12(0.00)	0.141
404	lbp-2D_gldm_SmallDependenceHighGrayLevelEmphasis_py	0.12(0.00)	0.12(0.00)	0.12(0.00)	0.141
405	lbp-2D_gldm_SmallDependenceLowGrayLevelEmphasis_py	0.12(0.00)	0.12(0.00)	0.12(0.00)	0.141
406	lbp-2D_glrlm_GrayLevelNonUniformity_py	208.76(76.62)	209.80(76.39)	202.59(78.79)	0.933
407	lbp-2D_glrlm_LongRunEmphasis_py	6112.27(5986.46)	6399.24(6323.92)	4407.28(2849.81)	0.164
408	lbp-2D_glrlm_LongRunHighGrayLevelEmphasis_py	6112.27(5986.46)	6399.24(6323.92)	4407.28(2849.81)	0.164
409	lbp-2D_glrlm_LongRunLowGrayLevelEmphasis_py	6112.27(5986.46)	6399.24(6323.92)	4407.28(2849.81)	0.164
410	lbp-2D_glrlm_RunEntropy_py	5.97(0.57)	6.00(0.58)	5.81(0.49)	0.071
411	lbp-2D_glrlm_RunLengthNonUniformity_py	4.43(1.81)	4.36(1.76)	4.91(2.04)	0.1

412	lbp-2D_glrlm_RunLengthNonUniformityNormalized_py*	0.02(0.01)	0.02(0.01)	0.03(0.01)	0.033
413	lbp-2D_glrlm_RunPercentage_py	0.02(0.01)	0.02(0.01)	0.02(0.01)	0.157
414	lbp-2D_glrlm_RunVariance_py	1612.73(1889.47)	1692.61(2000.31)	1138.14(883.76)	0.389
415	lbp-2D_glrlm_ShortRunEmphasis_py	0.02(0.01)	0.02(0.01)	0.02(0.01)	0.588
416	lbp-2D_glrlm_ShortRunHighGrayLevelEmphasis_py	0.02(0.01)	0.02(0.01)	0.02(0.01)	0.588
417	lbp-2D_glrlm_ShortRunLowGrayLevelEmphasis_py	0.02(0.01)	0.02(0.01)	0.02(0.01)	0.588
418	lbp-2D_glszm_GrayLevelNonUniformity_py	208.76(76.62)	209.80(76.39)	202.59(78.79)	0.933
419	lbp-2D_glszm_LargeAreaEmphasis_py	6112.27(5986.46)	6399.24(6323.92)	4407.28(2849.81)	0.164
420	lbp-2D_glszm_LargeAreaHighGrayLevelEmphasis_py	6112.27(5986.46)	6399.24(6323.92)	4407.28(2849.81)	0.164
421	lbp-2D_glszm_LargeAreaLowGrayLevelEmphasis_py	6112.27(5986.46)	6399.24(6323.92)	4407.28(2849.81)	0.164
422	lbp-2D_glszm_SizeZoneNonUniformity_py	4.43(1.81)	4.36(1.76)	4.91(2.04)	0.1
423	lbp-2D_glszm_SizeZoneNonUniformityNormalized_py*	0.02(0.01)	0.02(0.01)	0.03(0.01)	0.033
424	lbp-2D_glszm_SmallAreaEmphasis_py	0.02(0.01)	0.02(0.01)	0.02(0.01)	0.588
425	lbp-2D_glszm_SmallAreaHighGrayLevelEmphasis_py	0.02(0.01)	0.02(0.01)	0.02(0.01)	0.588
426	lbp-2D_glszm_SmallAreaLowGrayLevelEmphasis_py	0.02(0.01)	0.02(0.01)	0.02(0.01)	0.588
427	lbp-2D_glszm_ZoneEntropy_py	5.97(0.57)	6.00(0.58)	5.81(0.49)	0.071

428	lbp-2D_glszm_ZonePercentage_py	0.02(0.01)	0.02(0.01)	0.02(0.01)	0.157
429	lbp-2D_glszm_ZoneVariance_py	1612.73(1889.47)	1692.61(2000.31)	1138.14(883.76)	0.389
430	wavelet-H_firstorder_10Percentile_py*	-16.29(22.34)	-14.43(19.59)	-27.35(32.70)	0.012
431	wavelet-H_firstorder_90Percentile_py*	18.32(24.42)	16.38(21.85)	29.86(34.33)	0.016
432	wavelet-H_firstorder_Energy_py	19623763.40(14500673.17)	19341079.56(14405966.11)	21303237.98(15163143.71)	0.432
433	wavelet-H_firstorder_Entropy_py*	1.68(0.25)	1.66(0.25)	1.79(0.22)	0.005
434	wavelet-H_firstorder_InterquartileRange_py*	4.64(2.49)	4.46(2.40)	5.67(2.77)	0.005
435	wavelet-H_firstorder_Kurtosis_py*	14.48(22.71)	15.33(24.39)	9.48(4.24)	0.012
436	wavelet-H_firstorder_Maximum_py	213.38(19.26)	212.61(20.71)	217.93(2.08)	0.282
437	wavelet-H_firstorder_MeanAbsoluteDeviation_py*	16.44(6.67)	15.95(6.64)	19.34(6.22)	0.006
438	wavelet-H_firstorder_Mean_py	0.20(0.22)	0.20(0.23)	0.21(0.19)	0.575
439	wavelet-H_firstorder_Median_py	-0.07(0.07)	-0.07(0.07)	-0.07(0.08)	0.956
440	wavelet-H_firstorder_Minimum_py	-217.93(2.72)	-217.89(2.83)	-218.19(1.97)	0.925
441	wavelet-H_firstorder_Range_py	431.31(20.28)	430.50(21.78)	436.12(3.28)	0.422
442	wavelet-H_firstorder_RobustMeanAbsoluteDeviation_py*	2.77(2.00)	2.64(1.95)	3.52(2.20)	0.005
443	wavelet-H_firstorder_RootMeanSquared_py*	38.26(9.63)	37.55(9.73)	42.47(7.90)	0.006
444	wavelet-H_firstorder_Skewness_py*	-0.04(0.26)	-0.06(0.26)	0.03(0.22)	0.021
445	wavelet-H_firstorder_TotalEnergy_py	19623763.40(14500673.17)	19341079.56(14405966.11)	21303237.98(15163143.71)	0.432
446	wavelet-H_firstorder_Uniformity_py*	0.39(0.05)	0.39(0.05)	0.37(0.04)	0.006
447	wavelet-H_firstorder_Variance_py*	1556.21(701.71)	1504.30(697.89)	1864.59(652.19)	0.005
448	wavelet-H_glcm_Autocorrelation_py	137.36(10.60)	137.56(10.69)	136.19(10.15)	0.075
449	wavelet-H_glcm_ClusterProminence_py*	242.91(123.85)	232.94(121.59)	302.17(122.27)	0.002
450	wavelet-H_glcm_ClusterShade_py	0.24(2.35)	0.13(2.25)	0.93(2.80)	0.065

451	wavelet-H_glcm_ClusterTendency_py*	5.25(2.15)	5.08(2.13)	6.21(2.01)	0.004
452	wavelet-H_glcm_Contrast_py*	10.93(4.84)	10.57(4.82)	13.07(4.48)	0.005
453	wavelet-H_glcm_Correlation_py	-0.34(0.05)	-0.34(0.05)	-0.35(0.02)	0.144
454	wavelet-H_glcm_DifferenceAverage_py*	1.54(0.54)	1.50(0.53)	1.77(0.49)	0.007
455	wavelet-H_glcm_DifferenceEntropy_py*	1.79(0.30)	1.77(0.30)	1.92(0.25)	0.005
456	wavelet-H_glcm_DifferenceVariance_py*	8.26(3.17)	8.02(3.18)	9.69(2.75)	0.004
457	wavelet-H_glcm_Id_py*	0.69(0.06)	0.69(0.06)	0.66(0.06)	0.008
458	wavelet-H_glcm_Idm_py*	0.67(0.07)	0.67(0.07)	0.64(0.06)	0.008
459	wavelet-H_glcm_Idmn_py*	0.98(0.01)	0.98(0.01)	0.98(0.01)	0.005
460	wavelet-H_glcm_Idn_py*	0.95(0.02)	0.95(0.02)	0.94(0.02)	0.006
461	wavelet-H_glcm_Imc1_py*	-0.22(0.04)	-0.22(0.04)	-0.24(0.03)	0.004
462	wavelet-H_glcm_Imc2_py*	0.71(0.08)	0.71(0.08)	0.75(0.06)	0.005
463	wavelet-H_glcm_InverseVariance_py	0.34(0.01)	0.34(0.01)	0.34(0.01)	0.167
464	wavelet-H_glcm_JointAverage_py	11.77(0.45)	11.78(0.45)	11.74(0.43)	0.34
465	wavelet-H_glcm_JointEnergy_py*	0.18(0.04)	0.19(0.04)	0.17(0.03)	0.006
466	wavelet-H_glcm_JointEntropy_py*	2.98(0.39)	2.95(0.39)	3.15(0.34)	0.005
467	wavelet-H_glcm_MCC_py	0.65(0.04)	0.65(0.04)	0.65(0.03)	0.288
468	wavelet-H_glcm_MaximumProbability_py*	0.27(0.04)	0.27(0.04)	0.25(0.04)	0.004
469	wavelet-H_glcm_SumAverage_py	23.54(0.90)	23.56(0.90)	23.47(0.86)	0.34
470	wavelet-H_glcm_SumEntropy_py*	2.12(0.18)	2.11(0.18)	2.20(0.15)	0.005
471	wavelet-H_glcm_SumSquares_py*	4.04(1.75)	3.91(1.74)	4.82(1.62)	0.005
472	wavelet-H_gldm_DependenceEntropy_py*	2.97(0.19)	2.95(0.19)	3.05(0.15)	0.007
473	wavelet-H_gldm_DependenceNonUniformity_py	4785.97(3091.75)	4913.72(3203.44)	4026.96(2207.37)	0.223
474	wavelet-H_gldm_DependenceNonUniformityNormalized_py	0.36(0.02)	0.36(0.02)	0.36(0.01)	0.973
475	wavelet-H_gldm_DependenceVariance_py	0.55(0.04)	0.55(0.04)	0.56(0.03)	0.977

476	wavelet-H_gldm_GrayLevelNonUniformity_py	5258.53(3632.06)	5445.23(3780.16)	4149.28(2329.00)	0.097
477	wavelet-H_gldm_GrayLevelVariance_py*	4.07(1.73)	3.94(1.72)	4.83(1.61)	0.005
478	wavelet-H_gldm_HighGrayLevelEmphasis_py	142.85(11.36)	142.87(11.44)	142.74(11.05)	0.147
479	wavelet-H_gldm_LargeDependenceEmphasis_py*	4.47(0.52)	4.51(0.52)	4.25(0.47)	0.007
480	wavelet-H_gldm_LargeDependenceHighGrayLevelEmphasis_py*	622.15(73.90)	627.83(74.21)	588.40(63.07)	0.004
481	wavelet-H_gldm_LargeDependenceLowGrayLevelEmphasis_py	0.03(0.00)	0.04(0.00)	0.03(0.00)	0.171
482	wavelet-H_gldm_LowGrayLevelEmphasis_py	0.01(0.00)	0.01(0.00)	0.01(0.00)	0.08
483	wavelet-H_gldm_SmallDependenceEmphasis_py*	0.44(0.07)	0.43(0.07)	0.46(0.06)	0.012
484	wavelet-H_gldm_SmallDependenceHighGrayLevelEmphasis_py*	64.88(13.24)	64.18(13.24)	69.05(12.67)	0.047
485	wavelet-H_gldm_SmallDependenceLowGrayLevelEmphasis_py*	0.01(0.00)	0.01(0.00)	0.01(0.00)	0.012
486	wavelet-H_glrlm_GrayLevelNonUniformity_py	2085.91(1363.36)	2147.15(1414.84)	1722.07(940.51)	0.172
487	wavelet-H_glrlm_GrayLevelNonUniformityNormalized_py*	0.31(0.05)	0.32(0.06)	0.29(0.04)	0.007
488	wavelet-H_glrlm_GrayLevelVariance_py*	7.39(2.50)	7.20(2.51)	8.50(2.10)	0.005

489	wavelet-H_glrlm_HighGrayLevelRunEmphasis_py	146.79(11.66)	146.77(11.74)	146.92(11.30)	0.16
490	wavelet-H_glrlm_LongRunEmphasis_py*	6.05(1.66)	6.17(1.69)	5.33(1.31)	0.006
491	wavelet-H_glrlm_LongRunHighGrayLevelEmphasis_py*	840.37(215.96)	857.57(218.98)	738.19(165.79)	0.003
492	wavelet-H_glrlm_LongRunLowGrayLevelEmphasis_py*	0.05(0.01)	0.05(0.01)	0.04(0.01)	0.029
493	wavelet-H_glrlm_LowGrayLevelRunEmphasis_py	0.01(0.00)	0.01(0.00)	0.01(0.00)	0.111
494	wavelet-H_glrlm_RunEntropy_py	3.73(0.09)	3.73(0.09)	3.72(0.09)	0.697
495	wavelet-H_glrlm_RunLengthNonUniformity_py	2561.94(1702.49)	2562.73(1723.16)	2557.19(1598.47)	0.947
496	wavelet-H_glrlm_RunLengthNonUniformityNormalized_py*	0.38(0.08)	0.38(0.08)	0.41(0.07)	0.019
497	wavelet-H_glrlm_RunPercentage_py*	0.51(0.07)	0.51(0.07)	0.54(0.06)	0.008
498	wavelet-H_glrlm_RunVariance_py*	2.02(0.55)	2.06(0.55)	1.78(0.52)	0.007
499	wavelet-H_glrlm_ShortRunEmphasis_py*	0.63(0.08)	0.62(0.08)	0.66(0.06)	0.018
500	wavelet-H_glrlm_ShortRunHighGrayLevelEmphasis_py	95.71(16.67)	94.94(16.91)	100.31(14.54)	0.082
501	wavelet-H_glrlm_ShortRunLowGrayLevelEmphasis_py*	0.01(0.00)	0.01(0.00)	0.01(0.00)	0.038
502	wavelet-H_glszm_GrayLevelNonUniformity_py	2085.91(1363.36)	2147.15(1414.84)	1722.07(940.51)	0.172
503	wavelet-H_glszm_GrayLevelNonUniformityNormalized_py*	0.31(0.05)	0.32(0.06)	0.29(0.04)	0.007

504	wavelet-H_glszm_GrayLevelVariance_py*	7.39(2.50)	7.20(2.51)	8.50(2.10)	0.005
505	wavelet-H_glszm_HighGrayLevelZoneEmphasis_py	146.79(11.66)	146.77(11.74)	146.92(11.30)	0.16
506	wavelet-H_glszm_LargeAreaEmphasis_py*	6.05(1.66)	6.17(1.69)	5.33(1.31)	0.006
507	wavelet-H_glszm_LargeAreaHighGrayLevelEmphasis_py*	840.37(215.96)	857.57(218.98)	738.19(165.79)	0.003
508	wavelet-H_glszm_LargeAreaLowGrayLevelEmphasis_py*	0.05(0.01)	0.05(0.01)	0.04(0.01)	0.029
509	wavelet-H_glszm_LowGrayLevelZoneEmphasis_py	0.01(0.00)	0.01(0.00)	0.01(0.00)	0.111
510	wavelet-H_glszm_SizeZoneNonUniformity_py	2561.94(1702.49)	2562.73(1723.16)	2557.19(1598.47)	0.947
511	wavelet-H_glszm_SizeZoneNonUniformityNormalized_py*	0.38(0.08)	0.38(0.08)	0.41(0.07)	0.019
512	wavelet-H_glszm_SmallAreaEmphasis_py*	0.63(0.08)	0.62(0.08)	0.66(0.06)	0.018
513	wavelet-H_glszm_SmallAreaHighGrayLevelEmphasis_py	95.71(16.67)	94.94(16.91)	100.31(14.54)	0.082
514	wavelet-H_glszm_SmallAreaLowGrayLevelEmphasis_py*	0.01(0.00)	0.01(0.00)	0.01(0.00)	0.038
515	wavelet-H_glszm_ZoneEntropy_py	3.73(0.09)	3.73(0.09)	3.72(0.09)	0.697
516	wavelet-H_glszm_ZonePercentage_py*	0.51(0.07)	0.51(0.07)	0.54(0.06)	0.008
517	wavelet-H_glszm_ZoneVariance_py*	2.02(0.55)	2.06(0.55)	1.78(0.52)	0.007
518	wavelet-H_ngtdm_Busyness_py	12.38(6.91)	12.52(7.09)	11.53(5.77)	0.679
519	wavelet-H_ngtdm_Coarseness_py	0.00(0.00)	0.00(0.00)	0.00(0.00)	0.553

520	wavelet-H_ngtdm_Complexity_py	220.21(97.47)	215.70(97.01)	246.99(97.33)	0.083
521	wavelet-H_ngtdm_Contrast_py*	0.07(0.05)	0.07(0.05)	0.10(0.05)	<0.001
522	wavelet-H_ngtdm_Strength_py	0.09(0.05)	0.09(0.05)	0.09(0.06)	0.779
523	wavelet-L_firstorder_10Percentile_py	-160.81(33.53)	-162.25(26.34)	-152.25(60.75)	0.551
524	wavelet-L_firstorder_90Percentile_py*	128.47(76.79)	124.04(80.02)	154.79(46.38)	0.026
525	wavelet-L_firstorder_Energy_py	265350240.75(183366208.88)	271011252.91(190574985.89)	231717168.49(129851490.90)	0.468
526	wavelet-L_firstorder_Entropy_py	3.32(0.32)	3.30(0.33)	3.39(0.30)	0.137
527	wavelet-L_firstorder_InterquartileRange_py	174.73(112.00)	169.53(112.71)	205.63(103.93)	0.214
528	wavelet-L_firstorder_Kurtosis_py	3.94(4.79)	4.13(5.05)	2.79(2.50)	0.136
529	wavelet-L_firstorder_Maximum_py	218.44(3.29)	218.47(3.33)	218.29(3.09)	0.2
530	wavelet-L_firstorder_MeanAbsoluteDeviation_py	102.24(35.92)	100.67(36.75)	111.56(29.23)	0.16
531	wavelet-L_firstorder_Mean_py*	-34.97(58.83)	-39.85(56.74)	-5.94(63.44)	0.003
532	wavelet-L_firstorder_Median_py*	-55.72(105.97)	-64.48(100.88)	-3.68(121.34)	0.039
533	wavelet-L_firstorder_Minimum_py	-219.45(3.63)	-219.35(3.88)	-220.08(1.18)	0.585
534	wavelet-L_firstorder_Range_py	437.90(5.70)	437.82(6.03)	438.37(3.21)	0.316
535	wavelet-L_firstorder_RobustMeanAbsoluteDeviation_py	79.71(41.64)	78.09(42.27)	89.36(36.78)	0.182
536	wavelet-L_firstorder_RootMeanSquared_py	140.00(13.30)	139.45(13.80)	143.27(9.26)	0.213
537	wavelet-L_firstorder_Skewness_py*	0.71(1.28)	0.82(1.26)	0.08(1.22)	0.002
538	wavelet-L_firstorder_TotalEnergy_py	265350240.75(183366208.88)	271011252.91(190574985.89)	231717168.49(129851490.90)	0.468
539	wavelet-L_firstorder_Uniformity_py	0.13(0.04)	0.13(0.04)	0.12(0.04)	0.184
540	wavelet-L_firstorder_Variance_py	15107.79(6006.14)	14845.15(6165.45)	16668.12(4735.60)	0.187
541	wavelet-L_glcm_Autocorrelation_py*	148.93(77.41)	143.12(75.56)	183.40(80.40)	0.006

542	wavelet-L_glcm_ClusterProminence_py	47608.12(15884.63)	47024.09(16506.76)	51077.92(11078.54)	0.351
543	wavelet-L_glcm_ClusterShade_py	674.30(1383.82)	763.41(1328.57)	144.87(1596.37)	0.104
544	wavelet-L_glcm_ClusterTendency_py	141.69(57.75)	139.24(59.35)	156.26(45.17)	0.203
545	wavelet-L_glcm_Contrast_py*	8.48(3.70)	8.22(3.69)	10.01(3.45)	0.009
546	wavelet-L_glcm_Correlation_py	0.88(0.03)	0.88(0.03)	0.88(0.03)	0.281
547	wavelet-L_glcm_DifferenceAverage_py*	1.37(0.49)	1.34(0.49)	1.58(0.46)	0.007
548	wavelet-L_glcm_DifferenceEntropy_py*	1.94(0.39)	1.91(0.39)	2.10(0.34)	0.007
549	wavelet-L_glcm_DifferenceVariance_py*	6.35(2.36)	6.19(2.38)	7.30(2.03)	0.012
550	wavelet-L_glcm_Id_py*	0.71(0.08)	0.71(0.07)	0.68(0.07)	0.008
551	wavelet-L_glcm_Idm_py*	0.68(0.09)	0.69(0.08)	0.65(0.08)	0.008
552	wavelet-L_glcm_Idmn_py*	0.99(0.01)	0.99(0.01)	0.98(0.01)	0.002
553	wavelet-L_glcm_Idn_py*	0.95(0.02)	0.95(0.02)	0.94(0.02)	0.003
554	wavelet-L_glcm_Imc1_py*	-0.48(0.07)	-0.48(0.07)	-0.46(0.07)	0.039
555	wavelet-L_glcm_Imc2_py	0.98(0.01)	0.98(0.01)	0.97(0.01)	0.144
556	wavelet-L_glcm_InverseVariance_py	0.28(0.05)	0.28(0.05)	0.28(0.04)	0.355
557	wavelet-L_glcm_JointAverage_py*	10.29(3.12)	10.06(3.04)	11.67(3.32)	0.009
558	wavelet-L_glcm_JointEnergy_py	0.06(0.04)	0.07(0.04)	0.06(0.03)	0.106
559	wavelet-L_glcm_JointEntropy_py	5.03(0.64)	5.00(0.64)	5.22(0.61)	0.06
560	wavelet-L_glcm_MCC_py	0.93(0.02)	0.93(0.02)	0.92(0.02)	0.163
561	wavelet-L_glcm_MaximumProbability_py	0.15(0.06)	0.15(0.06)	0.14(0.06)	0.23
562	wavelet-L_glcm_SumAverage_py*	20.58(6.25)	20.12(6.07)	23.34(6.64)	0.009
563	wavelet-L_glcm_SumEntropy_py	4.00(0.39)	3.99(0.39)	4.06(0.38)	0.282
564	wavelet-L_glcm_SumSquares_py	37.54(15.16)	36.86(15.55)	41.57(11.94)	0.173
565	wavelet-L_gldm_DependenceEntropy_py	4.58(0.40)	4.56(0.40)	4.65(0.38)	0.261
566	wavelet-L_gldm_DependenceNonUniformity_py	4849.02(3328.83)	4989.65(3450.30)	4013.48(2358.16)	0.18
567	wavelet-L_gldm_DependenceNonUniformityNormalized_py	0.36(0.03)	0.36(0.03)	0.35(0.03)	0.148

568	wavelet-L_gldm_DependenceVariance_py	0.60(0.07)	0.60(0.07)	0.61(0.05)	0.413
569	wavelet-L_gldm_GrayLevelNonUniformity_py	1822.34(1553.08)	1892.51(1618.90)	1405.49(1002.11)	0.141
570	wavelet-L_gldm_GrayLevelVariance_py	37.75(14.97)	37.10(15.37)	41.64(11.80)	0.189
571	wavelet-L_gldm_HighGrayLevelEmphasis_py*	154.21(77.77)	148.32(76.00)	189.16(80.11)	0.005
572	wavelet-L_gldm_LargeDependenceEmphasis_py*	4.88(0.81)	4.94(0.81)	4.56(0.76)	0.012
573	wavelet-L_gldm_LargeDependenceHighGrayLevelEmphasis_py*	702.88(440.93)	676.95(428.80)	856.90(485.82)	0.026
574	wavelet-L_gldm_LargeDependenceLowGrayLevelEmphasis_py	0.16(0.07)	0.16(0.07)	0.14(0.08)	0.17
575	wavelet-L_gldm_LowGrayLevelEmphasis_py	0.03(0.01)	0.03(0.01)	0.03(0.02)	0.919
576	wavelet-L_gldm_SmallDependenceEmphasis_py*	0.42(0.09)	0.41(0.09)	0.46(0.08)	0.01
577	wavelet-L_gldm_SmallDependenceHighGrayLevelEmphasis_py*	71.45(31.04)	68.78(30.70)	87.35(28.54)	0.001
578	wavelet-L_gldm_SmallDependenceLowGrayLevelEmphasis_py	0.02(0.01)	0.02(0.01)	0.02(0.01)	0.203
579	wavelet-L_glrlm_GrayLevelNonUniformity_py	600.05(378.87)	611.21(391.92)	533.76(285.20)	0.496
580	wavelet-L_glrlm_GrayLevelNonUniformityNormalized_py	0.10(0.02)	0.10(0.02)	0.10(0.02)	0.098
581	wavelet-L_glrlm_GrayLevelVariance_py	42.70(12.22)	42.20(12.74)	45.68(8.07)	0.386

582	wavelet-L_glrlm_HighGrayLevelRunEmphasis_py*	164.61(61.21)	160.28(60.70)	190.33(58.69)	0.008
583	wavelet-L_glrlm_LongRunEmphasis_py*	11.13(7.66)	11.50(7.81)	8.88(6.41)	0.008
584	wavelet-L_glrlm_LongRunHighGrayLevelEmphasis_py	1449.29(1191.84)	1425.54(1174.18)	1590.43(1301.58)	0.464
585	wavelet-L_glrlm_LongRunLowGrayLevelEmphasis_py*	0.37(0.39)	0.39(0.41)	0.27(0.25)	0.014
586	wavelet-L_glrlm_LowGrayLevelRunEmphasis_py	0.04(0.01)	0.04(0.01)	0.04(0.02)	0.852
587	wavelet-L_glrlm_RunEntropy_py*	5.33(0.30)	5.35(0.30)	5.22(0.27)	0.022
588	wavelet-L_glrlm_RunLengthNonUniformity_py	2504.34(1704.19)	2472.92(1681.74)	2690.99(1847.54)	0.588
589	wavelet-L_glrlm_RunLengthNonUniformityNormalized_py*	0.41(0.10)	0.40(0.11)	0.45(0.09)	0.019
590	wavelet-L_glrlm_RunPercentage_py*	0.47(0.10)	0.46(0.10)	0.51(0.09)	0.011
591	wavelet-L_glrlm_RunVariance_py*	5.74(4.66)	5.95(4.77)	4.45(3.77)	0.012
592	wavelet-L_glrlm_ShortRunEmphasis_py*	0.66(0.10)	0.65(0.10)	0.69(0.08)	0.02
593	wavelet-L_glrlm_ShortRunHighGrayLevelEmphasis_py*	116.08(38.34)	113.29(38.81)	132.70(31.00)	0.006
594	wavelet-L_glrlm_ShortRunLowGrayLevelEmphasis_py	0.03(0.01)	0.03(0.01)	0.03(0.01)	0.221
595	wavelet-L_glszm_GrayLevelNonUniformity_py	600.05(378.87)	611.21(391.92)	533.76(285.20)	0.496
596	wavelet-L_glszm_GrayLevelNonUniformityNormalized_py	0.10(0.02)	0.10(0.02)	0.10(0.02)	0.098

597	wavelet-L_glszm_GrayLevelVariance_py	42.70(12.22)	42.20(12.74)	45.68(8.07)	0.386
598	wavelet-L_glszm_HighGrayLevelZoneEmphasis_py*	164.61(61.21)	160.28(60.70)	190.33(58.69)	0.008
599	wavelet-L_glszm_LargeAreaEmphasis_py*	11.13(7.66)	11.50(7.81)	8.88(6.41)	0.008
600	wavelet-L_glszm_LargeAreaHighGrayLevelEmphasis_py	1449.29(1191.84)	1425.54(1174.18)	1590.43(1301.58)	0.464
601	wavelet-L_glszm_LargeAreaLowGrayLevelEmphasis_py*	0.37(0.39)	0.39(0.41)	0.27(0.25)	0.014
602	wavelet-L_glszm_LowGrayLevelZoneEmphasis_py	0.04(0.01)	0.04(0.01)	0.04(0.02)	0.852
603	wavelet-L_glszm_SizeZoneNonUniformity_py	2504.34(1704.19)	2472.92(1681.74)	2690.99(1847.54)	0.588
604	wavelet-L_glszm_SizeZoneNonUniformityNormalized_py*	0.41(0.10)	0.40(0.11)	0.45(0.09)	0.019
605	wavelet-L_glszm_SmallAreaEmphasis_py*	0.66(0.10)	0.65(0.10)	0.69(0.08)	0.02
606	wavelet-L_glszm_SmallAreaHighGrayLevelEmphasis_py*	116.08(38.34)	113.29(38.81)	132.70(31.00)	0.006
607	wavelet-L_glszm_SmallAreaLowGrayLevelEmphasis_py	0.03(0.01)	0.03(0.01)	0.03(0.01)	0.221
608	wavelet-L_glszm_ZoneEntropy_py*	5.33(0.30)	5.35(0.30)	5.22(0.27)	0.022
609	wavelet-L_glszm_ZonePercentage_py*	0.47(0.10)	0.46(0.10)	0.51(0.09)	0.011
610	wavelet-L_glszm_ZoneVariance_py*	5.74(4.66)	5.95(4.77)	4.45(3.77)	0.012
611	wavelet-L_ngtdm_Busyness_py	3.44(2.19)	3.51(2.23)	3.01(1.93)	0.23
612	wavelet-L_ngtdm_Coarseness_py	0.00(0.00)	0.00(0.00)	0.00(0.00)	0.917

613	wavelet-L_ngtdm_Complexity_py	217.01(73.25)	213.51(73.91)	237.79(66.41)	0.074
614	wavelet-L_ngtdm_Contrast_py*	0.16(0.09)	0.15(0.09)	0.20(0.10)	0.006
615	wavelet-L_ngtdm_Strength_py	0.52(0.36)	0.52(0.35)	0.55(0.42)	0.756
